# Effects of Model Choice, Corpus Context, and Post Hoc Correction on Layer-Level Embedding Degradation in Clinical Document Retrieval: Experimental Study

**DOI:** 10.2196/99639

**Published:** 2026-07-29

**Authors:** Yngve Mikkelsen

**Affiliations:** 1 Saïd Business School University of Oxford Oxford, England United Kingdom

**Keywords:** anisotropy, clinical decision support, clinical informatics, embedding geometry, retrieval-augmented generation, transformer layers, ZCA whitening

## Abstract

**Background:**

Clinical retrieval-augmented generation depends on embedding models. A companion study found that non–retrieval-trained encoders underperformed retrieval-trained general-purpose embeddings and produced near-degenerate embedding geometry, but did not localize the architectural origin, separate training-domain from training-objective effects, or test whether the degradation can be corrected without retraining.

**Objective:**

This study aimed to (1) characterize layer-wise retrieval and geometric trajectories across 13 transformer configurations on clinical documents, (2) separate training-objective from training-domain effects through matched architectural comparisons, (3) reanalyze the panel under a per-query layer-wise linear mixed-effects (LME) framework, and (4) evaluate deployment-relevant post hoc geometric correction.

**Methods:**

Layer-wise embeddings were extracted from 13 transformer configurations on 3 clinical corpora (n=100 documents each: MTSamples, PMC-Patients, and Mistral-7B-Instruct–generated synthetic notes) under 2 query formats (keyword and natural-language via GPT-4o). Retrieval performance (mean reciprocal rank at cutoff 10 [MRR@10] and recall at cutoff 10 [recall@10]) and geometric properties (participation ratio, average pairwise cosine, and anisotropy) were measured at every layer. A per-query layer-wise LME model was fit independently per configuration. Corpus-only zero-phase component analysis (ZCA) whitening was evaluated as the primary deployment-relevant intervention, with 5-fold cross-validation, an epsilon sweep, a lexical-overlap audit, and a chunking sensitivity analysis.

**Results:**

Document embeddings clustered into 3 anisotropy tiers: extreme (average pairwise cosine >0.92) for non–retrieval-trained encoders and large language models (LLMs), moderate (0.65-0.92) for general retrievers and most LLMs, and reduced (<0.65) for BioLORD-2023, instruction-tuned E5-Mistral-7B, and Nomic-embed-text-nopfx. The per-query random-slope LME identified 2 layer-depth patterns: classical degradation with depth in 3 non–retrieval-trained encoders (all *P*<.001), vs net improvement with depth in the remaining 10 models (all *P*<.001), with the strongest negative coefficients in decoder LLMs. Matched-contrast tests confirmed significant training-objective × layer-depth interactions in all 3 matched pairs (all *P*<.001). Corpus-only ZCA whitening produced a 2-tier pattern under 5-fold cross-validation: tier 2 non–retrieval-trained models showed positive ΔMRR@10 (+0.066 to +0.304), while tier 1 retrieval-trained models showed negative ΔMRR@10 (–0.021 to –0.051). Best Match 25-vs-embedding Spearman rank correlations spanned –0.02 to 0.37, indicating substantial nonlexical contribution to retrieval. Same-source ranking stability replicated at 4-5× corpus scale (ρ=0.952 for PMC-500, ρ=0.929 for MTSamples-400) for the BERT-scale subset.

**Conclusions:**

Anisotropy in transformer embeddings is widespread across architectural classes and is lower in configurations with retrieval-specific training. Corpus-only ZCA whitening is a deployment-compatible, retraining-free post hoc correction candidate that improved retrieval for non–retrieval-trained models on this controlled benchmark but requires target-corpus validation before clinical deployment. The matched-comparison evidence supports training objective rather than training domain as the stronger explanatory axis, though residual confounding is not eliminated. The principal contribution is mechanistic: layer-level localization of embedding degradation and the geometric basis for the 2-tier intervention response.

## Introduction

### Overview

Retrieval-augmented generation (RAG) is the dominant approach for grounding large language model (LLM) outputs in verifiable evidence sources [1,2]. In a clinical RAG pipeline, the embedding model transforms queries and documents into vector representations, and retrieval quality depends on the geometry of the resulting embedding space. When embeddings cluster in a narrow region of the vector space, a phenomenon known as anisotropy, cosine similarity becomes less discriminative, and retrieval may fail regardless of downstream generation quality.

Retrieval failure in clinical RAG settings may affect downstream outputs consumed by practitioners or clinical decision support systems. This study does not evaluate clinical task performance, patient outcomes, or production electronic health record (EHR) deployment; it addresses the upstream question of where in the model architecture retrieval performance is determined and whether geometric properties can be corrected without retraining.

### Companion Study and Motivating Context

A recently accepted companion benchmark study [3] evaluated 10 embedding models across 3 clinical corpora and 294 experimental conditions. Two findings motivated the present analysis. First, model choice and clinical-context variables (corpus type and query format) contributed comparably to variance in mean reciprocal rank at cutoff 10 (MRR@10). Second, domain-specific models, BioBERT [4] and ClinicalBERT [5], produced near-degenerate embedding geometry, with pairwise cosine similarities exceeding 0.90, whereas general-purpose models (Beijing Academy of Artificial Intelligence General Embedding [BGE]-base [6], General Text Embeddings [GTE]-base [7], Nomic-embed-text [8]) achieved higher MRR@10 and more dispersed geometry. The companion study established that domain-pretrained encoders underperform but did not localize *where* in the network the gap appears, *what* causes it, or *whether* it can be corrected.

Comparing BERT-architecture domain encoders with retrieval-trained text-embedding models can conflate training domain (biomedical vs general) with training objective (retrieval-trained vs not). The present analysis therefore adds matched architectural controls to separate these dimensions as much as possible within the public model space.

### Scope and Demarcation

Retrieval failures in production systems arise from multiple sources beyond embedding model behavior: corpus heterogeneity, query formulation, chunking strategy, indexing choices, and vocabulary mismatch between queries and documents. This study isolates the embedding model’s contribution by holding corpus, query generation, and indexing constant across conditions, while the factorial design models residual corpus and query-format effects directly. Chunking effects were negligible in the companion study (η^2^=0.002) [3] but are reassessed here on a representative subset of 4 models to ensure robustness. The interventions evaluated here target embedding geometry; they do not address retrieval failures driven by vocabulary mismatch or chunking strategy at scale.

### Research Questions

The four research questions addressed are:

- RQ1: How does retrieval performance vary with transformer layer depth across the 13 model configurations, and what layer-wise patterns are consistent across architectural classes?
- RQ2: How much variation in retrieval performance is associated with model configuration vs clinical-context factors (corpus, query format), and does this attribution change with layer depth?
- RQ3: In matched architectural comparisons that hold the training objective constant or the training domain constant, which axis, the training objective or the training domain, drives the performance gap between models?
- RQ4: Can deployment-relevant post hoc geometric correction (corpus-only zero-phase component analysis [ZCA] whitening, fit on document embeddings alone) recover retrieval performance to a substantively meaningful extent in this controlled benchmark, and does this correction generalize across documents not used to fit the transform?

RQ2 is phrased in terms of association rather than causation because the study cannot fully isolate training objective from architecture, pooling, model size, tokenizer, and training data; it therefore quantifies variation associated with model configuration as a whole. RQ3 addresses the training-objective vs training-domain contrast using matched architectural comparisons.

The intended contribution is mechanistic and methodological. This study does not propose a deployment standard; practical implications are presented as discussion-level diagnostic considerations that require prospective validation on target institutional corpora before use in clinical retrieval systems.

### Relationship to Companion Study

This manuscript is a mechanistic companion to the accepted benchmark study [3]. It reuses the same clinical retrieval setting, corpora, and metadata-derived query design, but adds layer-wise extraction, geometric trajectory analysis, matched controls, ZCA evaluation, and per-query, layer-wise modeling. The 2 manuscripts are therefore complementary rather than duplicative.

### Related Work

#### Anisotropy and Embedding Geometry in Transformer Representations

The anisotropy of contextual word representations was characterized by Ethayarajh [9], who showed that contextual embeddings from BERT, GPT-2, and ELMo occupy a narrow cone in representation space across all noninput layers. Razzhigaev et al [10] demonstrated distinct anisotropy profiles for encoder vs decoder models. Mechanistic accounts diverge: Machina and Mercer [11] attribute anisotropy to tied input/output embedding matrices, whereas Godey et al [12] link it to sharp attention patterns. Subsequent work has explored isotropy-promoting training objectives (BioLORD-2023 [13] adopts an explicit isotropy term in its contrastive loss) and post hoc whitening transforms [14-16] that compress eigenspectra. These studies focus on general-domain text; to the author’s knowledge, no prior work has conducted layer-level geometric and retrieval analyses of clinical or biomedical text across the architectural classes evaluated here.

#### Clinical Embedding Benchmarks

In the clinical embedding benchmarking literature, Myers et al [17] evaluated 7 embedding models with different pooling strategies for EHR retrieval and reported that BGE-base outperformed domain-specific models, consistent with the companion study’s findings. Soffer et al [18] evaluated 39 embedding models across 7 medical semantic similarity tasks. Hosseini et al [19] showed that combining intermediate BERT layers improved semantic textual similarity by up to 16% in Spearman correlation, though this was evaluated on general-domain similarity tasks rather than clinical retrieval. None of these studies conducted layer-level analysis on clinical retrieval data.

#### Retrieval Evaluation Methodology

The information retrieval evaluation literature provides the methodological foundation for the metrics used here. The Massive Text Embedding Benchmark [20] established standardized retrieval evaluation across diverse domains, and the Benchmarking Information Retrieval (BEIR) suit [21] provided heterogeneous-domain retrieval benchmarks emphasizing zero-shot generalization. Both highlight that ranking metrics computed on small candidate sets can overestimate performance relative to production-scale retrieval, a consideration that motivated the validation experiments at 4-5× scale in this study.

Dense retrieval architectures pioneered by Karpukhin et al [22] have been widely adopted for clinical and biomedical retrieval, with their failure modes subsequently characterized in BEIR-style evaluations [23]. Reranking pipelines such as ColBERT [24] and BERT-based rerankers [25] partially mitigate the limitations of single-stage dense retrieval. Calibration of embedding similarity scores [26] is an active area; the present ZCA correction is a form of post hoc calibration applied at the embedding-vector level rather than at the score level. Clinical information retrieval evaluation has emphasized known-item retrieval [27] and the impact of query-document lexical overlap on apparent performance [23]; both inform the lexical-overlap audit reported below.

#### Gap Addressed by This Study

To the author’s knowledge, no prior study has conducted (1) layer-level geometric and retrieval analyses of clinical documents across 13 transformer configurations, including matched architectural controls that separate training-objective from training-domain effects; (2) layer-wise per-query linear mixed-effects (LME) analysis of retrieval performance; or (3) cross-validated evaluation of corpus-only ZCA whitening as a deployment-relevant geometric correction. This study addresses each of these gaps directly.

## Methods

### Models

#### Overview

A total of 11 transformer models across 13 configurations were evaluated (Table 1). The panel aligns with the companion study [3] by excluding the OpenAI application programming interface model (no hidden-state access) and Best Match 25 (BM25; nonneural), and by adding 2 new configurations to enable matched comparisons across training objective and training domain dimensions. The models span 6 categories: domain encoders (BioBERT [4], ClinicalBERT [5]), general encoders (BERT-base-uncased [28]), biomedical retrievers (BioLORD-2023 [13], MedCPT [29]), general-purpose retrievers (BGE-base [6], GTE-base [7], Nomic-embed-text [8]), general LLMs (E5-Mistral-7B [30], Phi-3-mini [31]), and biomedical LLMs (BioMistral-7B [32]). Two ablation variants are retained from the companion design: E5-Mistral-7B with mean pooling and no instruction prefix and Nomic-embed-text without search_query/search_document prefixes.

ClinicalBERT uses a DistilBERT architecture (6 transformer layers) rather than BERT-base (12 layers); this architectural difference is noted where relevant. BERT-base-scale models have 13 extraction points (input embeddings plus 12 transformer layers). LLM-scale models (E5-Mistral-7B, Phi-3-mini, BioMistral-7B, and the E5-Mistral-7B-ablation variant) have 33 extraction points (including the input and 32 layers). MedCPT, a dual-encoder model, was evaluated by extracting layers from the query and article encoders separately.

**Table 1:** Model configurations evaluated in the 13-configuration panel. BERT-base-uncased and BioMistral-7B were added beyond the companion-study panel to serve as matched-comparison controls.

Model	HuggingFace ID	Category^a^	Architecture^b^	Layers	Pool^c^	Notes
BioBERT	dmis-lab/biobert-v1.1	DE^d^	BERT-base	12	M	fp32^e^
ClinicalBERT	medicalai/ClinicalBERT	DE	DistilBERT	6	M	fp32
BERT-base	bert-base-uncased	GE^f^	BERT-base	12	M	fp32; matched control
BioLORD	FremyCompany/BioLORD	BR^g^	MPNet	12	M	fp32
MedCPT	ncbi/MedCPT-{Query,Article}-Encoder	BR	BERT-base	12	C	Dual encoder
BGE^h^-base	BAAI/bge-base-en-v1.5	GR^i^	BERT-base	12	M	fp32
GTE^j^-base	thenlper/gte-base	GR	BERT-base	12	M	fp32
Nomic	nomic-ai/nomic-embed-text-v1.5	GR	NomicBERT	12	M	With prefixes
Nomic-nopfx	nomic-ai/nomic-embed-text-v1.5	GR	NomicBERT	12	M	Without prefixes (ablation)
E5-Mistral	intfloat/e5-mistral-7b-instruct	GL^k^	Mistral-7B	32	E	fp16^l^; instruction-tuned
E5-Mistral- ablation	intfloat/e5-mistral-7b-instruct	GL	Mistral-7B	32	M	Mean pool, no instruction (ablation)
Phi-3-mini	microsoft/Phi-3-mini-4k-instruct	GL	Phi-3	32	M	fp16
BioMistral	BioMistral/BioMistral	DL^m^	Mistral-7B	32	M	fp16; matched control

^a^Model category (general encoder, biomedical encoder, general retriever, biomedical retriever, decoder LLM).

^b^Base architecture.

^c^Pooling strategy (CLS=classification [CLS] token; Mean=mean of token embeddings; Last=last token; EOS=end-of-sequence token embedding).

^d^DE: domain encoder.

^e^fp32: 32-bit floating-point precision.

^f^GE: general encoder.

^g^BR: biomedical retriever.

^h^BGE: Beijing Academy of Artificial Intelligence General Embedding.

^i^GR: general retriever.

^j^GTE: General Text Embeddings.

^k^GL: general large language model.

^l^fp16: 16-bit floating-point precision.

^m^DL: domain large language model.

#### Matched-Comparison Rationale

Two configurations were added beyond the companion panel to help separate the training objective from the training domain. BERT-base-uncased serves as a non–retrieval-trained BERT-base control for BioBERT and the BERT-family retrievers. BioMistral-7B serves as a biomedical Mistral-7B control for E5-Mistral-7B. These comparisons are architecture-matched or partially matched, but residual confounding from the tokenizer, instruction data, pooling, and model scale remains.

### Corpora and Queries

#### Overview

Three clinical corpora were reused from the companion study [3] at a 100-document working scale per corpus to enable mechanistic layer-by-layer analysis: MTSamples (n=100), PMC-Patients case reports from PubMed Central (n=100) [20], and synthetic clinical notes generated by Mistral-7B-Instruct-v0.2 (n=100). For each corpus, 100 keywords and 100 natural-language queries were generated from document metadata using GPT-4o, following the reduced-lexical-dependence design used in the companion study’s validation experiment [3]. Each query corresponds to a single known-relevant document (known-item retrieval design). The complete GPT-4o prompt is provided in Multimedia Appendix 1.

The known-item retrieval design with 100 documents per corpus provides a controlled environment for mechanistic, layer-by-layer analysis but is substantially smaller than production indices; absolute results should not be extrapolated directly to production-scale retrieval. Validation at 4-5× scale (500 PMC-Patients and 400 MTSamples) is reported in the “Validation on Independent Corpora” subsection of the Results section.

#### Alignment Audit for the Synthetic Corpus

The layer-extraction pipeline preserved the document order used to generate the metadata queries. A locally cached synthetic CSV used to reload the corpus for a sensitivity analysis had been resorted by synth_id, breaking the index alignment; query-document pairing for that sensitivity analysis was therefore recovered via BM25 top-1 matching with greedy one-to-one deduplication, replicating the alignment-recovery procedure documented in the companion study [3]. The full audit table is reproduced in Multimedia Appendix 2. The primary per-query layer-wise LME uses ranks derived from the original-pipeline alignment, not the recovered alignment.

#### BM25 Alignment-Check Baselines

Alignment between queries and documents was verified using BM25 on the 100-document subset before evaluating each embedding model, yielding MRR@10 of 0.87 (MTSamples), 0.92 (PMC-Patients), and 0.96 (synthetic). These baselines confirm that the queries are appropriately aligned with their target documents at the lexical level and serve as a sanity check on the experimental design. They are not evidence that retrieval can be reduced to lexical matching: the lexical-overlap audit measures the BM25-vs-embedding rank correlation directly across the 13 models and confirms a substantial nonlexical contribution to embedding-based retrieval (Spearman ρ range –0.02 to 0.37). This finding directly addresses the apparent contradiction between high BM25 baselines and the manuscript’s claim of reduced lexical dependence.

### Layer-Level Measurements

#### Overview

For each model and layer, embeddings were extracted across the 6 corpus × query format conditions using output_hidden_states=True (or equivalent forward hooks for architectures that do not support this parameter). Pooling matched vendor specifications: mean pooling for most models, classification token pooling for MedCPT, and last-token (end-of-sequence [EOS]) pooling for E5-Mistral-7B. All embeddings were L2-normalized.

#### Geometric Measures (Computed on Document Embeddings)

**Figure 1:**
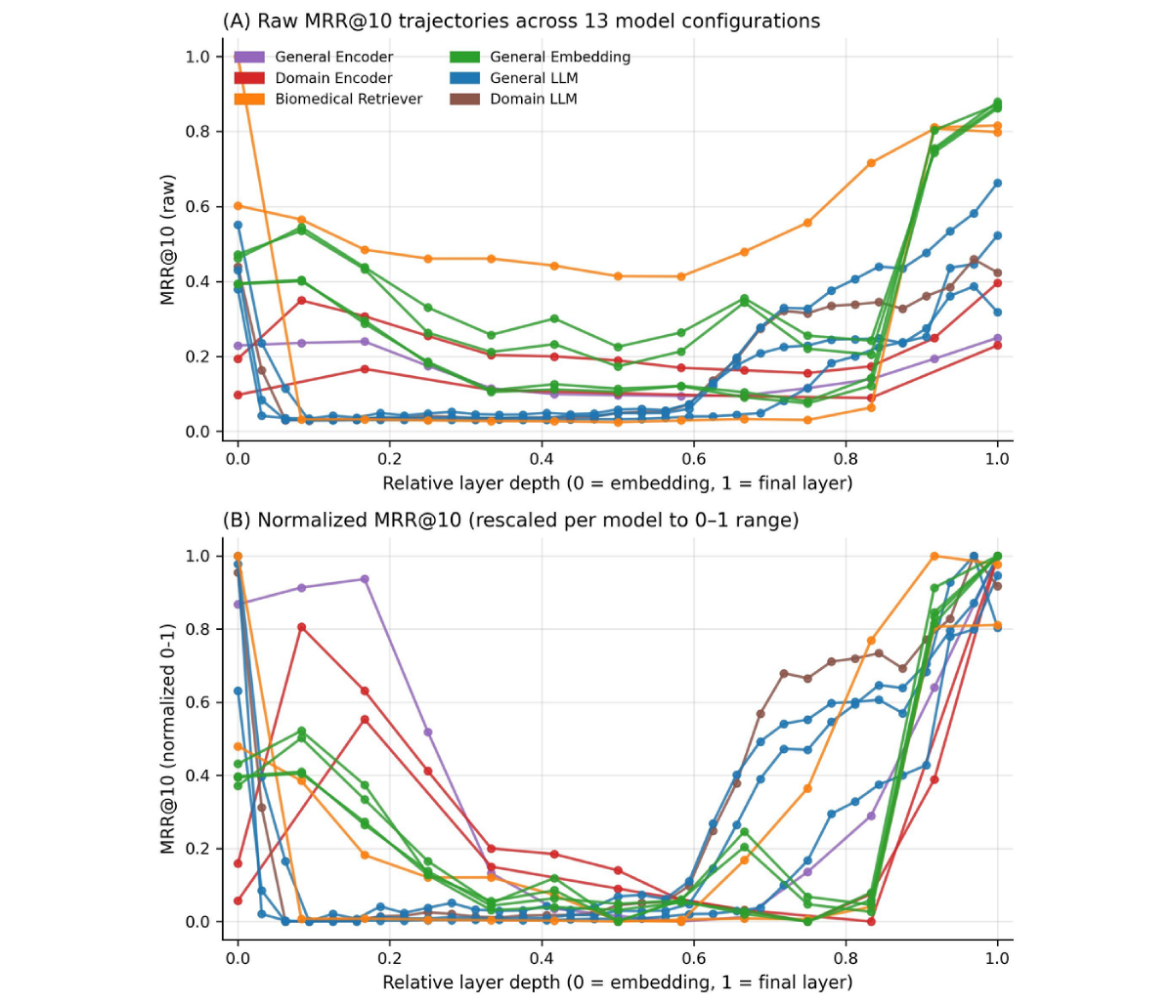
Layer-wise mean reciprocal rank at cutoff 10 (MRR@10) trajectories across all 13 model configurations. (A) Raw MRR@10 by relative layer depth (0=input embedding layer, 1=final layer); each line is one model configuration averaged across the 6 corpus × query-format conditions, color-coded by category. (B) The same data, minimum-maximum normalized per model to a 0-1 range to aid cross-model trajectory comparison (absolute performance differences are suppressed by the normalization). Most configurations show U-shaped or monotonically improving curves. In the per-query mixed-effects model, 3 non–retrieval-trained encoders worsen with depth (BERT-base-uncased, BioBERT, ClinicalBERT; positive rel_layer coefficients), whereas the remaining 10 configurations improve with depth, with the greatest improvement in the decoder large language models.

The following geometric measures were used:

- Participation ratio (PR): PR = (sum_i sigma_i^2^)^2^/sum_i sigma_i^4^, where sigma_i are the singular values of the centered embedding matrix. PR reflects the effective number of dimensions used by the embedding distribution; lower values indicate more collapsed representations.
- Average pairwise cosine similarity (average pairwise cosine): the mean cosine similarity across 10,000 randomly sampled embedding pairs. Higher values indicate a narrower embedding cone (more anisotropic).
- Anisotropy singular value decomposition–based: the top squared singular value as a fraction of the sum of all squared singular values, indicating concentration of variance along a single direction.
- Retrieval measures: MRR@10 was the primary retrieval metric. Recall at cutoff 10 (Recall@10; the proportion of queries for which the relevant document appeared in the top 10 results) was reported as a secondary metric. Both were computed by ranking documents using cosine similarity with a one-to-one query-document mapping.
- Normalized mean reciprocal rank (MRR): computed for cross-model trajectory visualization by rescaling each model’s layer-wise MRR@10 values to the 0-1 range. Raw MRR@10 values are reported alongside the normalized values in Figure 1.

#### Summary Trajectory Metrics

For the descriptive characterization of layer-wise trajectories, the following derived metrics are defined in the Methods section:

- Recovery ratio: (MRR_final – MRR_trough)/(MRR_peak – MRR_trough), where MRR_peak is the highest MRR@10 among layers 0%-25% of network depth, MRR_trough is the lowest MRR@10 among layers 35%-55% of network depth, and MRR_final is the final-layer MRR@10. Values above 1.0 indicate that the final layer exceeds the early-layer peak; values below 1.0 indicate incomplete recovery. The recovery ratio is a study-defined descriptive index, not a standard metric. Per-model values are descriptive observations rather than formal statistical tests. The 1.85 threshold used for a “strong recovery” classification is data-derived and reported as a hypothesis-generating screening signal only, not a deployment criterion.
- PR expansion: PR_final/PR_trough, where the trough is the layer with the lowest within-model PR. Values above 1.0 indicate dimensionality expansion at the final layer.

#### Document Length Analysis (Exploratory)

As an exploratory analysis, documents within each corpus were partitioned into terciles based on approximate token count (whitespace-tokenized). The following token boundaries were used (33.3rd and 66.7th word-count percentiles of each corpus’s 100-document working set): MTSamples (short ≤286 words, medium 287-453, long >453), PMC-Patients (short ≤331, medium 332-518, long >518), and synthetic (short ≤290, medium 291-308, long >308). The compressed synthetic range reflects that corpus’s structural uniformity. Per-tercile sample sizes are approximately 33 documents, and analyses at this sample size are statistically underpowered. Per-tercile retrieval metrics are reported in Multimedia Appendix 3; the main text reports only directional summaries.

### Statistical Analysis

The primary inferential analysis used a per-query, layer-wise LME model, fit separately for each of the 13 configurations. The outcome was log(rank+1), where rank is the position of the known relevant document among 100 candidates based on cosine similarity. The core model was log(rank+1) ~ C(intervention) + C(corpus) + C(query_format) + rel_layer, with a random intercept and random slope on rel_layer by corpus_query_idx (100 queries × 3 corpora = 300 groups per model). rel_layer was normalized to the range 0 to 1. Detailed coefficients, SEs, and convergence diagnostics are reported in Multimedia Appendix 4.

The primary specification was a random-slope model with a random intercept and a random slope for rel_layer by query identity. Each query was treated as a distinct group within its source corpus (300 query groups per model: 100 queries × 3 corpora). All 13 configurations converged. The estimated intraclass correlation coefficient ranged from 0.06 to 0.55 (median 0.41) across models, indicating substantial between-query variance in retrieval difficulty and supporting the per-query random-effects specification. Bootstrap CIs from 3 representative models aligned with Wald intervals in direction and approximate width. Fixed-effect *P* values are reported descriptively without multiple-comparison correction.

No formal multiple-comparison correction was applied across layers. Layer-level *P* values from the per-query LMEs are interpreted descriptively within the LME inferential framework rather than as confirmatory hypothesis tests. The principal inferences in this manuscript rest on the random-slope fixed-effect coefficients and the matched-comparison contrasts, not on layer-by-layer significance counts.

Cluster-bootstrap 95% CIs for the rel_layer fixed-effect coefficient were computed for 3 representative configurations spanning the model panel—BERT-base-uncased (β=+0.624, Wald 95% CI +0.537 to +0.712, bootstrap 95% CI +0.553 to +0.694), BGE-base (β=–0.165, Wald 95% CI –0.245 to –0.086, bootstrap 95% CI –0.229 to –0.104), and E5-Mistral-7B-ablation (β=–1.317, Wald 95% CI –1.392 to –1.243, bootstrap 95% CI –1.391 to –1.276)—using 50 cluster-bootstrap iterations clustered on corpus_query_idx. Bootstrap and Wald intervals agreed in sign, coefficient ordering, and approximate width across all 3 configurations; parametric SEs therefore provide a reasonable representation of uncertainty in the primary rel_layer coefficient.

For the matched architectural comparisons reported in the Results section (BERT-base architecture: BGE-base vs BERT-base-uncased; Mistral-7B architecture: E5-Mistral-7B-ablation vs BioMistral-7B; biomedical encoders: BioLORD-2023 vs BioBERT), 3 pairwise pooled LME models were fit with the specification log(rank + 1) ~ rel_layer × C(model) + C(corpus) + C(query_format), with a random intercept and random slope on rel_layer by corpus_query_idx. The rel_layer × C(model) interaction term tested whether the 2 paired configurations differed in layer-depth trajectory; the reported *P* values are from Wald tests on the interaction coefficient. The additive per-configuration models described above and the pairwise pooled interaction models here address different inferential questions: the per-configuration slopes describe each model’s own layer-depth pattern, whereas the pooled interaction tests whether paired configurations differ in that pattern within a shared architecture.

The cross-validation design used to evaluate the corpus-only ZCA intervention (post hoc interventions, below) fits the ZCA covariance from 80 training-fold documents per fold in a hidden-state space of 768-4096 dimensions, depending on the model. The resulting sample covariance is rank-deficient by 689-4017 dimensions; the whitening transform relies on the ε ridge regularizer to be well-defined outside the empirical ~80-dimensional subspace captured by the nonzero eigenvalues, where it reduces to ε-scaled near-identity behavior. The ZCA intervention in this cross-validation setup therefore operates as a hybrid of data-driven whitening within the empirical 80-dimensional subspace and ε-regularized near-identity transformation in the residual subspace. Empirically, the ε sensitivity sweep reported in Multimedia Appendix 5 confirms that the intervention’s effect on retrieval depends materially on ε within the explored range (10^–7^ to 10^–2^): per-model ΔMRR@10 ranges of up to 0.75 in absolute magnitude were observed, with the sign of ΔMRR@10 changing across the sweep for several models. This is consistent with the hybrid behavior described above, where ε determines the whitening transform’s behavior in the unspanned subspace. This is a mathematical property of small-sample covariance estimation in high-dimensional spaces, not specific to the present implementation; its consequences for the interpretation of the cross-validated intervention results are discussed in the Limitations section.

### Post Hoc Interventions

A total of 5 no-retraining interventions were evaluated across all 13 configurations: corpus-only ZCA whitening (the primary deployment-relevant analysis), transductive ZCA whitening (a query-contaminated upper-bound reference), mean centering, post hoc layer selection, and post hoc layer combination. Layer selection and layer combination are reported only as descriptive ceilings because they select layers based on test-set performance.

For corpus-only ZCA, W = U(Λ + εI)^(–1/2) U^T and x’ = W(x – μ_D)/||W(x – μ_D)||, where UΛU^T is the eigendecomposition of the centered document covariance, μ_D is the document mean, and ε is a ridge regularizer. W and μ_D are fit on document embeddings only; queries are transformed afterward using the same W. The default ε was 10^–5^, with a sensitivity sweep over 10^–7^ to 10^–2^ (Multimedia Appendix 5). The sweep showed that ε=10^–5^ is not panel-wide optimal: tier 2 models reached their largest gains at ε in the 10^–5^ to 10^–3^ range (most at 10^–4^), whereas tier 1 models were degraded by corpus-only ZCA at every ε tested. The headline 2-tier pattern is preserved across the sweep; ε=10^–5^ was retained as a single conservative default rather than a per-model tuned optimum.

Five-fold cross-validation was performed for corpus-only ZCA by fitting W and μ_D on 80 documents and evaluating on 20 held-out documents within each corpus and query-format condition. This tests whether the transform generalizes beyond the documents used to fit it.

### Lexical-Overlap Audit

To quantify the contribution of lexical matching to embedding-based retrieval across the 13 models, Spearman rank correlations between BM25 and embedding ranks were computed for each (model, corpus, and query format) condition. For each query, the BM25 rank assigns a position based on lexical overlap between the tokenized query and document; the embedding rank assigns a position based on cosine similarity between query and document embeddings. The Spearman correlation between these two ranks measures how well embedding-based retrieval recapitulates lexical matching: a correlation of 1.0 implies the embedding model is effectively BM25 with extra steps, while a correlation near 0 implies the embedding model uses purely nonlexical signal. Results are reported in the “Lexical Overlap Between BM25 and Embedding-Based Retrieval” subsection of the Results section and address the apparent contradiction between high BM25 baselines and the reduced-lexical-dependence claim.

### Chunking Sensitivity

To assess sensitivity to document chunking, a sensitivity analysis was conducted on 4 representative models (BERT-base-uncased, BGE-base, MedCPT, and BioMistral-7B), each evaluated under 3 token-level truncation strategies. Documents were tokenized with the model-specific tokenizer; for the first strategy, the first N input tokens were retained, for the last strategy, the last N tokens were retained, and for the full strategy, the entire document was passed to the model with the tokenizer’s native truncation at max_length. N was set to the model’s max_length: 512 for BERT-base-uncased, BGE-base, and MedCPT; 2048 for BioMistral-7B. Because tokenizer-native truncation retained the leading max_length tokens, the full strategy produced the same leading-token input as the explicit first strategy. The last strategy therefore tested sensitivity to using document-tail rather than document-head content. Query texts remained unchanged across strategies. For each strategy, document embeddings were re-extracted, and retrieval performance was recomputed. The 4-model subset was chosen to span architecture classes: general encoder, general retriever, biomedical retriever, and biomedical LLM. Results are reported in the “Chunking Sensitivity” subsection of the Results section.

### Validation Corpora

To evaluate generalization, the layer-level analysis was repeated on 2 independent validation corpora representing different clinical document types: 500 PMC-Patients case reports (random seed 123, no overlap with the primary 100-document sample) and 400 MTSamples medical transcriptions (the remaining documents after excluding the primary 100, drawn from the same locally cached CSV used in the companion study). Queries were generated using the same metadata-based GPT-4o approach as in the primary experiment. Validation was conducted in 2 stages. First, the 8 BERT-scale configurations from the original panel were evaluated on PMC-500 and MTSamples-400; results are reported in the “Validation on Independent Corpora” subsection of the Results section. Second, the 5 configurations not included in the first stage, BERT-base-uncased, BioMistral-7B, Phi-3-mini, E5-Mistral-7B, and E5-Mistral-7B-ablation, were evaluated on the same expanded natural-text corpora; results are reported in the “Independent Validation of the Remaining Configurations” subsection. Together, the 2 stages provide independent-corpus validation for all 13 panel configurations.

### Ethical Considerations

This study used only publicly available, deidentified datasets and synthetic clinical notes generated by a language model. MTSamples comprises publicly posted, deidentified medical transcriptions accessed at MTSamples [33] in accordance with the site’s terms of service. PMC-Patients comprises published case reports from PubMed Central [34]. Synthetic clinical notes were generated by Mistral-7B-Instruct-v0.2 and contain no real patient data.

No human subjects were involved in this study, and no institutional review board approval was required. No identifiable individual patient information appears in any of the corpora, the manuscript, or its supplementary materials. No compensation was provided because no participants were enrolled. As a secondary analysis of deidentified public datasets (MTSamples or PMC-Patients) and entirely model-generated synthetic notes, the study qualifies for ethics review exemption under common research ethics frameworks for non–human subject research. The original informed consent and license terms governing the source datasets cover secondary research use; no additional participant consent was applicable.

### Compute Environment

The layer-extraction pipeline was run on Google Colab Pro+ with an NVIDIA H100 80GB GPU. Additional analyses, including matched comparisons, corpus-only ZCA cross-validation, per-query LME, lexical overlap, chunking sensitivity, and intervention sweeps, were run on Google Colab Pro+ with an NVIDIA RTX PRO 6000 Blackwell 96GB GPU. Pooling and precision choices align with the companion study configuration to ensure cross-paper reproducibility. Code is available as described in the Data Availability section.

## Results

### Layer-Wise Retrieval Trajectories Across 13 Configurations

All 13 model configurations exhibited identifiable layer-wise structure, but the dominant pattern across the panel is more heterogeneous than that of a single U-shaped collapse model. Figure 1 shows raw MRR@10 trajectories (panel A) and per-model minimum-maximum normalized trajectories (panel B) as a function of relative layer depth (0=embedding layer, 1=final layer), averaged across the 6 corpus × query-format conditions.

Two principal trajectory patterns emerge:

1. Classical midlayer collapse with final-layer recovery. Observed in retrieval-trained encoder models (BGE-base, GTE-base, BioLORD-2023, MedCPT, Nomic-embed-text, and Nomic-nopfx). These models drop from early-layer MRR@10 of 0.40-0.60 to a midlayer trough of 0.04-0.25 (35%-55% relative depth), then recover sharply in the final 1-2 layers to MRR@10 of 0.80-0.88. The recovery is driven by a sharp geometric restructuring, as documented in the “Final-Layer Geometric Restructuring Is Associated With Retrieval Recovery” section.
2. Monotonic improvement with layer depth is observed in most decoder-LLM configurations (E5-Mistral-7B, Phi-3-mini, BioMistral-7B, and E5-Mistral-7B-ablation). These models show retrieval performance that increases with depth, without a deep midlayer trough; final-layer values are the highest within their trajectory.

A third, less common pattern, partial recovery with high final-layer anisotropy, appears in BioBERT, ClinicalBERT, and BERT-base-uncased. These 3 non–retrieval-trained encoder models show a midlayer dip but recover only modestly at the final layer (BioBERT 0.40, ClinicalBERT 0.23, and BERT-base-uncased 0.25), well below the retrieval-trained encoder family.

The per-model raw and normalized values are documented in Multimedia Appendix 6. The U-shaped trajectory is concentrated among retrieval-trained encoder models and partially evident in non–retrieval-trained encoders, but it is not characteristic of decoder LLMs. Raw MRR values and CIs are reported alongside the normalized values in Multimedia Appendix 6.

### Final-Layer Trajectory Metrics

Table 2 summarizes per-model peak, trough, and final-layer MRR@10 values, along with the recovery ratio and PR expansion factors. Complete model-level baseline and intervention results are provided in Multimedia Appendix 7. MedCPT’s layer-0 anomaly (MRR@10=1.000, an artifact of dual-encoder shared vocabulary embeddings) is excluded from the early-peak window. The recovery ratio is structurally degenerate for MedCPT because the early-layer MRR (L1-L6 ≈ 0.025-0.033) is essentially identical to the midlayer trough, producing a near-zero denominator. We report the value with this caveat and note that MedCPT’s trajectory does not follow the U-shape geometry the metric was designed to summarize.

A descriptive threshold combining recovery ratio >1.85 and PR expansion >1.9× is associated with final-layer MRR@10 >0.80 in the retrieval-trained encoder subset (BGE-base, GTE-base, BioLORD-2023, Nomic family; all final MRR >0.80, all PR expansion ≥1.90×). The matched controls and LLM-scale models confirm that this threshold is data-derived and exploratory rather than a deployment criterion.

Cosine drop is negative for BioBERT (–0.030) and Phi-3-mini (–0.236), indicating that the final layer is more anisotropic than the early peak. Both fall into the extreme-anisotropy tier. The “geometric restructuring at the final layer” pattern observed in retrieval-trained encoders (positive cosine drop combined with substantial PR expansion) is therefore not a property of all transformer architectures but is specific to retrieval-objective-trained encoders.

**Table 2:** Layer-level trajectory characteristics per model.^a^

Model	Peak^b^ (layer, MRR^c^)	Trough^d^ (layer, MRR)	Final MRR	Recovery	PR expansion^e^	Cosine drop^f^
BGE^g^-base	L1, 0.400	L4, 0.106	0.872	2.60	2.57×	+0.236
GTE^h^-base	L1, 0.408	L4, 0.110	0.881	2.59	2.63×	+0.172
BioLORD	L0, 0.602	L7, 0.414	0.798	2.04	1.90×	+0.439
Nomic	L1, 0.545	L6, 0.225	0.863	2.00	2.66×	+0.313
Nomic-nopfx	L1, 0.535	L6, 0.174	0.863	1.91	2.28×	+0.357
MedCPT^i^	L2, 0.033	L6, 0.025	0.816	Degenerate^i^	4.01×	+0.242
*BERT-base*	L2, 0.240	L7, 0.094	0.250	1.07	1.15×	+0.009
BioBERT	L1, 0.350	L7, 0.170	0.398	1.27	1.35×	–0.030
ClinicalBERT	L1, 0.167	L3, 0.102	0.230	1.97	1.38×	+0.025
*BioMistral*	L0, 0.440	L11, 0.035	0.424	0.96	1.45×	+0.144
Phi-3-mini	L0, 0.551	L12, 0.031	0.522	0.94	2.68×	–0.236
E5-Mistral	L0, 0.379	L11, 0.044	0.318	0.82	0.97×	+0.036
E5-Mistral-ablation	L0, 0.430	L11, 0.036	0.663	1.59	2.24×	+0.149

^a^All values are averaged across 6 corpus × query-format conditions. Final MRR values match the intervention-pipeline final-layer baseline; layer-wise trajectory extraction (streams A and G) produces final-layer MRR values differing by <1% from the intervention-pipeline baseline. Peak, Trough, PR expansion, and Cosine drop are computed from the layer-wise trajectory extraction. Values for new matched controls (BERT-base-uncased, BioMistral-7B) are highlighted.

^b^Early peak=highest MRR@10 in the first quarter of layers (excluding layer 0 for MedCPT).

^c^MRR: mean reciprocal rank.

^d^Trough=lowest MRR@10 in the middle layers (35%-55% relative depth).

^e^PR expansion=participation ratio at the final layer divided by participation ratio at the trough.

^f^Cosine drop=average cosine at the peak layer minus average cosine at the final layer (negative values indicate increased anisotropy from peak to final).

^g^BGE: Beijing Academy of Artificial Intelligence General Embedding.

^h^GTE: General Text Embeddings.

^i^MedCPT: layer-0 MRR@10=1.000 (excluded; reflects shared vocabulary embeddings in the dual-encoder architecture). Layers 1-6 produce near-zero MRR (range: 0.025-0.033) before a sharp recovery in the later layers; the recovery ratio, as defined in Methods, has a near-zero denominator (peak – trough ≈ 0.008) and is not meaningfully computable for this model. The PR expansion factor (4.01×) remains interpretable and is the largest in the panel.

### Matched Architectural Comparisons Support the Training-Objective Interpretation Over Training Domain

The matched comparisons supported the training objective over the training domain as the stronger explanatory axis. BERT-base-uncased and BGE-base share the BERT-base architecture but differ in retrieval fine-tuning; final-layer MRR@10 was 0.250 vs 0.872. BioMistral-7B and E5-Mistral-7B share the Mistral-7B base; the E5-Mistral ablation with mean pooling reached 0.663 vs 0.424 for BioMistral-7B. BioBERT vs BioLORD-2023 was directionally consistent (0.398 vs 0.798) but confounded by the BERT vs MPNet architecture.

Formal pooled LMEs confirmed significant rel_layer × model interactions across all 3 reported contrasts (all *P*<.001). These findings are consistent with a role for retrieval-objective training but do not establish causality because tokenizer, pretraining corpus, instruction data, pooling, and scale remain potential confounders.

### Final-Layer Geometric Restructuring Is Associated With Retrieval Recovery

Recovery from midlayer collapse in the retrieval-trained encoder family is driven by sharp geometric restructuring in the final 1-2 layers. For BGE-base, the PR expanded substantially across the final layers (eg, from layer 10 to layer 12), while average cosine similarity declined and MRR@10 increased sharply. GTE-base, MedCPT, and Nomic-embed-text exhibited similar transition patterns. Layer-to-layer PR, cosine, and MRR values per model are reported in Multimedia Appendix 6.

In contrast, BioBERT’s PR increased modestly across layers, and its average cosine remained above 0.95 throughout. Its final-layer MRR@10 matched the value reported in Table 2. BERT-base-uncased, the matched control, exhibited similar limitations, with modest PR expansion. ClinicalBERT, with 6 transformer layers (DistilBERT architecture), lacked the depth required for a comparable recovery phase.

**Figure 2:**
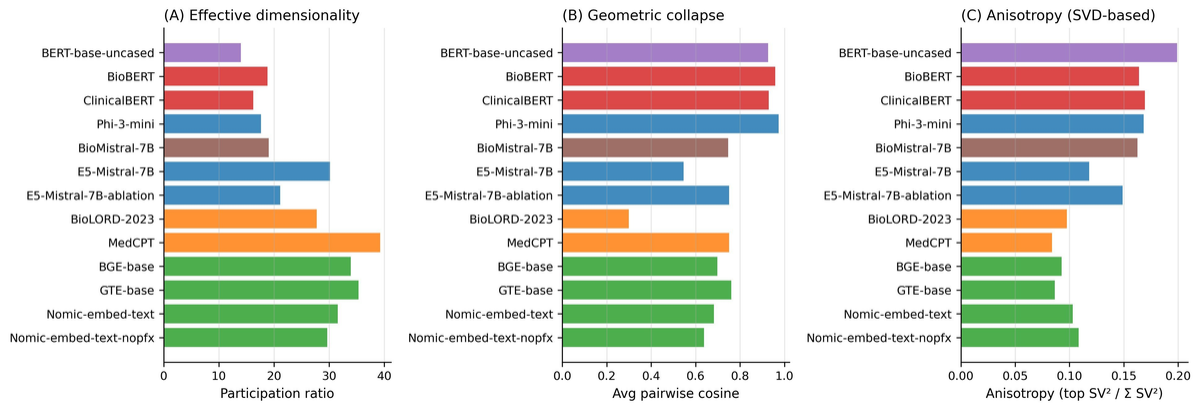
Final-layer geometric diagnostics across all 13 model configurations. (A) Participation ratio (effective embedding dimensionality), (B) average pairwise cosine similarity (a measure of representational anisotropy), and (C) anisotropy index (largest squared singular value divided by the sum of squared singular values). All quantities are computed on document embeddings and averaged across the 6 corpus × query-format conditions; points are color-coded by model category. Extreme anisotropy (average cosine >0.92) appears in the non–retrieval-trained encoders and the decoder large language model Phi 3-mini (BERT-base-uncased, BioBERT, ClinicalBERT, and Phi-3-mini), whereas BioLORD-2023—whose contrastive training objective explicitly promotes isotropy—reaches the lowest average cosine in the panel (0.30).

**Figure 3:**
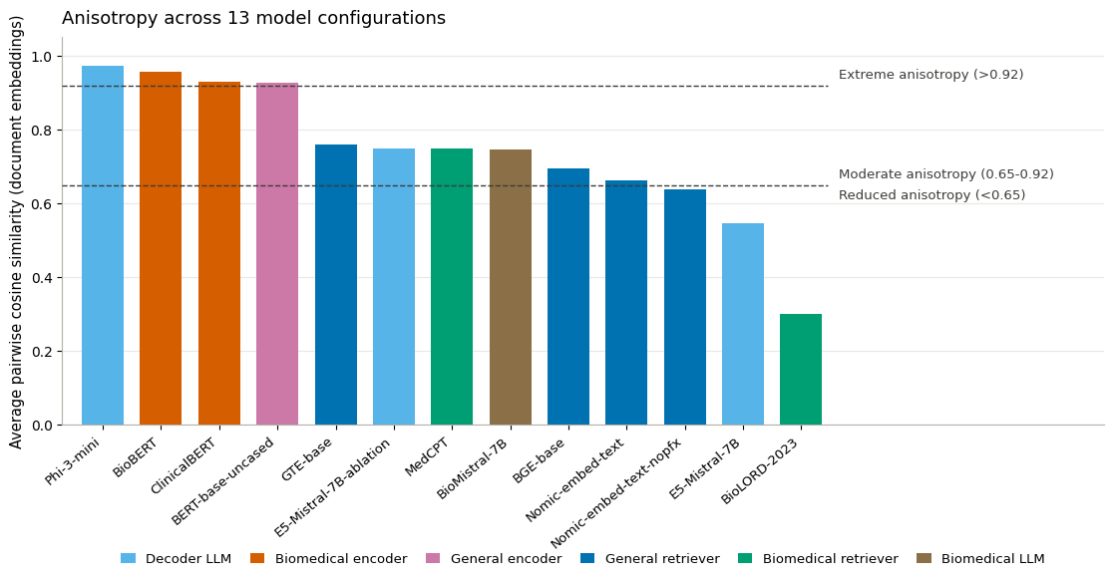
Anisotropy tiers in final-layer document embeddings across all 13 model configurations. Final-layer document-embedding average pairwise cosine similarity, sorted in descending order, revealing 3 empirical tiers. Extreme anisotropy (>0.92): Phi-3-mini (0.974), BioBERT (0.958), ClinicalBERT (0.930), and BERT-base-uncased (0.926). Moderate anisotropy (0.65-0.92): most general retrievers and the remaining large language models (LLMs). Reduced anisotropy (<0.65): Nomic-embed-text-nopfx (0.638), instruction-tuned E5 Mistral-7B (0.546), and BioLORD-2023 (0.300). BioLORD-2023’s isotropy-promoting contrastive objective yields the lowest average cosine in the panel.

Across the full 13-model panel, the final-layer geometric properties are summarized by category in Figure 2, and the average document-embedding cosine is shown in the 3-tier visualization in Figure 3. The matched-control evidence and geometric data converge: the retrieval-training mechanism that produces the final-layer dimensionality expansion is not produced by domain-specific pretraining alone.

### Per-Query Layer-Wise LME Analysis

The per-query random-slope LME converged for all 13 configurations, each of which was fit with 300 query groups (100 queries × 3 corpora). The intraclass correlation coefficient ranged from 0.06 to 0.55 (median 0.41), indicating that between-query difficulty accounted for a substantial share of variance and supporting the use of the per-query random-effects model. Two layer-depth patterns emerged. Three non–retrieval-trained encoders worsened with depth (positive rel_layer coefficients: BERT-base-uncased +0.62, BioBERT +0.22, ClinicalBERT +0.20; all *P*<.001), whereas the remaining 10 configurations improved with depth (negative rel_layer coefficients, all *P*<.001), with the strongest improvement in the decoder LLMs (E5-Mistral-7B=–1.77; E5-Mistral-7B-ablation=–1.32).

Additive LME coefficients for corpus-only ZCA—a layer-averaged summary—are heterogeneous across tier 1 models (negative for BGE-base, GTE-base, and Nomic-embed-text; positive for BioLORD-2023 and MedCPT) and cannot be interpreted as evidence for a uniform final-layer effect. The primary evidence for the 2-tier ZCA response is the cross-validated corpus-only ZCA analysis in Table 3, where all 6 tier 1 configurations show final-layer ΔMRR@10 losses (0.021 to 0.051) and all 7 tier 2 configurations show gains (0.066-0.304). Layer-dependent intervention effects were not interpreted from the additive LME specification. A quadratic rel_layer sensitivity check was statistically preferred across all 11 cleanly fit models, confirming that many trajectories are nonmonotonic; the linear rel_layer term is therefore interpreted as a net-direction summary. Full coefficients and diagnostics are in Multimedia Appendix 4.

**Table 3:** Five-fold cross-validation (CV): corpus-only zero-phase component analysis mean reciprocal rank (MRR) at cutoff 10 vs baseline, per model, with SE across 30 observations per model (5 folds × 6 corpus × query-format conditions). Sorted by tier and Δ.

Model	Baseline MRR	ΔMRR@10, CV fold mean	ΔMRR	SE	n	Tier
BioLORD	0.798	0.777	–0.021	0.017	30	1
MedCPT	0.816	0.770	–0.045	0.022	30	1
BGE^a^-base	0.872	0.826	–0.046	0.017	30	1
Nomic-nopfx	0.867	0.818	–0.049	0.017	30	1
GTE^b^-base	0.879	0.830	–0.050	0.017	30	1
Nomic	0.862	0.811	–0.051	0.015	30	1
E5-Mistral	0.318	0.622	+0.304	0.023	30	2
ClinicalBERT	0.229	0.436	+0.207	0.029	30	2
BERT-base	0.250	0.449	+0.200	0.029	30	2
BioBERT	0.396	0.592	+0.196	0.033	30	2
BioMistral	0.424	0.585	+0.161	0.030	30	2
Phi-3-mini	0.523	0.683	+0.160	0.022	30	2
E5-Mistral-ablation	0.663	0.729	+0.066	0.032	30	2

^a^BGE: Beijing Academy of Artificial Intelligence General Embedding.

^b^GTE: General Text Embeddings.

### Pooling and Instruction Effects in LLM-Scale Models

The E5-Mistral-7B ablation provides complementary evidence on the pooling strategy. Mean pooling without an instruction prefix achieved a final-layer MRR@10 of 0.663, compared with 0.318 for vendor-specified EOS pooling with an instruction prefix; the +0.345 net improvement is consistent with the combined effects of mean pooling and removal of the instruction prefix and cannot be decomposed into pooling-only and instruction-only contributions from the available data, since both factors are confounded in the ablation. This finding motivates a deeper intervention sweep on E5-Mistral-7B-ablation.

### Cross-Validation of Corpus-Only ZCA Generalizes to Held-Out Documents

**Figure 4:**
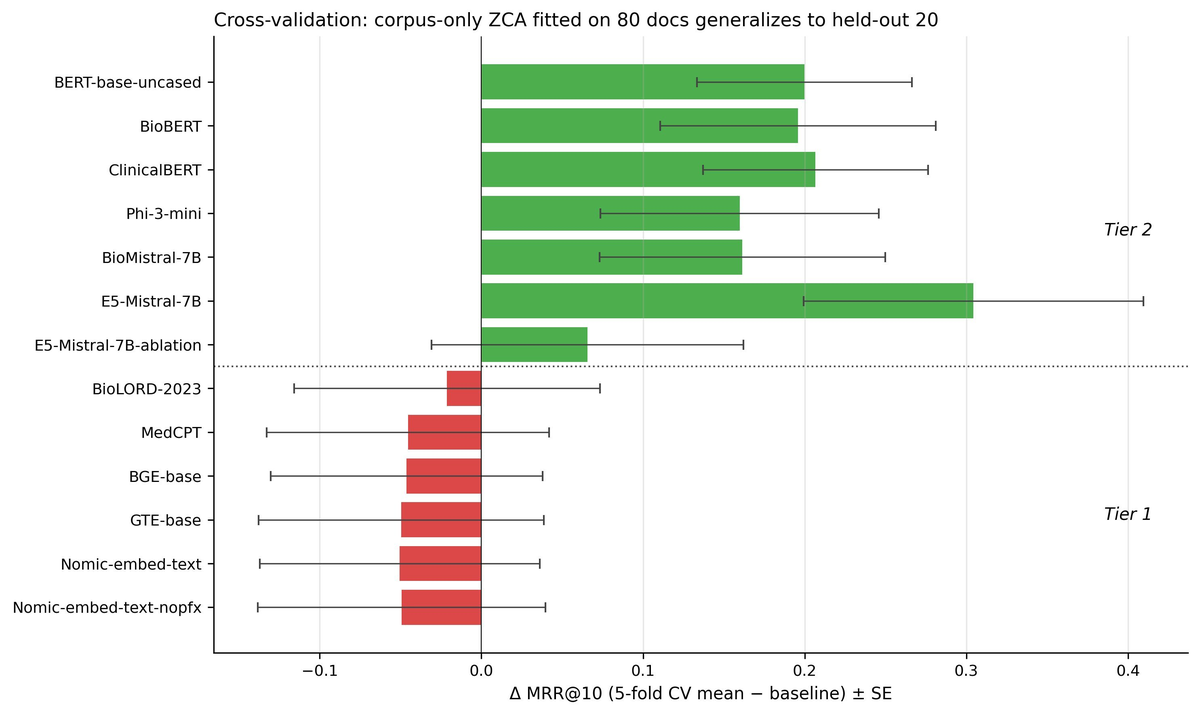
Cross-validated corpus-only zero-phase component analysis (ZCA) whitening across all 13 model configurations. Mean change in mean reciprocal rank at cutoff 10 (ΔMRR@10; corpus-only ZCA minus no-intervention baseline) per model under 5-fold cross-validation (CV). Within each fold, the ZCA transform was fit on 80 documents and evaluated on the held-out 20 documents, ranked against the full 100-document candidate set; bars show the mean across the 30 observations per model (5 folds × 6 corpus × query-format conditions), with error bars giving the standard error of that mean. The 2-tier pattern is preserved under cross-validation: tier 2 (non–retrieval-trained) models show positive deltas (+0.066 to +0.304), while tier 1 (retrieval-trained) models show small negative deltas (–0.021 to –0.051). Exact per-model values are reported in Table 3. The result confirms that the whitening transform generalizes from the fitting documents to held-out documents, helping uncalibrated encoders while modestly degrading already-calibrated retrieval models.

Five-fold cross-validation was performed across all 13 models to assess the risk of test-set contamination. The ZCA transform was fit on 80 documents per fold and evaluated on the held-out 20 documents, with results ranked against the full 100-document candidate set. The mean ΔMRR@10 across folds, relative to the no-intervention baseline, is summarized in Figure 4 and Table 3.

The 2-tier pattern is clear under cross-validated evaluation. Tier 2 non–retrieval-trained models show positive ΔMRR@10 of +0.066 to +0.304 (mean +0.185 across the 7 tier 2 models). Tier 1 retrieval-trained models show negative ΔMRR@10 of –0.021 to –0.051 (mean –0.044 across the 6 tier 1 models). The cross-validation confirms that the ZCA transform fitted on 80 documents (1) generalizes to held-out documents within the same corpus, and (2) produces the expected bimodal effect: it helps the uncalibrated, modestly hurts the already-calibrated. The smallest tier 2 gain (E5-Mistral-7B-ablation=+0.066) cannot be cleanly attributed to model scale. The ablation removes the instruction prefix and switches from EOS to mean pooling simultaneously, so neither factor’s effect can be measured in isolation. Mean pooling is mathematically expected to reduce anisotropy compared with EOS pooling and may contribute to the small gain; however, the empirical anisotropy values for the 2 configurations (mean-pooled ablation 0.751; EOS-pooled instruction-tuned 0.546) represent the net difference associated with both changes simultaneously and cannot be decomposed without matched contrasts that separately vary pooling strategy and instruction formatting on the same underlying checkpoint.

### Anisotropy Across 13 Models

Document-embedding geometry divided the panel into 3 tiers based on average cosine. Extreme anisotropy (>0.92) was observed in Phi-3-mini (0.974), BioBERT (0.958), ClinicalBERT (0.930), and BERT-base-uncased (0.926). Moderate anisotropy (0.65-0.92) encompassed most general retrievers and LLMs. Reduced anisotropy (<0.65) was observed in Nomic-embed-text-nopfx (0.638), E5-Mistral-7B with instruction (0.546), and BioLORD-2023 (0.300). Across all 13 models, the final-layer PR showed a descriptive correlation with final-layer MRR@10 (Spearman ρ=0.74; n=13). Per-condition geometry is reported in Multimedia Appendix 6.

Per-model, layer-wise Spearman correlations between PR and MRR@10, and between average pairwise cosine and MRR@10, were computed across layers for the 11 configurations included in the full per-layer geometric correlation table in Multimedia Appendix 8. BERT-base-uncased and BioMistral-7B were extracted layer-wise for the trajectory metrics reported in Table 2 and for the rel_layer LME analysis, but were not included in the Multimedia Appendix 8 correlation table. Layer-wise PR-vs-MRR Spearman ρ ranged from +0.017 (MedCPT) to +0.989 (GTE-base), with a median of +0.939; the absolute value of the corresponding cosine-vs-MRR ρ was smaller than that of PR-vs-MRR for 9 of the 11 configurations. The 2 exceptions are MedCPT, where neither correlation reaches significance because of the layer-0 dual-encoder artifact discussed earlier, and instruction-tuned E5-Mistral-7B, whose layer-wise cosine-vs-MRR ρ is positive (+0.488; *P*=.004), opposite in sign to the other 10 configurations and consistent with this model’s anomalous instruction-conditioned trajectory. Across the 11 configurations, the PR is the more reliable layer-wise diagnostic. The full per-model correlation table is reported in Multimedia Appendix 8.

### Lexical Overlap Between BM25 and Embedding-Based Retrieval

BM25-vs-embedding Spearman rank correlations were low to moderate across the panel, ranging from near zero for E5-Mistral-7B (median ρ=–0.017) to 0.366 for GTE-base. No model exceeded ρ=0.37. The high BM25 alignment baselines therefore indicate that the generated queries are aligned with their targets, while the low-to-moderate BM25-embedding correlations show that embedding retrieval is not simply lexical matching.

### Chunking Sensitivity

For 4 representative models, BERT-base-uncased (general encoder), BGE-base (general retriever), MedCPT (biomedical retriever, dual-encoder), and BioMistral-7B (biomedical LLM), document embeddings were re-extracted using 3 chunking strategies (first-N tokens, last-N tokens, and full document, where N is each model’s maximum sequence length), and retrieval was re-evaluated. Mean MRR@10 across 6 corpus × query-format conditions are reported in Table 4.

First-N and Full produce identical inputs for the three 512-ceiling models (BERT-base-uncased, BGE-base, and MedCPT) because the Full strategy relies on the tokenizer’s default front-truncation at max_length, yielding the same leading N tokens as the explicit First-N slice regardless of document length. That some documents do exceed N tokens is separately established by the Last-N vs First-N gap for these 3 models (BERT-base 0.219 vs 0.250; BGE-base 0.790 vs 0.872; MedCPT 0.782 vs 0.816); if no document exceeded 512 tokens, First-N and Last-N would return identical text for these models. For BioMistral-7B (max_length=2048), Last-N essentially matches First/Full (0.423 vs 0.424), consistent with no document exceeding 2048 tokens in the present evaluation corpora. Last-N underperforms First/Full for the three 512-ceiling encoder/retriever models by 3-8 percentage points (BERT-base 0.219 vs 0.250=–0.031; BGE-base 0.790 vs 0.872=–0.082; MedCPT 0.782 vs 0.816=–0.034) and is essentially neutral for BioMistral-7B (0.423 vs 0.424=–0.001). The pattern is consistent with clinical notes structurally front-loading key diagnostic and presenting-complaint information; encoders without retrieval-specific positional weighting perform worse when the late portion is the only input. For BioMistral-7B, the 3 strategies coincide in the present evaluation because no document required truncation within the 2048-token context window.

The chunking sensitivity analysis assesses whether the principal findings are robust to alternative chunking choices. Results across the 4 representative models indicate that First-N chunking is equivalent to Full-document encoding under tokenizer-native leading truncation, and that retrieval performance is materially affected by the choice between document-head and document-tail truncation in the 512-ceiling encoder/retriever models. The principal findings are therefore not driven by the chunking choice.

**Table 4:** Chunking sensitivity for 4 representative models under token-level truncation.^a^

Model	First-N^b^	Full^c^	Last-N^d^
BERT-base	0.250	0.250	0.219
BGE-base	0.872	0.872	0.790
MedCPT	0.816	0.816	0.782
BioMistral	0.424	0.424	0.423

^a^N=2048 for BioMistral-7B. Full uses tokenizer-native leading truncation at max_length; therefore, for tokenizer-default front truncation, Full and First-N produce the same leading-token input. Values are the MRR@10 across the 6 corpus × query-format conditions.

^b^First-N=first N input tokens.

^c^Full=full document with tokenizer-level truncation at max_length.

^d^Last-N=last N input tokens. N is each model’s max_length: N=512 for BERT-base, BGE-base, and MedCPT.

### Synthetic Corpus Distributional Audit

To characterize differences between the synthetic and natural-text corpora, descriptive statistics were calculated; per-corpus document-length, vocabulary size, and type-token ratio statistics are reported in Multimedia Appendix 9.

The synthetic corpus has a mean document length that is approximately 30% shorter, a vocabulary that is 58% smaller, and a type-token ratio that is 40% lower than those of the natural-text corpora. These distributional differences explain why MedCPT and other retrieval models trained on real biomedical text underperform on synthetic data, and why corpus-only ZCA whitening is less effective on the synthetic corpus; the low-rank covariance structure of the more uniform synthetic corpus amplifies noise during whitening. This finding informs the discussion of the limitations and appropriateness of synthetic data.

### Post Hoc Interventions: Corpus-Only ZCA as Primary Methodology

Corpus-only ZCA was the primary post hoc intervention because it applies the transform only to document embeddings. Transductive ZCA was retained as a query-contaminated reference. Table 3 (cross-validated) shows the 2-tier pattern, and Multimedia Appendix 7 reports the aggregate intervention results averaged across the 6 corpus × query-format conditions: non–retrieval-trained tier 2 models generally improved under corpus-only ZCA, whereas retrieval-trained tier 1 models were degraded by additional whitening.

### Corpus-Dependent Collapse Patterns

Midlayer collapse varied across corpora: for BioBERT, MTSamples showed a substantial decline, whereas the synthetic corpus showed minimal collapse. Natural-language queries produced deeper troughs than keyword queries but recovered to match keyword performance at the final layer. At intermediate layers, model rankings inverted relative to the final layer (Kendall τ=–0.524 between layer-9 and layer-12 rankings of the 8 BERT-scale configurations); intermediate-layer representation quality does not predict final-layer retrieval performance. Per-corpus values are reported in Multimedia Appendix 6.

### Validation on Independent Corpora

The first-stage validation evaluated 8 BERT-scale configurations on 500 PMC-Patients case reports and 400 MTSamples medical transcriptions. Rankings were consistent with the primary experiment (PMC-500 ρ=0.952; *P*<.001; MTSamples-400 ρ=0.929; *P*=.001). Cross-source ranking between PMC case reports and medical transcriptions was high (ρ=0.976; *P*<.001), but this primarily reflects categorical stability, as the subset separates strongly into retrieval-trained and non–retrieval-trained groups.

### Independent Validation of the Remaining Configurations

The second-stage validation evaluated the 5 configurations not covered by the first-stage BERT-scale subset (BERT-base-uncased, BioMistral-7B, Phi-3-mini, E5-Mistral-7B, and E5-Mistral-7B-ablation)—4 LLM-scale configurations plus the BERT-base-uncased matched-comparison addition, which was not part of the original 8 BERT-scale configurations covered in the first stage—on the same expanded PMC-Patients (500) and MTSamples (400) corpora. Across the 20 evaluation conditions (5 models × 2 corpora × 2 query formats), Spearman rank correlation between primary-experiment final-layer MRR@10 and validation-corpus final-layer MRR@10 was ρ=0.839 (Kendall τ=0.632; *P*<.001; n=20). All 20 conditions showed lower MRR@10 on the validation scale than on the primary 100-document scale, consistent with the expected effect of larger candidate pools on retrieval difficulty. Per-model mean ΔMRR@10 (averaged across the 4 corpus-by-query-format conditions per model) ranged from –0.097 for E5-Mistral-7B (smallest mean drop) to –0.247 for E5-Mistral-7B-ablation (largest), with intermediate values for BERT-base-uncased (–0.109), BioMistral-7B (–0.218), and Phi-3-mini (–0.241). The rank ordering of the 5 configurations was preserved at validation scale: E5-Mistral-7B-ablation produced the highest validation MRR@10 in all 4 corpus-by-query-format cells (matching its ordering in the primary experiment), BERT-base-uncased the lowest in all 4, and the previously reported anomaly that E5-Mistral-7B-ablation outperforms its instruction-tuned counterpart in known-item retrieval was preserved across all 4 paired validation cells. Combined with the BERT-scale validation reported above, the 2 stages together provide independent-corpus rank-stability evidence for all 13 panel configurations. Per-condition raw MRR@10 values are reported in Multimedia Appendix 6.

## Discussion

### Principal Results

#### Overview

The results support 4 principal conclusions. First, anisotropy is widespread across model classes, and retrieval-objective training is associated with reduced anisotropy. Second, corpus-only ZCA explicitly reduces anisotropy and shows a 2-tier effect: it improves non–retrieval-trained models and degrades already calibrated retrieval-trained models. Third, layer-wise dynamics are heterogeneous rather than uniformly U-shaped: retrieval-trained encoders show midlayer collapse with strong final-layer recovery, non–retrieval-trained encoders recover incompletely, and decoder LLMs generally improve with depth. Fourth, BM25-overlap audits indicate that the findings are not merely lexical artifacts.

The central mechanistic finding is that high final-layer retrieval performance depends on final-layer geometric restructuring rather than on the absence of midlayer collapse. In retrieval-trained encoders, midlayer troughs are followed by participation-ratio expansion and reduced cosine collapse; in non–retrieval-trained encoders, the final layer remains anisotropic, and retrieval performance remains lower. This pattern aligns with the architecture of contrastive and instruction-tuning training objectives, which compute discrimination losses at the final-layer output, making the final layer the natural locus of representation specialization. In this reading, midlayer collapse in retrieval-trained encoders is a design property of the training objective rather than a failure mode; the failure mode is the absence of subsequent final-layer geometric restructuring, which distinguishes non–retrieval-trained encoders. This framing also explains why corpus-only ZCA can approximate the geometric pattern associated with retrieval-objective training: both are associated with reshaped final-layer geometry, one through training and the other through post hoc covariance correction.

#### Generalization to Larger and Noisier Corpora

The primary 100-document known-item design is smaller and simpler than production clinical retrieval. Validation at 4-5× scale partially addresses this, but production corpora contain near-duplicates, copy-forwarded text, abbreviations, templated sections, deidentification artifacts, and institution-specific terminology. ZCA effectiveness depends on the covariance structure, so direct testing on production-scale institutional corpora remains necessary.

### Comparison With Prior Work

The present analysis builds on and refines 3 threads of prior literature. First, Ethayarajh’s [9] characterization of anisotropy across BERT, GPT-2, and ELMo embeddings is corroborated in the clinical retrieval setting, with 2 additions: the 13-model layer-level analysis localizes anisotropy to specific model classes (extreme in non–retrieval-trained encoders and Phi-3-mini; moderate in retrieval-trained encoders and most LLMs; reduced in BioLORD-2023 and instruction-tuned E5-Mistral-7B), and the matched-comparison evidence supports training-objective choice rather than training-domain choice as the stronger explanatory axis for the observed anisotropy differences. Second, the geometric-correction literature is extended by Su et al’s [16] BERT-whitening, a transductive ZCA variant, to a deployment-relevant, corpus-only ZCA variant fitted to document embeddings without test-query information. The 2-tier pattern observed here (tier 2 models gain, tier 1 models degrade) refines the conventional framing of ZCA as a uniform improvement: the correction approximately reproduces the geometric pattern associated with retrieval-objective training. Tier membership is defined empirically by the response to whitening and, therefore, by the embedding geometry, rather than by the nominal training label. E5-Mistral-7B is the instructive exception: although it is retrieval-instruction-tuned, its decoder embeddings remain geometrically uncalibrated (final-layer average cosine 0.55, baseline MRR@10 0.32), and it shows the largest cross-validated gain in the panel (+0.304), placing it with the tier 2 models. This dissociation between the training label and geometric calibration reinforces the central claim that it is the geometry of the final-layer representation, not the training objective per se, that determines whether post hoc whitening helps. A directly parallel pattern has recently been reported in the code-search domain by Diera et al [15], who applied Soft-ZCA whitening to 3 code language models (CodeBERT, CodeT5+, and Code Llama) and observed the same 2-tier effect: standard ZCA improved pretrained models substantially but degraded contrastively fine-tuned variants, with the latter requiring much smaller eigenvalue regularizers for benefit. Where retrieval-calibrated geometry is absent, ZCA tends to help; where such geometry is already present, additional whitening tends to disrupt. Third, the clinical embedding benchmarking literature, including Myers et al [17] on clinical-trial retrieval, Soffer et al [18] on 39 embedding models across 7 medical semantic similarity tasks reframed as retrieval problems, Khodadad et al [35] on a 51-task medical embedding benchmark spanning classification, clustering, and retrieval, and the companion benchmark study [3] across 3 clinical corpora and 294 conditions, is extended along 2 dimensions: layer-level analysis replaces final-layer-only evaluation, and matched-architectural controls begin to separate training-objective from training-domain contributions to performance gaps. The present manuscript is positioned as a mechanistic companion to [3]: the empirical performance claim is replicated and localized rather than displaced.

### Limitations

The study has several limitations. The primary corpora contain only 100 documents each and include a single known-relevant document per query, whereas production retrieval involves larger indices and multiple relevant documents. Queries were generated from metadata rather than written by clinicians, so they may be more label-like and less noisy than real clinical queries. The synthetic corpus differs from natural text in length, vocabulary size, and type-token ratio, limiting the generalizability of results from synthetic-only experiments.

The LME uses a random-slope specification, fit separately for each of the 13 configurations, with each query treated as a distinct group within its source corpus. All models converged, showing substantial between-query variance (median intraclass correlation coefficient 0.41), computed as the variance attributable to the query random intercept divided by the total variance, per Nakagawa and Schielzeth [36]. Quadratic sensitivity analyses support the substantive conclusions. Recovery ratio and PR-expansion thresholds are data-derived and should not be treated as deployment criteria. Geometry-retrieval correlations are descriptive and computed across nonindependent observations. Finally, this is a single-author study.

Two further limitations and one observation merit explicit treatment. First, the known-item retrieval design assigns one relevant document per query, whereas many production clinical RAG settings involve multiple relevant documents per query, near-duplicates, and copy-forwarded notes. Multirelevant-document scoring (graded relevance, normalized discounted cumulative gain, or set-based recall) may yield different model orderings than the binary MRR@10/recall@10 used here, particularly for retrieval-trained models whose contrastive objective is optimized for single-document discrimination. Second, exploratory query-level inspection of failure cases (queries where final-layer MRR@10 was below 0.10 in the primary experiment) did not reveal a systematic pattern associated with query length, clinical specialty, or BM25-vs-embedding rank divergence; this is consistent with the BM25-vs-embedding Spearman rank correlations of –0.02 to 0.37 reported above, which indicate that embedding retrieval relies on substantial nonlexical signal that the present analysis does not further decompose. Third, the cross-source ranking stability (ρ=0.976 between PMC case reports and MTSamples medical transcriptions) primarily reflects the bimodal separation of the 8 BERT-scale subset into retrieval-trained and non–retrieval-trained groups, rather than fine-grained within-group rank stability.

The corpus-only ZCA cross-validation fits the whitening transform from 80 training-fold documents per fold in a 768- to 4096-dimensional hidden-state space; the resulting sample covariance is rank-deficient by 689-4017 dimensions, and the whitening transform relies heavily on the ε ridge regularizer to be well-defined outside the empirical ~80-dimensional subspace. For the corpus covariance to be approximately full-rank (eigenvalue magnitudes dominating ε across all relevant dimensions), the number of training-fold documents must approach or exceed the hidden-state dimensionality, at least ~768 documents for BERT-scale models and ~4096 for LLM-scale. Institutional EHR corpora at the scale of 10^4^-10^6^ documents are therefore expected to produce well-conditioned covariance estimates across all model classes evaluated here. The 80-document fold rank-deficiency is best understood as a methodological constraint of the diagnostic cross-validation design rather than a property of the intervention itself: at deployment scale, the covariance estimate is expected to be well-conditioned, the ε regularizer to be immaterial relative to the data-driven eigenstructure, and the ZCA intervention’s behavior to be governed by corpus geometry rather than small-sample subspace structure. Prospective evaluation at institutional scale is therefore required before any operational claim about ZCA-based correction at scale can be supported.

### Future Work

Production-scale validation on institutional EHR corpora is the most important next step. Such validation should include (1) multirelevant-document labeling with graded relevance scoring rather than known-item retrieval; (2) clinician-authored queries reflecting real information needs rather than metadata-derived queries; (3) larger candidate indices in the 10^4^-10^6^ document range; (4) near-duplicate notes, copy-forwarded text, abbreviations, templated sections, and deidentification artifacts characteristic of production EHRs; (5) temporal-drift testing across calendar periods to detect concept-drift effects on embedding geometry; (6) integration with reranking and approximate-nearest-neighbor indexing layers downstream of embedding retrieval; and (7) prospective failure analysis of retrieval misses against clinician-judged relevance. The corpus-only ZCA whitening intervention should be re-evaluated under each of these conditions before being considered for clinical deployment; the synthetic-corpus failure observed here suggests that ZCA effectiveness depends materially on the covariance structure of the deployment corpus. The matched-comparison framework introduced here should be extended to include additional clean architectural pairs as new domain-trained and retrieval-trained models become available, particularly matched LLM-scale pairs that differ only in training objective. None of the screening thresholds reported in this manuscript should be treated as deployment criteria without independent prospective validation.

Matched contrasts on a single underlying model are also required to separate the pooling-attributable from the instruction-formatting-attributable components of the E5-Mistral-7B-ablation’s small post-ZCA gain. Specifically, mean-pooled and EOS-pooled representations should be compared under the same instruction-formatting protocol, and instruction-prefixed vs no-prefix representations should be compared under the same pooling strategy. These contrasts were not performed in this study and are identified as specific follow-up investigations.

### Conclusions

Across 13 transformer configurations, retrieval performance was strongly associated with embedding geometry. Retrieval-specific training was associated with lower anisotropy, whereas corpus-only ZCA reduced anisotropy explicitly as a post hoc correction. The resulting 2-tier pattern is the central finding: non–retrieval-trained models generally improved under ZCA, whereas retrieval-trained models were degraded by additional whitening. Matched architectural contrasts support the training objective rather than the biomedical training domain as the stronger explanatory axis, although residual confounding remains.

These findings provide a mechanistic basis for layer-level diagnostics and post hoc geometric correction in clinical retrieval benchmarks. They do not constitute deployment guidance. Institutional EHR validation using real queries, larger indices, multirelevant-document labels, and downstream RAG evaluation is required before clinical deployment.

## Appendix

Multimedia Appendix 1 GPT-4o prompt used to generate metadata-derived queries (full prompt text, temperature, format examples).

Multimedia Appendix 2 Best Match 25 alignment-recovery audit for the Synthetic corpus used in the mixed-effects sensitivity check—full pair-recovery table.

Multimedia Appendix 3 Document-length tercile analysis with token boundaries and per-tercile retrieval metrics for the 13 models—descriptive.

Multimedia Appendix 4 Per-query linear mixed-effects fixed-effect coefficients and variance summary for all 13 models (each fit with 300 query groups), including fixed-effect coefficients, standard errors, z values, and *P* values; intraclass correlation coefficients ranged from 0.06 to 0.55 (median 0.41).

Multimedia Appendix 5 Epsilon sensitivity sweep results for corpus-only zero-phase component analysis across 6 ε values × 13 models × 6 conditions.

Multimedia Appendix 6 Per-condition raw (nonnormalized) final-layer mean reciprocal rank at cutoff 10 and recall at cutoff 10 for the 11 panel configurations included in this per-condition raw-values table; BERT-base-uncased and BioMistral-7B were extracted layer-wise for the Table 2 trajectory metrics and rel_layer linear mixed-effects analysis, but were not included in this table.

Multimedia Appendix 7 Per-model final-layer intervention summary: baseline, corpus-only zero-phase component analysis, and transductive zero-phase component analysis, averaged across 3 corpora × 2 query formats for all 13 models.

Multimedia Appendix 8 Per-model layer-wise Spearman correlations between geometric measures (participation ratio, average pairwise cosine) and mean reciprocal rank at cutoff 10 across layers, for the 11 configurations included in the full per-layer geometric correlation table. Includes PR-vs-MRR ρ, cosine-vs-MRR ρ, *P* values, and the per-model PR-stronger-than-cosine comparison.

Multimedia Appendix 9 Synthetic corpus distributional audit: per-corpus document-length, vocabulary size, and type-token ratio statistics for MTSamples, PMC-Patients, and the model-generated Synthetic corpus.

## Abbreviations

BEIR: Benchmarking Information Retrieval
BGE: Beijing Academy of Artificial Intelligence General Embedding
BM25: Best Match 25
EHR: electronic health record
EOS: end of sequence
GTE: General Text Embeddings
LLM: large language model
LME: linear mixed-effects
MRR: mean reciprocal rank
MRR@10: mean reciprocal rank at cutoff 10
PR: participation ratio
RAG: retrieval-augmented generation
Recall@10: recall at cutoff 10
ZCA: zero-phase component analysis

## Data Availability

Code, layer-level results, intervention data, validation results, analysis scripts, and figure-generation code are available in the GitHub repository for this study, with archived snapshots at Zenodo [37] under a CC BY 4.0 license. The compute scripts and outputs are included in the same repository. Query sets, synthetic clinical notes, and the MTSamples cache used here are available in the companion study repository [38]. The ZCA whitening implementation evaluated here is available as the clinical-embedding-fix software library, deposited on Zenodo [39]. PMC-Patients data can be reproduced from the zhengyun21/PMC-Patients dataset using the seeds specified in the analysis scripts.
