# Supplementary material for: Effects of Model Choice, Corpus Context, and Post Hoc Correction on Layer-Level Embedding Degradation in Clinical Document Retrieval: Experimental Study
Source: JMIR Med Inform. 2026 Jul 29;14:e99639. doi: 10.2196/99639 (PMC13469822; doi:10.2196/99639)
Supplement: Supplementary file 1 [file medinform_v14i1e99639_app1.docx]

# Multimedia Appendix 1

GPT-4o prompt templates used to generate metadata-derived queries for the layer-level retrieval evaluation. Queries were generated from document metadata only (specialty, note type, primary diagnosis, secondary diagnoses, patient demographics) and never from the document body text, by design, to limit lexical leakage from document into query. Model: gpt-4o (OpenAI Chat Completions API). Temperature: 0.3. Max tokens: 150. Up to 3 retry attempts per call with exponential backoff; on persistent failure the primary diagnosis string was used as a fallback query. Generation covered all documents across the 3 corpora in both query formats (natural-language and keyword).

## Natural-language query prompt

Based ONLY on this metadata, write a natural language clinical question (1-2 sentences):
Specialty: {specialty} | Note type: {note_type} | Diagnosis: {primary_diagnosis} | Other: {secondary} | Patient: {demographics}
Query:

## Keyword query prompt

Based ONLY on this metadata, output 3-6 clinical search keywords (space-separated):
Specialty: {specialty} | Note type: {note_type} | Diagnosis: {primary_diagnosis} | Other: {secondary} | Patient: {demographics}
Keywords:

## Placeholder fields

Placeholder fields were populated per document from extracted metadata: {specialty} = document specialty (default 'Unknown'); {note_type} = note type (default 'Clinical Note'); {primary_diagnosis} = primary diagnosis or chief complaint (default 'Unknown'); {secondary} = comma-separated secondary diagnoses (default 'none'); {demographics} = patient age range and sex (default 'adult'). The metadata fields were themselves extracted from each document in a separate GPT-4o metadata-extraction step that returned structured JSON; the query-generation step above received only the extracted metadata, never the document text.
