# Supplementary material for: Effects of Model Choice, Corpus Context, and Post Hoc Correction on Layer-Level Embedding Degradation in Clinical Document Retrieval: Experimental Study
Source: JMIR Med Inform. 2026 Jul 29;14:e99639. doi: 10.2196/99639 (PMC13469822; doi:10.2196/99639)
Supplement: Supplementary file 2 [file medinform_v14i1e99639_app2.docx]

# Multimedia Appendix 2

Synthetic corpus alignment-recovery audit, addressing Reviewer AL comment 3.3. The original layer-extraction pipeline preserved the document ordering against which the metadata-derived queries were generated. The locally cached Synthetic CSV used to reload the corpus for the per-query mixed-effects sensitivity analysis had subsequently been re-sorted by synth_id, breaking that index alignment. Query–document pairing for the sensitivity analysis was therefore recovered using BM25 top-1 matching with greedy one-to-one deduplication: each keyword query was scored against all candidate documents with BM25 (Okapi), and assigned to its highest-scoring not-yet-assigned document, enforcing a one-to-one mapping.

## Recovery procedure

1. 100 synthetic clinical notes (generated via Mistral-7B-Instruct-v0.2 in the companion study) were loaded from the repository CSV.
2. 100 keyword queries (generated via GPT-4o from document metadata) were tokenized and scored against all 100 documents using BM25 Okapi.
3. Each query was assigned to its highest-scoring document not already assigned to an earlier query (greedy one-to-one deduplication).
4. The recovered alignment was validated by re-running BM25 on the reordered documents and computing MRR@10 against the diagonal (each query's assigned document).

## Recovery results

| **Audit metric** | **Value** |
| --- | --- |
| Total synthetic queries (keyword) | 100 |
| Candidate documents | 100 |
| Queries assigned to a unique document (1-to-1) | 71 |
| Queries reassigned to next-best document after collision | 29 |
| BM25 MRR@10 on recovered alignment (sanity check) | 0.963 |
| Expected BM25 MRR@10 (companion-study baseline) | ≈0.96 |

## Interpretation and impact on primary analyses

The 29 collisions arise because multiple metadata-derived keyword queries share overlapping clinical terminology and therefore best-match the same document under BM25; the greedy deduplication resolves these by assigning each colliding query to its next-best available document. The BM25 MRR@10 of 0.963 on the recovered alignment matches the companion-study Synthetic baseline (≈0.96), confirming the recovery is lexically sound. Critically, the recovered alignment is used only in the per-query mixed-effects sensitivity analysis; the per-query layer-wise linear mixed-effects model reported in the main Results uses ranks derived from the original-pipeline alignment, not the recovered alignment. Alignment recovery therefore does not enter the inferential pathway for any principal statistical result.
