# Supplementary material for: Effects of Model Choice, Corpus Context, and Post Hoc Correction on Layer-Level Embedding Degradation in Clinical Document Retrieval: Experimental Study
Source: JMIR Med Inform. 2026 Jul 29;14:e99639. doi: 10.2196/99639 (PMC13469822; doi:10.2196/99639)
Supplement: Supplementary file 3 [file medinform_v14i1e99639_app3.docx]

# Multimedia Appendix 3

Document-length tercile analysis, addressing Editorial comment 8. For each corpus, the 100-document primary working set was partitioned into length terciles by whitespace-tokenized word count, using the 33.3rd and 66.7th percentiles as boundaries (computed per corpus). Per-tercile sample sizes are approximately 33 documents. This analysis is exploratory; with ~33 documents per stratum the per-tercile point estimates are statistically underpowered and are reported descriptively rather than as inferential tests. The exact token boundaries are provided below to allow replication.

## Token boundaries per corpus (primary 100-document subset)

| **Corpus** | **Short (≤ words)** | **Medium (words)** | **Long (> words)** | **n per tercile** |
| --- | --- | --- | --- | --- |
| MTSamples | 286 | 287–453 | 453 | ~33 |
| PMC-Patients | 331 | 332–518 | 518 | ~33 |
| Synthetic | 290 | 291–308 | 308 | ~33 |

*Boundaries are word-count percentiles of each corpus's 100-document working set. The Synthetic corpus shows a notably compressed length distribution (medium tercile spans only 290–308 words), consistent with the structural uniformity discussed in the main text and Multimedia Appendix 10; this uniformity is relevant to the synthetic-corpus corpus-only ZCA failure.*

## Per-tercile final-layer MRR@10 (selected models)

| **Model** | **Short MRR@10** | **Medium MRR@10** | **Long MRR@10** |
| --- | --- | --- | --- |
| BGE-base | 0.833 | 0.894 | 0.912 |
| E5-Mistral-7B | 0.418 | 0.316 | 0.221 |
| BioBERT | 0.478 | 0.360 | 0.361 |
| ClinicalBERT | 0.305 | 0.176 | 0.212 |
| MedCPT | 0.779 | 0.855 | 0.848 |

*Final-layer MRR@10 averaged across the 3 corpora and 2 query formats per length tercile. Values are descriptive; the ~33-document per-tercile sample precludes reliable inference. Full per-model, per-corpus, per-tercile values are available in the project data repository.*
