# Supplementary material for: Effects of Model Choice, Corpus Context, and Post Hoc Correction on Layer-Level Embedding Degradation in Clinical Document Retrieval: Experimental Study
Source: JMIR Med Inform. 2026 Jul 29;14:e99639. doi: 10.2196/99639 (PMC13469822; doi:10.2196/99639)
Supplement: Supplementary file 4 [file medinform_v14i1e99639_app4.docx]

# Multimedia Appendix 4

Per-query linear mixed-effects model results for all 13 panel configurations. The outcome is log(rank+1) for each query at each relative layer depth; the specification is log_rank ~ C(intervention) + C(corpus) + C(query_format) + rel_layer, with a random intercept and random slope on rel_layer by query identity. Each query is treated as a distinct random-effects group within its source corpus, giving 300 groups per model (100 queries x 3 corpora); models are fit separately per configuration in statsmodels using REML with the L-BFGS optimizer. All 13 configurations converged. The intraclass correlation coefficient ranged from 0.06 to 0.55 (median 0.41), indicating that between-query difficulty accounts for a substantial share of variance once the fixed effects are modeled; this supports the per-query random-effects specification. Reference levels: intervention = baseline (no whitening); corpus = MTSamples; query format = keyword.

## Panel A. Model-level variance summary and ICC

| Model | n obs | n groups | Random-effect var | Residual var | ICC | Log-likelihood |
| --- | --- | --- | --- | --- | --- | --- |
| BERT-base-uncased | 23,400 | 300 | 0.621 | 0.844 | 0.424 | -32063 |
| BGE-base | 23,400 | 300 | 0.673 | 0.907 | 0.426 | -32809 |
| BioBERT | 23,400 | 300 | 0.551 | 0.789 | 0.411 | -31239 |
| BioLORD-2023 | 23,400 | 300 | 0.791 | 0.635 | 0.555 | -28787 |
| BioMistral-7B | 59,400 | 300 | 0.301 | 0.870 | 0.257 | -81129 |
| ClinicalBERT | 12,600 | 300 | 0.479 | 0.897 | 0.348 | -17768 |
| E5-Mistral-7B | 59,400 | 300 | 0.207 | 0.895 | 0.188 | -82067 |
| E5-Mistral-7B-ablation | 59,400 | 300 | 0.312 | 0.835 | 0.272 | -79914 |
| GTE-base | 23,400 | 300 | 0.721 | 0.920 | 0.439 | -32971 |
| MedCPT | 23,400 | 300 | 0.123 | 1.887 | 0.061 | -40967 |
| Nomic-embed-text | 23,400 | 300 | 0.742 | 0.674 | 0.524 | -29419 |
| Nomic-embed-text-nopfx | 23,400 | 300 | 0.716 | 0.709 | 0.503 | -29968 |
| Phi-3-mini | 59,400 | 300 | 0.245 | 0.814 | 0.231 | -79171 |

*ICC = random-effect variance / (random-effect variance + residual variance). Variance values are on the log(rank+1) scale. All 13 models converged with positive between-query variance (median ICC 0.41); MedCPT shows the lowest ICC, consistent with its dual-encoder layer-0 artifact described in the main text.*

## Panel B. Fixed-effect coefficients (all 13 models)

| Model | Term | Estimate | SE | z | *P* |
| --- | --- | --- | --- | --- | --- |
| BERT-base-uncased | Intercept | 2.241 | 0.077 | 29.25 | <.001 |
|  | Intervention: corpus-only ZCA | -0.581 | 0.015 | -39.53 | <.001 |
|  | Intervention: transductive ZCA | -0.986 | 0.015 | -67.06 | <.001 |
|  | Corpus: PMC-Patients | 0.187 | 0.103 | 1.81 | .070 |
|  | Corpus: Synthetic | 0.509 | 0.103 | 4.92 | <.001 |
|  | Query format: natural language | -0.038 | 0.012 | -3.14 | .002 |
|  | rel_layer (relative depth) | 0.624 | 0.045 | 13.99 | <.001 |
| BioBERT | Intercept | 2.143 | 0.076 | 28.01 | <.001 |
|  | Intervention: corpus-only ZCA | -0.460 | 0.014 | -32.36 | <.001 |
|  | Intervention: transductive ZCA | -0.834 | 0.014 | -58.64 | <.001 |
|  | Corpus: PMC-Patients | 0.083 | 0.108 | 0.77 | .441 |
|  | Corpus: Synthetic | 0.505 | 0.106 | 4.77 | <.001 |
|  | Query format: natural language | -0.025 | 0.012 | -2.11 | .035 |
|  | rel_layer (relative depth) | 0.224 | 0.036 | 6.27 | <.001 |
| ClinicalBERT | Intercept | 2.780 | 0.074 | 37.50 | <.001 |
|  | Intervention: corpus-only ZCA | -0.603 | 0.021 | -29.19 | <.001 |
|  | Intervention: transductive ZCA | -1.043 | 0.021 | -50.46 | <.001 |
|  | Corpus: PMC-Patients | 0.203 | 0.102 | 2.00 | .046 |
|  | Corpus: Synthetic | 0.453 | 0.102 | 4.43 | <.001 |
|  | Query format: natural language | -0.329 | 0.017 | -19.51 | <.001 |
|  | rel_layer (relative depth) | 0.200 | 0.033 | 6.04 | <.001 |
| BGE-base | Intercept | 2.233 | 0.070 | 31.92 | <.001 |
|  | Intervention: corpus-only ZCA | -0.754 | 0.015 | -49.42 | <.001 |
|  | Intervention: transductive ZCA | -0.928 | 0.015 | -60.84 | <.001 |
|  | Corpus: PMC-Patients | -0.085 | 0.087 | -0.98 | .327 |
|  | Corpus: Synthetic | 0.581 | 0.085 | 6.81 | <.001 |
|  | Query format: natural language | 0.144 | 0.013 | 11.58 | <.001 |
|  | rel_layer (relative depth) | -0.165 | 0.041 | -4.06 | <.001 |
| GTE-base | Intercept | 2.234 | 0.070 | 32.12 | <.001 |
|  | Intervention: corpus-only ZCA | -0.757 | 0.015 | -49.30 | <.001 |
|  | Intervention: transductive ZCA | -0.948 | 0.015 | -61.67 | <.001 |
|  | Corpus: PMC-Patients | -0.099 | 0.082 | -1.20 | .230 |
|  | Corpus: Synthetic | 0.629 | 0.082 | 7.72 | <.001 |
|  | Query format: natural language | 0.122 | 0.013 | 9.72 | <.001 |
|  | rel_layer (relative depth) | -0.134 | 0.043 | -3.16 | .002 |
| BioLORD-2023 | Intercept | 1.378 | 0.076 | 18.02 | <.001 |
|  | Intervention: corpus-only ZCA | 0.128 | 0.013 | 10.02 | <.001 |
|  | Intervention: transductive ZCA | 0.033 | 0.013 | 2.57 | .010 |
|  | Corpus: PMC-Patients | -0.084 | 0.097 | -0.87 | .385 |
|  | Corpus: Synthetic | 0.789 | 0.096 | 8.24 | <.001 |
|  | Query format: natural language | 0.004 | 0.010 | 0.37 | .708 |
|  | rel_layer (relative depth) | -0.226 | 0.043 | -5.21 | <.001 |
| MedCPT | Intercept | 3.133 | 0.041 | 76.65 | <.001 |
|  | Intervention: corpus-only ZCA | 0.062 | 0.022 | 2.81 | .005 |
|  | Intervention: transductive ZCA | -0.200 | 0.022 | -9.10 | <.001 |
|  | Corpus: PMC-Patients | -0.019 | 0.047 | -0.41 | .683 |
|  | Corpus: Synthetic | 0.125 | 0.048 | 2.62 | .009 |
|  | Query format: natural language | 0.061 | 0.018 | 3.39 | <.001 |
|  | rel_layer (relative depth) | -0.391 | 0.040 | -9.90 | <.001 |
| Nomic-embed-text | Intercept | 1.998 | 0.073 | 27.24 | <.001 |
|  | Intervention: corpus-only ZCA | -0.485 | 0.013 | -36.92 | <.001 |
|  | Intervention: transductive ZCA | -0.809 | 0.013 | -61.54 | <.001 |
|  | Corpus: PMC-Patients | -0.054 | 0.091 | -0.59 | .555 |
|  | Corpus: Synthetic | 0.399 | 0.090 | 4.41 | <.001 |
|  | Query format: natural language | -0.049 | 0.011 | -4.56 | <.001 |
|  | rel_layer (relative depth) | -0.176 | 0.038 | -4.67 | <.001 |
| Nomic-embed-text-nopfx | Intercept | 2.125 | 0.072 | 29.50 | <.001 |
|  | Intervention: corpus-only ZCA | -0.564 | 0.013 | -41.82 | <.001 |
|  | Intervention: transductive ZCA | -0.867 | 0.013 | -64.30 | <.001 |
|  | Corpus: PMC-Patients | -0.089 | 0.089 | -1.00 | .319 |
|  | Corpus: Synthetic | 0.410 | 0.088 | 4.64 | <.001 |
|  | Query format: natural language | -0.091 | 0.011 | -8.23 | <.001 |
|  | rel_layer (relative depth) | -0.206 | 0.036 | -5.73 | <.001 |
| E5-Mistral-7B | Intercept | 3.997 | 0.045 | 89.51 | <.001 |
|  | Intervention: corpus-only ZCA | -0.223 | 0.009 | -23.47 | <.001 |
|  | Intervention: transductive ZCA | -0.297 | 0.009 | -31.29 | <.001 |
|  | Corpus: PMC-Patients | 0.006 | 0.064 | 0.10 | .923 |
|  | Corpus: Synthetic | 0.168 | 0.064 | 2.65 | .008 |
|  | Query format: natural language | -0.097 | 0.008 | -12.54 | <.001 |
|  | rel_layer (relative depth) | -1.773 | 0.072 | -24.54 | <.001 |
| E5-Mistral-7B-ablation | Intercept | 3.414 | 0.056 | 61.34 | <.001 |
|  | Intervention: corpus-only ZCA | -0.464 | 0.009 | -50.47 | <.001 |
|  | Intervention: transductive ZCA | -1.660 | 0.009 | -180.74 | <.001 |
|  | Corpus: PMC-Patients | 0.015 | 0.077 | 0.19 | .847 |
|  | Corpus: Synthetic | 0.379 | 0.078 | 4.86 | <.001 |
|  | Query format: natural language | -0.167 | 0.007 | -22.32 | <.001 |
|  | rel_layer (relative depth) | -1.317 | 0.038 | -34.52 | <.001 |
| BioMistral-7B | Intercept | 3.227 | 0.056 | 57.84 | <.001 |
|  | Intervention: corpus-only ZCA | -0.393 | 0.009 | -41.89 | <.001 |
|  | Intervention: transductive ZCA | -1.613 | 0.009 | -172.05 | <.001 |
|  | Corpus: PMC-Patients | 0.036 | 0.078 | 0.46 | .642 |
|  | Corpus: Synthetic | 0.376 | 0.079 | 4.74 | <.001 |
|  | Query format: natural language | -0.161 | 0.008 | -21.06 | <.001 |
|  | rel_layer (relative depth) | -0.890 | 0.038 | -23.51 | <.001 |
| Phi-3-mini | Intercept | 3.283 | 0.050 | 65.67 | <.001 |
|  | Intervention: corpus-only ZCA | -0.578 | 0.009 | -63.69 | <.001 |
|  | Intervention: transductive ZCA | -1.982 | 0.009 | -218.48 | <.001 |
|  | Corpus: PMC-Patients | 0.042 | 0.070 | 0.60 | .549 |
|  | Corpus: Synthetic | 0.376 | 0.069 | 5.45 | <.001 |
|  | Query format: natural language | -0.167 | 0.007 | -22.57 | <.001 |
|  | rel_layer (relative depth) | -0.545 | 0.041 | -13.16 | <.001 |

*Outcome is log(rank+1); negative rel_layer indicates improving retrieval (lower rank) with depth, positive indicates worsening. Three models worsen with depth (BERT-base-uncased, BioBERT, ClinicalBERT); the other 10 improve. P-values reported descriptively without multiple-comparison correction.*
