# Supplementary material for: Effects of Model Choice, Corpus Context, and Post Hoc Correction on Layer-Level Embedding Degradation in Clinical Document Retrieval: Experimental Study
Source: JMIR Med Inform. 2026 Jul 29;14:e99639. doi: 10.2196/99639 (PMC13469822; doi:10.2196/99639)
Supplement: Supplementary file 5 [file medinform_v14i1e99639_app5.docx]

# Multimedia Appendix 5

Epsilon sensitivity sweep for corpus-only ZCA whitening. The ridge regularizer ε in the whitening transform W = U(Lambda + epsilon I)^(-1/2) U^T was swept across six values (1e-7 to 1e-2) for all 13 panel configurations. Each cell reports mean ΔMRR@10 (corpus-only ZCA minus baseline) averaged across the 6 corpus × query-format conditions (n=6 per cell). The value ε=1e-5 used in the main analysis is a defensible global choice but is not uniformly optimal: Tier 2 (non-retrieval-trained) models reach their largest positive ΔMRR@10 at ε in the 1e-5 to 1e-3 range (most at 1e-4), whereas Tier 1 (retrieval-trained) models are degraded by corpus-only ZCA at every ε value tested, with the degradation merely smallest at the largest ε (1e-2). This asymmetry reinforces the two-tier interpretation: retrieval-objective training already performs the geometric calibration that corpus-only ZCA approximates, so no ε setting makes additional whitening beneficial for Tier 1 models.

| Model | Tier | ε=1e-7 | ε=1e-6 | ε=1e-5 | ε=1e-4 | ε=1e-3 | ε=1e-2 |
| --- | --- | --- | --- | --- | --- | --- | --- |
| E5-Mistral-7B | 2 | +0.078 | +0.164 | **+0.203** | +0.199 | +0.169 | +0.050 |
| BioBERT | 2 | +0.035 | +0.036 | +0.063 | **+0.122** | +0.078 | -0.025 |
| ClinicalBERT | 2 | +0.043 | +0.044 | +0.053 | **+0.092** | +0.084 | +0.002 |
| BioMistral-7B | 2 | -0.039 | +0.055 | +0.059 | **+0.069** | +0.065 | +0.016 |
| BERT-base-uncased | 2 | +0.023 | +0.024 | +0.037 | **+0.059** | +0.039 | -0.020 |
| Phi-3-mini | 2 | -0.029 | +0.028 | +0.039 | **+0.053** | +0.045 | +0.015 |
| E5-Mistral-7B-ablation | 2 | -0.158 | -0.016 | -0.016 | -0.001 | **+0.035** | +0.011 |
| BGE-base | 1 | -0.074 | -0.073 | -0.073 | -0.060 | -0.026 | **-0.008** |
| GTE-base | 1 | -0.247 | -0.105 | -0.069 | -0.058 | -0.032 | **-0.012** |
| BioLORD-2023 | 1 | -0.081 | -0.082 | -0.079 | -0.075 | -0.037 | **-0.011** |
| Nomic-embed-text | 1 | -0.086 | -0.086 | -0.085 | -0.070 | -0.029 | **-0.010** |
| Nomic-embed-text-nopfx | 1 | -0.083 | -0.082 | -0.081 | -0.065 | -0.032 | **-0.010** |
| MedCPT | 1 | -0.138 | -0.138 | -0.135 | -0.118 | -0.049 | **-0.017** |

*Bold = per-model peak (largest mean ΔMRR@10 across the ε sweep). For Tier 2 models the peak is a positive gain; for Tier 1 models the bold cell marks the least-negative value, not a benefit. Per-condition (corpus × query-format) values underlying these means are available in the project data repository.*
