# Supplementary material for: Effects of Model Choice, Corpus Context, and Post Hoc Correction on Layer-Level Embedding Degradation in Clinical Document Retrieval: Experimental Study
Source: JMIR Med Inform. 2026 Jul 29;14:e99639. doi: 10.2196/99639 (PMC13469822; doi:10.2196/99639)
Supplement: Supplementary file 6 [file medinform_v14i1e99639_app6.docx]

**Multimedia Appendix 6**

Layer-wise per-condition retrieval and geometric values for the 11 panel configurations included in this table, across the 3 corpora (MTSamples, PMC-Patients, Synthetic) and 2 query formats (keyword, natural language). One row is reported per model × corpus × query format × layer, giving 1182 rows in total. Layer 0 denotes the input embedding layer and the final layer is the model maximum; rel_layer = layer / max_layer. ClinicalBERT has 7 layers because its checkpoint is DistilBERT-based; E5-Mistral-7B, E5-Mistral-7B-ablation and Phi-3-mini have 33 layers; the remaining models have 13. BERT-base-uncased and BioMistral-7B were extracted layer-wise for the Table 2 trajectory metrics and the rel_layer LME analysis, but layer-wise geometry was not retained for those two configurations and they are therefore not included in this table. All values are computed on the full-length document set (length tercile = all) at each layer with no post hoc intervention applied; intervention results are reported in Multimedia Appendix 7. MRR@10 and Recall@10 are from the layer-wise trajectory extraction and average to values within 0.4-1.4% of the intervention-pipeline final-layer baseline used in Tables 2 and 3 (BGE-base 0.879 vs 0.872; MedCPT 0.828 vs 0.816; BioLORD-2023 0.804 vs 0.798; E5-Mistral-7B-ablation 0.666 vs 0.663); Tables 2 and 3 adopt the intervention-pipeline baseline for consistency with the cross-validated corpus-only ZCA analysis and the chunking sensitivity analysis in Table 4. Two normalizations are provided, both min-max rescalings to the 0-1 range. MRR@10 norm rescales MRR@10 across layers within each model × corpus × query format condition. MRR@10 model mean is MRR@10 averaged across the 6 conditions at each layer, and MRR@10 model mean norm is that condition-averaged trajectory rescaled across layers within each model; the latter pair reproduces the trajectories plotted in Figure 1, panels A and B respectively, and repeats across the 6 condition rows of a given model × layer cell. CI lower and CI upper are nonparametric bootstrap 95% percentile intervals for MRR@10, resampling the 100 queries within each condition with 2000 replicates (seed 42); they are computed from the per-query rank data and contain the tabulated MRR@10 in 100% of rows. Participation ratio, average pairwise cosine and anisotropy are computed on document embeddings and are therefore identical across the 2 query formats within a given model × corpus × layer cell. Anisotropy is the singular-value-decomposition-based measure defined in the Methods section.

| **Model** | **Corpus** | **Query format** | **Layer** | **rel_layer** | **MRR@10** | **MRR@10 norm** | **CI lower** | **CI upper** | **Recall@10** | **MRR@10 model mean** | **MRR@10 model mean norm** | **Participation ratio** | **Avg cosine** | **Anisotropy** |
| --- | --- | --- | --- | --- | --- | --- | --- | --- | --- | --- | --- | --- | --- | --- |
| BGE-base | MTSamples | keyword | 0 | 0.0000 | 0.5841 | 0.6079 | 0.5092 | 0.6602 | 0.8400 | 0.3931 | 0.3912 | 21.0157 | 0.8933 | 0.1288 |
| BGE-base | MTSamples | keyword | 1 | 0.0833 | 0.5926 | 0.6177 | 0.5117 | 0.6781 | 0.8200 | 0.4003 | 0.4002 | 21.3471 | 0.9173 | 0.1206 |
| BGE-base | MTSamples | keyword | 2 | 0.1667 | 0.4329 | 0.4336 | 0.3551 | 0.5168 | 0.6500 | 0.2979 | 0.2718 | 19.6801 | 0.9417 | 0.1268 |
| BGE-base | MTSamples | keyword | 3 | 0.2500 | 0.2106 | 0.1773 | 0.1453 | 0.2804 | 0.4100 | 0.1819 | 0.1264 | 17.0689 | 0.9434 | 0.1504 |
| BGE-base | MTSamples | keyword | 4 | 0.3333 | 0.0736 | 0.0194 | 0.0438 | 0.1078 | 0.2400 | 0.1056 | 0.0306 | 12.9307 | 0.9493 | 0.2085 |
| BGE-base | MTSamples | keyword | 5 | 0.4167 | 0.0826 | 0.0297 | 0.0477 | 0.1223 | 0.2400 | 0.1124 | 0.0392 | 14.0007 | 0.9439 | 0.1797 |
| BGE-base | MTSamples | keyword | 6 | 0.5000 | 0.0849 | 0.0324 | 0.0492 | 0.1248 | 0.2400 | 0.1062 | 0.0314 | 13.4679 | 0.9370 | 0.1792 |
| BGE-base | MTSamples | keyword | 7 | 0.5833 | 0.1052 | 0.0558 | 0.0658 | 0.1517 | 0.2800 | 0.1222 | 0.0514 | 13.4533 | 0.9270 | 0.1727 |
| BGE-base | MTSamples | keyword | 8 | 0.6667 | 0.0920 | 0.0406 | 0.0529 | 0.1405 | 0.2300 | 0.1046 | 0.0294 | 11.8907 | 0.9215 | 0.1882 |
| BGE-base | MTSamples | keyword | 9 | 0.7500 | 0.0568 | 0.0000 | 0.0290 | 0.0920 | 0.1600 | 0.0812 | 0.0000 | 10.1499 | 0.8996 | 0.2385 |
| BGE-base | MTSamples | keyword | 10 | 0.8333 | 0.0859 | 0.0335 | 0.0484 | 0.1285 | 0.2200 | 0.1439 | 0.0787 | 12.0375 | 0.9215 | 0.1861 |
| BGE-base | MTSamples | keyword | 11 | 0.9167 | 0.8469 | 0.9109 | 0.7795 | 0.8988 | 0.9600 | 0.8081 | 0.9114 | 25.2329 | 0.8032 | 0.1458 |
| BGE-base | MTSamples | keyword | 12 | 1.0000 | 0.9242 | 1.0000 | 0.8750 | 0.9658 | 0.9600 | 0.8788 | 1.0000 | 37.5876 | 0.6716 | 0.0981 |
| BGE-base | MTSamples | natural_language | 0 | 0.0000 | 0.2674 | 0.2360 | 0.2101 | 0.3312 | 0.7300 | 0.3931 | 0.3912 | 21.0157 | 0.8933 | 0.1288 |
| BGE-base | MTSamples | natural_language | 1 | 0.0833 | 0.3333 | 0.3121 | 0.2697 | 0.3984 | 0.7900 | 0.4003 | 0.4002 | 21.3471 | 0.9173 | 0.1206 |
| BGE-base | MTSamples | natural_language | 2 | 0.1667 | 0.2074 | 0.1667 | 0.1464 | 0.2756 | 0.4900 | 0.2979 | 0.2718 | 19.6801 | 0.9417 | 0.1268 |
| BGE-base | MTSamples | natural_language | 3 | 0.2500 | 0.1150 | 0.0600 | 0.0661 | 0.1675 | 0.2400 | 0.1819 | 0.1264 | 17.0689 | 0.9434 | 0.1504 |
| BGE-base | MTSamples | natural_language | 4 | 0.3333 | 0.0633 | 0.0003 | 0.0300 | 0.1041 | 0.1700 | 0.1056 | 0.0306 | 12.9307 | 0.9493 | 0.2085 |
| BGE-base | MTSamples | natural_language | 5 | 0.4167 | 0.0637 | 0.0008 | 0.0294 | 0.1020 | 0.1600 | 0.1124 | 0.0392 | 14.0007 | 0.9439 | 0.1797 |
| BGE-base | MTSamples | natural_language | 6 | 0.5000 | 0.0630 | 0.0000 | 0.0298 | 0.0999 | 0.1700 | 0.1062 | 0.0314 | 13.4679 | 0.9370 | 0.1792 |
| BGE-base | MTSamples | natural_language | 7 | 0.5833 | 0.0692 | 0.0072 | 0.0366 | 0.1091 | 0.2000 | 0.1222 | 0.0514 | 13.4533 | 0.9270 | 0.1727 |
| BGE-base | MTSamples | natural_language | 8 | 0.6667 | 0.0690 | 0.0069 | 0.0331 | 0.1120 | 0.1900 | 0.1046 | 0.0294 | 11.8907 | 0.9215 | 0.1882 |
| BGE-base | MTSamples | natural_language | 9 | 0.7500 | 0.0722 | 0.0106 | 0.0345 | 0.1169 | 0.1800 | 0.0812 | 0.0000 | 10.1499 | 0.8996 | 0.2385 |
| BGE-base | MTSamples | natural_language | 10 | 0.8333 | 0.1032 | 0.0464 | 0.0577 | 0.1528 | 0.2500 | 0.1439 | 0.0787 | 12.0375 | 0.9215 | 0.1861 |
| BGE-base | MTSamples | natural_language | 11 | 0.9167 | 0.8971 | 0.9629 | 0.8367 | 0.9417 | 0.9700 | 0.8081 | 0.9114 | 25.2329 | 0.8032 | 0.1458 |
| BGE-base | MTSamples | natural_language | 12 | 1.0000 | 0.9292 | 1.0000 | 0.8700 | 0.9625 | 0.9600 | 0.8788 | 1.0000 | 37.5876 | 0.6716 | 0.0981 |
| BGE-base | PMC-Patients | keyword | 0 | 0.0000 | 0.4363 | 0.4160 | 0.3587 | 0.5149 | 0.7400 | 0.3931 | 0.3912 | 24.6717 | 0.9042 | 0.1345 |
| BGE-base | PMC-Patients | keyword | 1 | 0.0833 | 0.3460 | 0.3146 | 0.2732 | 0.4274 | 0.6400 | 0.4003 | 0.4002 | 23.5271 | 0.9264 | 0.1332 |
| BGE-base | PMC-Patients | keyword | 2 | 0.1667 | 0.1983 | 0.1487 | 0.1388 | 0.2602 | 0.4400 | 0.2979 | 0.2718 | 19.2997 | 0.9467 | 0.1561 |
| BGE-base | PMC-Patients | keyword | 3 | 0.2500 | 0.1113 | 0.0510 | 0.0652 | 0.1665 | 0.2700 | 0.1819 | 0.1264 | 14.7837 | 0.9478 | 0.2025 |
| BGE-base | PMC-Patients | keyword | 4 | 0.3333 | 0.0659 | 0.0000 | 0.0307 | 0.1119 | 0.1600 | 0.1056 | 0.0306 | 10.2806 | 0.9518 | 0.2712 |
| BGE-base | PMC-Patients | keyword | 5 | 0.4167 | 0.0741 | 0.0092 | 0.0357 | 0.1178 | 0.1900 | 0.1124 | 0.0392 | 11.5281 | 0.9472 | 0.2450 |
| BGE-base | PMC-Patients | keyword | 6 | 0.5000 | 0.0742 | 0.0093 | 0.0364 | 0.1195 | 0.2000 | 0.1062 | 0.0314 | 11.4163 | 0.9443 | 0.2473 |
| BGE-base | PMC-Patients | keyword | 7 | 0.5833 | 0.1002 | 0.0385 | 0.0545 | 0.1507 | 0.2500 | 0.1222 | 0.0514 | 12.5027 | 0.9356 | 0.2280 |
| BGE-base | PMC-Patients | keyword | 8 | 0.6667 | 0.0836 | 0.0199 | 0.0421 | 0.1331 | 0.2300 | 0.1046 | 0.0294 | 13.1282 | 0.9328 | 0.2105 |
| BGE-base | PMC-Patients | keyword | 9 | 0.7500 | 0.0755 | 0.0108 | 0.0351 | 0.1257 | 0.1700 | 0.0812 | 0.0000 | 10.1983 | 0.9147 | 0.2639 |
| BGE-base | PMC-Patients | keyword | 10 | 0.8333 | 0.1104 | 0.0500 | 0.0640 | 0.1647 | 0.2600 | 0.1439 | 0.0787 | 14.2485 | 0.9284 | 0.1993 |
| BGE-base | PMC-Patients | keyword | 11 | 0.9167 | 0.9284 | 0.9688 | 0.8839 | 0.9675 | 0.9800 | 0.8081 | 0.9114 | 36.3325 | 0.8161 | 0.0787 |
| BGE-base | PMC-Patients | keyword | 12 | 1.0000 | 0.9562 | 1.0000 | 0.9204 | 0.9834 | 1.0000 | 0.8788 | 1.0000 | 43.0634 | 0.6676 | 0.0643 |
| BGE-base | PMC-Patients | natural_language | 0 | 0.0000 | 0.1540 | 0.0965 | 0.0985 | 0.2173 | 0.3700 | 0.3931 | 0.3912 | 24.6717 | 0.9042 | 0.1345 |
| BGE-base | PMC-Patients | natural_language | 1 | 0.0833 | 0.1593 | 0.1024 | 0.1047 | 0.2190 | 0.3700 | 0.4003 | 0.4002 | 23.5271 | 0.9264 | 0.1332 |
| BGE-base | PMC-Patients | natural_language | 2 | 0.1667 | 0.1161 | 0.0545 | 0.0692 | 0.1721 | 0.2800 | 0.2979 | 0.2718 | 19.2997 | 0.9467 | 0.1561 |
| BGE-base | PMC-Patients | natural_language | 3 | 0.2500 | 0.0798 | 0.0142 | 0.0406 | 0.1269 | 0.2000 | 0.1819 | 0.1264 | 14.7837 | 0.9478 | 0.2025 |
| BGE-base | PMC-Patients | natural_language | 4 | 0.3333 | 0.0673 | 0.0003 | 0.0302 | 0.1125 | 0.1600 | 0.1056 | 0.0306 | 10.2806 | 0.9518 | 0.2712 |
| BGE-base | PMC-Patients | natural_language | 5 | 0.4167 | 0.0670 | 0.0000 | 0.0300 | 0.1100 | 0.1600 | 0.1124 | 0.0392 | 11.5281 | 0.9472 | 0.2450 |
| BGE-base | PMC-Patients | natural_language | 6 | 0.5000 | 0.0676 | 0.0007 | 0.0312 | 0.1142 | 0.1600 | 0.1062 | 0.0314 | 11.4163 | 0.9443 | 0.2473 |
| BGE-base | PMC-Patients | natural_language | 7 | 0.5833 | 0.0697 | 0.0030 | 0.0303 | 0.1141 | 0.1600 | 0.1222 | 0.0514 | 12.5027 | 0.9356 | 0.2280 |
| BGE-base | PMC-Patients | natural_language | 8 | 0.6667 | 0.0748 | 0.0087 | 0.0324 | 0.1240 | 0.1500 | 0.1046 | 0.0294 | 13.1282 | 0.9328 | 0.2105 |
| BGE-base | PMC-Patients | natural_language | 9 | 0.7500 | 0.0775 | 0.0116 | 0.0352 | 0.1274 | 0.1500 | 0.0812 | 0.0000 | 10.1983 | 0.9147 | 0.2639 |
| BGE-base | PMC-Patients | natural_language | 10 | 0.8333 | 0.1001 | 0.0367 | 0.0564 | 0.1483 | 0.2600 | 0.1439 | 0.0787 | 14.2485 | 0.9284 | 0.1993 |
| BGE-base | PMC-Patients | natural_language | 11 | 0.9167 | 0.9200 | 0.9464 | 0.8750 | 0.9617 | 0.9900 | 0.8081 | 0.9114 | 36.3325 | 0.8161 | 0.0787 |
| BGE-base | PMC-Patients | natural_language | 12 | 1.0000 | 0.9683 | 1.0000 | 0.9367 | 0.9933 | 0.9900 | 0.8788 | 1.0000 | 43.0634 | 0.6676 | 0.0643 |
| BGE-base | Synthetic | keyword | 0 | 0.0000 | 0.5487 | 0.6864 | 0.4714 | 0.6251 | 0.8600 | 0.3931 | 0.3912 | 18.8305 | 0.9522 | 0.1673 |
| BGE-base | Synthetic | keyword | 1 | 0.0833 | 0.5125 | 0.6268 | 0.4394 | 0.5935 | 0.8200 | 0.4003 | 0.4002 | 17.7916 | 0.9594 | 0.1663 |
| BGE-base | Synthetic | keyword | 2 | 0.1667 | 0.4511 | 0.5258 | 0.3665 | 0.5174 | 0.7500 | 0.2979 | 0.2718 | 17.2716 | 0.9726 | 0.1732 |
| BGE-base | Synthetic | keyword | 3 | 0.2500 | 0.3641 | 0.3826 | 0.2922 | 0.4382 | 0.6900 | 0.1819 | 0.1264 | 17.4923 | 0.9753 | 0.1754 |
| BGE-base | Synthetic | keyword | 4 | 0.3333 | 0.2551 | 0.2031 | 0.1936 | 0.3217 | 0.6200 | 0.1056 | 0.0306 | 16.4647 | 0.9802 | 0.1901 |
| BGE-base | Synthetic | keyword | 5 | 0.4167 | 0.2865 | 0.2548 | 0.2188 | 0.3525 | 0.6500 | 0.1124 | 0.0392 | 15.5759 | 0.9779 | 0.1975 |
| BGE-base | Synthetic | keyword | 6 | 0.5000 | 0.2604 | 0.2119 | 0.1982 | 0.3260 | 0.6400 | 0.1062 | 0.0314 | 14.6331 | 0.9760 | 0.2094 |
| BGE-base | Synthetic | keyword | 7 | 0.5833 | 0.2988 | 0.2751 | 0.2308 | 0.3706 | 0.6600 | 0.1222 | 0.0514 | 16.0219 | 0.9716 | 0.1870 |
| BGE-base | Synthetic | keyword | 8 | 0.6667 | 0.2389 | 0.1765 | 0.1793 | 0.3022 | 0.5700 | 0.1046 | 0.0294 | 15.4394 | 0.9709 | 0.1943 |
| BGE-base | Synthetic | keyword | 9 | 0.7500 | 0.1317 | 0.0000 | 0.0879 | 0.1892 | 0.4700 | 0.0812 | 0.0000 | 16.0932 | 0.9689 | 0.1467 |
| BGE-base | Synthetic | keyword | 10 | 0.8333 | 0.2707 | 0.2288 | 0.2064 | 0.3380 | 0.5900 | 0.1439 | 0.0787 | 16.5259 | 0.9718 | 0.1514 |
| BGE-base | Synthetic | keyword | 11 | 0.9167 | 0.6608 | 0.8709 | 0.5701 | 0.7240 | 0.8900 | 0.8081 | 0.9114 | 18.8393 | 0.8845 | 0.1329 |
| BGE-base | Synthetic | keyword | 12 | 1.0000 | 0.7392 | 1.0000 | 0.6593 | 0.7947 | 0.9500 | 0.8788 | 1.0000 | 21.1808 | 0.7546 | 0.1158 |
| BGE-base | Synthetic | natural_language | 0 | 0.0000 | 0.3684 | 0.4360 | 0.2919 | 0.4458 | 0.6100 | 0.3931 | 0.3912 | 18.8305 | 0.9522 | 0.1673 |
| BGE-base | Synthetic | natural_language | 1 | 0.0833 | 0.4583 | 0.5670 | 0.3766 | 0.5435 | 0.7100 | 0.4003 | 0.4002 | 17.7916 | 0.9594 | 0.1663 |
| BGE-base | Synthetic | natural_language | 2 | 0.1667 | 0.3817 | 0.4554 | 0.3052 | 0.4639 | 0.6500 | 0.2979 | 0.2718 | 17.2716 | 0.9726 | 0.1732 |
| BGE-base | Synthetic | natural_language | 3 | 0.2500 | 0.2109 | 0.2066 | 0.1565 | 0.2690 | 0.5900 | 0.1819 | 0.1264 | 17.4923 | 0.9753 | 0.1754 |
| BGE-base | Synthetic | natural_language | 4 | 0.3333 | 0.1083 | 0.0571 | 0.0726 | 0.1441 | 0.4200 | 0.1056 | 0.0306 | 16.4647 | 0.9802 | 0.1901 |
| BGE-base | Synthetic | natural_language | 5 | 0.4167 | 0.1007 | 0.0460 | 0.0667 | 0.1423 | 0.3700 | 0.1124 | 0.0392 | 15.5759 | 0.9779 | 0.1975 |
| BGE-base | Synthetic | natural_language | 6 | 0.5000 | 0.0869 | 0.0259 | 0.0583 | 0.1190 | 0.3500 | 0.1062 | 0.0314 | 14.6331 | 0.9760 | 0.2094 |
| BGE-base | Synthetic | natural_language | 7 | 0.5833 | 0.0898 | 0.0302 | 0.0589 | 0.1248 | 0.3500 | 0.1222 | 0.0514 | 16.0219 | 0.9716 | 0.1870 |
| BGE-base | Synthetic | natural_language | 8 | 0.6667 | 0.0691 | 0.0000 | 0.0407 | 0.1037 | 0.3100 | 0.1046 | 0.0294 | 15.4394 | 0.9709 | 0.1943 |
| BGE-base | Synthetic | natural_language | 9 | 0.7500 | 0.0732 | 0.0060 | 0.0359 | 0.1064 | 0.2400 | 0.0812 | 0.0000 | 16.0932 | 0.9689 | 0.1467 |
| BGE-base | Synthetic | natural_language | 10 | 0.8333 | 0.1931 | 0.1807 | 0.1314 | 0.2600 | 0.4100 | 0.1439 | 0.0787 | 16.5259 | 0.9718 | 0.1514 |
| BGE-base | Synthetic | natural_language | 11 | 0.9167 | 0.5954 | 0.7668 | 0.5047 | 0.6602 | 0.8700 | 0.8081 | 0.9114 | 18.8393 | 0.8845 | 0.1329 |
| BGE-base | Synthetic | natural_language | 12 | 1.0000 | 0.7555 | 1.0000 | 0.6636 | 0.8048 | 0.9100 | 0.8788 | 1.0000 | 21.1808 | 0.7546 | 0.1158 |
| BioBERT | MTSamples | keyword | 0 | 0.0000 | 0.1527 | 0.1308 | 0.1177 | 0.1881 | 0.5200 | 0.1938 | 0.1578 | 14.3177 | 0.8384 | 0.2101 |
| BioBERT | MTSamples | keyword | 1 | 0.0833 | 0.5049 | 1.0000 | 0.4213 | 0.5872 | 0.7600 | 0.3498 | 0.8017 | 17.4248 | 0.9148 | 0.1566 |
| BioBERT | MTSamples | keyword | 2 | 0.1667 | 0.4487 | 0.8613 | 0.3680 | 0.5326 | 0.7300 | 0.3082 | 0.6301 | 14.9228 | 0.9336 | 0.1836 |
| BioBERT | MTSamples | keyword | 3 | 0.2500 | 0.3066 | 0.5106 | 0.2422 | 0.3723 | 0.6800 | 0.2547 | 0.4090 | 14.2958 | 0.9432 | 0.1871 |
| BioBERT | MTSamples | keyword | 4 | 0.3333 | 0.1972 | 0.2406 | 0.1429 | 0.2549 | 0.5700 | 0.2037 | 0.1988 | 14.0173 | 0.9532 | 0.1837 |
| BioBERT | MTSamples | keyword | 5 | 0.4167 | 0.1689 | 0.1708 | 0.1187 | 0.2254 | 0.4600 | 0.2005 | 0.1852 | 13.3971 | 0.9569 | 0.1848 |
| BioBERT | MTSamples | keyword | 6 | 0.5000 | 0.1734 | 0.1819 | 0.1176 | 0.2318 | 0.4500 | 0.1895 | 0.1400 | 13.2224 | 0.9569 | 0.1897 |
| BioBERT | MTSamples | keyword | 7 | 0.5833 | 0.1398 | 0.0990 | 0.0939 | 0.1936 | 0.4400 | 0.1697 | 0.0581 | 12.8298 | 0.9471 | 0.1946 |
| BioBERT | MTSamples | keyword | 8 | 0.6667 | 0.1357 | 0.0888 | 0.0837 | 0.1927 | 0.3400 | 0.1625 | 0.0284 | 12.9524 | 0.9430 | 0.1922 |
| BioBERT | MTSamples | keyword | 9 | 0.7500 | 0.0997 | 0.0000 | 0.0630 | 0.1440 | 0.3000 | 0.1556 | 0.0000 | 12.4238 | 0.9438 | 0.2028 |
| BioBERT | MTSamples | keyword | 10 | 0.8333 | 0.1209 | 0.0523 | 0.0747 | 0.1685 | 0.3400 | 0.1738 | 0.0750 | 12.7476 | 0.9382 | 0.2063 |
| BioBERT | MTSamples | keyword | 11 | 0.9167 | 0.2395 | 0.3450 | 0.1808 | 0.3032 | 0.5500 | 0.2491 | 0.3861 | 13.0363 | 0.9602 | 0.2098 |
| BioBERT | MTSamples | keyword | 12 | 1.0000 | 0.4290 | 0.8127 | 0.3442 | 0.5091 | 0.6800 | 0.3978 | 1.0000 | 16.7103 | 0.9511 | 0.1803 |
| BioBERT | MTSamples | natural_language | 0 | 0.0000 | 0.1034 | 0.1099 | 0.0718 | 0.1392 | 0.4000 | 0.1938 | 0.1578 | 14.3177 | 0.8384 | 0.2101 |
| BioBERT | MTSamples | natural_language | 1 | 0.0833 | 0.4136 | 1.0000 | 0.3438 | 0.4841 | 0.8000 | 0.3498 | 0.8017 | 17.4248 | 0.9148 | 0.1566 |
| BioBERT | MTSamples | natural_language | 2 | 0.1667 | 0.2769 | 0.6077 | 0.2106 | 0.3329 | 0.7300 | 0.3082 | 0.6301 | 14.9228 | 0.9336 | 0.1836 |
| BioBERT | MTSamples | natural_language | 3 | 0.2500 | 0.2107 | 0.4178 | 0.1566 | 0.2692 | 0.5900 | 0.2547 | 0.4090 | 14.2958 | 0.9432 | 0.1871 |
| BioBERT | MTSamples | natural_language | 4 | 0.3333 | 0.1364 | 0.2046 | 0.0927 | 0.1874 | 0.4100 | 0.2037 | 0.1988 | 14.0173 | 0.9532 | 0.1837 |
| BioBERT | MTSamples | natural_language | 5 | 0.4167 | 0.1142 | 0.1409 | 0.0701 | 0.1620 | 0.3000 | 0.2005 | 0.1852 | 13.3971 | 0.9569 | 0.1848 |
| BioBERT | MTSamples | natural_language | 6 | 0.5000 | 0.1077 | 0.1222 | 0.0675 | 0.1502 | 0.3400 | 0.1895 | 0.1400 | 13.2224 | 0.9569 | 0.1897 |
| BioBERT | MTSamples | natural_language | 7 | 0.5833 | 0.0933 | 0.0809 | 0.0519 | 0.1401 | 0.2500 | 0.1697 | 0.0581 | 12.8298 | 0.9471 | 0.1946 |
| BioBERT | MTSamples | natural_language | 8 | 0.6667 | 0.0823 | 0.0494 | 0.0488 | 0.1257 | 0.2400 | 0.1625 | 0.0284 | 12.9524 | 0.9430 | 0.1922 |
| BioBERT | MTSamples | natural_language | 9 | 0.7500 | 0.0651 | 0.0000 | 0.0384 | 0.0980 | 0.2200 | 0.1556 | 0.0000 | 12.4238 | 0.9438 | 0.2028 |
| BioBERT | MTSamples | natural_language | 10 | 0.8333 | 0.1041 | 0.1119 | 0.0624 | 0.1528 | 0.2800 | 0.1738 | 0.0750 | 12.7476 | 0.9382 | 0.2063 |
| BioBERT | MTSamples | natural_language | 11 | 0.9167 | 0.1435 | 0.2250 | 0.0985 | 0.1919 | 0.4000 | 0.2491 | 0.3861 | 13.0363 | 0.9602 | 0.2098 |
| BioBERT | MTSamples | natural_language | 12 | 1.0000 | 0.3439 | 0.8000 | 0.2747 | 0.4190 | 0.6700 | 0.3978 | 1.0000 | 16.7103 | 0.9511 | 0.1803 |
| BioBERT | PMC-Patients | keyword | 0 | 0.0000 | 0.0901 | 0.0000 | 0.0575 | 0.1259 | 0.3300 | 0.1938 | 0.1578 | 10.3402 | 0.8289 | 0.2819 |
| BioBERT | PMC-Patients | keyword | 1 | 0.0833 | 0.3075 | 0.6556 | 0.2463 | 0.3809 | 0.7000 | 0.3498 | 0.8017 | 20.1855 | 0.9139 | 0.1429 |
| BioBERT | PMC-Patients | keyword | 2 | 0.1667 | 0.2864 | 0.5920 | 0.2180 | 0.3556 | 0.6400 | 0.3082 | 0.6301 | 19.5822 | 0.9368 | 0.1423 |
| BioBERT | PMC-Patients | keyword | 3 | 0.2500 | 0.2155 | 0.3782 | 0.1569 | 0.2793 | 0.5300 | 0.2547 | 0.4090 | 19.1987 | 0.9469 | 0.1420 |
| BioBERT | PMC-Patients | keyword | 4 | 0.3333 | 0.1543 | 0.1936 | 0.1082 | 0.2124 | 0.4200 | 0.2037 | 0.1988 | 19.1330 | 0.9581 | 0.1414 |
| BioBERT | PMC-Patients | keyword | 5 | 0.4167 | 0.1647 | 0.2250 | 0.1129 | 0.2242 | 0.4000 | 0.2005 | 0.1852 | 19.0559 | 0.9606 | 0.1458 |
| BioBERT | PMC-Patients | keyword | 6 | 0.5000 | 0.1724 | 0.2482 | 0.1178 | 0.2312 | 0.4500 | 0.1895 | 0.1400 | 18.4048 | 0.9606 | 0.1570 |
| BioBERT | PMC-Patients | keyword | 7 | 0.5833 | 0.1838 | 0.2826 | 0.1220 | 0.2473 | 0.4000 | 0.1697 | 0.0581 | 18.1137 | 0.9520 | 0.1589 |
| BioBERT | PMC-Patients | keyword | 8 | 0.6667 | 0.1779 | 0.2648 | 0.1192 | 0.2459 | 0.3500 | 0.1625 | 0.0284 | 17.6536 | 0.9475 | 0.1634 |
| BioBERT | PMC-Patients | keyword | 9 | 0.7500 | 0.1892 | 0.2989 | 0.1262 | 0.2583 | 0.3700 | 0.1556 | 0.0000 | 17.2900 | 0.9438 | 0.1611 |
| BioBERT | PMC-Patients | keyword | 10 | 0.8333 | 0.2588 | 0.5087 | 0.1906 | 0.3299 | 0.4400 | 0.1738 | 0.0750 | 18.7663 | 0.9372 | 0.1474 |
| BioBERT | PMC-Patients | keyword | 11 | 0.9167 | 0.3678 | 0.8375 | 0.2884 | 0.4500 | 0.6500 | 0.2491 | 0.3861 | 20.3615 | 0.9592 | 0.1412 |
| BioBERT | PMC-Patients | keyword | 12 | 1.0000 | 0.4217 | 1.0000 | 0.3439 | 0.4999 | 0.7300 | 0.3978 | 1.0000 | 24.5709 | 0.9473 | 0.1224 |
| BioBERT | PMC-Patients | natural_language | 0 | 0.0000 | 0.0953 | 0.0000 | 0.0527 | 0.1474 | 0.3100 | 0.1938 | 0.1578 | 10.3402 | 0.8289 | 0.2819 |
| BioBERT | PMC-Patients | natural_language | 1 | 0.0833 | 0.2540 | 0.4536 | 0.1958 | 0.3199 | 0.6100 | 0.3498 | 0.8017 | 20.1855 | 0.9139 | 0.1429 |
| BioBERT | PMC-Patients | natural_language | 2 | 0.1667 | 0.2168 | 0.3472 | 0.1599 | 0.2798 | 0.5100 | 0.3082 | 0.6301 | 19.5822 | 0.9368 | 0.1423 |
| BioBERT | PMC-Patients | natural_language | 3 | 0.2500 | 0.1600 | 0.1849 | 0.1104 | 0.2168 | 0.4200 | 0.2547 | 0.4090 | 19.1987 | 0.9469 | 0.1420 |
| BioBERT | PMC-Patients | natural_language | 4 | 0.3333 | 0.1347 | 0.1126 | 0.0824 | 0.1908 | 0.3400 | 0.2037 | 0.1988 | 19.1330 | 0.9581 | 0.1414 |
| BioBERT | PMC-Patients | natural_language | 5 | 0.4167 | 0.1269 | 0.0903 | 0.0801 | 0.1813 | 0.3200 | 0.2005 | 0.1852 | 19.0559 | 0.9606 | 0.1458 |
| BioBERT | PMC-Patients | natural_language | 6 | 0.5000 | 0.1359 | 0.1160 | 0.0859 | 0.1946 | 0.3000 | 0.1895 | 0.1400 | 18.4048 | 0.9606 | 0.1570 |
| BioBERT | PMC-Patients | natural_language | 7 | 0.5833 | 0.1193 | 0.0686 | 0.0687 | 0.1726 | 0.2900 | 0.1697 | 0.0581 | 18.1137 | 0.9520 | 0.1589 |
| BioBERT | PMC-Patients | natural_language | 8 | 0.6667 | 0.1051 | 0.0280 | 0.0611 | 0.1610 | 0.2700 | 0.1625 | 0.0284 | 17.6536 | 0.9475 | 0.1634 |
| BioBERT | PMC-Patients | natural_language | 9 | 0.7500 | 0.0994 | 0.0117 | 0.0554 | 0.1502 | 0.2600 | 0.1556 | 0.0000 | 17.2900 | 0.9438 | 0.1611 |
| BioBERT | PMC-Patients | natural_language | 10 | 0.8333 | 0.1453 | 0.1429 | 0.0960 | 0.2006 | 0.3700 | 0.1738 | 0.0750 | 18.7663 | 0.9372 | 0.1474 |
| BioBERT | PMC-Patients | natural_language | 11 | 0.9167 | 0.2306 | 0.3867 | 0.1652 | 0.3062 | 0.4900 | 0.2491 | 0.3861 | 20.3615 | 0.9592 | 0.1412 |
| BioBERT | PMC-Patients | natural_language | 12 | 1.0000 | 0.4452 | 1.0000 | 0.3648 | 0.5253 | 0.7200 | 0.3978 | 1.0000 | 24.5709 | 0.9473 | 0.1224 |
| BioBERT | Synthetic | keyword | 0 | 0.0000 | 0.4070 | 0.7423 | 0.3301 | 0.4810 | 0.7400 | 0.1938 | 0.1578 | 17.0433 | 0.9343 | 0.1759 |
| BioBERT | Synthetic | keyword | 1 | 0.0833 | 0.3168 | 0.2188 | 0.2498 | 0.3865 | 0.6600 | 0.3498 | 0.8017 | 14.6254 | 0.9562 | 0.1842 |
| BioBERT | Synthetic | keyword | 2 | 0.1667 | 0.3397 | 0.3517 | 0.2695 | 0.4177 | 0.7000 | 0.3082 | 0.6301 | 14.1119 | 0.9678 | 0.1928 |
| BioBERT | Synthetic | keyword | 3 | 0.2500 | 0.3573 | 0.4539 | 0.2860 | 0.4315 | 0.6900 | 0.2547 | 0.4090 | 14.1204 | 0.9744 | 0.1999 |
| BioBERT | Synthetic | keyword | 4 | 0.3333 | 0.3489 | 0.4051 | 0.2762 | 0.4259 | 0.6500 | 0.2037 | 0.1988 | 13.8021 | 0.9801 | 0.2104 |
| BioBERT | Synthetic | keyword | 5 | 0.4167 | 0.3936 | 0.6645 | 0.3178 | 0.4709 | 0.6900 | 0.2005 | 0.1852 | 12.7021 | 0.9821 | 0.2271 |
| BioBERT | Synthetic | keyword | 6 | 0.5000 | 0.3645 | 0.4956 | 0.2799 | 0.4425 | 0.6200 | 0.1895 | 0.1400 | 11.5703 | 0.9819 | 0.2462 |
| BioBERT | Synthetic | keyword | 7 | 0.5833 | 0.3173 | 0.2217 | 0.2435 | 0.3987 | 0.5900 | 0.1697 | 0.0581 | 10.7548 | 0.9771 | 0.2612 |
| BioBERT | Synthetic | keyword | 8 | 0.6667 | 0.3029 | 0.1381 | 0.2297 | 0.3772 | 0.5600 | 0.1625 | 0.0284 | 10.4059 | 0.9749 | 0.2671 |
| BioBERT | Synthetic | keyword | 9 | 0.7500 | 0.3174 | 0.2223 | 0.2462 | 0.3941 | 0.6200 | 0.1556 | 0.0000 | 11.0774 | 0.9746 | 0.2524 |
| BioBERT | Synthetic | keyword | 10 | 0.8333 | 0.2791 | 0.0000 | 0.2102 | 0.3546 | 0.5600 | 0.1738 | 0.0750 | 11.4215 | 0.9720 | 0.2467 |
| BioBERT | Synthetic | keyword | 11 | 0.9167 | 0.3409 | 0.3587 | 0.2642 | 0.4216 | 0.5800 | 0.2491 | 0.3861 | 11.8906 | 0.9819 | 0.2389 |
| BioBERT | Synthetic | keyword | 12 | 1.0000 | 0.4514 | 1.0000 | 0.3705 | 0.5298 | 0.7400 | 0.3978 | 1.0000 | 15.1725 | 0.9764 | 0.1897 |
| BioBERT | Synthetic | natural_language | 0 | 0.0000 | 0.3145 | 1.0000 | 0.2430 | 0.3905 | 0.6100 | 0.1938 | 0.1578 | 17.0433 | 0.9343 | 0.1759 |
| BioBERT | Synthetic | natural_language | 1 | 0.0833 | 0.3017 | 0.9289 | 0.2311 | 0.3725 | 0.5900 | 0.3498 | 0.8017 | 14.6254 | 0.9562 | 0.1842 |
| BioBERT | Synthetic | natural_language | 2 | 0.1667 | 0.2806 | 0.8117 | 0.2128 | 0.3506 | 0.5500 | 0.3082 | 0.6301 | 14.1119 | 0.9678 | 0.1928 |
| BioBERT | Synthetic | natural_language | 3 | 0.2500 | 0.2779 | 0.7967 | 0.2110 | 0.3499 | 0.5500 | 0.2547 | 0.4090 | 14.1204 | 0.9744 | 0.1999 |
| BioBERT | Synthetic | natural_language | 4 | 0.3333 | 0.2510 | 0.6472 | 0.1799 | 0.3260 | 0.4600 | 0.2037 | 0.1988 | 13.8021 | 0.9801 | 0.2104 |
| BioBERT | Synthetic | natural_language | 5 | 0.4167 | 0.2345 | 0.5556 | 0.1665 | 0.3120 | 0.4500 | 0.2005 | 0.1852 | 12.7021 | 0.9821 | 0.2271 |
| BioBERT | Synthetic | natural_language | 6 | 0.5000 | 0.1832 | 0.2706 | 0.1248 | 0.2456 | 0.4200 | 0.1895 | 0.1400 | 11.5703 | 0.9819 | 0.2462 |
| BioBERT | Synthetic | natural_language | 7 | 0.5833 | 0.1646 | 0.1672 | 0.1070 | 0.2299 | 0.3600 | 0.1697 | 0.0581 | 10.7548 | 0.9771 | 0.2612 |
| BioBERT | Synthetic | natural_language | 8 | 0.6667 | 0.1711 | 0.2033 | 0.1104 | 0.2453 | 0.3100 | 0.1625 | 0.0284 | 10.4059 | 0.9749 | 0.2671 |
| BioBERT | Synthetic | natural_language | 9 | 0.7500 | 0.1629 | 0.1578 | 0.1008 | 0.2253 | 0.3400 | 0.1556 | 0.0000 | 11.0774 | 0.9746 | 0.2524 |
| BioBERT | Synthetic | natural_language | 10 | 0.8333 | 0.1345 | 0.0000 | 0.0846 | 0.1871 | 0.3400 | 0.1738 | 0.0750 | 11.4215 | 0.9720 | 0.2467 |
| BioBERT | Synthetic | natural_language | 11 | 0.9167 | 0.1724 | 0.2106 | 0.1182 | 0.2334 | 0.4100 | 0.2491 | 0.3861 | 11.8906 | 0.9819 | 0.2389 |
| BioBERT | Synthetic | natural_language | 12 | 1.0000 | 0.2954 | 0.8939 | 0.2232 | 0.3643 | 0.5200 | 0.3978 | 1.0000 | 15.1725 | 0.9764 | 0.1897 |
| BioLORD-2023 | MTSamples | keyword | 0 | 0.0000 | 0.7093 | 0.7068 | 0.6291 | 0.7946 | 0.8700 | 0.6021 | 0.4759 | 27.3953 | 0.6615 | 0.1123 |
| BioLORD-2023 | MTSamples | keyword | 1 | 0.0833 | 0.6863 | 0.6470 | 0.6055 | 0.7713 | 0.8300 | 0.5674 | 0.3883 | 23.8240 | 0.8036 | 0.1268 |
| BioLORD-2023 | MTSamples | keyword | 2 | 0.1667 | 0.5243 | 0.2252 | 0.4388 | 0.6033 | 0.7300 | 0.4857 | 0.1817 | 17.7908 | 0.8005 | 0.1594 |
| BioLORD-2023 | MTSamples | keyword | 3 | 0.2500 | 0.4378 | 0.0000 | 0.3578 | 0.5223 | 0.7200 | 0.4623 | 0.1226 | 15.0404 | 0.7949 | 0.1858 |
| BioLORD-2023 | MTSamples | keyword | 4 | 0.3333 | 0.4771 | 0.1023 | 0.3972 | 0.5592 | 0.7600 | 0.4617 | 0.1212 | 12.9832 | 0.8058 | 0.2130 |
| BioLORD-2023 | MTSamples | keyword | 5 | 0.4167 | 0.4978 | 0.1562 | 0.4141 | 0.5771 | 0.7800 | 0.4426 | 0.0730 | 11.2079 | 0.7918 | 0.2442 |
| BioLORD-2023 | MTSamples | keyword | 6 | 0.5000 | 0.4695 | 0.0825 | 0.3891 | 0.5485 | 0.7600 | 0.4144 | 0.0016 | 11.4763 | 0.8111 | 0.2420 |
| BioLORD-2023 | MTSamples | keyword | 7 | 0.5833 | 0.4869 | 0.1278 | 0.4090 | 0.5642 | 0.8000 | 0.4137 | 0.0000 | 10.6917 | 0.8244 | 0.2587 |
| BioLORD-2023 | MTSamples | keyword | 8 | 0.6667 | 0.5785 | 0.3663 | 0.4961 | 0.6506 | 0.8800 | 0.4817 | 0.1718 | 11.2126 | 0.8012 | 0.2514 |
| BioLORD-2023 | MTSamples | keyword | 9 | 0.7500 | 0.6182 | 0.4697 | 0.5364 | 0.6940 | 0.8600 | 0.5582 | 0.3650 | 12.0922 | 0.7734 | 0.2379 |
| BioLORD-2023 | MTSamples | keyword | 10 | 0.8333 | 0.7638 | 0.8487 | 0.6877 | 0.8223 | 0.9600 | 0.7199 | 0.7733 | 17.5876 | 0.7149 | 0.1791 |
| BioLORD-2023 | MTSamples | keyword | 11 | 0.9167 | 0.8219 | 1.0000 | 0.7597 | 0.8818 | 0.9600 | 0.8096 | 1.0000 | 24.6948 | 0.5533 | 0.1238 |
| BioLORD-2023 | MTSamples | keyword | 12 | 1.0000 | 0.8156 | 0.9836 | 0.7493 | 0.8718 | 0.9600 | 0.8037 | 0.9851 | 29.7263 | 0.2413 | 0.1006 |
| BioLORD-2023 | MTSamples | natural_language | 0 | 0.0000 | 0.6505 | 0.5286 | 0.5726 | 0.7277 | 0.8700 | 0.6021 | 0.4759 | 27.3953 | 0.6615 | 0.1123 |
| BioLORD-2023 | MTSamples | natural_language | 1 | 0.0833 | 0.6397 | 0.5069 | 0.5603 | 0.7148 | 0.8700 | 0.5674 | 0.3883 | 23.8240 | 0.8036 | 0.1268 |
| BioLORD-2023 | MTSamples | natural_language | 2 | 0.1667 | 0.5817 | 0.3907 | 0.5029 | 0.6600 | 0.8300 | 0.4857 | 0.1817 | 17.7908 | 0.8005 | 0.1594 |
| BioLORD-2023 | MTSamples | natural_language | 3 | 0.2500 | 0.5549 | 0.3370 | 0.4723 | 0.6354 | 0.8200 | 0.4623 | 0.1226 | 15.0404 | 0.7949 | 0.1858 |
| BioLORD-2023 | MTSamples | natural_language | 4 | 0.3333 | 0.5153 | 0.2577 | 0.4339 | 0.5883 | 0.8300 | 0.4617 | 0.1212 | 12.9832 | 0.8058 | 0.2130 |
| BioLORD-2023 | MTSamples | natural_language | 5 | 0.4167 | 0.4441 | 0.1150 | 0.3665 | 0.5177 | 0.7900 | 0.4426 | 0.0730 | 11.2079 | 0.7918 | 0.2442 |
| BioLORD-2023 | MTSamples | natural_language | 6 | 0.5000 | 0.3867 | 0.0000 | 0.3139 | 0.4579 | 0.7600 | 0.4144 | 0.0016 | 11.4763 | 0.8111 | 0.2420 |
| BioLORD-2023 | MTSamples | natural_language | 7 | 0.5833 | 0.4230 | 0.0727 | 0.3512 | 0.5009 | 0.8100 | 0.4137 | 0.0000 | 10.6917 | 0.8244 | 0.2587 |
| BioLORD-2023 | MTSamples | natural_language | 8 | 0.6667 | 0.5520 | 0.3312 | 0.4717 | 0.6258 | 0.8700 | 0.4817 | 0.1718 | 11.2126 | 0.8012 | 0.2514 |
| BioLORD-2023 | MTSamples | natural_language | 9 | 0.7500 | 0.6361 | 0.4997 | 0.5526 | 0.7055 | 0.8900 | 0.5582 | 0.3650 | 12.0922 | 0.7734 | 0.2379 |
| BioLORD-2023 | MTSamples | natural_language | 10 | 0.8333 | 0.8107 | 0.8495 | 0.7461 | 0.8713 | 0.9500 | 0.7199 | 0.7733 | 17.5876 | 0.7149 | 0.1791 |
| BioLORD-2023 | MTSamples | natural_language | 11 | 0.9167 | 0.8824 | 0.9932 | 0.8179 | 0.9300 | 0.9500 | 0.8096 | 1.0000 | 24.6948 | 0.5533 | 0.1238 |
| BioLORD-2023 | MTSamples | natural_language | 12 | 1.0000 | 0.8858 | 1.0000 | 0.8210 | 0.9360 | 0.9600 | 0.8037 | 0.9851 | 29.7263 | 0.2413 | 0.1006 |
| BioLORD-2023 | PMC-Patients | keyword | 0 | 0.0000 | 0.5294 | 0.2286 | 0.4385 | 0.6164 | 0.7200 | 0.6021 | 0.4759 | 30.6093 | 0.7033 | 0.1142 |
| BioLORD-2023 | PMC-Patients | keyword | 1 | 0.0833 | 0.4913 | 0.1508 | 0.3987 | 0.5757 | 0.6700 | 0.5674 | 0.3883 | 25.6188 | 0.8312 | 0.1297 |
| BioLORD-2023 | PMC-Patients | keyword | 2 | 0.1667 | 0.4315 | 0.0286 | 0.3504 | 0.5135 | 0.6500 | 0.4857 | 0.1817 | 19.9706 | 0.8187 | 0.1544 |
| BioLORD-2023 | PMC-Patients | keyword | 3 | 0.2500 | 0.4175 | 0.0000 | 0.3400 | 0.4951 | 0.6800 | 0.4623 | 0.1226 | 18.6022 | 0.8133 | 0.1561 |
| BioLORD-2023 | PMC-Patients | keyword | 4 | 0.3333 | 0.4462 | 0.0586 | 0.3632 | 0.5223 | 0.7300 | 0.4617 | 0.1212 | 18.2069 | 0.8330 | 0.1594 |
| BioLORD-2023 | PMC-Patients | keyword | 5 | 0.4167 | 0.4558 | 0.0783 | 0.3745 | 0.5393 | 0.7100 | 0.4426 | 0.0730 | 19.4917 | 0.8293 | 0.1351 |
| BioLORD-2023 | PMC-Patients | keyword | 6 | 0.5000 | 0.4712 | 0.1097 | 0.3911 | 0.5562 | 0.7200 | 0.4144 | 0.0016 | 20.5551 | 0.8477 | 0.1295 |
| BioLORD-2023 | PMC-Patients | keyword | 7 | 0.5833 | 0.4796 | 0.1269 | 0.3989 | 0.5619 | 0.7600 | 0.4137 | 0.0000 | 20.4374 | 0.8671 | 0.1359 |
| BioLORD-2023 | PMC-Patients | keyword | 8 | 0.6667 | 0.5599 | 0.2910 | 0.4821 | 0.6416 | 0.8500 | 0.4817 | 0.1718 | 22.6139 | 0.8494 | 0.1253 |
| BioLORD-2023 | PMC-Patients | keyword | 9 | 0.7500 | 0.6358 | 0.4461 | 0.5576 | 0.7115 | 0.9100 | 0.5582 | 0.3650 | 22.5273 | 0.8262 | 0.1156 |
| BioLORD-2023 | PMC-Patients | keyword | 10 | 0.8333 | 0.7870 | 0.7550 | 0.7207 | 0.8507 | 0.9700 | 0.7199 | 0.7733 | 23.2311 | 0.7481 | 0.1208 |
| BioLORD-2023 | PMC-Patients | keyword | 11 | 0.9167 | 0.9069 | 1.0000 | 0.8592 | 0.9484 | 0.9900 | 0.8096 | 1.0000 | 28.5821 | 0.5850 | 0.0862 |
| BioLORD-2023 | PMC-Patients | keyword | 12 | 1.0000 | 0.8969 | 0.9796 | 0.8472 | 0.9417 | 0.9900 | 0.8037 | 0.9851 | 33.1911 | 0.2607 | 0.0814 |
| BioLORD-2023 | PMC-Patients | natural_language | 0 | 0.0000 | 0.5869 | 0.3961 | 0.5061 | 0.6734 | 0.8100 | 0.6021 | 0.4759 | 30.6093 | 0.7033 | 0.1142 |
| BioLORD-2023 | PMC-Patients | natural_language | 1 | 0.0833 | 0.5578 | 0.3515 | 0.4709 | 0.6432 | 0.7600 | 0.5674 | 0.3883 | 25.6188 | 0.8312 | 0.1297 |
| BioLORD-2023 | PMC-Patients | natural_language | 2 | 0.1667 | 0.4783 | 0.2299 | 0.3983 | 0.5676 | 0.7400 | 0.4857 | 0.1817 | 19.9706 | 0.8187 | 0.1544 |
| BioLORD-2023 | PMC-Patients | natural_language | 3 | 0.2500 | 0.4781 | 0.2296 | 0.3929 | 0.5630 | 0.7200 | 0.4623 | 0.1226 | 18.6022 | 0.8133 | 0.1561 |
| BioLORD-2023 | PMC-Patients | natural_language | 4 | 0.3333 | 0.4723 | 0.2207 | 0.3920 | 0.5515 | 0.7400 | 0.4617 | 0.1212 | 18.2069 | 0.8330 | 0.1594 |
| BioLORD-2023 | PMC-Patients | natural_language | 5 | 0.4167 | 0.4151 | 0.1332 | 0.3348 | 0.4947 | 0.6600 | 0.4426 | 0.0730 | 19.4917 | 0.8293 | 0.1351 |
| BioLORD-2023 | PMC-Patients | natural_language | 6 | 0.5000 | 0.3719 | 0.0672 | 0.2941 | 0.4524 | 0.6500 | 0.4144 | 0.0016 | 20.5551 | 0.8477 | 0.1295 |
| BioLORD-2023 | PMC-Patients | natural_language | 7 | 0.5833 | 0.3280 | 0.0000 | 0.2501 | 0.4084 | 0.6100 | 0.4137 | 0.0000 | 20.4374 | 0.8671 | 0.1359 |
| BioLORD-2023 | PMC-Patients | natural_language | 8 | 0.6667 | 0.4020 | 0.1132 | 0.3269 | 0.4793 | 0.7200 | 0.4817 | 0.1718 | 22.6139 | 0.8494 | 0.1253 |
| BioLORD-2023 | PMC-Patients | natural_language | 9 | 0.7500 | 0.5548 | 0.3469 | 0.4814 | 0.6273 | 0.8800 | 0.5582 | 0.3650 | 22.5273 | 0.8262 | 0.1156 |
| BioLORD-2023 | PMC-Patients | natural_language | 10 | 0.8333 | 0.8129 | 0.7418 | 0.7477 | 0.8751 | 0.9700 | 0.7199 | 0.7733 | 23.2311 | 0.7481 | 0.1208 |
| BioLORD-2023 | PMC-Patients | natural_language | 11 | 0.9167 | 0.9598 | 0.9665 | 0.9279 | 0.9850 | 1.0000 | 0.8096 | 1.0000 | 28.5821 | 0.5850 | 0.0862 |
| BioLORD-2023 | PMC-Patients | natural_language | 12 | 1.0000 | 0.9817 | 1.0000 | 0.9583 | 1.0000 | 1.0000 | 0.8037 | 0.9851 | 33.1911 | 0.2607 | 0.0814 |
| BioLORD-2023 | Synthetic | keyword | 0 | 0.0000 | 0.5552 | 0.6708 | 0.4858 | 0.6247 | 0.9100 | 0.6021 | 0.4759 | 19.3752 | 0.8513 | 0.1561 |
| BioLORD-2023 | Synthetic | keyword | 1 | 0.0833 | 0.5078 | 0.4382 | 0.4238 | 0.5790 | 0.8000 | 0.5674 | 0.3883 | 17.4464 | 0.9079 | 0.1631 |
| BioLORD-2023 | Synthetic | keyword | 2 | 0.1667 | 0.4185 | 0.0000 | 0.3474 | 0.4836 | 0.8000 | 0.4857 | 0.1817 | 14.2482 | 0.8998 | 0.1921 |
| BioLORD-2023 | Synthetic | keyword | 3 | 0.2500 | 0.4389 | 0.1001 | 0.3556 | 0.5069 | 0.7700 | 0.4623 | 0.1226 | 13.1480 | 0.9004 | 0.2064 |
| BioLORD-2023 | Synthetic | keyword | 4 | 0.3333 | 0.4495 | 0.1521 | 0.3711 | 0.5282 | 0.7700 | 0.4617 | 0.1212 | 13.0952 | 0.9127 | 0.2072 |
| BioLORD-2023 | Synthetic | keyword | 5 | 0.4167 | 0.4705 | 0.2552 | 0.3897 | 0.5508 | 0.7500 | 0.4426 | 0.0730 | 13.5461 | 0.9139 | 0.1995 |
| BioLORD-2023 | Synthetic | keyword | 6 | 0.5000 | 0.4330 | 0.0711 | 0.3588 | 0.5123 | 0.7300 | 0.4144 | 0.0016 | 13.0329 | 0.9223 | 0.2145 |
| BioLORD-2023 | Synthetic | keyword | 7 | 0.5833 | 0.4466 | 0.1379 | 0.3710 | 0.5265 | 0.7700 | 0.4137 | 0.0000 | 12.8178 | 0.9297 | 0.2200 |
| BioLORD-2023 | Synthetic | keyword | 8 | 0.6667 | 0.5044 | 0.4215 | 0.4186 | 0.5743 | 0.8200 | 0.4817 | 0.1718 | 14.2286 | 0.9190 | 0.1965 |
| BioLORD-2023 | Synthetic | keyword | 9 | 0.7500 | 0.5082 | 0.4401 | 0.4322 | 0.5969 | 0.8500 | 0.5582 | 0.3650 | 14.8954 | 0.9036 | 0.1840 |
| BioLORD-2023 | Synthetic | keyword | 10 | 0.8333 | 0.5773 | 0.7792 | 0.4953 | 0.6457 | 0.9100 | 0.7199 | 0.7733 | 16.9662 | 0.8286 | 0.1354 |
| BioLORD-2023 | Synthetic | keyword | 11 | 0.9167 | 0.6223 | 1.0000 | 0.5381 | 0.6898 | 0.9200 | 0.8096 | 1.0000 | 17.8686 | 0.6906 | 0.1202 |
| BioLORD-2023 | Synthetic | keyword | 12 | 1.0000 | 0.5836 | 0.8101 | 0.4874 | 0.6425 | 0.8500 | 0.8037 | 0.9851 | 20.3621 | 0.3979 | 0.1111 |
| BioLORD-2023 | Synthetic | natural_language | 0 | 0.0000 | 0.5814 | 0.7764 | 0.5003 | 0.6606 | 0.8200 | 0.6021 | 0.4759 | 19.3752 | 0.8513 | 0.1561 |
| BioLORD-2023 | Synthetic | natural_language | 1 | 0.0833 | 0.5218 | 0.6157 | 0.4356 | 0.5968 | 0.7800 | 0.5674 | 0.3883 | 17.4464 | 0.9079 | 0.1631 |
| BioLORD-2023 | Synthetic | natural_language | 2 | 0.1667 | 0.4797 | 0.5022 | 0.4091 | 0.5589 | 0.7800 | 0.4857 | 0.1817 | 14.2482 | 0.8998 | 0.1921 |
| BioLORD-2023 | Synthetic | natural_language | 3 | 0.2500 | 0.4464 | 0.4124 | 0.3630 | 0.5284 | 0.7500 | 0.4623 | 0.1226 | 13.1480 | 0.9004 | 0.2064 |
| BioLORD-2023 | Synthetic | natural_language | 4 | 0.3333 | 0.4099 | 0.3139 | 0.3309 | 0.4884 | 0.7000 | 0.4617 | 0.1212 | 13.0952 | 0.9127 | 0.2072 |
| BioLORD-2023 | Synthetic | natural_language | 5 | 0.4167 | 0.3724 | 0.2128 | 0.2988 | 0.4484 | 0.7200 | 0.4426 | 0.0730 | 13.5461 | 0.9139 | 0.1995 |
| BioLORD-2023 | Synthetic | natural_language | 6 | 0.5000 | 0.3539 | 0.1629 | 0.2868 | 0.4252 | 0.7000 | 0.4144 | 0.0016 | 13.0329 | 0.9223 | 0.2145 |
| BioLORD-2023 | Synthetic | natural_language | 7 | 0.5833 | 0.3182 | 0.0666 | 0.2579 | 0.3839 | 0.7000 | 0.4137 | 0.0000 | 12.8178 | 0.9297 | 0.2200 |
| BioLORD-2023 | Synthetic | natural_language | 8 | 0.6667 | 0.2935 | 0.0000 | 0.2280 | 0.3610 | 0.6700 | 0.4817 | 0.1718 | 14.2286 | 0.9190 | 0.1965 |
| BioLORD-2023 | Synthetic | natural_language | 9 | 0.7500 | 0.3962 | 0.2770 | 0.3240 | 0.4717 | 0.7300 | 0.5582 | 0.3650 | 14.8954 | 0.9036 | 0.1840 |
| BioLORD-2023 | Synthetic | natural_language | 10 | 0.8333 | 0.5675 | 0.7389 | 0.4840 | 0.6377 | 0.8700 | 0.7199 | 0.7733 | 16.9662 | 0.8286 | 0.1354 |
| BioLORD-2023 | Synthetic | natural_language | 11 | 0.9167 | 0.6643 | 1.0000 | 0.5841 | 0.7365 | 0.9400 | 0.8096 | 1.0000 | 17.8686 | 0.6906 | 0.1202 |
| BioLORD-2023 | Synthetic | natural_language | 12 | 1.0000 | 0.6587 | 0.9849 | 0.5716 | 0.7294 | 0.8700 | 0.8037 | 0.9851 | 20.3621 | 0.3979 | 0.1111 |
| ClinicalBERT | MTSamples | keyword | 0 | 0.0000 | 0.0468 | 0.0000 | 0.0248 | 0.0754 | 0.1900 | 0.0974 | 0.0576 | 12.9618 | 0.7587 | 0.2211 |
| ClinicalBERT | MTSamples | keyword | 1 | 0.1667 | 0.1412 | 0.6870 | 0.0991 | 0.1856 | 0.4400 | 0.1669 | 0.5529 | 14.0443 | 0.9422 | 0.1638 |
| ClinicalBERT | MTSamples | keyword | 2 | 0.3333 | 0.0682 | 0.1557 | 0.0378 | 0.1032 | 0.2300 | 0.1105 | 0.1504 | 11.9928 | 0.9687 | 0.1920 |
| ClinicalBERT | MTSamples | keyword | 3 | 0.5000 | 0.0667 | 0.1448 | 0.0376 | 0.0997 | 0.2200 | 0.1022 | 0.0911 | 11.3633 | 0.9755 | 0.1901 |
| ClinicalBERT | MTSamples | keyword | 4 | 0.6667 | 0.0656 | 0.1368 | 0.0341 | 0.1037 | 0.1800 | 0.0938 | 0.0317 | 10.1619 | 0.9690 | 0.2207 |
| ClinicalBERT | MTSamples | keyword | 5 | 0.8333 | 0.0698 | 0.1674 | 0.0388 | 0.1085 | 0.2200 | 0.0894 | 0.0000 | 11.0192 | 0.9675 | 0.2071 |
| ClinicalBERT | MTSamples | keyword | 6 | 1.0000 | 0.1842 | 1.0000 | 0.1269 | 0.2494 | 0.4000 | 0.2296 | 1.0000 | 13.8535 | 0.9083 | 0.2003 |
| ClinicalBERT | MTSamples | natural_language | 0 | 0.0000 | 0.0687 | 0.0545 | 0.0298 | 0.1138 | 0.1600 | 0.0974 | 0.0576 | 12.9618 | 0.7587 | 0.2211 |
| ClinicalBERT | MTSamples | natural_language | 1 | 0.1667 | 0.1412 | 0.3894 | 0.0869 | 0.2021 | 0.3600 | 0.1669 | 0.5529 | 14.0443 | 0.9422 | 0.1638 |
| ClinicalBERT | MTSamples | natural_language | 2 | 0.3333 | 0.0848 | 0.1289 | 0.0425 | 0.1315 | 0.2200 | 0.1105 | 0.1504 | 11.9928 | 0.9687 | 0.1920 |
| ClinicalBERT | MTSamples | natural_language | 3 | 0.5000 | 0.0745 | 0.0813 | 0.0369 | 0.1198 | 0.2000 | 0.1022 | 0.0911 | 11.3633 | 0.9755 | 0.1901 |
| ClinicalBERT | MTSamples | natural_language | 4 | 0.6667 | 0.0569 | 0.0000 | 0.0319 | 0.0873 | 0.2100 | 0.0938 | 0.0317 | 10.1619 | 0.9690 | 0.2207 |
| ClinicalBERT | MTSamples | natural_language | 5 | 0.8333 | 0.0658 | 0.0411 | 0.0387 | 0.0995 | 0.2300 | 0.0894 | 0.0000 | 11.0192 | 0.9675 | 0.2071 |
| ClinicalBERT | MTSamples | natural_language | 6 | 1.0000 | 0.2734 | 1.0000 | 0.2120 | 0.3375 | 0.6300 | 0.2296 | 1.0000 | 13.8535 | 0.9083 | 0.2003 |
| ClinicalBERT | PMC-Patients | keyword | 0 | 0.0000 | 0.0443 | 0.0000 | 0.0221 | 0.0711 | 0.1800 | 0.0974 | 0.0576 | 9.7717 | 0.7401 | 0.2909 |
| ClinicalBERT | PMC-Patients | keyword | 1 | 0.1667 | 0.0804 | 0.3963 | 0.0508 | 0.1188 | 0.2900 | 0.1669 | 0.5529 | 14.8941 | 0.9476 | 0.1858 |
| ClinicalBERT | PMC-Patients | keyword | 2 | 0.3333 | 0.0552 | 0.1196 | 0.0291 | 0.0861 | 0.1800 | 0.1105 | 0.1504 | 10.2641 | 0.9719 | 0.2616 |
| ClinicalBERT | PMC-Patients | keyword | 3 | 0.5000 | 0.0495 | 0.0571 | 0.0252 | 0.0793 | 0.1800 | 0.1022 | 0.0911 | 10.7225 | 0.9781 | 0.2455 |
| ClinicalBERT | PMC-Patients | keyword | 4 | 0.6667 | 0.0480 | 0.0406 | 0.0234 | 0.0784 | 0.1600 | 0.0938 | 0.0317 | 10.8505 | 0.9739 | 0.2383 |
| ClinicalBERT | PMC-Patients | keyword | 5 | 0.8333 | 0.0558 | 0.1262 | 0.0314 | 0.0865 | 0.2000 | 0.0894 | 0.0000 | 12.2905 | 0.9748 | 0.1983 |
| ClinicalBERT | PMC-Patients | keyword | 6 | 1.0000 | 0.1354 | 1.0000 | 0.0835 | 0.1954 | 0.3200 | 0.2296 | 1.0000 | 19.1024 | 0.9188 | 0.1406 |
| ClinicalBERT | PMC-Patients | natural_language | 0 | 0.0000 | 0.0740 | 0.0245 | 0.0361 | 0.1212 | 0.2200 | 0.0974 | 0.0576 | 9.7717 | 0.7401 | 0.2909 |
| ClinicalBERT | PMC-Patients | natural_language | 1 | 0.1667 | 0.1352 | 0.3507 | 0.0805 | 0.1989 | 0.2800 | 0.1669 | 0.5529 | 14.8941 | 0.9476 | 0.1858 |
| ClinicalBERT | PMC-Patients | natural_language | 2 | 0.3333 | 0.0778 | 0.0448 | 0.0416 | 0.1210 | 0.2300 | 0.1105 | 0.1504 | 10.2641 | 0.9719 | 0.2616 |
| ClinicalBERT | PMC-Patients | natural_language | 3 | 0.5000 | 0.0712 | 0.0096 | 0.0349 | 0.1153 | 0.1900 | 0.1022 | 0.0911 | 10.7225 | 0.9781 | 0.2455 |
| ClinicalBERT | PMC-Patients | natural_language | 4 | 0.6667 | 0.0694 | 0.0000 | 0.0329 | 0.1120 | 0.1800 | 0.0938 | 0.0317 | 10.8505 | 0.9739 | 0.2383 |
| ClinicalBERT | PMC-Patients | natural_language | 5 | 0.8333 | 0.0800 | 0.0565 | 0.0420 | 0.1243 | 0.2000 | 0.0894 | 0.0000 | 12.2905 | 0.9748 | 0.1983 |
| ClinicalBERT | PMC-Patients | natural_language | 6 | 1.0000 | 0.2570 | 1.0000 | 0.1856 | 0.3315 | 0.4900 | 0.2296 | 1.0000 | 19.1024 | 0.9188 | 0.1406 |
| ClinicalBERT | Synthetic | keyword | 0 | 0.0000 | 0.2138 | 1.0000 | 0.1478 | 0.2827 | 0.4500 | 0.0974 | 0.0576 | 17.3333 | 0.8856 | 0.1719 |
| ClinicalBERT | Synthetic | keyword | 1 | 0.1667 | 0.2114 | 0.9678 | 0.1520 | 0.2758 | 0.4500 | 0.1669 | 0.5529 | 13.8036 | 0.9744 | 0.2116 |
| ClinicalBERT | Synthetic | keyword | 2 | 0.3333 | 0.1888 | 0.6644 | 0.1315 | 0.2558 | 0.4100 | 0.1105 | 0.1504 | 12.7804 | 0.9883 | 0.2218 |
| ClinicalBERT | Synthetic | keyword | 3 | 0.5000 | 0.1967 | 0.7705 | 0.1361 | 0.2658 | 0.4100 | 0.1022 | 0.0911 | 13.1897 | 0.9911 | 0.2049 |
| ClinicalBERT | Synthetic | keyword | 4 | 0.6667 | 0.1861 | 0.6282 | 0.1264 | 0.2511 | 0.4100 | 0.0938 | 0.0317 | 13.1757 | 0.9888 | 0.1964 |
| ClinicalBERT | Synthetic | keyword | 5 | 0.8333 | 0.1393 | 0.0000 | 0.0916 | 0.1900 | 0.3500 | 0.0894 | 0.0000 | 13.6666 | 0.9876 | 0.1890 |
| ClinicalBERT | Synthetic | keyword | 6 | 1.0000 | 0.2107 | 0.9584 | 0.1524 | 0.2730 | 0.5100 | 0.2296 | 1.0000 | 15.7276 | 0.9621 | 0.1671 |
| ClinicalBERT | Synthetic | natural_language | 0 | 0.0000 | 0.1371 | 0.0606 | 0.1046 | 0.1753 | 0.5500 | 0.0974 | 0.0576 | 17.3333 | 0.8856 | 0.1719 |
| ClinicalBERT | Synthetic | natural_language | 1 | 0.1667 | 0.2921 | 0.8700 | 0.2221 | 0.3630 | 0.5900 | 0.1669 | 0.5529 | 13.8036 | 0.9744 | 0.2116 |
| ClinicalBERT | Synthetic | natural_language | 2 | 0.3333 | 0.1880 | 0.3264 | 0.1316 | 0.2466 | 0.4900 | 0.1105 | 0.1504 | 12.7804 | 0.9883 | 0.2218 |
| ClinicalBERT | Synthetic | natural_language | 3 | 0.5000 | 0.1543 | 0.1504 | 0.1005 | 0.2103 | 0.3500 | 0.1022 | 0.0911 | 13.1897 | 0.9911 | 0.2049 |
| ClinicalBERT | Synthetic | natural_language | 4 | 0.6667 | 0.1369 | 0.0595 | 0.0829 | 0.1969 | 0.3200 | 0.0938 | 0.0317 | 13.1757 | 0.9888 | 0.1964 |
| ClinicalBERT | Synthetic | natural_language | 5 | 0.8333 | 0.1255 | 0.0000 | 0.0823 | 0.1761 | 0.3600 | 0.0894 | 0.0000 | 13.6666 | 0.9876 | 0.1890 |
| ClinicalBERT | Synthetic | natural_language | 6 | 1.0000 | 0.3170 | 1.0000 | 0.2496 | 0.3905 | 0.7000 | 0.2296 | 1.0000 | 15.7276 | 0.9621 | 0.1671 |
| E5-Mistral-7B | MTSamples | keyword | 0 | 0.0000 | 0.2461 | 0.6521 | 0.1979 | 0.3010 | 0.9700 | 0.3789 | 0.9704 | 1.9942 | 0.8744 | 0.6806 |
| E5-Mistral-7B | MTSamples | keyword | 1 | 0.0312 | 0.0217 | 0.0000 | 0.0088 | 0.0389 | 0.1000 | 0.0424 | 0.0202 | 6.9789 | 0.8365 | 0.3385 |
| E5-Mistral-7B | MTSamples | keyword | 2 | 0.0625 | 0.0295 | 0.0227 | 0.0087 | 0.0567 | 0.0800 | 0.0363 | 0.0030 | 16.8220 | 0.7204 | 0.1791 |
| E5-Mistral-7B | MTSamples | keyword | 3 | 0.0938 | 0.0307 | 0.0262 | 0.0146 | 0.0522 | 0.1100 | 0.0353 | 0.0000 | 14.9985 | 0.7379 | 0.1984 |
| E5-Mistral-7B | MTSamples | keyword | 4 | 0.1250 | 0.0470 | 0.0735 | 0.0179 | 0.0787 | 0.1400 | 0.0430 | 0.0219 | 22.7802 | 0.6283 | 0.1404 |
| E5-Mistral-7B | MTSamples | keyword | 5 | 0.1562 | 0.0275 | 0.0169 | 0.0129 | 0.0444 | 0.1300 | 0.0378 | 0.0071 | 25.2754 | 0.5639 | 0.1320 |
| E5-Mistral-7B | MTSamples | keyword | 6 | 0.1875 | 0.0642 | 0.1235 | 0.0344 | 0.0960 | 0.2000 | 0.0502 | 0.0422 | 26.5242 | 0.5452 | 0.1317 |
| E5-Mistral-7B | MTSamples | keyword | 7 | 0.2188 | 0.0472 | 0.0741 | 0.0232 | 0.0763 | 0.1700 | 0.0430 | 0.0219 | 27.4315 | 0.5032 | 0.1287 |
| E5-Mistral-7B | MTSamples | keyword | 8 | 0.2500 | 0.0531 | 0.0913 | 0.0243 | 0.0888 | 0.1700 | 0.0492 | 0.0393 | 28.9038 | 0.4796 | 0.1231 |
| E5-Mistral-7B | MTSamples | keyword | 9 | 0.2812 | 0.0438 | 0.0642 | 0.0203 | 0.0727 | 0.1700 | 0.0522 | 0.0479 | 31.1120 | 0.4529 | 0.1170 |
| E5-Mistral-7B | MTSamples | keyword | 10 | 0.3125 | 0.0587 | 0.1075 | 0.0281 | 0.0932 | 0.1600 | 0.0447 | 0.0266 | 29.5847 | 0.4510 | 0.1263 |
| E5-Mistral-7B | MTSamples | keyword | 11 | 0.3438 | 0.0553 | 0.0976 | 0.0249 | 0.0920 | 0.1500 | 0.0445 | 0.0261 | 30.2516 | 0.4417 | 0.1271 |
| E5-Mistral-7B | MTSamples | keyword | 12 | 0.3750 | 0.0379 | 0.0471 | 0.0153 | 0.0657 | 0.1500 | 0.0452 | 0.0281 | 31.1429 | 0.4460 | 0.1242 |
| E5-Mistral-7B | MTSamples | keyword | 13 | 0.4062 | 0.0532 | 0.0915 | 0.0246 | 0.0888 | 0.1400 | 0.0495 | 0.0403 | 31.2594 | 0.4514 | 0.1217 |
| E5-Mistral-7B | MTSamples | keyword | 14 | 0.4375 | 0.0489 | 0.0790 | 0.0231 | 0.0802 | 0.1500 | 0.0458 | 0.0297 | 30.6135 | 0.4367 | 0.1258 |
| E5-Mistral-7B | MTSamples | keyword | 15 | 0.4688 | 0.0370 | 0.0445 | 0.0181 | 0.0581 | 0.1500 | 0.0488 | 0.0383 | 29.3536 | 0.4388 | 0.1290 |
| E5-Mistral-7B | MTSamples | keyword | 16 | 0.5000 | 0.0320 | 0.0299 | 0.0167 | 0.0484 | 0.1500 | 0.0586 | 0.0659 | 28.5411 | 0.4610 | 0.1292 |
| E5-Mistral-7B | MTSamples | keyword | 17 | 0.5312 | 0.0353 | 0.0395 | 0.0165 | 0.0552 | 0.1500 | 0.0606 | 0.0714 | 26.5639 | 0.5080 | 0.1353 |
| E5-Mistral-7B | MTSamples | keyword | 18 | 0.5625 | 0.0354 | 0.0398 | 0.0183 | 0.0543 | 0.1600 | 0.0568 | 0.0608 | 26.6895 | 0.5152 | 0.1327 |
| E5-Mistral-7B | MTSamples | keyword | 19 | 0.5938 | 0.0532 | 0.0915 | 0.0276 | 0.0956 | 0.1900 | 0.0724 | 0.1048 | 25.8848 | 0.5413 | 0.1318 |
| E5-Mistral-7B | MTSamples | keyword | 20 | 0.6250 | 0.0849 | 0.1837 | 0.0433 | 0.1171 | 0.2300 | 0.1302 | 0.2681 | 24.9870 | 0.5737 | 0.1336 |
| E5-Mistral-7B | MTSamples | keyword | 21 | 0.6562 | 0.0962 | 0.2165 | 0.0574 | 0.1392 | 0.2800 | 0.1754 | 0.3957 | 23.5615 | 0.6214 | 0.1369 |
| E5-Mistral-7B | MTSamples | keyword | 22 | 0.6875 | 0.1391 | 0.3412 | 0.0833 | 0.1899 | 0.3100 | 0.2094 | 0.4918 | 22.4827 | 0.6462 | 0.1378 |
| E5-Mistral-7B | MTSamples | keyword | 23 | 0.7188 | 0.1396 | 0.3426 | 0.0852 | 0.1808 | 0.3300 | 0.2282 | 0.5448 | 21.8611 | 0.6506 | 0.1422 |
| E5-Mistral-7B | MTSamples | keyword | 24 | 0.7500 | 0.1377 | 0.3371 | 0.0858 | 0.1856 | 0.3400 | 0.2317 | 0.5547 | 21.7475 | 0.6540 | 0.1429 |
| E5-Mistral-7B | MTSamples | keyword | 25 | 0.7812 | 0.1577 | 0.3952 | 0.0990 | 0.2072 | 0.3500 | 0.2471 | 0.5981 | 21.5338 | 0.6493 | 0.1440 |
| E5-Mistral-7B | MTSamples | keyword | 26 | 0.8125 | 0.1877 | 0.4824 | 0.1224 | 0.2463 | 0.3900 | 0.2490 | 0.6036 | 21.8040 | 0.6488 | 0.1428 |
| E5-Mistral-7B | MTSamples | keyword | 27 | 0.8438 | 0.1752 | 0.4461 | 0.1140 | 0.2291 | 0.3700 | 0.2496 | 0.6052 | 22.0520 | 0.6613 | 0.1402 |
| E5-Mistral-7B | MTSamples | keyword | 28 | 0.8750 | 0.1596 | 0.4008 | 0.1014 | 0.2125 | 0.3300 | 0.2384 | 0.5734 | 22.3349 | 0.6625 | 0.1387 |
| E5-Mistral-7B | MTSamples | keyword | 29 | 0.9062 | 0.2259 | 0.5934 | 0.1548 | 0.2959 | 0.4200 | 0.2770 | 0.6827 | 23.3984 | 0.6640 | 0.1356 |
| E5-Mistral-7B | MTSamples | keyword | 30 | 0.9375 | 0.3635 | 0.9933 | 0.2823 | 0.4448 | 0.5700 | 0.3622 | 0.9233 | 25.5301 | 0.6717 | 0.1296 |
| E5-Mistral-7B | MTSamples | keyword | 31 | 0.9688 | 0.3658 | 1.0000 | 0.2809 | 0.4413 | 0.5500 | 0.3894 | 1.0000 | 26.0774 | 0.6968 | 0.1286 |
| E5-Mistral-7B | MTSamples | keyword | 32 | 1.0000 | 0.1709 | 0.4336 | 0.1044 | 0.2335 | 0.2900 | 0.3179 | 0.7981 | 19.1466 | 0.6857 | 0.1780 |
| E5-Mistral-7B | MTSamples | natural_language | 0 | 0.0000 | 0.9100 | 1.0000 | 0.8500 | 0.9600 | 0.9100 | 0.3789 | 0.9704 | 1.9942 | 0.8744 | 0.6806 |
| E5-Mistral-7B | MTSamples | natural_language | 1 | 0.0312 | 0.0874 | 0.0708 | 0.0381 | 0.1340 | 0.1700 | 0.0424 | 0.0202 | 6.9789 | 0.8365 | 0.3385 |
| E5-Mistral-7B | MTSamples | natural_language | 2 | 0.0625 | 0.0400 | 0.0173 | 0.0143 | 0.0613 | 0.1300 | 0.0363 | 0.0030 | 16.8220 | 0.7204 | 0.1791 |
| E5-Mistral-7B | MTSamples | natural_language | 3 | 0.0938 | 0.0456 | 0.0236 | 0.0178 | 0.0696 | 0.1400 | 0.0353 | 0.0000 | 14.9985 | 0.7379 | 0.1984 |
| E5-Mistral-7B | MTSamples | natural_language | 4 | 0.1250 | 0.0347 | 0.0113 | 0.0083 | 0.0667 | 0.0800 | 0.0430 | 0.0219 | 22.7802 | 0.6283 | 0.1404 |
| E5-Mistral-7B | MTSamples | natural_language | 5 | 0.1562 | 0.0247 | 0.0000 | 0.0079 | 0.0480 | 0.1100 | 0.0378 | 0.0071 | 25.2754 | 0.5639 | 0.1320 |
| E5-Mistral-7B | MTSamples | natural_language | 6 | 0.1875 | 0.0368 | 0.0137 | 0.0108 | 0.0573 | 0.1100 | 0.0502 | 0.0422 | 26.5242 | 0.5452 | 0.1317 |
| E5-Mistral-7B | MTSamples | natural_language | 7 | 0.2188 | 0.0359 | 0.0127 | 0.0134 | 0.0637 | 0.1200 | 0.0430 | 0.0219 | 27.4315 | 0.5032 | 0.1287 |
| E5-Mistral-7B | MTSamples | natural_language | 8 | 0.2500 | 0.0289 | 0.0047 | 0.0106 | 0.0530 | 0.1200 | 0.0492 | 0.0393 | 28.9038 | 0.4796 | 0.1231 |
| E5-Mistral-7B | MTSamples | natural_language | 9 | 0.2812 | 0.0503 | 0.0289 | 0.0202 | 0.0865 | 0.1400 | 0.0522 | 0.0479 | 31.1120 | 0.4529 | 0.1170 |
| E5-Mistral-7B | MTSamples | natural_language | 10 | 0.3125 | 0.0309 | 0.0070 | 0.0148 | 0.0627 | 0.1400 | 0.0447 | 0.0266 | 29.5847 | 0.4510 | 0.1263 |
| E5-Mistral-7B | MTSamples | natural_language | 11 | 0.3438 | 0.0326 | 0.0089 | 0.0168 | 0.0519 | 0.1600 | 0.0445 | 0.0261 | 30.2516 | 0.4417 | 0.1271 |
| E5-Mistral-7B | MTSamples | natural_language | 12 | 0.3750 | 0.0428 | 0.0204 | 0.0201 | 0.0708 | 0.1700 | 0.0452 | 0.0281 | 31.1429 | 0.4460 | 0.1242 |
| E5-Mistral-7B | MTSamples | natural_language | 13 | 0.4062 | 0.0462 | 0.0243 | 0.0231 | 0.0753 | 0.1900 | 0.0495 | 0.0403 | 31.2594 | 0.4514 | 0.1217 |
| E5-Mistral-7B | MTSamples | natural_language | 14 | 0.4375 | 0.0443 | 0.0221 | 0.0213 | 0.0740 | 0.1800 | 0.0458 | 0.0297 | 30.6135 | 0.4367 | 0.1258 |
| E5-Mistral-7B | MTSamples | natural_language | 15 | 0.4688 | 0.0600 | 0.0399 | 0.0299 | 0.1005 | 0.1800 | 0.0488 | 0.0383 | 29.3536 | 0.4388 | 0.1290 |
| E5-Mistral-7B | MTSamples | natural_language | 16 | 0.5000 | 0.0637 | 0.0441 | 0.0348 | 0.0978 | 0.2300 | 0.0586 | 0.0659 | 28.5411 | 0.4610 | 0.1292 |
| E5-Mistral-7B | MTSamples | natural_language | 17 | 0.5312 | 0.0647 | 0.0452 | 0.0355 | 0.1026 | 0.2000 | 0.0606 | 0.0714 | 26.5639 | 0.5080 | 0.1353 |
| E5-Mistral-7B | MTSamples | natural_language | 18 | 0.5625 | 0.0690 | 0.0500 | 0.0364 | 0.1056 | 0.2200 | 0.0568 | 0.0608 | 26.6895 | 0.5152 | 0.1327 |
| E5-Mistral-7B | MTSamples | natural_language | 19 | 0.5938 | 0.0846 | 0.0677 | 0.0457 | 0.1293 | 0.2400 | 0.0724 | 0.1048 | 25.8848 | 0.5413 | 0.1318 |
| E5-Mistral-7B | MTSamples | natural_language | 20 | 0.6250 | 0.1271 | 0.1157 | 0.0759 | 0.1852 | 0.3300 | 0.1302 | 0.2681 | 24.9870 | 0.5737 | 0.1336 |
| E5-Mistral-7B | MTSamples | natural_language | 21 | 0.6562 | 0.1551 | 0.1473 | 0.0999 | 0.2144 | 0.3500 | 0.1754 | 0.3957 | 23.5615 | 0.6214 | 0.1369 |
| E5-Mistral-7B | MTSamples | natural_language | 22 | 0.6875 | 0.1637 | 0.1570 | 0.1085 | 0.2250 | 0.3800 | 0.2094 | 0.4918 | 22.4827 | 0.6462 | 0.1378 |
| E5-Mistral-7B | MTSamples | natural_language | 23 | 0.7188 | 0.1943 | 0.1916 | 0.1277 | 0.2563 | 0.4200 | 0.2282 | 0.5448 | 21.8611 | 0.6506 | 0.1422 |
| E5-Mistral-7B | MTSamples | natural_language | 24 | 0.7500 | 0.1993 | 0.1972 | 0.1323 | 0.2596 | 0.4500 | 0.2317 | 0.5547 | 21.7475 | 0.6540 | 0.1429 |
| E5-Mistral-7B | MTSamples | natural_language | 25 | 0.7812 | 0.2134 | 0.2131 | 0.1477 | 0.2785 | 0.4500 | 0.2471 | 0.5981 | 21.5338 | 0.6493 | 0.1440 |
| E5-Mistral-7B | MTSamples | natural_language | 26 | 0.8125 | 0.2389 | 0.2420 | 0.1777 | 0.3081 | 0.5000 | 0.2490 | 0.6036 | 21.8040 | 0.6488 | 0.1428 |
| E5-Mistral-7B | MTSamples | natural_language | 27 | 0.8438 | 0.2391 | 0.2422 | 0.1691 | 0.3017 | 0.5300 | 0.2496 | 0.6052 | 22.0520 | 0.6613 | 0.1402 |
| E5-Mistral-7B | MTSamples | natural_language | 28 | 0.8750 | 0.2391 | 0.2422 | 0.1695 | 0.3037 | 0.5400 | 0.2384 | 0.5734 | 22.3349 | 0.6625 | 0.1387 |
| E5-Mistral-7B | MTSamples | natural_language | 29 | 0.9062 | 0.3469 | 0.3639 | 0.2638 | 0.4190 | 0.5800 | 0.2770 | 0.6827 | 23.3984 | 0.6640 | 0.1356 |
| E5-Mistral-7B | MTSamples | natural_language | 30 | 0.9375 | 0.4665 | 0.4990 | 0.3807 | 0.5547 | 0.6500 | 0.3622 | 0.9233 | 25.5301 | 0.6717 | 0.1296 |
| E5-Mistral-7B | MTSamples | natural_language | 31 | 0.9688 | 0.5270 | 0.5674 | 0.4371 | 0.6060 | 0.7200 | 0.3894 | 1.0000 | 26.0774 | 0.6968 | 0.1286 |
| E5-Mistral-7B | MTSamples | natural_language | 32 | 1.0000 | 0.4175 | 0.4437 | 0.3297 | 0.4965 | 0.6100 | 0.3179 | 0.7981 | 19.1466 | 0.6857 | 0.1780 |
| E5-Mistral-7B | PMC-Patients | keyword | 0 | 0.0000 | 0.1372 | 0.2308 | 0.0900 | 0.1891 | 0.3700 | 0.3789 | 0.9704 | 2.3654 | 0.8019 | 0.6137 |
| E5-Mistral-7B | PMC-Patients | keyword | 1 | 0.0312 | 0.0216 | 0.0000 | 0.0088 | 0.0381 | 0.1000 | 0.0424 | 0.0202 | 6.5222 | 0.8011 | 0.3518 |
| E5-Mistral-7B | PMC-Patients | keyword | 2 | 0.0625 | 0.0333 | 0.0234 | 0.0087 | 0.0663 | 0.0800 | 0.0363 | 0.0030 | 12.4588 | 0.6778 | 0.2465 |
| E5-Mistral-7B | PMC-Patients | keyword | 3 | 0.0938 | 0.0253 | 0.0074 | 0.0062 | 0.0513 | 0.0800 | 0.0353 | 0.0000 | 10.2105 | 0.7252 | 0.2838 |
| E5-Mistral-7B | PMC-Patients | keyword | 4 | 0.1250 | 0.0571 | 0.0709 | 0.0267 | 0.0911 | 0.1700 | 0.0430 | 0.0219 | 14.5085 | 0.6321 | 0.2295 |
| E5-Mistral-7B | PMC-Patients | keyword | 5 | 0.1562 | 0.0567 | 0.0701 | 0.0280 | 0.0941 | 0.1500 | 0.0378 | 0.0071 | 19.9921 | 0.6015 | 0.1834 |
| E5-Mistral-7B | PMC-Patients | keyword | 6 | 0.1875 | 0.0863 | 0.1292 | 0.0476 | 0.1286 | 0.2600 | 0.0502 | 0.0422 | 23.7191 | 0.5980 | 0.1566 |
| E5-Mistral-7B | PMC-Patients | keyword | 7 | 0.2188 | 0.0726 | 0.1018 | 0.0348 | 0.1183 | 0.1800 | 0.0430 | 0.0219 | 25.5541 | 0.5647 | 0.1429 |
| E5-Mistral-7B | PMC-Patients | keyword | 8 | 0.2500 | 0.0865 | 0.1296 | 0.0497 | 0.1293 | 0.2300 | 0.0492 | 0.0393 | 29.0045 | 0.5463 | 0.1271 |
| E5-Mistral-7B | PMC-Patients | keyword | 9 | 0.2812 | 0.1035 | 0.1635 | 0.0532 | 0.1594 | 0.1800 | 0.0522 | 0.0479 | 34.1136 | 0.4872 | 0.1114 |
| E5-Mistral-7B | PMC-Patients | keyword | 10 | 0.3125 | 0.0722 | 0.1010 | 0.0356 | 0.1161 | 0.1900 | 0.0447 | 0.0266 | 32.5443 | 0.4812 | 0.1157 |
| E5-Mistral-7B | PMC-Patients | keyword | 11 | 0.3438 | 0.0789 | 0.1144 | 0.0389 | 0.1251 | 0.1800 | 0.0445 | 0.0261 | 34.4479 | 0.4735 | 0.1118 |
| E5-Mistral-7B | PMC-Patients | keyword | 12 | 0.3750 | 0.0738 | 0.1042 | 0.0368 | 0.1157 | 0.1800 | 0.0452 | 0.0281 | 36.6276 | 0.4795 | 0.1047 |
| E5-Mistral-7B | PMC-Patients | keyword | 13 | 0.4062 | 0.0776 | 0.1118 | 0.0381 | 0.1231 | 0.1700 | 0.0495 | 0.0403 | 35.4335 | 0.4877 | 0.1083 |
| E5-Mistral-7B | PMC-Patients | keyword | 14 | 0.4375 | 0.0682 | 0.0930 | 0.0301 | 0.1154 | 0.1500 | 0.0458 | 0.0297 | 36.5930 | 0.4743 | 0.1058 |
| E5-Mistral-7B | PMC-Patients | keyword | 15 | 0.4688 | 0.0669 | 0.0904 | 0.0303 | 0.1080 | 0.1600 | 0.0488 | 0.0383 | 37.7001 | 0.4635 | 0.1022 |
| E5-Mistral-7B | PMC-Patients | keyword | 16 | 0.5000 | 0.0877 | 0.1320 | 0.0467 | 0.1366 | 0.2000 | 0.0586 | 0.0659 | 36.7642 | 0.4849 | 0.1059 |
| E5-Mistral-7B | PMC-Patients | keyword | 17 | 0.5312 | 0.0868 | 0.1302 | 0.0462 | 0.1339 | 0.2200 | 0.0606 | 0.0714 | 35.0411 | 0.5317 | 0.1084 |
| E5-Mistral-7B | PMC-Patients | keyword | 18 | 0.5625 | 0.0796 | 0.1158 | 0.0402 | 0.1231 | 0.1900 | 0.0568 | 0.0608 | 34.6539 | 0.5235 | 0.1071 |
| E5-Mistral-7B | PMC-Patients | keyword | 19 | 0.5938 | 0.1077 | 0.1719 | 0.0605 | 0.1614 | 0.2400 | 0.0724 | 0.1048 | 34.0126 | 0.5459 | 0.1044 |
| E5-Mistral-7B | PMC-Patients | keyword | 20 | 0.6250 | 0.1912 | 0.3386 | 0.1247 | 0.2596 | 0.3700 | 0.1302 | 0.2681 | 34.4118 | 0.5647 | 0.0999 |
| E5-Mistral-7B | PMC-Patients | keyword | 21 | 0.6562 | 0.2358 | 0.4276 | 0.1669 | 0.3058 | 0.4400 | 0.1754 | 0.3957 | 34.6795 | 0.6136 | 0.0942 |
| E5-Mistral-7B | PMC-Patients | keyword | 22 | 0.6875 | 0.3254 | 0.6065 | 0.2505 | 0.4049 | 0.5400 | 0.2094 | 0.4918 | 34.9592 | 0.6422 | 0.0916 |
| E5-Mistral-7B | PMC-Patients | keyword | 23 | 0.7188 | 0.3475 | 0.6506 | 0.2675 | 0.4315 | 0.5600 | 0.2282 | 0.5448 | 34.7777 | 0.6485 | 0.0918 |
| E5-Mistral-7B | PMC-Patients | keyword | 24 | 0.7500 | 0.3517 | 0.6590 | 0.2695 | 0.4341 | 0.5800 | 0.2317 | 0.5547 | 35.9782 | 0.6488 | 0.0861 |
| E5-Mistral-7B | PMC-Patients | keyword | 25 | 0.7812 | 0.3870 | 0.7295 | 0.3107 | 0.4753 | 0.6000 | 0.2471 | 0.5981 | 36.4246 | 0.6456 | 0.0852 |
| E5-Mistral-7B | PMC-Patients | keyword | 26 | 0.8125 | 0.3601 | 0.6758 | 0.2861 | 0.4425 | 0.5900 | 0.2490 | 0.6036 | 37.4888 | 0.6546 | 0.0816 |
| E5-Mistral-7B | PMC-Patients | keyword | 27 | 0.8438 | 0.3810 | 0.7175 | 0.3057 | 0.4669 | 0.6200 | 0.2496 | 0.6052 | 38.4784 | 0.6664 | 0.0808 |
| E5-Mistral-7B | PMC-Patients | keyword | 28 | 0.8750 | 0.3519 | 0.6594 | 0.2729 | 0.4335 | 0.6000 | 0.2384 | 0.5734 | 39.2939 | 0.6684 | 0.0775 |
| E5-Mistral-7B | PMC-Patients | keyword | 29 | 0.9062 | 0.3909 | 0.7373 | 0.2997 | 0.4790 | 0.6200 | 0.2770 | 0.6827 | 41.1894 | 0.6590 | 0.0730 |
| E5-Mistral-7B | PMC-Patients | keyword | 30 | 0.9375 | 0.4964 | 0.9479 | 0.4124 | 0.5847 | 0.6700 | 0.3622 | 0.9233 | 43.9776 | 0.6691 | 0.0676 |
| E5-Mistral-7B | PMC-Patients | keyword | 31 | 0.9688 | 0.5225 | 1.0000 | 0.4386 | 0.6087 | 0.7400 | 0.3894 | 1.0000 | 45.7223 | 0.6896 | 0.0660 |
| E5-Mistral-7B | PMC-Patients | keyword | 32 | 1.0000 | 0.5051 | 0.9653 | 0.4207 | 0.5935 | 0.7000 | 0.3179 | 0.7981 | 43.4814 | 0.6902 | 0.0699 |
| E5-Mistral-7B | PMC-Patients | natural_language | 0 | 0.0000 | 0.8000 | 1.0000 | 0.7200 | 0.8800 | 0.8000 | 0.3789 | 0.9704 | 2.3654 | 0.8019 | 0.6137 |
| E5-Mistral-7B | PMC-Patients | natural_language | 1 | 0.0312 | 0.0338 | 0.0198 | 0.0123 | 0.0627 | 0.1200 | 0.0424 | 0.0202 | 6.5222 | 0.8011 | 0.3518 |
| E5-Mistral-7B | PMC-Patients | natural_language | 2 | 0.0625 | 0.0238 | 0.0070 | 0.0096 | 0.0422 | 0.1100 | 0.0363 | 0.0030 | 12.4588 | 0.6778 | 0.2465 |
| E5-Mistral-7B | PMC-Patients | natural_language | 3 | 0.0938 | 0.0183 | 0.0000 | 0.0068 | 0.0333 | 0.0900 | 0.0353 | 0.0000 | 10.2105 | 0.7252 | 0.2838 |
| E5-Mistral-7B | PMC-Patients | natural_language | 4 | 0.1250 | 0.0318 | 0.0173 | 0.0112 | 0.0579 | 0.1100 | 0.0430 | 0.0219 | 14.5085 | 0.6321 | 0.2295 |
| E5-Mistral-7B | PMC-Patients | natural_language | 5 | 0.1562 | 0.0426 | 0.0311 | 0.0148 | 0.0767 | 0.1000 | 0.0378 | 0.0071 | 19.9921 | 0.6015 | 0.1834 |
| E5-Mistral-7B | PMC-Patients | natural_language | 6 | 0.1875 | 0.0435 | 0.0322 | 0.0156 | 0.0756 | 0.1400 | 0.0502 | 0.0422 | 23.7191 | 0.5980 | 0.1566 |
| E5-Mistral-7B | PMC-Patients | natural_language | 7 | 0.2188 | 0.0384 | 0.0257 | 0.0135 | 0.0728 | 0.1200 | 0.0430 | 0.0219 | 25.5541 | 0.5647 | 0.1429 |
| E5-Mistral-7B | PMC-Patients | natural_language | 8 | 0.2500 | 0.0506 | 0.0413 | 0.0216 | 0.0859 | 0.1500 | 0.0492 | 0.0393 | 29.0045 | 0.5463 | 0.1271 |
| E5-Mistral-7B | PMC-Patients | natural_language | 9 | 0.2812 | 0.0585 | 0.0514 | 0.0234 | 0.1025 | 0.1300 | 0.0522 | 0.0479 | 34.1136 | 0.4872 | 0.1114 |
| E5-Mistral-7B | PMC-Patients | natural_language | 10 | 0.3125 | 0.0510 | 0.0418 | 0.0199 | 0.0874 | 0.1400 | 0.0447 | 0.0266 | 32.5443 | 0.4812 | 0.1157 |
| E5-Mistral-7B | PMC-Patients | natural_language | 11 | 0.3438 | 0.0426 | 0.0311 | 0.0157 | 0.0766 | 0.1300 | 0.0445 | 0.0261 | 34.4479 | 0.4735 | 0.1118 |
| E5-Mistral-7B | PMC-Patients | natural_language | 12 | 0.3750 | 0.0526 | 0.0439 | 0.0204 | 0.0923 | 0.1400 | 0.0452 | 0.0281 | 36.6276 | 0.4795 | 0.1047 |
| E5-Mistral-7B | PMC-Patients | natural_language | 13 | 0.4062 | 0.0573 | 0.0499 | 0.0251 | 0.0992 | 0.1500 | 0.0495 | 0.0403 | 35.4335 | 0.4877 | 0.1083 |
| E5-Mistral-7B | PMC-Patients | natural_language | 14 | 0.4375 | 0.0548 | 0.0467 | 0.0247 | 0.0918 | 0.1700 | 0.0458 | 0.0297 | 36.5930 | 0.4743 | 0.1058 |
| E5-Mistral-7B | PMC-Patients | natural_language | 15 | 0.4688 | 0.0654 | 0.0603 | 0.0335 | 0.1165 | 0.1800 | 0.0488 | 0.0383 | 37.7001 | 0.4635 | 0.1022 |
| E5-Mistral-7B | PMC-Patients | natural_language | 16 | 0.5000 | 0.0925 | 0.0949 | 0.0477 | 0.1381 | 0.2000 | 0.0586 | 0.0659 | 36.7642 | 0.4849 | 0.1059 |
| E5-Mistral-7B | PMC-Patients | natural_language | 17 | 0.5312 | 0.1008 | 0.1055 | 0.0552 | 0.1545 | 0.2100 | 0.0606 | 0.0714 | 35.0411 | 0.5317 | 0.1084 |
| E5-Mistral-7B | PMC-Patients | natural_language | 18 | 0.5625 | 0.0893 | 0.0908 | 0.0430 | 0.1399 | 0.1900 | 0.0568 | 0.0608 | 34.6539 | 0.5235 | 0.1071 |
| E5-Mistral-7B | PMC-Patients | natural_language | 19 | 0.5938 | 0.1089 | 0.1159 | 0.0601 | 0.1630 | 0.2700 | 0.0724 | 0.1048 | 34.0126 | 0.5459 | 0.1044 |
| E5-Mistral-7B | PMC-Patients | natural_language | 20 | 0.6250 | 0.2220 | 0.2606 | 0.1568 | 0.2864 | 0.4500 | 0.1302 | 0.2681 | 34.4118 | 0.5647 | 0.0999 |
| E5-Mistral-7B | PMC-Patients | natural_language | 21 | 0.6562 | 0.3527 | 0.4278 | 0.2747 | 0.4383 | 0.5800 | 0.1754 | 0.3957 | 34.6795 | 0.6136 | 0.0942 |
| E5-Mistral-7B | PMC-Patients | natural_language | 22 | 0.6875 | 0.4067 | 0.4969 | 0.3290 | 0.4847 | 0.6600 | 0.2094 | 0.4918 | 34.9592 | 0.6422 | 0.0916 |
| E5-Mistral-7B | PMC-Patients | natural_language | 23 | 0.7188 | 0.4270 | 0.5228 | 0.3425 | 0.5140 | 0.6500 | 0.2282 | 0.5448 | 34.7777 | 0.6485 | 0.0918 |
| E5-Mistral-7B | PMC-Patients | natural_language | 24 | 0.7500 | 0.4428 | 0.5430 | 0.3664 | 0.5318 | 0.7000 | 0.2317 | 0.5547 | 35.9782 | 0.6488 | 0.0861 |
| E5-Mistral-7B | PMC-Patients | natural_language | 25 | 0.7812 | 0.4784 | 0.5886 | 0.3952 | 0.5613 | 0.7400 | 0.2471 | 0.5981 | 36.4246 | 0.6456 | 0.0852 |
| E5-Mistral-7B | PMC-Patients | natural_language | 26 | 0.8125 | 0.4695 | 0.5772 | 0.3832 | 0.5483 | 0.7200 | 0.2490 | 0.6036 | 37.4888 | 0.6546 | 0.0816 |
| E5-Mistral-7B | PMC-Patients | natural_language | 27 | 0.8438 | 0.4762 | 0.5858 | 0.3933 | 0.5653 | 0.7300 | 0.2496 | 0.6052 | 38.4784 | 0.6664 | 0.0808 |
| E5-Mistral-7B | PMC-Patients | natural_language | 28 | 0.8750 | 0.4650 | 0.5714 | 0.3827 | 0.5504 | 0.7400 | 0.2384 | 0.5734 | 39.2939 | 0.6684 | 0.0775 |
| E5-Mistral-7B | PMC-Patients | natural_language | 29 | 0.9062 | 0.4872 | 0.5998 | 0.4029 | 0.5669 | 0.7500 | 0.2770 | 0.6827 | 41.1894 | 0.6590 | 0.0730 |
| E5-Mistral-7B | PMC-Patients | natural_language | 30 | 0.9375 | 0.5962 | 0.7393 | 0.5156 | 0.6800 | 0.8500 | 0.3622 | 0.9233 | 43.9776 | 0.6691 | 0.0676 |
| E5-Mistral-7B | PMC-Patients | natural_language | 31 | 0.9688 | 0.6612 | 0.8224 | 0.5788 | 0.7410 | 0.8300 | 0.3894 | 1.0000 | 45.7223 | 0.6896 | 0.0660 |
| E5-Mistral-7B | PMC-Patients | natural_language | 32 | 1.0000 | 0.6205 | 0.7704 | 0.5378 | 0.7037 | 0.8400 | 0.3179 | 0.7981 | 43.4814 | 0.6902 | 0.0699 |
| E5-Mistral-7B | Synthetic | keyword | 0 | 0.0000 | 0.0304 | 0.0382 | 0.0097 | 0.0585 | 0.1000 | 0.3789 | 0.9704 | 23.4754 | 0.0712 | 0.1434 |
| E5-Mistral-7B | Synthetic | keyword | 1 | 0.0312 | 0.0462 | 0.1722 | 0.0202 | 0.0759 | 0.1400 | 0.0424 | 0.0202 | 25.2446 | 0.2703 | 0.1307 |
| E5-Mistral-7B | Synthetic | keyword | 2 | 0.0625 | 0.0570 | 0.2638 | 0.0307 | 0.0878 | 0.1900 | 0.0363 | 0.0030 | 29.2636 | 0.2103 | 0.1132 |
| E5-Mistral-7B | Synthetic | keyword | 3 | 0.0938 | 0.0593 | 0.2833 | 0.0283 | 0.0959 | 0.1500 | 0.0353 | 0.0000 | 27.6461 | 0.2260 | 0.1184 |
| E5-Mistral-7B | Synthetic | keyword | 4 | 0.1250 | 0.0534 | 0.2332 | 0.0209 | 0.0867 | 0.1500 | 0.0430 | 0.0219 | 30.3155 | 0.2169 | 0.1080 |
| E5-Mistral-7B | Synthetic | keyword | 5 | 0.1562 | 0.0428 | 0.1433 | 0.0142 | 0.0716 | 0.1300 | 0.0378 | 0.0071 | 31.1644 | 0.2280 | 0.1017 |
| E5-Mistral-7B | Synthetic | keyword | 6 | 0.1875 | 0.0403 | 0.1221 | 0.0167 | 0.0718 | 0.1200 | 0.0502 | 0.0422 | 30.3212 | 0.2111 | 0.1047 |
| E5-Mistral-7B | Synthetic | keyword | 7 | 0.2188 | 0.0338 | 0.0670 | 0.0105 | 0.0633 | 0.0900 | 0.0430 | 0.0219 | 30.0111 | 0.1880 | 0.1056 |
| E5-Mistral-7B | Synthetic | keyword | 8 | 0.2500 | 0.0465 | 0.1747 | 0.0182 | 0.0637 | 0.1300 | 0.0492 | 0.0393 | 29.1703 | 0.2098 | 0.1063 |
| E5-Mistral-7B | Synthetic | keyword | 9 | 0.2812 | 0.0269 | 0.0085 | 0.0138 | 0.0483 | 0.1400 | 0.0522 | 0.0479 | 28.6771 | 0.2160 | 0.1050 |
| E5-Mistral-7B | Synthetic | keyword | 10 | 0.3125 | 0.0259 | 0.0000 | 0.0132 | 0.0470 | 0.1300 | 0.0447 | 0.0266 | 28.3744 | 0.2164 | 0.1046 |
| E5-Mistral-7B | Synthetic | keyword | 11 | 0.3438 | 0.0283 | 0.0204 | 0.0148 | 0.0501 | 0.1400 | 0.0445 | 0.0261 | 28.6618 | 0.2366 | 0.1015 |
| E5-Mistral-7B | Synthetic | keyword | 12 | 0.3750 | 0.0333 | 0.0628 | 0.0156 | 0.0546 | 0.1300 | 0.0452 | 0.0281 | 27.8569 | 0.2450 | 0.1061 |
| E5-Mistral-7B | Synthetic | keyword | 13 | 0.4062 | 0.0345 | 0.0729 | 0.0131 | 0.0626 | 0.1100 | 0.0495 | 0.0403 | 27.1003 | 0.2437 | 0.1089 |
| E5-Mistral-7B | Synthetic | keyword | 14 | 0.4375 | 0.0315 | 0.0475 | 0.0133 | 0.0550 | 0.1000 | 0.0458 | 0.0297 | 27.3985 | 0.2438 | 0.1076 |
| E5-Mistral-7B | Synthetic | keyword | 15 | 0.4688 | 0.0397 | 0.1170 | 0.0164 | 0.0533 | 0.1500 | 0.0488 | 0.0383 | 26.8691 | 0.2561 | 0.1102 |
| E5-Mistral-7B | Synthetic | keyword | 16 | 0.5000 | 0.0338 | 0.0670 | 0.0159 | 0.0551 | 0.1300 | 0.0586 | 0.0659 | 26.5046 | 0.2521 | 0.1107 |
| E5-Mistral-7B | Synthetic | keyword | 17 | 0.5312 | 0.0356 | 0.0823 | 0.0156 | 0.0533 | 0.1400 | 0.0606 | 0.0714 | 26.7174 | 0.2513 | 0.1113 |
| E5-Mistral-7B | Synthetic | keyword | 18 | 0.5625 | 0.0339 | 0.0679 | 0.0146 | 0.0531 | 0.1400 | 0.0568 | 0.0608 | 27.2241 | 0.2390 | 0.1115 |
| E5-Mistral-7B | Synthetic | keyword | 19 | 0.5938 | 0.0346 | 0.0738 | 0.0144 | 0.0655 | 0.1200 | 0.0724 | 0.1048 | 27.9020 | 0.2340 | 0.1064 |
| E5-Mistral-7B | Synthetic | keyword | 20 | 0.6250 | 0.0606 | 0.2943 | 0.0319 | 0.1001 | 0.2000 | 0.1302 | 0.2681 | 28.8389 | 0.2391 | 0.1029 |
| E5-Mistral-7B | Synthetic | keyword | 21 | 0.6562 | 0.0710 | 0.3825 | 0.0402 | 0.1217 | 0.2000 | 0.1754 | 0.3957 | 29.7507 | 0.2535 | 0.0976 |
| E5-Mistral-7B | Synthetic | keyword | 22 | 0.6875 | 0.0956 | 0.5912 | 0.0556 | 0.1398 | 0.2400 | 0.2094 | 0.4918 | 30.2143 | 0.2525 | 0.0953 |
| E5-Mistral-7B | Synthetic | keyword | 23 | 0.7188 | 0.1084 | 0.6997 | 0.0567 | 0.1461 | 0.2300 | 0.2282 | 0.5448 | 30.2669 | 0.2593 | 0.0943 |
| E5-Mistral-7B | Synthetic | keyword | 24 | 0.7500 | 0.1077 | 0.6938 | 0.0593 | 0.1467 | 0.2300 | 0.2317 | 0.5547 | 30.3386 | 0.2669 | 0.0945 |
| E5-Mistral-7B | Synthetic | keyword | 25 | 0.7812 | 0.0963 | 0.5971 | 0.0558 | 0.1414 | 0.2600 | 0.2471 | 0.5981 | 30.3298 | 0.2704 | 0.0949 |
| E5-Mistral-7B | Synthetic | keyword | 26 | 0.8125 | 0.0835 | 0.4885 | 0.0441 | 0.1285 | 0.2200 | 0.2490 | 0.6036 | 30.4670 | 0.2760 | 0.0956 |
| E5-Mistral-7B | Synthetic | keyword | 27 | 0.8438 | 0.0915 | 0.5564 | 0.0487 | 0.1355 | 0.2200 | 0.2496 | 0.6052 | 31.0251 | 0.2915 | 0.0936 |
| E5-Mistral-7B | Synthetic | keyword | 28 | 0.8750 | 0.0885 | 0.5310 | 0.0464 | 0.1261 | 0.2100 | 0.2384 | 0.5734 | 31.2660 | 0.3164 | 0.0927 |
| E5-Mistral-7B | Synthetic | keyword | 29 | 0.9062 | 0.0881 | 0.5276 | 0.0502 | 0.1312 | 0.2300 | 0.2770 | 0.6827 | 31.6163 | 0.3291 | 0.0910 |
| E5-Mistral-7B | Synthetic | keyword | 30 | 0.9375 | 0.1340 | 0.9169 | 0.0822 | 0.1937 | 0.3100 | 0.3622 | 0.9233 | 31.2943 | 0.3890 | 0.0911 |
| E5-Mistral-7B | Synthetic | keyword | 31 | 0.9688 | 0.1438 | 1.0000 | 0.0867 | 0.2006 | 0.3300 | 0.3894 | 1.0000 | 30.8168 | 0.4386 | 0.0929 |
| E5-Mistral-7B | Synthetic | keyword | 32 | 1.0000 | 0.0959 | 0.5937 | 0.0568 | 0.1476 | 0.2200 | 0.3179 | 0.7981 | 27.8723 | 0.2624 | 0.1064 |
| E5-Mistral-7B | Synthetic | natural_language | 0 | 0.0000 | 0.1500 | 0.9656 | 0.0900 | 0.2200 | 0.1500 | 0.3789 | 0.9704 | 23.4754 | 0.0712 | 0.1434 |
| E5-Mistral-7B | Synthetic | natural_language | 1 | 0.0312 | 0.0438 | 0.1530 | 0.0158 | 0.0806 | 0.1100 | 0.0424 | 0.0202 | 25.2446 | 0.2703 | 0.1307 |
| E5-Mistral-7B | Synthetic | natural_language | 2 | 0.0625 | 0.0342 | 0.0796 | 0.0125 | 0.0640 | 0.1100 | 0.0363 | 0.0030 | 29.2636 | 0.2103 | 0.1132 |
| E5-Mistral-7B | Synthetic | natural_language | 3 | 0.0938 | 0.0323 | 0.0650 | 0.0115 | 0.0592 | 0.1000 | 0.0353 | 0.0000 | 27.6461 | 0.2260 | 0.1184 |
| E5-Mistral-7B | Synthetic | natural_language | 4 | 0.1250 | 0.0340 | 0.0780 | 0.0116 | 0.0613 | 0.1000 | 0.0430 | 0.0219 | 30.3155 | 0.2169 | 0.1080 |
| E5-Mistral-7B | Synthetic | natural_language | 5 | 0.1562 | 0.0323 | 0.0650 | 0.0112 | 0.0615 | 0.1000 | 0.0378 | 0.0071 | 31.1644 | 0.2280 | 0.1017 |
| E5-Mistral-7B | Synthetic | natural_language | 6 | 0.1875 | 0.0301 | 0.0482 | 0.0101 | 0.0565 | 0.1000 | 0.0502 | 0.0422 | 30.3212 | 0.2111 | 0.1047 |
| E5-Mistral-7B | Synthetic | natural_language | 7 | 0.2188 | 0.0301 | 0.0482 | 0.0109 | 0.0573 | 0.1000 | 0.0430 | 0.0219 | 30.0111 | 0.1880 | 0.1056 |
| E5-Mistral-7B | Synthetic | natural_language | 8 | 0.2500 | 0.0294 | 0.0428 | 0.0092 | 0.0570 | 0.1000 | 0.0492 | 0.0393 | 29.1703 | 0.2098 | 0.1063 |
| E5-Mistral-7B | Synthetic | natural_language | 9 | 0.2812 | 0.0303 | 0.0497 | 0.0107 | 0.0581 | 0.1100 | 0.0522 | 0.0479 | 28.6771 | 0.2160 | 0.1050 |
| E5-Mistral-7B | Synthetic | natural_language | 10 | 0.3125 | 0.0294 | 0.0428 | 0.0104 | 0.0550 | 0.1000 | 0.0447 | 0.0266 | 28.3744 | 0.2164 | 0.1046 |
| E5-Mistral-7B | Synthetic | natural_language | 11 | 0.3438 | 0.0293 | 0.0421 | 0.0094 | 0.0563 | 0.0900 | 0.0445 | 0.0261 | 28.6618 | 0.2366 | 0.1015 |
| E5-Mistral-7B | Synthetic | natural_language | 12 | 0.3750 | 0.0309 | 0.0543 | 0.0108 | 0.0567 | 0.1100 | 0.0452 | 0.0281 | 27.8569 | 0.2450 | 0.1061 |
| E5-Mistral-7B | Synthetic | natural_language | 13 | 0.4062 | 0.0284 | 0.0352 | 0.0088 | 0.0563 | 0.1000 | 0.0495 | 0.0403 | 27.1003 | 0.2437 | 0.1089 |
| E5-Mistral-7B | Synthetic | natural_language | 14 | 0.4375 | 0.0269 | 0.0237 | 0.0085 | 0.0564 | 0.0900 | 0.0458 | 0.0297 | 27.3985 | 0.2438 | 0.1076 |
| E5-Mistral-7B | Synthetic | natural_language | 15 | 0.4688 | 0.0238 | 0.0000 | 0.0062 | 0.0479 | 0.0800 | 0.0488 | 0.0383 | 26.8691 | 0.2561 | 0.1102 |
| E5-Mistral-7B | Synthetic | natural_language | 16 | 0.5000 | 0.0419 | 0.1385 | 0.0160 | 0.0770 | 0.1400 | 0.0586 | 0.0659 | 26.5046 | 0.2521 | 0.1107 |
| E5-Mistral-7B | Synthetic | natural_language | 17 | 0.5312 | 0.0401 | 0.1247 | 0.0122 | 0.0745 | 0.1000 | 0.0606 | 0.0714 | 26.7174 | 0.2513 | 0.1113 |
| E5-Mistral-7B | Synthetic | natural_language | 18 | 0.5625 | 0.0336 | 0.0750 | 0.0127 | 0.0629 | 0.1200 | 0.0568 | 0.0608 | 27.2241 | 0.2390 | 0.1115 |
| E5-Mistral-7B | Synthetic | natural_language | 19 | 0.5938 | 0.0452 | 0.1637 | 0.0165 | 0.0814 | 0.1300 | 0.0724 | 0.1048 | 27.9020 | 0.2340 | 0.1064 |
| E5-Mistral-7B | Synthetic | natural_language | 20 | 0.6250 | 0.0955 | 0.5486 | 0.0520 | 0.1432 | 0.2400 | 0.1302 | 0.2681 | 28.8389 | 0.2391 | 0.1029 |
| E5-Mistral-7B | Synthetic | natural_language | 21 | 0.6562 | 0.1415 | 0.9005 | 0.0909 | 0.2035 | 0.3200 | 0.1754 | 0.3957 | 29.7507 | 0.2535 | 0.0976 |
| E5-Mistral-7B | Synthetic | natural_language | 22 | 0.6875 | 0.1261 | 0.7827 | 0.0774 | 0.1802 | 0.3100 | 0.2094 | 0.4918 | 30.2143 | 0.2525 | 0.0953 |
| E5-Mistral-7B | Synthetic | natural_language | 23 | 0.7188 | 0.1524 | 0.9839 | 0.0989 | 0.2234 | 0.3000 | 0.2282 | 0.5448 | 30.2669 | 0.2593 | 0.0943 |
| E5-Mistral-7B | Synthetic | natural_language | 24 | 0.7500 | 0.1510 | 0.9732 | 0.0891 | 0.2166 | 0.2600 | 0.2317 | 0.5547 | 30.3386 | 0.2669 | 0.0945 |
| E5-Mistral-7B | Synthetic | natural_language | 25 | 0.7812 | 0.1498 | 0.9640 | 0.0906 | 0.2160 | 0.2800 | 0.2471 | 0.5981 | 30.3298 | 0.2704 | 0.0949 |
| E5-Mistral-7B | Synthetic | natural_language | 26 | 0.8125 | 0.1545 | 1.0000 | 0.0909 | 0.2113 | 0.2800 | 0.2490 | 0.6036 | 30.4670 | 0.2760 | 0.0956 |
| E5-Mistral-7B | Synthetic | natural_language | 27 | 0.8438 | 0.1346 | 0.8477 | 0.0794 | 0.1904 | 0.2800 | 0.2496 | 0.6052 | 31.0251 | 0.2915 | 0.0936 |
| E5-Mistral-7B | Synthetic | natural_language | 28 | 0.8750 | 0.1260 | 0.7819 | 0.0720 | 0.1864 | 0.2600 | 0.2384 | 0.5734 | 31.2660 | 0.3164 | 0.0927 |
| E5-Mistral-7B | Synthetic | natural_language | 29 | 0.9062 | 0.1232 | 0.7605 | 0.0704 | 0.1816 | 0.2400 | 0.2770 | 0.6827 | 31.6163 | 0.3291 | 0.0910 |
| E5-Mistral-7B | Synthetic | natural_language | 30 | 0.9375 | 0.1169 | 0.7123 | 0.0663 | 0.1724 | 0.2500 | 0.3622 | 0.9233 | 31.2943 | 0.3890 | 0.0911 |
| E5-Mistral-7B | Synthetic | natural_language | 31 | 0.9688 | 0.1163 | 0.7077 | 0.0647 | 0.1771 | 0.2400 | 0.3894 | 1.0000 | 30.8168 | 0.4386 | 0.0929 |
| E5-Mistral-7B | Synthetic | natural_language | 32 | 1.0000 | 0.0977 | 0.5654 | 0.0493 | 0.1580 | 0.1500 | 0.3179 | 0.7981 | 27.8723 | 0.2624 | 0.1064 |
| E5-Mistral-7B-ablation | MTSamples | keyword | 0 | 0.0000 | 0.2410 | 0.2930 | 0.1747 | 0.3068 | 0.5200 | 0.4300 | 0.6291 | 21.3110 | 0.8801 | 0.1540 |
| E5-Mistral-7B-ablation | MTSamples | keyword | 1 | 0.0312 | 0.0316 | 0.0030 | 0.0116 | 0.0572 | 0.1000 | 0.0858 | 0.0869 | 12.2786 | 0.9633 | 0.2087 |
| E5-Mistral-7B-ablation | MTSamples | keyword | 2 | 0.0625 | 0.0294 | 0.0000 | 0.0088 | 0.0563 | 0.1000 | 0.0306 | 0.0000 | 1.4589 | 0.9514 | 0.8265 |
| E5-Mistral-7B-ablation | MTSamples | keyword | 3 | 0.0938 | 0.0294 | 0.0000 | 0.0100 | 0.0558 | 0.1000 | 0.0310 | 0.0006 | 1.7575 | 0.9485 | 0.7505 |
| E5-Mistral-7B-ablation | MTSamples | keyword | 4 | 0.1250 | 0.0294 | 0.0000 | 0.0088 | 0.0569 | 0.1000 | 0.0309 | 0.0004 | 2.5809 | 0.9338 | 0.6098 |
| E5-Mistral-7B-ablation | MTSamples | keyword | 5 | 0.1562 | 0.0294 | 0.0000 | 0.0092 | 0.0578 | 0.1000 | 0.0308 | 0.0003 | 3.6029 | 0.9138 | 0.4981 |
| E5-Mistral-7B-ablation | MTSamples | keyword | 6 | 0.1875 | 0.0297 | 0.0004 | 0.0091 | 0.0567 | 0.1000 | 0.0385 | 0.0123 | 6.4240 | 0.8705 | 0.3226 |
| E5-Mistral-7B-ablation | MTSamples | keyword | 7 | 0.2188 | 0.0297 | 0.0004 | 0.0097 | 0.0578 | 0.1000 | 0.0361 | 0.0086 | 6.7777 | 0.8531 | 0.3044 |
| E5-Mistral-7B-ablation | MTSamples | keyword | 8 | 0.2500 | 0.0320 | 0.0036 | 0.0124 | 0.0590 | 0.1200 | 0.0354 | 0.0075 | 8.1268 | 0.8220 | 0.2462 |
| E5-Mistral-7B-ablation | MTSamples | keyword | 9 | 0.2812 | 0.0321 | 0.0037 | 0.0120 | 0.0588 | 0.1200 | 0.0386 | 0.0125 | 7.9694 | 0.8130 | 0.2455 |
| E5-Mistral-7B-ablation | MTSamples | keyword | 10 | 0.3125 | 0.0294 | 0.0000 | 0.0092 | 0.0573 | 0.1000 | 0.0364 | 0.0091 | 8.6933 | 0.8205 | 0.2432 |
| E5-Mistral-7B-ablation | MTSamples | keyword | 11 | 0.3438 | 0.0299 | 0.0007 | 0.0098 | 0.0556 | 0.1000 | 0.0356 | 0.0078 | 8.5145 | 0.8257 | 0.2388 |
| E5-Mistral-7B-ablation | MTSamples | keyword | 12 | 0.3750 | 0.0299 | 0.0007 | 0.0096 | 0.0557 | 0.1000 | 0.0371 | 0.0102 | 9.2200 | 0.8232 | 0.2251 |
| E5-Mistral-7B-ablation | MTSamples | keyword | 13 | 0.4062 | 0.0301 | 0.0010 | 0.0095 | 0.0571 | 0.1000 | 0.0364 | 0.0091 | 9.8241 | 0.8253 | 0.2189 |
| E5-Mistral-7B-ablation | MTSamples | keyword | 14 | 0.4375 | 0.0311 | 0.0024 | 0.0112 | 0.0586 | 0.1100 | 0.0422 | 0.0182 | 9.5585 | 0.8243 | 0.2259 |
| E5-Mistral-7B-ablation | MTSamples | keyword | 15 | 0.4688 | 0.0324 | 0.0042 | 0.0124 | 0.0597 | 0.1200 | 0.0415 | 0.0171 | 10.0416 | 0.8305 | 0.2320 |
| E5-Mistral-7B-ablation | MTSamples | keyword | 16 | 0.5000 | 0.0334 | 0.0055 | 0.0131 | 0.0586 | 0.1300 | 0.0490 | 0.0289 | 10.5409 | 0.8278 | 0.2388 |
| E5-Mistral-7B-ablation | MTSamples | keyword | 17 | 0.5312 | 0.0321 | 0.0037 | 0.0119 | 0.0593 | 0.1100 | 0.0487 | 0.0284 | 10.6914 | 0.8416 | 0.2491 |
| E5-Mistral-7B-ablation | MTSamples | keyword | 18 | 0.5625 | 0.0344 | 0.0069 | 0.0136 | 0.0617 | 0.1300 | 0.0494 | 0.0295 | 11.0547 | 0.8479 | 0.2450 |
| E5-Mistral-7B-ablation | MTSamples | keyword | 19 | 0.5938 | 0.0362 | 0.0094 | 0.0162 | 0.0625 | 0.1300 | 0.0599 | 0.0460 | 12.6741 | 0.8193 | 0.2273 |
| E5-Mistral-7B-ablation | MTSamples | keyword | 20 | 0.6250 | 0.0569 | 0.0381 | 0.0326 | 0.0861 | 0.2300 | 0.1230 | 0.1455 | 14.5543 | 0.8002 | 0.2124 |
| E5-Mistral-7B-ablation | MTSamples | keyword | 21 | 0.6562 | 0.0828 | 0.0739 | 0.0544 | 0.1150 | 0.3500 | 0.1987 | 0.2648 | 14.4524 | 0.7962 | 0.2164 |
| E5-Mistral-7B-ablation | MTSamples | keyword | 22 | 0.6875 | 0.1224 | 0.1288 | 0.0875 | 0.1569 | 0.4900 | 0.2781 | 0.3898 | 14.2262 | 0.7776 | 0.2176 |
| E5-Mistral-7B-ablation | MTSamples | keyword | 23 | 0.7188 | 0.1561 | 0.1754 | 0.1213 | 0.1908 | 0.6000 | 0.3308 | 0.4727 | 14.5579 | 0.7848 | 0.2155 |
| E5-Mistral-7B-ablation | MTSamples | keyword | 24 | 0.7500 | 0.1800 | 0.2085 | 0.1427 | 0.2204 | 0.6400 | 0.3296 | 0.4708 | 14.6225 | 0.7812 | 0.2151 |
| E5-Mistral-7B-ablation | MTSamples | keyword | 25 | 0.7812 | 0.2178 | 0.2608 | 0.1777 | 0.2629 | 0.7200 | 0.3774 | 0.5462 | 14.8664 | 0.7769 | 0.2138 |
| E5-Mistral-7B-ablation | MTSamples | keyword | 26 | 0.8125 | 0.2419 | 0.2942 | 0.1987 | 0.2827 | 0.7600 | 0.4106 | 0.5984 | 15.3528 | 0.7912 | 0.2098 |
| E5-Mistral-7B-ablation | MTSamples | keyword | 27 | 0.8438 | 0.2750 | 0.3400 | 0.2322 | 0.3208 | 0.8200 | 0.4418 | 0.6477 | 15.8905 | 0.8067 | 0.2055 |
| E5-Mistral-7B-ablation | MTSamples | keyword | 28 | 0.8750 | 0.2860 | 0.3553 | 0.2449 | 0.3277 | 0.8200 | 0.4362 | 0.6388 | 16.1880 | 0.8251 | 0.2038 |
| E5-Mistral-7B-ablation | MTSamples | keyword | 29 | 0.9062 | 0.3250 | 0.4092 | 0.2761 | 0.3607 | 0.8500 | 0.4788 | 0.7059 | 17.2694 | 0.8258 | 0.1958 |
| E5-Mistral-7B-ablation | MTSamples | keyword | 30 | 0.9375 | 0.3732 | 0.4760 | 0.3272 | 0.4177 | 0.8900 | 0.5370 | 0.7975 | 18.0165 | 0.8404 | 0.1906 |
| E5-Mistral-7B-ablation | MTSamples | keyword | 31 | 0.9688 | 0.4200 | 0.5408 | 0.3693 | 0.4656 | 0.9200 | 0.5834 | 0.8707 | 19.0420 | 0.8660 | 0.1829 |
| E5-Mistral-7B-ablation | MTSamples | keyword | 32 | 1.0000 | 0.7517 | 1.0000 | 0.6748 | 0.8163 | 0.8900 | 0.6655 | 1.0000 | 16.3031 | 0.7083 | 0.2051 |
| E5-Mistral-7B-ablation | MTSamples | natural_language | 0 | 0.0000 | 0.5627 | 0.9005 | 0.4739 | 0.6476 | 0.7500 | 0.4300 | 0.6291 | 21.3110 | 0.8801 | 0.1540 |
| E5-Mistral-7B-ablation | MTSamples | natural_language | 1 | 0.0312 | 0.0497 | 0.0343 | 0.0208 | 0.0832 | 0.1500 | 0.0858 | 0.0869 | 12.2786 | 0.9633 | 0.2087 |
| E5-Mistral-7B-ablation | MTSamples | natural_language | 2 | 0.0625 | 0.0294 | 0.0000 | 0.0095 | 0.0562 | 0.1000 | 0.0306 | 0.0000 | 1.4589 | 0.9514 | 0.8265 |
| E5-Mistral-7B-ablation | MTSamples | natural_language | 3 | 0.0938 | 0.0294 | 0.0000 | 0.0098 | 0.0566 | 0.1000 | 0.0310 | 0.0006 | 1.7575 | 0.9485 | 0.7505 |
| E5-Mistral-7B-ablation | MTSamples | natural_language | 4 | 0.1250 | 0.0297 | 0.0005 | 0.0097 | 0.0563 | 0.1000 | 0.0309 | 0.0004 | 2.5809 | 0.9338 | 0.6098 |
| E5-Mistral-7B-ablation | MTSamples | natural_language | 5 | 0.1562 | 0.0297 | 0.0005 | 0.0096 | 0.0563 | 0.1000 | 0.0308 | 0.0003 | 3.6029 | 0.9138 | 0.4981 |
| E5-Mistral-7B-ablation | MTSamples | natural_language | 6 | 0.1875 | 0.0335 | 0.0069 | 0.0135 | 0.0608 | 0.1200 | 0.0385 | 0.0123 | 6.4240 | 0.8705 | 0.3226 |
| E5-Mistral-7B-ablation | MTSamples | natural_language | 7 | 0.2188 | 0.0328 | 0.0057 | 0.0115 | 0.0595 | 0.1200 | 0.0361 | 0.0086 | 6.7777 | 0.8531 | 0.3044 |
| E5-Mistral-7B-ablation | MTSamples | natural_language | 8 | 0.2500 | 0.0342 | 0.0081 | 0.0140 | 0.0612 | 0.1300 | 0.0354 | 0.0075 | 8.1268 | 0.8220 | 0.2462 |
| E5-Mistral-7B-ablation | MTSamples | natural_language | 9 | 0.2812 | 0.0344 | 0.0084 | 0.0132 | 0.0622 | 0.1300 | 0.0386 | 0.0125 | 7.9694 | 0.8130 | 0.2455 |
| E5-Mistral-7B-ablation | MTSamples | natural_language | 10 | 0.3125 | 0.0330 | 0.0061 | 0.0116 | 0.0598 | 0.1200 | 0.0364 | 0.0091 | 8.6933 | 0.8205 | 0.2432 |
| E5-Mistral-7B-ablation | MTSamples | natural_language | 11 | 0.3438 | 0.0311 | 0.0029 | 0.0112 | 0.0572 | 0.1000 | 0.0356 | 0.0078 | 8.5145 | 0.8257 | 0.2388 |
| E5-Mistral-7B-ablation | MTSamples | natural_language | 12 | 0.3750 | 0.0338 | 0.0074 | 0.0120 | 0.0600 | 0.1200 | 0.0371 | 0.0102 | 9.2200 | 0.8232 | 0.2251 |
| E5-Mistral-7B-ablation | MTSamples | natural_language | 13 | 0.4062 | 0.0337 | 0.0073 | 0.0129 | 0.0603 | 0.1200 | 0.0364 | 0.0091 | 9.8241 | 0.8253 | 0.2189 |
| E5-Mistral-7B-ablation | MTSamples | natural_language | 14 | 0.4375 | 0.0344 | 0.0084 | 0.0125 | 0.0613 | 0.1200 | 0.0422 | 0.0182 | 9.5585 | 0.8243 | 0.2259 |
| E5-Mistral-7B-ablation | MTSamples | natural_language | 15 | 0.4688 | 0.0348 | 0.0091 | 0.0140 | 0.0616 | 0.1200 | 0.0415 | 0.0171 | 10.0416 | 0.8305 | 0.2320 |
| E5-Mistral-7B-ablation | MTSamples | natural_language | 16 | 0.5000 | 0.0415 | 0.0204 | 0.0196 | 0.0682 | 0.1600 | 0.0490 | 0.0289 | 10.5409 | 0.8278 | 0.2388 |
| E5-Mistral-7B-ablation | MTSamples | natural_language | 17 | 0.5312 | 0.0462 | 0.0284 | 0.0228 | 0.0749 | 0.1700 | 0.0487 | 0.0284 | 10.6914 | 0.8416 | 0.2491 |
| E5-Mistral-7B-ablation | MTSamples | natural_language | 18 | 0.5625 | 0.0458 | 0.0277 | 0.0204 | 0.0747 | 0.1500 | 0.0494 | 0.0295 | 11.0547 | 0.8479 | 0.2450 |
| E5-Mistral-7B-ablation | MTSamples | natural_language | 19 | 0.5938 | 0.0553 | 0.0437 | 0.0281 | 0.0843 | 0.1800 | 0.0599 | 0.0460 | 12.6741 | 0.8193 | 0.2273 |
| E5-Mistral-7B-ablation | MTSamples | natural_language | 20 | 0.6250 | 0.1074 | 0.1317 | 0.0654 | 0.1428 | 0.3100 | 0.1230 | 0.1455 | 14.5543 | 0.8002 | 0.2124 |
| E5-Mistral-7B-ablation | MTSamples | natural_language | 21 | 0.6562 | 0.1541 | 0.2106 | 0.1071 | 0.1956 | 0.4600 | 0.1987 | 0.2648 | 14.4524 | 0.7962 | 0.2164 |
| E5-Mistral-7B-ablation | MTSamples | natural_language | 22 | 0.6875 | 0.2207 | 0.3230 | 0.1646 | 0.2707 | 0.6000 | 0.2781 | 0.3898 | 14.2262 | 0.7776 | 0.2176 |
| E5-Mistral-7B-ablation | MTSamples | natural_language | 23 | 0.7188 | 0.2816 | 0.4259 | 0.2192 | 0.3396 | 0.7300 | 0.3308 | 0.4727 | 14.5579 | 0.7848 | 0.2155 |
| E5-Mistral-7B-ablation | MTSamples | natural_language | 24 | 0.7500 | 0.2899 | 0.4399 | 0.2247 | 0.3499 | 0.7400 | 0.3296 | 0.4708 | 14.6225 | 0.7812 | 0.2151 |
| E5-Mistral-7B-ablation | MTSamples | natural_language | 25 | 0.7812 | 0.3183 | 0.4878 | 0.2539 | 0.3772 | 0.7700 | 0.3774 | 0.5462 | 14.8664 | 0.7769 | 0.2138 |
| E5-Mistral-7B-ablation | MTSamples | natural_language | 26 | 0.8125 | 0.3477 | 0.5375 | 0.2768 | 0.4037 | 0.7800 | 0.4106 | 0.5984 | 15.3528 | 0.7912 | 0.2098 |
| E5-Mistral-7B-ablation | MTSamples | natural_language | 27 | 0.8438 | 0.4022 | 0.6295 | 0.3279 | 0.4665 | 0.8200 | 0.4418 | 0.6477 | 15.8905 | 0.8067 | 0.2055 |
| E5-Mistral-7B-ablation | MTSamples | natural_language | 28 | 0.8750 | 0.4013 | 0.6280 | 0.3303 | 0.4714 | 0.8100 | 0.4362 | 0.6388 | 16.1880 | 0.8251 | 0.2038 |
| E5-Mistral-7B-ablation | MTSamples | natural_language | 29 | 0.9062 | 0.4593 | 0.7259 | 0.3830 | 0.5280 | 0.8400 | 0.4788 | 0.7059 | 17.2694 | 0.8258 | 0.1958 |
| E5-Mistral-7B-ablation | MTSamples | natural_language | 30 | 0.9375 | 0.5346 | 0.8531 | 0.4539 | 0.6058 | 0.8700 | 0.5370 | 0.7975 | 18.0165 | 0.8404 | 0.1906 |
| E5-Mistral-7B-ablation | MTSamples | natural_language | 31 | 0.9688 | 0.6216 | 1.0000 | 0.5421 | 0.6939 | 0.8900 | 0.5834 | 0.8707 | 19.0420 | 0.8660 | 0.1829 |
| E5-Mistral-7B-ablation | MTSamples | natural_language | 32 | 1.0000 | 0.5559 | 0.8891 | 0.4723 | 0.6354 | 0.8500 | 0.6655 | 1.0000 | 16.3031 | 0.7083 | 0.2051 |
| E5-Mistral-7B-ablation | PMC-Patients | keyword | 0 | 0.0000 | 0.3000 | 0.3797 | 0.2166 | 0.3861 | 0.4800 | 0.4300 | 0.6291 | 19.4850 | 0.8902 | 0.1916 |
| E5-Mistral-7B-ablation | PMC-Patients | keyword | 1 | 0.0312 | 0.0343 | 0.0072 | 0.0105 | 0.0632 | 0.1000 | 0.0858 | 0.0869 | 12.6032 | 0.9682 | 0.2098 |
| E5-Mistral-7B-ablation | PMC-Patients | keyword | 2 | 0.0625 | 0.0293 | 0.0001 | 0.0091 | 0.0555 | 0.1000 | 0.0306 | 0.0000 | 2.3104 | 0.9751 | 0.5365 |
| E5-Mistral-7B-ablation | PMC-Patients | keyword | 3 | 0.0938 | 0.0293 | 0.0001 | 0.0104 | 0.0580 | 0.1000 | 0.0310 | 0.0006 | 2.3265 | 0.9714 | 0.5962 |
| E5-Mistral-7B-ablation | PMC-Patients | keyword | 4 | 0.1250 | 0.0292 | 0.0000 | 0.0091 | 0.0573 | 0.1000 | 0.0309 | 0.0004 | 2.7989 | 0.9629 | 0.5645 |
| E5-Mistral-7B-ablation | PMC-Patients | keyword | 5 | 0.1562 | 0.0297 | 0.0007 | 0.0095 | 0.0577 | 0.1000 | 0.0308 | 0.0003 | 3.3469 | 0.9487 | 0.5263 |
| E5-Mistral-7B-ablation | PMC-Patients | keyword | 6 | 0.1875 | 0.0320 | 0.0039 | 0.0098 | 0.0587 | 0.1000 | 0.0385 | 0.0123 | 5.3382 | 0.9206 | 0.4124 |
| E5-Mistral-7B-ablation | PMC-Patients | keyword | 7 | 0.2188 | 0.0311 | 0.0027 | 0.0108 | 0.0585 | 0.1000 | 0.0361 | 0.0086 | 5.6998 | 0.9045 | 0.3986 |
| E5-Mistral-7B-ablation | PMC-Patients | keyword | 8 | 0.2500 | 0.0320 | 0.0039 | 0.0112 | 0.0596 | 0.1000 | 0.0354 | 0.0075 | 7.8369 | 0.8806 | 0.3307 |
| E5-Mistral-7B-ablation | PMC-Patients | keyword | 9 | 0.2812 | 0.0343 | 0.0072 | 0.0119 | 0.0599 | 0.1200 | 0.0386 | 0.0125 | 8.1314 | 0.8690 | 0.3235 |
| E5-Mistral-7B-ablation | PMC-Patients | keyword | 10 | 0.3125 | 0.0327 | 0.0049 | 0.0110 | 0.0618 | 0.1100 | 0.0364 | 0.0091 | 8.3220 | 0.8631 | 0.3196 |
| E5-Mistral-7B-ablation | PMC-Patients | keyword | 11 | 0.3438 | 0.0331 | 0.0055 | 0.0120 | 0.0605 | 0.1200 | 0.0356 | 0.0078 | 8.4544 | 0.8587 | 0.3174 |
| E5-Mistral-7B-ablation | PMC-Patients | keyword | 12 | 0.3750 | 0.0343 | 0.0072 | 0.0129 | 0.0616 | 0.1200 | 0.0371 | 0.0102 | 9.3845 | 0.8604 | 0.2968 |
| E5-Mistral-7B-ablation | PMC-Patients | keyword | 13 | 0.4062 | 0.0317 | 0.0035 | 0.0106 | 0.0578 | 0.1000 | 0.0364 | 0.0091 | 9.6973 | 0.8562 | 0.2901 |
| E5-Mistral-7B-ablation | PMC-Patients | keyword | 14 | 0.4375 | 0.0356 | 0.0090 | 0.0140 | 0.0643 | 0.1300 | 0.0422 | 0.0182 | 9.8647 | 0.8567 | 0.2862 |
| E5-Mistral-7B-ablation | PMC-Patients | keyword | 15 | 0.4688 | 0.0375 | 0.0116 | 0.0153 | 0.0658 | 0.1200 | 0.0415 | 0.0171 | 11.5786 | 0.8701 | 0.2561 |
| E5-Mistral-7B-ablation | PMC-Patients | keyword | 16 | 0.5000 | 0.0378 | 0.0121 | 0.0149 | 0.0672 | 0.1200 | 0.0490 | 0.0289 | 15.1552 | 0.8622 | 0.2073 |
| E5-Mistral-7B-ablation | PMC-Patients | keyword | 17 | 0.5312 | 0.0393 | 0.0142 | 0.0140 | 0.0693 | 0.1200 | 0.0487 | 0.0284 | 17.5074 | 0.8716 | 0.1809 |
| E5-Mistral-7B-ablation | PMC-Patients | keyword | 18 | 0.5625 | 0.0399 | 0.0150 | 0.0154 | 0.0673 | 0.1200 | 0.0494 | 0.0295 | 18.8218 | 0.8805 | 0.1689 |
| E5-Mistral-7B-ablation | PMC-Patients | keyword | 19 | 0.5938 | 0.0459 | 0.0234 | 0.0166 | 0.0818 | 0.1200 | 0.0599 | 0.0460 | 23.7634 | 0.8420 | 0.1343 |
| E5-Mistral-7B-ablation | PMC-Patients | keyword | 20 | 0.6250 | 0.0687 | 0.0554 | 0.0343 | 0.1137 | 0.1900 | 0.1230 | 0.1455 | 28.4043 | 0.8088 | 0.1060 |
| E5-Mistral-7B-ablation | PMC-Patients | keyword | 21 | 0.6562 | 0.0892 | 0.0841 | 0.0477 | 0.1370 | 0.2400 | 0.1987 | 0.2648 | 29.1111 | 0.7970 | 0.1042 |
| E5-Mistral-7B-ablation | PMC-Patients | keyword | 22 | 0.6875 | 0.1544 | 0.1755 | 0.1006 | 0.2163 | 0.4200 | 0.2781 | 0.3898 | 29.2735 | 0.7758 | 0.0999 |
| E5-Mistral-7B-ablation | PMC-Patients | keyword | 23 | 0.7188 | 0.2050 | 0.2465 | 0.1406 | 0.2688 | 0.5100 | 0.3308 | 0.4727 | 30.0667 | 0.7859 | 0.0965 |
| E5-Mistral-7B-ablation | PMC-Patients | keyword | 24 | 0.7500 | 0.2193 | 0.2665 | 0.1566 | 0.2907 | 0.5600 | 0.3296 | 0.4708 | 30.4945 | 0.7814 | 0.0956 |
| E5-Mistral-7B-ablation | PMC-Patients | keyword | 25 | 0.7812 | 0.2628 | 0.3275 | 0.1965 | 0.3347 | 0.5700 | 0.3774 | 0.5462 | 31.3660 | 0.7836 | 0.0927 |
| E5-Mistral-7B-ablation | PMC-Patients | keyword | 26 | 0.8125 | 0.3039 | 0.3852 | 0.2304 | 0.3781 | 0.6400 | 0.4106 | 0.5984 | 31.9529 | 0.7984 | 0.0915 |
| E5-Mistral-7B-ablation | PMC-Patients | keyword | 27 | 0.8438 | 0.3630 | 0.4680 | 0.2872 | 0.4436 | 0.6800 | 0.4418 | 0.6477 | 32.5438 | 0.8133 | 0.0896 |
| E5-Mistral-7B-ablation | PMC-Patients | keyword | 28 | 0.8750 | 0.3341 | 0.4275 | 0.2572 | 0.4098 | 0.6600 | 0.4362 | 0.6388 | 33.0713 | 0.8303 | 0.0887 |
| E5-Mistral-7B-ablation | PMC-Patients | keyword | 29 | 0.9062 | 0.3607 | 0.4648 | 0.2805 | 0.4446 | 0.6900 | 0.4788 | 0.7059 | 34.2273 | 0.8337 | 0.0867 |
| E5-Mistral-7B-ablation | PMC-Patients | keyword | 30 | 0.9375 | 0.4366 | 0.5712 | 0.3570 | 0.5189 | 0.7900 | 0.5370 | 0.7975 | 34.6408 | 0.8543 | 0.0884 |
| E5-Mistral-7B-ablation | PMC-Patients | keyword | 31 | 0.9688 | 0.5207 | 0.6891 | 0.4390 | 0.6039 | 0.8400 | 0.5834 | 0.8707 | 35.2428 | 0.8794 | 0.0885 |
| E5-Mistral-7B-ablation | PMC-Patients | keyword | 32 | 1.0000 | 0.7424 | 1.0000 | 0.6698 | 0.8103 | 0.9100 | 0.6655 | 1.0000 | 29.7713 | 0.7240 | 0.1100 |
| E5-Mistral-7B-ablation | PMC-Patients | natural_language | 0 | 0.0000 | 0.5536 | 0.7189 | 0.4648 | 0.6372 | 0.7300 | 0.4300 | 0.6291 | 19.4850 | 0.8902 | 0.1916 |
| E5-Mistral-7B-ablation | PMC-Patients | natural_language | 1 | 0.0312 | 0.0450 | 0.0215 | 0.0150 | 0.0802 | 0.1000 | 0.0858 | 0.0869 | 12.6032 | 0.9682 | 0.2098 |
| E5-Mistral-7B-ablation | PMC-Patients | natural_language | 2 | 0.0625 | 0.0293 | 0.0000 | 0.0090 | 0.0547 | 0.1000 | 0.0306 | 0.0000 | 2.3104 | 0.9751 | 0.5365 |
| E5-Mistral-7B-ablation | PMC-Patients | natural_language | 3 | 0.0938 | 0.0310 | 0.0023 | 0.0104 | 0.0612 | 0.1000 | 0.0310 | 0.0006 | 2.3265 | 0.9714 | 0.5962 |
| E5-Mistral-7B-ablation | PMC-Patients | natural_language | 4 | 0.1250 | 0.0301 | 0.0011 | 0.0102 | 0.0557 | 0.1100 | 0.0309 | 0.0004 | 2.7989 | 0.9629 | 0.5645 |
| E5-Mistral-7B-ablation | PMC-Patients | natural_language | 5 | 0.1562 | 0.0319 | 0.0036 | 0.0106 | 0.0595 | 0.1000 | 0.0308 | 0.0003 | 3.3469 | 0.9487 | 0.5263 |
| E5-Mistral-7B-ablation | PMC-Patients | natural_language | 6 | 0.1875 | 0.0332 | 0.0053 | 0.0112 | 0.0625 | 0.1000 | 0.0385 | 0.0123 | 5.3382 | 0.9206 | 0.4124 |
| E5-Mistral-7B-ablation | PMC-Patients | natural_language | 7 | 0.2188 | 0.0368 | 0.0103 | 0.0133 | 0.0675 | 0.1000 | 0.0361 | 0.0086 | 5.6998 | 0.9045 | 0.3986 |
| E5-Mistral-7B-ablation | PMC-Patients | natural_language | 8 | 0.2500 | 0.0398 | 0.0144 | 0.0165 | 0.0719 | 0.1100 | 0.0354 | 0.0075 | 7.8369 | 0.8806 | 0.3307 |
| E5-Mistral-7B-ablation | PMC-Patients | natural_language | 9 | 0.2812 | 0.0516 | 0.0306 | 0.0184 | 0.0889 | 0.1200 | 0.0386 | 0.0125 | 8.1314 | 0.8690 | 0.3235 |
| E5-Mistral-7B-ablation | PMC-Patients | natural_language | 10 | 0.3125 | 0.0494 | 0.0276 | 0.0182 | 0.0882 | 0.1200 | 0.0364 | 0.0091 | 8.3220 | 0.8631 | 0.3196 |
| E5-Mistral-7B-ablation | PMC-Patients | natural_language | 11 | 0.3438 | 0.0486 | 0.0265 | 0.0186 | 0.0877 | 0.1200 | 0.0356 | 0.0078 | 8.4544 | 0.8587 | 0.3174 |
| E5-Mistral-7B-ablation | PMC-Patients | natural_language | 12 | 0.3750 | 0.0490 | 0.0270 | 0.0183 | 0.0891 | 0.1200 | 0.0371 | 0.0102 | 9.3845 | 0.8604 | 0.2968 |
| E5-Mistral-7B-ablation | PMC-Patients | natural_language | 13 | 0.4062 | 0.0499 | 0.0282 | 0.0179 | 0.0905 | 0.1300 | 0.0364 | 0.0091 | 9.6973 | 0.8562 | 0.2901 |
| E5-Mistral-7B-ablation | PMC-Patients | natural_language | 14 | 0.4375 | 0.0503 | 0.0288 | 0.0189 | 0.0899 | 0.1300 | 0.0422 | 0.0182 | 9.8647 | 0.8567 | 0.2862 |
| E5-Mistral-7B-ablation | PMC-Patients | natural_language | 15 | 0.4688 | 0.0482 | 0.0259 | 0.0179 | 0.0881 | 0.1200 | 0.0415 | 0.0171 | 11.5786 | 0.8701 | 0.2561 |
| E5-Mistral-7B-ablation | PMC-Patients | natural_language | 16 | 0.5000 | 0.0524 | 0.0317 | 0.0209 | 0.0916 | 0.1400 | 0.0490 | 0.0289 | 15.1552 | 0.8622 | 0.2073 |
| E5-Mistral-7B-ablation | PMC-Patients | natural_language | 17 | 0.5312 | 0.0577 | 0.0389 | 0.0256 | 0.0966 | 0.1600 | 0.0487 | 0.0284 | 17.5074 | 0.8716 | 0.1809 |
| E5-Mistral-7B-ablation | PMC-Patients | natural_language | 18 | 0.5625 | 0.0621 | 0.0450 | 0.0294 | 0.1025 | 0.1800 | 0.0494 | 0.0295 | 18.8218 | 0.8805 | 0.1689 |
| E5-Mistral-7B-ablation | PMC-Patients | natural_language | 19 | 0.5938 | 0.0741 | 0.0614 | 0.0369 | 0.1135 | 0.2100 | 0.0599 | 0.0460 | 23.7634 | 0.8420 | 0.1343 |
| E5-Mistral-7B-ablation | PMC-Patients | natural_language | 20 | 0.6250 | 0.1301 | 0.1382 | 0.0759 | 0.1936 | 0.2700 | 0.1230 | 0.1455 | 28.4043 | 0.8088 | 0.1060 |
| E5-Mistral-7B-ablation | PMC-Patients | natural_language | 21 | 0.6562 | 0.1989 | 0.2326 | 0.1371 | 0.2640 | 0.4600 | 0.1987 | 0.2648 | 29.1111 | 0.7970 | 0.1042 |
| E5-Mistral-7B-ablation | PMC-Patients | natural_language | 22 | 0.6875 | 0.2963 | 0.3661 | 0.2231 | 0.3754 | 0.5900 | 0.2781 | 0.3898 | 29.2735 | 0.7758 | 0.0999 |
| E5-Mistral-7B-ablation | PMC-Patients | natural_language | 23 | 0.7188 | 0.3492 | 0.4386 | 0.2746 | 0.4304 | 0.6700 | 0.3308 | 0.4727 | 30.0667 | 0.7859 | 0.0965 |
| E5-Mistral-7B-ablation | PMC-Patients | natural_language | 24 | 0.7500 | 0.3457 | 0.4338 | 0.2703 | 0.4242 | 0.6900 | 0.3296 | 0.4708 | 30.4945 | 0.7814 | 0.0956 |
| E5-Mistral-7B-ablation | PMC-Patients | natural_language | 25 | 0.7812 | 0.4222 | 0.5387 | 0.3348 | 0.4953 | 0.7400 | 0.3774 | 0.5462 | 31.3660 | 0.7836 | 0.0927 |
| E5-Mistral-7B-ablation | PMC-Patients | natural_language | 26 | 0.8125 | 0.4737 | 0.6094 | 0.3803 | 0.5499 | 0.7900 | 0.4106 | 0.5984 | 31.9529 | 0.7984 | 0.0915 |
| E5-Mistral-7B-ablation | PMC-Patients | natural_language | 27 | 0.8438 | 0.5050 | 0.6523 | 0.4255 | 0.5860 | 0.8400 | 0.4418 | 0.6477 | 32.5438 | 0.8133 | 0.0896 |
| E5-Mistral-7B-ablation | PMC-Patients | natural_language | 28 | 0.8750 | 0.4742 | 0.6100 | 0.3948 | 0.5595 | 0.8000 | 0.4362 | 0.6388 | 33.0713 | 0.8303 | 0.0887 |
| E5-Mistral-7B-ablation | PMC-Patients | natural_language | 29 | 0.9062 | 0.5064 | 0.6542 | 0.4235 | 0.5883 | 0.8200 | 0.4788 | 0.7059 | 34.2273 | 0.8337 | 0.0867 |
| E5-Mistral-7B-ablation | PMC-Patients | natural_language | 30 | 0.9375 | 0.5521 | 0.7169 | 0.4736 | 0.6295 | 0.8400 | 0.5370 | 0.7975 | 34.6408 | 0.8543 | 0.0884 |
| E5-Mistral-7B-ablation | PMC-Patients | natural_language | 31 | 0.9688 | 0.6055 | 0.7901 | 0.5294 | 0.6823 | 0.8700 | 0.5834 | 0.8707 | 35.2428 | 0.8794 | 0.0885 |
| E5-Mistral-7B-ablation | PMC-Patients | natural_language | 32 | 1.0000 | 0.7586 | 1.0000 | 0.6880 | 0.8253 | 0.9400 | 0.6655 | 1.0000 | 29.7713 | 0.7240 | 0.1100 |
| E5-Mistral-7B-ablation | Synthetic | keyword | 0 | 0.0000 | 0.3592 | 0.5010 | 0.2809 | 0.4390 | 0.6000 | 0.4300 | 0.6291 | 13.4812 | 0.9306 | 0.2370 |
| E5-Mistral-7B-ablation | Synthetic | keyword | 1 | 0.0312 | 0.1390 | 0.1639 | 0.0807 | 0.1869 | 0.3300 | 0.0858 | 0.0869 | 9.6666 | 0.9845 | 0.2863 |
| E5-Mistral-7B-ablation | Synthetic | keyword | 2 | 0.0625 | 0.0324 | 0.0008 | 0.0124 | 0.0616 | 0.1100 | 0.0306 | 0.0000 | 11.8897 | 0.9992 | 0.2471 |
| E5-Mistral-7B-ablation | Synthetic | keyword | 3 | 0.0938 | 0.0323 | 0.0006 | 0.0117 | 0.0609 | 0.1000 | 0.0310 | 0.0006 | 13.4806 | 0.9984 | 0.2243 |
| E5-Mistral-7B-ablation | Synthetic | keyword | 4 | 0.1250 | 0.0340 | 0.0032 | 0.0130 | 0.0619 | 0.1200 | 0.0309 | 0.0004 | 14.3315 | 0.9964 | 0.2077 |
| E5-Mistral-7B-ablation | Synthetic | keyword | 5 | 0.1562 | 0.0319 | 0.0000 | 0.0110 | 0.0600 | 0.1000 | 0.0308 | 0.0003 | 13.9203 | 0.9929 | 0.2069 |
| E5-Mistral-7B-ablation | Synthetic | keyword | 6 | 0.1875 | 0.0480 | 0.0246 | 0.0257 | 0.0749 | 0.2400 | 0.0385 | 0.0123 | 14.7980 | 0.9807 | 0.1698 |
| E5-Mistral-7B-ablation | Synthetic | keyword | 7 | 0.2188 | 0.0365 | 0.0070 | 0.0153 | 0.0629 | 0.1400 | 0.0361 | 0.0086 | 14.9234 | 0.9753 | 0.1763 |
| E5-Mistral-7B-ablation | Synthetic | keyword | 8 | 0.2500 | 0.0336 | 0.0026 | 0.0123 | 0.0613 | 0.1200 | 0.0354 | 0.0075 | 14.2481 | 0.9645 | 0.1722 |
| E5-Mistral-7B-ablation | Synthetic | keyword | 9 | 0.2812 | 0.0370 | 0.0078 | 0.0147 | 0.0640 | 0.1500 | 0.0386 | 0.0125 | 13.6333 | 0.9631 | 0.1917 |
| E5-Mistral-7B-ablation | Synthetic | keyword | 10 | 0.3125 | 0.0360 | 0.0063 | 0.0142 | 0.0632 | 0.1400 | 0.0364 | 0.0091 | 13.3643 | 0.9602 | 0.2014 |
| E5-Mistral-7B-ablation | Synthetic | keyword | 11 | 0.3438 | 0.0340 | 0.0032 | 0.0125 | 0.0626 | 0.1200 | 0.0356 | 0.0078 | 11.3121 | 0.9592 | 0.2354 |
| E5-Mistral-7B-ablation | Synthetic | keyword | 12 | 0.3750 | 0.0343 | 0.0037 | 0.0133 | 0.0614 | 0.1200 | 0.0371 | 0.0102 | 10.0945 | 0.9553 | 0.2618 |
| E5-Mistral-7B-ablation | Synthetic | keyword | 13 | 0.4062 | 0.0337 | 0.0028 | 0.0111 | 0.0629 | 0.1100 | 0.0364 | 0.0091 | 10.5036 | 0.9534 | 0.2542 |
| E5-Mistral-7B-ablation | Synthetic | keyword | 14 | 0.4375 | 0.0464 | 0.0222 | 0.0240 | 0.0748 | 0.2100 | 0.0422 | 0.0182 | 10.6584 | 0.9558 | 0.2520 |
| E5-Mistral-7B-ablation | Synthetic | keyword | 15 | 0.4688 | 0.0431 | 0.0171 | 0.0205 | 0.0700 | 0.1700 | 0.0415 | 0.0171 | 11.3650 | 0.9585 | 0.2404 |
| E5-Mistral-7B-ablation | Synthetic | keyword | 16 | 0.5000 | 0.0533 | 0.0328 | 0.0263 | 0.0884 | 0.1800 | 0.0490 | 0.0289 | 13.4469 | 0.9437 | 0.1968 |
| E5-Mistral-7B-ablation | Synthetic | keyword | 17 | 0.5312 | 0.0508 | 0.0289 | 0.0282 | 0.0827 | 0.2300 | 0.0487 | 0.0284 | 14.6840 | 0.9408 | 0.1749 |
| E5-Mistral-7B-ablation | Synthetic | keyword | 18 | 0.5625 | 0.0493 | 0.0266 | 0.0263 | 0.0805 | 0.2000 | 0.0494 | 0.0295 | 14.8034 | 0.9427 | 0.1767 |
| E5-Mistral-7B-ablation | Synthetic | keyword | 19 | 0.5938 | 0.0527 | 0.0318 | 0.0309 | 0.0920 | 0.2100 | 0.0599 | 0.0460 | 15.1005 | 0.9166 | 0.1657 |
| E5-Mistral-7B-ablation | Synthetic | keyword | 20 | 0.6250 | 0.1587 | 0.1941 | 0.1018 | 0.2205 | 0.4300 | 0.1230 | 0.1455 | 16.5089 | 0.8827 | 0.1384 |
| E5-Mistral-7B-ablation | Synthetic | keyword | 21 | 0.6562 | 0.3130 | 0.4303 | 0.2417 | 0.3776 | 0.7300 | 0.1987 | 0.2648 | 16.0214 | 0.8724 | 0.1434 |
| E5-Mistral-7B-ablation | Synthetic | keyword | 22 | 0.6875 | 0.4372 | 0.6204 | 0.3587 | 0.5120 | 0.8100 | 0.2781 | 0.3898 | 16.1184 | 0.8545 | 0.1385 |
| E5-Mistral-7B-ablation | Synthetic | keyword | 23 | 0.7188 | 0.5035 | 0.7219 | 0.4243 | 0.5752 | 0.9000 | 0.3308 | 0.4727 | 15.9346 | 0.8574 | 0.1421 |
| E5-Mistral-7B-ablation | Synthetic | keyword | 24 | 0.7500 | 0.4881 | 0.6983 | 0.3988 | 0.5504 | 0.8700 | 0.3296 | 0.4708 | 15.6633 | 0.8572 | 0.1492 |
| E5-Mistral-7B-ablation | Synthetic | keyword | 25 | 0.7812 | 0.5491 | 0.7917 | 0.4629 | 0.6232 | 0.9000 | 0.3774 | 0.5462 | 15.7777 | 0.8586 | 0.1495 |
| E5-Mistral-7B-ablation | Synthetic | keyword | 26 | 0.8125 | 0.5770 | 0.8344 | 0.4880 | 0.6352 | 0.9100 | 0.4106 | 0.5984 | 15.8954 | 0.8658 | 0.1484 |
| E5-Mistral-7B-ablation | Synthetic | keyword | 27 | 0.8438 | 0.5608 | 0.8096 | 0.4798 | 0.6297 | 0.9000 | 0.4418 | 0.6477 | 15.9248 | 0.8765 | 0.1493 |
| E5-Mistral-7B-ablation | Synthetic | keyword | 28 | 0.8750 | 0.5751 | 0.8315 | 0.4970 | 0.6412 | 0.9100 | 0.4362 | 0.6388 | 16.0609 | 0.8921 | 0.1506 |
| E5-Mistral-7B-ablation | Synthetic | keyword | 29 | 0.9062 | 0.6165 | 0.8948 | 0.5403 | 0.6888 | 0.9200 | 0.4788 | 0.7059 | 16.3310 | 0.8973 | 0.1497 |
| E5-Mistral-7B-ablation | Synthetic | keyword | 30 | 0.9375 | 0.6757 | 0.9855 | 0.5967 | 0.7400 | 0.9400 | 0.5370 | 0.7975 | 16.7481 | 0.9142 | 0.1430 |
| E5-Mistral-7B-ablation | Synthetic | keyword | 31 | 0.9688 | 0.6852 | 1.0000 | 0.6040 | 0.7451 | 0.9400 | 0.5834 | 0.8707 | 17.0740 | 0.9288 | 0.1384 |
| E5-Mistral-7B-ablation | Synthetic | keyword | 32 | 1.0000 | 0.6559 | 0.9552 | 0.5833 | 0.7307 | 0.9400 | 0.6655 | 1.0000 | 17.3666 | 0.8216 | 0.1322 |
| E5-Mistral-7B-ablation | Synthetic | natural_language | 0 | 0.0000 | 0.5636 | 0.8607 | 0.4789 | 0.6441 | 0.8400 | 0.4300 | 0.6291 | 13.4812 | 0.9306 | 0.2370 |
| E5-Mistral-7B-ablation | Synthetic | natural_language | 1 | 0.0312 | 0.2154 | 0.2967 | 0.1651 | 0.2737 | 0.6600 | 0.0858 | 0.0869 | 9.6666 | 0.9845 | 0.2863 |
| E5-Mistral-7B-ablation | Synthetic | natural_language | 2 | 0.0625 | 0.0340 | 0.0029 | 0.0134 | 0.0620 | 0.1200 | 0.0306 | 0.0000 | 11.8897 | 0.9992 | 0.2471 |
| E5-Mistral-7B-ablation | Synthetic | natural_language | 3 | 0.0938 | 0.0346 | 0.0039 | 0.0137 | 0.0636 | 0.1300 | 0.0310 | 0.0006 | 13.4806 | 0.9984 | 0.2243 |
| E5-Mistral-7B-ablation | Synthetic | natural_language | 4 | 0.1250 | 0.0330 | 0.0013 | 0.0114 | 0.0608 | 0.1100 | 0.0309 | 0.0004 | 14.3315 | 0.9964 | 0.2077 |
| E5-Mistral-7B-ablation | Synthetic | natural_language | 5 | 0.1562 | 0.0322 | 0.0000 | 0.0109 | 0.0603 | 0.1000 | 0.0308 | 0.0003 | 13.9203 | 0.9929 | 0.2069 |
| E5-Mistral-7B-ablation | Synthetic | natural_language | 6 | 0.1875 | 0.0543 | 0.0358 | 0.0324 | 0.0842 | 0.2700 | 0.0385 | 0.0123 | 14.7980 | 0.9807 | 0.1698 |
| E5-Mistral-7B-ablation | Synthetic | natural_language | 7 | 0.2188 | 0.0496 | 0.0282 | 0.0271 | 0.0773 | 0.2400 | 0.0361 | 0.0086 | 14.9234 | 0.9753 | 0.1763 |
| E5-Mistral-7B-ablation | Synthetic | natural_language | 8 | 0.2500 | 0.0408 | 0.0139 | 0.0186 | 0.0701 | 0.1700 | 0.0354 | 0.0075 | 14.2481 | 0.9645 | 0.1722 |
| E5-Mistral-7B-ablation | Synthetic | natural_language | 9 | 0.2812 | 0.0420 | 0.0159 | 0.0217 | 0.0709 | 0.1900 | 0.0386 | 0.0125 | 13.6333 | 0.9631 | 0.1917 |
| E5-Mistral-7B-ablation | Synthetic | natural_language | 10 | 0.3125 | 0.0380 | 0.0094 | 0.0166 | 0.0650 | 0.1500 | 0.0364 | 0.0091 | 13.3643 | 0.9602 | 0.2014 |
| E5-Mistral-7B-ablation | Synthetic | natural_language | 11 | 0.3438 | 0.0368 | 0.0075 | 0.0156 | 0.0651 | 0.1500 | 0.0356 | 0.0078 | 11.3121 | 0.9592 | 0.2354 |
| E5-Mistral-7B-ablation | Synthetic | natural_language | 12 | 0.3750 | 0.0414 | 0.0149 | 0.0191 | 0.0691 | 0.1700 | 0.0371 | 0.0102 | 10.0945 | 0.9553 | 0.2618 |
| E5-Mistral-7B-ablation | Synthetic | natural_language | 13 | 0.4062 | 0.0393 | 0.0115 | 0.0171 | 0.0679 | 0.1500 | 0.0364 | 0.0091 | 10.5036 | 0.9534 | 0.2542 |
| E5-Mistral-7B-ablation | Synthetic | natural_language | 14 | 0.4375 | 0.0553 | 0.0374 | 0.0305 | 0.0833 | 0.2500 | 0.0422 | 0.0182 | 10.6584 | 0.9558 | 0.2520 |
| E5-Mistral-7B-ablation | Synthetic | natural_language | 15 | 0.4688 | 0.0531 | 0.0339 | 0.0293 | 0.0829 | 0.2200 | 0.0415 | 0.0171 | 11.3650 | 0.9585 | 0.2404 |
| E5-Mistral-7B-ablation | Synthetic | natural_language | 16 | 0.5000 | 0.0753 | 0.0698 | 0.0433 | 0.1171 | 0.2400 | 0.0490 | 0.0289 | 13.4469 | 0.9437 | 0.1968 |
| E5-Mistral-7B-ablation | Synthetic | natural_language | 17 | 0.5312 | 0.0659 | 0.0546 | 0.0373 | 0.1003 | 0.2400 | 0.0487 | 0.0284 | 14.6840 | 0.9408 | 0.1749 |
| E5-Mistral-7B-ablation | Synthetic | natural_language | 18 | 0.5625 | 0.0648 | 0.0528 | 0.0368 | 0.0982 | 0.2400 | 0.0494 | 0.0295 | 14.8034 | 0.9427 | 0.1767 |
| E5-Mistral-7B-ablation | Synthetic | natural_language | 19 | 0.5938 | 0.0950 | 0.1017 | 0.0547 | 0.1405 | 0.2800 | 0.0599 | 0.0460 | 15.1005 | 0.9166 | 0.1657 |
| E5-Mistral-7B-ablation | Synthetic | natural_language | 20 | 0.6250 | 0.2162 | 0.2980 | 0.1533 | 0.2785 | 0.5500 | 0.1230 | 0.1455 | 16.5089 | 0.8827 | 0.1384 |
| E5-Mistral-7B-ablation | Synthetic | natural_language | 21 | 0.6562 | 0.3543 | 0.5217 | 0.2904 | 0.4349 | 0.7800 | 0.1987 | 0.2648 | 16.0214 | 0.8724 | 0.1434 |
| E5-Mistral-7B-ablation | Synthetic | natural_language | 22 | 0.6875 | 0.4377 | 0.6568 | 0.3691 | 0.5176 | 0.8600 | 0.2781 | 0.3898 | 16.1184 | 0.8545 | 0.1385 |
| E5-Mistral-7B-ablation | Synthetic | natural_language | 23 | 0.7188 | 0.4891 | 0.7400 | 0.4255 | 0.5729 | 0.9100 | 0.3308 | 0.4727 | 15.9346 | 0.8574 | 0.1421 |
| E5-Mistral-7B-ablation | Synthetic | natural_language | 24 | 0.7500 | 0.4543 | 0.6837 | 0.3888 | 0.5356 | 0.9000 | 0.3296 | 0.4708 | 15.6633 | 0.8572 | 0.1492 |
| E5-Mistral-7B-ablation | Synthetic | natural_language | 25 | 0.7812 | 0.4943 | 0.7485 | 0.4250 | 0.5799 | 0.9100 | 0.3774 | 0.5462 | 15.7777 | 0.8586 | 0.1495 |
| E5-Mistral-7B-ablation | Synthetic | natural_language | 26 | 0.8125 | 0.5192 | 0.7888 | 0.4463 | 0.6018 | 0.9000 | 0.4106 | 0.5984 | 15.8954 | 0.8658 | 0.1484 |
| E5-Mistral-7B-ablation | Synthetic | natural_language | 27 | 0.8438 | 0.5449 | 0.8304 | 0.4688 | 0.6216 | 0.9000 | 0.4418 | 0.6477 | 15.9248 | 0.8765 | 0.1493 |
| E5-Mistral-7B-ablation | Synthetic | natural_language | 28 | 0.8750 | 0.5464 | 0.8328 | 0.4754 | 0.6246 | 0.9100 | 0.4362 | 0.6388 | 16.0609 | 0.8921 | 0.1506 |
| E5-Mistral-7B-ablation | Synthetic | natural_language | 29 | 0.9062 | 0.6049 | 0.9276 | 0.5314 | 0.6794 | 0.9100 | 0.4788 | 0.7059 | 16.3310 | 0.8973 | 0.1497 |
| E5-Mistral-7B-ablation | Synthetic | natural_language | 30 | 0.9375 | 0.6496 | 1.0000 | 0.5721 | 0.7191 | 0.9200 | 0.5370 | 0.7975 | 16.7481 | 0.9142 | 0.1430 |
| E5-Mistral-7B-ablation | Synthetic | natural_language | 31 | 0.9688 | 0.6474 | 0.9964 | 0.5716 | 0.7256 | 0.9100 | 0.5834 | 0.8707 | 17.0740 | 0.9288 | 0.1384 |
| E5-Mistral-7B-ablation | Synthetic | natural_language | 32 | 1.0000 | 0.5285 | 0.8039 | 0.4493 | 0.5997 | 0.8400 | 0.6655 | 1.0000 | 17.3666 | 0.8216 | 0.1322 |
| GTE-base | MTSamples | keyword | 0 | 0.0000 | 0.5891 | 0.6132 | 0.5092 | 0.6671 | 0.8400 | 0.3944 | 0.3965 | 21.1071 | 0.8926 | 0.1276 |
| GTE-base | MTSamples | keyword | 1 | 0.0833 | 0.5862 | 0.6099 | 0.5012 | 0.6636 | 0.8100 | 0.4078 | 0.4131 | 21.4136 | 0.9163 | 0.1218 |
| GTE-base | MTSamples | keyword | 2 | 0.1667 | 0.4123 | 0.4137 | 0.3292 | 0.4985 | 0.6300 | 0.2869 | 0.2631 | 19.0607 | 0.9408 | 0.1343 |
| GTE-base | MTSamples | keyword | 3 | 0.2500 | 0.2126 | 0.1883 | 0.1418 | 0.2780 | 0.4000 | 0.1864 | 0.1385 | 16.9922 | 0.9437 | 0.1485 |
| GTE-base | MTSamples | keyword | 4 | 0.3333 | 0.0786 | 0.0370 | 0.0465 | 0.1145 | 0.2300 | 0.1098 | 0.0434 | 13.3896 | 0.9496 | 0.1976 |
| GTE-base | MTSamples | keyword | 5 | 0.4167 | 0.0951 | 0.0556 | 0.0559 | 0.1378 | 0.2500 | 0.1258 | 0.0632 | 13.9843 | 0.9450 | 0.1756 |
| GTE-base | MTSamples | keyword | 6 | 0.5000 | 0.0900 | 0.0499 | 0.0515 | 0.1313 | 0.2400 | 0.1138 | 0.0484 | 13.3394 | 0.9373 | 0.1815 |
| GTE-base | MTSamples | keyword | 7 | 0.5833 | 0.1082 | 0.0704 | 0.0641 | 0.1545 | 0.2800 | 0.1204 | 0.0565 | 12.8098 | 0.9216 | 0.1895 |
| GTE-base | MTSamples | keyword | 8 | 0.6667 | 0.0733 | 0.0310 | 0.0392 | 0.1138 | 0.2000 | 0.0912 | 0.0203 | 11.0710 | 0.9113 | 0.2098 |
| GTE-base | MTSamples | keyword | 9 | 0.7500 | 0.0458 | 0.0000 | 0.0223 | 0.0753 | 0.1500 | 0.0749 | 0.0000 | 8.9917 | 0.8999 | 0.2647 |
| GTE-base | MTSamples | keyword | 10 | 0.8333 | 0.0606 | 0.0167 | 0.0314 | 0.0951 | 0.1700 | 0.1220 | 0.0586 | 10.7899 | 0.9386 | 0.2435 |
| GTE-base | MTSamples | keyword | 11 | 0.9167 | 0.7879 | 0.8376 | 0.7261 | 0.8524 | 0.9600 | 0.7546 | 0.8435 | 30.1724 | 0.8287 | 0.1130 |
| GTE-base | MTSamples | keyword | 12 | 1.0000 | 0.9318 | 1.0000 | 0.8807 | 0.9675 | 0.9800 | 0.8806 | 1.0000 | 40.2811 | 0.7566 | 0.0796 |
| GTE-base | MTSamples | natural_language | 0 | 0.0000 | 0.2696 | 0.2360 | 0.2159 | 0.3293 | 0.7400 | 0.3944 | 0.3965 | 21.1071 | 0.8926 | 0.1276 |
| GTE-base | MTSamples | natural_language | 1 | 0.0833 | 0.3470 | 0.3242 | 0.2875 | 0.4105 | 0.8000 | 0.4078 | 0.4131 | 21.4136 | 0.9163 | 0.1218 |
| GTE-base | MTSamples | natural_language | 2 | 0.1667 | 0.2132 | 0.1716 | 0.1531 | 0.2841 | 0.4800 | 0.2869 | 0.2631 | 19.0607 | 0.9408 | 0.1343 |
| GTE-base | MTSamples | natural_language | 3 | 0.2500 | 0.1210 | 0.0665 | 0.0736 | 0.1748 | 0.2500 | 0.1864 | 0.1385 | 16.9922 | 0.9437 | 0.1485 |
| GTE-base | MTSamples | natural_language | 4 | 0.3333 | 0.0664 | 0.0042 | 0.0318 | 0.1060 | 0.1800 | 0.1098 | 0.0434 | 13.3896 | 0.9496 | 0.1976 |
| GTE-base | MTSamples | natural_language | 5 | 0.4167 | 0.0661 | 0.0039 | 0.0316 | 0.1066 | 0.1700 | 0.1258 | 0.0632 | 13.9843 | 0.9450 | 0.1756 |
| GTE-base | MTSamples | natural_language | 6 | 0.5000 | 0.0627 | 0.0000 | 0.0285 | 0.1050 | 0.1700 | 0.1138 | 0.0484 | 13.3394 | 0.9373 | 0.1815 |
| GTE-base | MTSamples | natural_language | 7 | 0.5833 | 0.0725 | 0.0112 | 0.0366 | 0.1126 | 0.2000 | 0.1204 | 0.0565 | 12.8098 | 0.9216 | 0.1895 |
| GTE-base | MTSamples | natural_language | 8 | 0.6667 | 0.0644 | 0.0019 | 0.0309 | 0.1023 | 0.1800 | 0.0912 | 0.0203 | 11.0710 | 0.9113 | 0.2098 |
| GTE-base | MTSamples | natural_language | 9 | 0.7500 | 0.0659 | 0.0036 | 0.0279 | 0.1099 | 0.1500 | 0.0749 | 0.0000 | 8.9917 | 0.8999 | 0.2647 |
| GTE-base | MTSamples | natural_language | 10 | 0.8333 | 0.0933 | 0.0349 | 0.0458 | 0.1471 | 0.1900 | 0.1220 | 0.0586 | 10.7899 | 0.9386 | 0.2435 |
| GTE-base | MTSamples | natural_language | 11 | 0.9167 | 0.8760 | 0.9276 | 0.8115 | 0.9268 | 0.9600 | 0.7546 | 0.8435 | 30.1724 | 0.8287 | 0.1130 |
| GTE-base | MTSamples | natural_language | 12 | 1.0000 | 0.9395 | 1.0000 | 0.8882 | 0.9733 | 0.9800 | 0.8806 | 1.0000 | 40.2811 | 0.7566 | 0.0796 |
| GTE-base | PMC-Patients | keyword | 0 | 0.0000 | 0.4406 | 0.4051 | 0.3629 | 0.5186 | 0.7500 | 0.3944 | 0.3965 | 24.7700 | 0.9035 | 0.1342 |
| GTE-base | PMC-Patients | keyword | 1 | 0.0833 | 0.3638 | 0.3207 | 0.2853 | 0.4388 | 0.6400 | 0.4078 | 0.4131 | 23.3830 | 0.9256 | 0.1347 |
| GTE-base | PMC-Patients | keyword | 2 | 0.1667 | 0.1940 | 0.1340 | 0.1326 | 0.2592 | 0.4200 | 0.2869 | 0.2631 | 18.9882 | 0.9462 | 0.1558 |
| GTE-base | PMC-Patients | keyword | 3 | 0.2500 | 0.1134 | 0.0454 | 0.0661 | 0.1677 | 0.2600 | 0.1864 | 0.1385 | 15.0109 | 0.9484 | 0.1963 |
| GTE-base | PMC-Patients | keyword | 4 | 0.3333 | 0.0721 | 0.0000 | 0.0337 | 0.1152 | 0.1700 | 0.1098 | 0.0434 | 11.1131 | 0.9527 | 0.2539 |
| GTE-base | PMC-Patients | keyword | 5 | 0.4167 | 0.0799 | 0.0086 | 0.0409 | 0.1235 | 0.2000 | 0.1258 | 0.0632 | 12.6273 | 0.9488 | 0.2256 |
| GTE-base | PMC-Patients | keyword | 6 | 0.5000 | 0.0872 | 0.0166 | 0.0441 | 0.1361 | 0.2200 | 0.1138 | 0.0484 | 12.7546 | 0.9464 | 0.2255 |
| GTE-base | PMC-Patients | keyword | 7 | 0.5833 | 0.0996 | 0.0302 | 0.0556 | 0.1501 | 0.2500 | 0.1204 | 0.0565 | 13.1544 | 0.9329 | 0.2170 |
| GTE-base | PMC-Patients | keyword | 8 | 0.6667 | 0.0807 | 0.0095 | 0.0392 | 0.1291 | 0.2100 | 0.0912 | 0.0203 | 12.9820 | 0.9252 | 0.2100 |
| GTE-base | PMC-Patients | keyword | 9 | 0.7500 | 0.0728 | 0.0008 | 0.0331 | 0.1217 | 0.1700 | 0.0749 | 0.0000 | 10.0319 | 0.9174 | 0.2663 |
| GTE-base | PMC-Patients | keyword | 10 | 0.8333 | 0.0843 | 0.0134 | 0.0416 | 0.1308 | 0.2000 | 0.1220 | 0.0586 | 13.3925 | 0.9417 | 0.2128 |
| GTE-base | PMC-Patients | keyword | 11 | 0.9167 | 0.8338 | 0.8374 | 0.7771 | 0.8879 | 0.9900 | 0.7546 | 0.8435 | 32.0735 | 0.8229 | 0.0819 |
| GTE-base | PMC-Patients | keyword | 12 | 1.0000 | 0.9817 | 1.0000 | 0.9567 | 1.0000 | 1.0000 | 0.8806 | 1.0000 | 44.4455 | 0.7326 | 0.0601 |
| GTE-base | PMC-Patients | natural_language | 0 | 0.0000 | 0.1580 | 0.0971 | 0.1016 | 0.2193 | 0.3800 | 0.3944 | 0.3965 | 24.7700 | 0.9035 | 0.1342 |
| GTE-base | PMC-Patients | natural_language | 1 | 0.0833 | 0.1778 | 0.1187 | 0.1201 | 0.2461 | 0.4000 | 0.4078 | 0.4131 | 23.3830 | 0.9256 | 0.1347 |
| GTE-base | PMC-Patients | natural_language | 2 | 0.1667 | 0.1159 | 0.0511 | 0.0674 | 0.1701 | 0.2800 | 0.2869 | 0.2631 | 18.9882 | 0.9462 | 0.1558 |
| GTE-base | PMC-Patients | natural_language | 3 | 0.2500 | 0.0806 | 0.0126 | 0.0406 | 0.1280 | 0.2000 | 0.1864 | 0.1385 | 15.0109 | 0.9484 | 0.1963 |
| GTE-base | PMC-Patients | natural_language | 4 | 0.3333 | 0.0693 | 0.0002 | 0.0328 | 0.1134 | 0.1700 | 0.1098 | 0.0434 | 11.1131 | 0.9527 | 0.2539 |
| GTE-base | PMC-Patients | natural_language | 5 | 0.4167 | 0.0695 | 0.0004 | 0.0310 | 0.1163 | 0.1700 | 0.1258 | 0.0632 | 12.6273 | 0.9488 | 0.2256 |
| GTE-base | PMC-Patients | natural_language | 6 | 0.5000 | 0.0691 | 0.0000 | 0.0314 | 0.1146 | 0.1600 | 0.1138 | 0.0484 | 12.7546 | 0.9464 | 0.2255 |
| GTE-base | PMC-Patients | natural_language | 7 | 0.5833 | 0.0758 | 0.0073 | 0.0346 | 0.1262 | 0.1600 | 0.1204 | 0.0565 | 13.1544 | 0.9329 | 0.2170 |
| GTE-base | PMC-Patients | natural_language | 8 | 0.6667 | 0.0775 | 0.0092 | 0.0364 | 0.1259 | 0.1700 | 0.0912 | 0.0203 | 12.9820 | 0.9252 | 0.2100 |
| GTE-base | PMC-Patients | natural_language | 9 | 0.7500 | 0.0762 | 0.0078 | 0.0335 | 0.1289 | 0.1400 | 0.0749 | 0.0000 | 10.0319 | 0.9174 | 0.2663 |
| GTE-base | PMC-Patients | natural_language | 10 | 0.8333 | 0.0938 | 0.0270 | 0.0464 | 0.1441 | 0.2000 | 0.1220 | 0.0586 | 13.3925 | 0.9417 | 0.2128 |
| GTE-base | PMC-Patients | natural_language | 11 | 0.9167 | 0.8963 | 0.9032 | 0.8447 | 0.9404 | 0.9900 | 0.7546 | 0.8435 | 32.0735 | 0.8229 | 0.0819 |
| GTE-base | PMC-Patients | natural_language | 12 | 1.0000 | 0.9850 | 1.0000 | 0.9600 | 1.0000 | 0.9900 | 0.8806 | 1.0000 | 44.4455 | 0.7326 | 0.0601 |
| GTE-base | Synthetic | keyword | 0 | 0.0000 | 0.5474 | 0.6927 | 0.4709 | 0.6234 | 0.8600 | 0.3944 | 0.3965 | 18.8024 | 0.9516 | 0.1676 |
| GTE-base | Synthetic | keyword | 1 | 0.0833 | 0.5026 | 0.6184 | 0.4144 | 0.5629 | 0.8000 | 0.4078 | 0.4131 | 17.7707 | 0.9588 | 0.1660 |
| GTE-base | Synthetic | keyword | 2 | 0.1667 | 0.4163 | 0.4752 | 0.3453 | 0.4995 | 0.6900 | 0.2869 | 0.2631 | 17.0575 | 0.9722 | 0.1737 |
| GTE-base | Synthetic | keyword | 3 | 0.2500 | 0.3462 | 0.3589 | 0.2727 | 0.4203 | 0.6700 | 0.1864 | 0.1385 | 17.0002 | 0.9751 | 0.1793 |
| GTE-base | Synthetic | keyword | 4 | 0.3333 | 0.2577 | 0.2120 | 0.1955 | 0.3244 | 0.5800 | 0.1098 | 0.0434 | 15.7900 | 0.9797 | 0.1966 |
| GTE-base | Synthetic | keyword | 5 | 0.4167 | 0.3359 | 0.3418 | 0.2637 | 0.4133 | 0.6600 | 0.1258 | 0.0632 | 15.2165 | 0.9776 | 0.1995 |
| GTE-base | Synthetic | keyword | 6 | 0.5000 | 0.2912 | 0.2676 | 0.2291 | 0.3615 | 0.6300 | 0.1138 | 0.0484 | 14.4730 | 0.9757 | 0.2096 |
| GTE-base | Synthetic | keyword | 7 | 0.5833 | 0.2722 | 0.2361 | 0.2122 | 0.3408 | 0.6500 | 0.1204 | 0.0565 | 15.3413 | 0.9698 | 0.1901 |
| GTE-base | Synthetic | keyword | 8 | 0.6667 | 0.1879 | 0.0962 | 0.1392 | 0.2465 | 0.5100 | 0.0912 | 0.0203 | 14.3614 | 0.9683 | 0.2026 |
| GTE-base | Synthetic | keyword | 9 | 0.7500 | 0.1299 | 0.0000 | 0.0852 | 0.1816 | 0.4000 | 0.0749 | 0.0000 | 14.6557 | 0.9732 | 0.1730 |
| GTE-base | Synthetic | keyword | 10 | 0.8333 | 0.2536 | 0.2052 | 0.1913 | 0.3192 | 0.5700 | 0.1220 | 0.0586 | 16.6379 | 0.9786 | 0.1463 |
| GTE-base | Synthetic | keyword | 11 | 0.9167 | 0.5575 | 0.7095 | 0.4806 | 0.6286 | 0.8800 | 0.7546 | 0.8435 | 16.4606 | 0.8894 | 0.1565 |
| GTE-base | Synthetic | keyword | 12 | 1.0000 | 0.7326 | 1.0000 | 0.6578 | 0.7944 | 0.9500 | 0.8806 | 1.0000 | 21.1933 | 0.7956 | 0.1199 |
| GTE-base | Synthetic | natural_language | 0 | 0.0000 | 0.3614 | 0.4626 | 0.2832 | 0.4412 | 0.6100 | 0.3944 | 0.3965 | 18.8024 | 0.9516 | 0.1676 |
| GTE-base | Synthetic | natural_language | 1 | 0.0833 | 0.4691 | 0.6272 | 0.3774 | 0.5451 | 0.7100 | 0.4078 | 0.4131 | 17.7707 | 0.9588 | 0.1660 |
| GTE-base | Synthetic | natural_language | 2 | 0.1667 | 0.3695 | 0.4750 | 0.2894 | 0.4494 | 0.6400 | 0.2869 | 0.2631 | 17.0575 | 0.9722 | 0.1737 |
| GTE-base | Synthetic | natural_language | 3 | 0.2500 | 0.2449 | 0.2846 | 0.1780 | 0.3191 | 0.5500 | 0.1864 | 0.1385 | 17.0002 | 0.9751 | 0.1793 |
| GTE-base | Synthetic | natural_language | 4 | 0.3333 | 0.1147 | 0.0857 | 0.0810 | 0.1560 | 0.4600 | 0.1098 | 0.0434 | 15.7900 | 0.9797 | 0.1966 |
| GTE-base | Synthetic | natural_language | 5 | 0.4167 | 0.1080 | 0.0755 | 0.0710 | 0.1534 | 0.3800 | 0.1258 | 0.0632 | 15.2165 | 0.9776 | 0.1995 |
| GTE-base | Synthetic | natural_language | 6 | 0.5000 | 0.0829 | 0.0371 | 0.0567 | 0.1133 | 0.3400 | 0.1138 | 0.0484 | 14.4730 | 0.9757 | 0.2096 |
| GTE-base | Synthetic | natural_language | 7 | 0.5833 | 0.0940 | 0.0541 | 0.0636 | 0.1318 | 0.3800 | 0.1204 | 0.0565 | 15.3413 | 0.9698 | 0.1901 |
| GTE-base | Synthetic | natural_language | 8 | 0.6667 | 0.0634 | 0.0073 | 0.0358 | 0.0981 | 0.2600 | 0.0912 | 0.0203 | 14.3614 | 0.9683 | 0.2026 |
| GTE-base | Synthetic | natural_language | 9 | 0.7500 | 0.0586 | 0.0000 | 0.0252 | 0.0972 | 0.1500 | 0.0749 | 0.0000 | 14.6557 | 0.9732 | 0.1730 |
| GTE-base | Synthetic | natural_language | 10 | 0.8333 | 0.1467 | 0.1346 | 0.1005 | 0.1974 | 0.3900 | 0.1220 | 0.0586 | 16.6379 | 0.9786 | 0.1463 |
| GTE-base | Synthetic | natural_language | 11 | 0.9167 | 0.5758 | 0.7902 | 0.5073 | 0.6565 | 0.8900 | 0.7546 | 0.8435 | 16.4606 | 0.8894 | 0.1565 |
| GTE-base | Synthetic | natural_language | 12 | 1.0000 | 0.7131 | 1.0000 | 0.6431 | 0.7862 | 0.9400 | 0.8806 | 1.0000 | 21.1933 | 0.7956 | 0.1199 |
| MedCPT | MTSamples | keyword | 0 | 0.0000 | 1.0000 | 1.0000 | 1.0000 | 1.0000 | 1.0000 | 1.0000 | 1.0000 | 1.0000 | 1.0000 | 1.0000 |
| MedCPT | MTSamples | keyword | 1 | 0.0833 | 0.0326 | 0.0070 | 0.0120 | 0.0592 | 0.1100 | 0.0320 | 0.0069 | 2.0602 | 0.9869 | 0.6894 |
| MedCPT | MTSamples | keyword | 2 | 0.1667 | 0.0366 | 0.0111 | 0.0138 | 0.0620 | 0.1200 | 0.0328 | 0.0077 | 3.2092 | 0.9905 | 0.5274 |
| MedCPT | MTSamples | keyword | 3 | 0.2500 | 0.0294 | 0.0037 | 0.0097 | 0.0538 | 0.1000 | 0.0301 | 0.0049 | 9.9095 | 0.9786 | 0.2746 |
| MedCPT | MTSamples | keyword | 4 | 0.3333 | 0.0293 | 0.0036 | 0.0090 | 0.0566 | 0.1000 | 0.0282 | 0.0030 | 5.6816 | 0.9702 | 0.3924 |
| MedCPT | MTSamples | keyword | 5 | 0.4167 | 0.0291 | 0.0034 | 0.0085 | 0.0557 | 0.0900 | 0.0282 | 0.0030 | 8.1440 | 0.9633 | 0.3050 |
| MedCPT | MTSamples | keyword | 6 | 0.5000 | 0.0258 | 0.0000 | 0.0077 | 0.0506 | 0.1000 | 0.0253 | 0.0000 | 6.6400 | 0.9440 | 0.3472 |
| MedCPT | MTSamples | keyword | 7 | 0.5833 | 0.0343 | 0.0087 | 0.0097 | 0.0553 | 0.1000 | 0.0316 | 0.0065 | 5.7752 | 0.9539 | 0.3847 |
| MedCPT | MTSamples | keyword | 8 | 0.6667 | 0.0407 | 0.0153 | 0.0095 | 0.0775 | 0.1000 | 0.0330 | 0.0079 | 4.1303 | 0.9617 | 0.4698 |
| MedCPT | MTSamples | keyword | 9 | 0.7500 | 0.0299 | 0.0042 | 0.0098 | 0.0569 | 0.1000 | 0.0310 | 0.0059 | 3.6604 | 0.9753 | 0.5034 |
| MedCPT | MTSamples | keyword | 10 | 0.8333 | 0.0617 | 0.0369 | 0.0317 | 0.0947 | 0.2100 | 0.0642 | 0.0399 | 9.3181 | 0.9504 | 0.2847 |
| MedCPT | MTSamples | keyword | 11 | 0.9167 | 0.8680 | 0.8645 | 0.8068 | 0.9180 | 0.9600 | 0.8203 | 0.8157 | 27.9422 | 0.8784 | 0.1335 |
| MedCPT | MTSamples | keyword | 12 | 1.0000 | 0.8808 | 0.8776 | 0.8200 | 0.9283 | 0.9500 | 0.8274 | 0.8229 | 35.7609 | 0.7214 | 0.1097 |
| MedCPT | MTSamples | natural_language | 0 | 0.0000 | 1.0000 | 1.0000 | 1.0000 | 1.0000 | 1.0000 | 1.0000 | 1.0000 | 1.0000 | 1.0000 | 1.0000 |
| MedCPT | MTSamples | natural_language | 1 | 0.0833 | 0.0334 | 0.0099 | 0.0134 | 0.0608 | 0.1200 | 0.0320 | 0.0069 | 2.0602 | 0.9869 | 0.6894 |
| MedCPT | MTSamples | natural_language | 2 | 0.1667 | 0.0328 | 0.0093 | 0.0114 | 0.0587 | 0.1100 | 0.0328 | 0.0077 | 3.2092 | 0.9905 | 0.5274 |
| MedCPT | MTSamples | natural_language | 3 | 0.2500 | 0.0273 | 0.0037 | 0.0087 | 0.0512 | 0.1000 | 0.0301 | 0.0049 | 9.9095 | 0.9786 | 0.2746 |
| MedCPT | MTSamples | natural_language | 4 | 0.3333 | 0.0291 | 0.0055 | 0.0086 | 0.0574 | 0.1000 | 0.0282 | 0.0030 | 5.6816 | 0.9702 | 0.3924 |
| MedCPT | MTSamples | natural_language | 5 | 0.4167 | 0.0268 | 0.0032 | 0.0071 | 0.0527 | 0.0900 | 0.0282 | 0.0030 | 8.1440 | 0.9633 | 0.3050 |
| MedCPT | MTSamples | natural_language | 6 | 0.5000 | 0.0237 | 0.0000 | 0.0078 | 0.0475 | 0.1000 | 0.0253 | 0.0000 | 6.6400 | 0.9440 | 0.3472 |
| MedCPT | MTSamples | natural_language | 7 | 0.5833 | 0.0343 | 0.0109 | 0.0094 | 0.0556 | 0.1000 | 0.0316 | 0.0065 | 5.7752 | 0.9539 | 0.3847 |
| MedCPT | MTSamples | natural_language | 8 | 0.6667 | 0.0360 | 0.0126 | 0.0110 | 0.0680 | 0.1000 | 0.0330 | 0.0079 | 4.1303 | 0.9617 | 0.4698 |
| MedCPT | MTSamples | natural_language | 9 | 0.7500 | 0.0300 | 0.0065 | 0.0092 | 0.0570 | 0.1000 | 0.0310 | 0.0059 | 3.6604 | 0.9753 | 0.5034 |
| MedCPT | MTSamples | natural_language | 10 | 0.8333 | 0.0441 | 0.0209 | 0.0162 | 0.0782 | 0.1200 | 0.0642 | 0.0399 | 9.3181 | 0.9504 | 0.2847 |
| MedCPT | MTSamples | natural_language | 11 | 0.9167 | 0.7948 | 0.7898 | 0.7207 | 0.8606 | 0.9200 | 0.8203 | 0.8157 | 27.9422 | 0.8784 | 0.1335 |
| MedCPT | MTSamples | natural_language | 12 | 1.0000 | 0.8036 | 0.7988 | 0.7260 | 0.8591 | 0.9300 | 0.8274 | 0.8229 | 35.7609 | 0.7214 | 0.1097 |
| MedCPT | PMC-Patients | keyword | 0 | 0.0000 | 1.0000 | 1.0000 | 1.0000 | 1.0000 | 1.0000 | 1.0000 | 1.0000 | 1.0000 | 1.0000 | 1.0000 |
| MedCPT | PMC-Patients | keyword | 1 | 0.0833 | 0.0295 | 0.0080 | 0.0093 | 0.0558 | 0.1000 | 0.0320 | 0.0069 | 2.2282 | 0.9884 | 0.6579 |
| MedCPT | PMC-Patients | keyword | 2 | 0.1667 | 0.0324 | 0.0109 | 0.0109 | 0.0594 | 0.1000 | 0.0328 | 0.0077 | 3.8139 | 0.9907 | 0.4601 |
| MedCPT | PMC-Patients | keyword | 3 | 0.2500 | 0.0348 | 0.0134 | 0.0097 | 0.0672 | 0.1000 | 0.0301 | 0.0049 | 9.2785 | 0.9807 | 0.2933 |
| MedCPT | PMC-Patients | keyword | 4 | 0.3333 | 0.0304 | 0.0089 | 0.0105 | 0.0569 | 0.1200 | 0.0282 | 0.0030 | 6.4395 | 0.9744 | 0.3659 |
| MedCPT | PMC-Patients | keyword | 5 | 0.4167 | 0.0217 | 0.0000 | 0.0073 | 0.0395 | 0.0800 | 0.0282 | 0.0030 | 10.4020 | 0.9668 | 0.2637 |
| MedCPT | PMC-Patients | keyword | 6 | 0.5000 | 0.0240 | 0.0024 | 0.0053 | 0.0503 | 0.0600 | 0.0253 | 0.0000 | 11.4495 | 0.9455 | 0.2166 |
| MedCPT | PMC-Patients | keyword | 7 | 0.5833 | 0.0293 | 0.0078 | 0.0096 | 0.0569 | 0.1000 | 0.0316 | 0.0065 | 10.3688 | 0.9554 | 0.2518 |
| MedCPT | PMC-Patients | keyword | 8 | 0.6667 | 0.0293 | 0.0078 | 0.0095 | 0.0548 | 0.1000 | 0.0330 | 0.0079 | 6.2973 | 0.9644 | 0.3712 |
| MedCPT | PMC-Patients | keyword | 9 | 0.7500 | 0.0315 | 0.0100 | 0.0101 | 0.0585 | 0.1000 | 0.0310 | 0.0059 | 5.7324 | 0.9789 | 0.3933 |
| MedCPT | PMC-Patients | keyword | 10 | 0.8333 | 0.0957 | 0.0756 | 0.0537 | 0.1424 | 0.2900 | 0.0642 | 0.0399 | 16.9641 | 0.9522 | 0.1912 |
| MedCPT | PMC-Patients | keyword | 11 | 0.9167 | 0.9233 | 0.9216 | 0.8833 | 0.9583 | 1.0000 | 0.8203 | 0.8157 | 45.5973 | 0.8720 | 0.0647 |
| MedCPT | PMC-Patients | keyword | 12 | 1.0000 | 0.9267 | 0.9251 | 0.8883 | 0.9617 | 1.0000 | 0.8274 | 0.8229 | 54.2270 | 0.7080 | 0.0530 |
| MedCPT | PMC-Patients | natural_language | 0 | 0.0000 | 1.0000 | 1.0000 | 1.0000 | 1.0000 | 1.0000 | 1.0000 | 1.0000 | 1.0000 | 1.0000 | 1.0000 |
| MedCPT | PMC-Patients | natural_language | 1 | 0.0833 | 0.0293 | 0.0117 | 0.0090 | 0.0562 | 0.1000 | 0.0320 | 0.0069 | 2.2282 | 0.9884 | 0.6579 |
| MedCPT | PMC-Patients | natural_language | 2 | 0.1667 | 0.0316 | 0.0141 | 0.0114 | 0.0614 | 0.1000 | 0.0328 | 0.0077 | 3.8139 | 0.9907 | 0.4601 |
| MedCPT | PMC-Patients | natural_language | 3 | 0.2500 | 0.0304 | 0.0128 | 0.0101 | 0.0586 | 0.1000 | 0.0301 | 0.0049 | 9.2785 | 0.9807 | 0.2933 |
| MedCPT | PMC-Patients | natural_language | 4 | 0.3333 | 0.0272 | 0.0096 | 0.0065 | 0.0541 | 0.0800 | 0.0282 | 0.0030 | 6.4395 | 0.9744 | 0.3659 |
| MedCPT | PMC-Patients | natural_language | 5 | 0.4167 | 0.0226 | 0.0049 | 0.0082 | 0.0405 | 0.0900 | 0.0282 | 0.0030 | 10.4020 | 0.9668 | 0.2637 |
| MedCPT | PMC-Patients | natural_language | 6 | 0.5000 | 0.0178 | 0.0000 | 0.0045 | 0.0342 | 0.0600 | 0.0253 | 0.0000 | 11.4495 | 0.9455 | 0.2166 |
| MedCPT | PMC-Patients | natural_language | 7 | 0.5833 | 0.0293 | 0.0117 | 0.0087 | 0.0557 | 0.1000 | 0.0316 | 0.0065 | 10.3688 | 0.9554 | 0.2518 |
| MedCPT | PMC-Patients | natural_language | 8 | 0.6667 | 0.0293 | 0.0117 | 0.0095 | 0.0553 | 0.1000 | 0.0330 | 0.0079 | 6.2973 | 0.9644 | 0.3712 |
| MedCPT | PMC-Patients | natural_language | 9 | 0.7500 | 0.0310 | 0.0134 | 0.0095 | 0.0571 | 0.1000 | 0.0310 | 0.0059 | 5.7324 | 0.9789 | 0.3933 |
| MedCPT | PMC-Patients | natural_language | 10 | 0.8333 | 0.0576 | 0.0405 | 0.0261 | 0.0975 | 0.1800 | 0.0642 | 0.0399 | 16.9641 | 0.9522 | 0.1912 |
| MedCPT | PMC-Patients | natural_language | 11 | 0.9167 | 0.8908 | 0.8888 | 0.8432 | 0.9353 | 1.0000 | 0.8203 | 0.8157 | 45.5973 | 0.8720 | 0.0647 |
| MedCPT | PMC-Patients | natural_language | 12 | 1.0000 | 0.8839 | 0.8818 | 0.8370 | 0.9275 | 0.9900 | 0.8274 | 0.8229 | 54.2270 | 0.7080 | 0.0530 |
| MedCPT | Synthetic | keyword | 0 | 0.0000 | 1.0000 | 1.0000 | 1.0000 | 1.0000 | 1.0000 | 1.0000 | 1.0000 | 1.0000 | 1.0000 | 1.0000 |
| MedCPT | Synthetic | keyword | 1 | 0.0833 | 0.0350 | 0.0059 | 0.0143 | 0.0621 | 0.1400 | 0.0320 | 0.0069 | 6.6327 | 0.9976 | 0.3399 |
| MedCPT | Synthetic | keyword | 2 | 0.1667 | 0.0312 | 0.0020 | 0.0115 | 0.0576 | 0.1000 | 0.0328 | 0.0077 | 9.9490 | 0.9978 | 0.2654 |
| MedCPT | Synthetic | keyword | 3 | 0.2500 | 0.0293 | 0.0000 | 0.0088 | 0.0567 | 0.0900 | 0.0301 | 0.0049 | 17.6852 | 0.9932 | 0.1225 |
| MedCPT | Synthetic | keyword | 4 | 0.3333 | 0.0300 | 0.0007 | 0.0094 | 0.0554 | 0.0900 | 0.0282 | 0.0030 | 15.0090 | 0.9919 | 0.1682 |
| MedCPT | Synthetic | keyword | 5 | 0.4167 | 0.0341 | 0.0049 | 0.0101 | 0.0668 | 0.1000 | 0.0282 | 0.0030 | 15.6615 | 0.9879 | 0.1487 |
| MedCPT | Synthetic | keyword | 6 | 0.5000 | 0.0308 | 0.0015 | 0.0100 | 0.0575 | 0.1000 | 0.0253 | 0.0000 | 11.2790 | 0.9814 | 0.2192 |
| MedCPT | Synthetic | keyword | 7 | 0.5833 | 0.0313 | 0.0021 | 0.0096 | 0.0543 | 0.1000 | 0.0316 | 0.0065 | 11.0703 | 0.9836 | 0.2131 |
| MedCPT | Synthetic | keyword | 8 | 0.6667 | 0.0314 | 0.0022 | 0.0104 | 0.0584 | 0.1000 | 0.0330 | 0.0079 | 10.3381 | 0.9884 | 0.2443 |
| MedCPT | Synthetic | keyword | 9 | 0.7500 | 0.0322 | 0.0030 | 0.0105 | 0.0582 | 0.1100 | 0.0310 | 0.0059 | 9.4979 | 0.9934 | 0.2700 |
| MedCPT | Synthetic | keyword | 10 | 0.8333 | 0.0833 | 0.0556 | 0.0476 | 0.1261 | 0.2600 | 0.0642 | 0.0399 | 18.1468 | 0.9787 | 0.1412 |
| MedCPT | Synthetic | keyword | 11 | 0.9167 | 0.7490 | 0.7414 | 0.6478 | 0.7806 | 0.9700 | 0.8203 | 0.8157 | 25.6250 | 0.9284 | 0.0972 |
| MedCPT | Synthetic | keyword | 12 | 1.0000 | 0.7431 | 0.7353 | 0.6405 | 0.7710 | 0.9600 | 0.8274 | 0.8229 | 27.7061 | 0.8227 | 0.0891 |
| MedCPT | Synthetic | natural_language | 0 | 0.0000 | 1.0000 | 1.0000 | 1.0000 | 1.0000 | 1.0000 | 1.0000 | 1.0000 | 1.0000 | 1.0000 | 1.0000 |
| MedCPT | Synthetic | natural_language | 1 | 0.0833 | 0.0322 | 0.0090 | 0.0125 | 0.0588 | 0.1200 | 0.0320 | 0.0069 | 6.6327 | 0.9976 | 0.3399 |
| MedCPT | Synthetic | natural_language | 2 | 0.1667 | 0.0323 | 0.0091 | 0.0110 | 0.0598 | 0.1100 | 0.0328 | 0.0077 | 9.9490 | 0.9978 | 0.2654 |
| MedCPT | Synthetic | natural_language | 3 | 0.2500 | 0.0294 | 0.0061 | 0.0095 | 0.0552 | 0.1000 | 0.0301 | 0.0049 | 17.6852 | 0.9932 | 0.1225 |
| MedCPT | Synthetic | natural_language | 4 | 0.3333 | 0.0234 | 0.0000 | 0.0079 | 0.0408 | 0.0900 | 0.0282 | 0.0030 | 15.0090 | 0.9919 | 0.1682 |
| MedCPT | Synthetic | natural_language | 5 | 0.4167 | 0.0348 | 0.0117 | 0.0093 | 0.0563 | 0.1000 | 0.0282 | 0.0030 | 15.6615 | 0.9879 | 0.1487 |
| MedCPT | Synthetic | natural_language | 6 | 0.5000 | 0.0296 | 0.0063 | 0.0084 | 0.0546 | 0.0900 | 0.0253 | 0.0000 | 11.2790 | 0.9814 | 0.2192 |
| MedCPT | Synthetic | natural_language | 7 | 0.5833 | 0.0313 | 0.0081 | 0.0094 | 0.0569 | 0.1000 | 0.0316 | 0.0065 | 11.0703 | 0.9836 | 0.2131 |
| MedCPT | Synthetic | natural_language | 8 | 0.6667 | 0.0314 | 0.0082 | 0.0096 | 0.0596 | 0.1000 | 0.0330 | 0.0079 | 10.3381 | 0.9884 | 0.2443 |
| MedCPT | Synthetic | natural_language | 9 | 0.7500 | 0.0316 | 0.0084 | 0.0100 | 0.0571 | 0.1100 | 0.0310 | 0.0059 | 9.4979 | 0.9934 | 0.2700 |
| MedCPT | Synthetic | natural_language | 10 | 0.8333 | 0.0428 | 0.0199 | 0.0197 | 0.0711 | 0.1600 | 0.0642 | 0.0399 | 18.1468 | 0.9787 | 0.1412 |
| MedCPT | Synthetic | natural_language | 11 | 0.9167 | 0.6960 | 0.6887 | 0.6102 | 0.7500 | 0.9300 | 0.8203 | 0.8157 | 25.6250 | 0.9284 | 0.0972 |
| MedCPT | Synthetic | natural_language | 12 | 1.0000 | 0.7263 | 0.7197 | 0.6246 | 0.7669 | 0.9500 | 0.8274 | 0.8229 | 27.7061 | 0.8227 | 0.0891 |
| Nomic-embed-text | MTSamples | keyword | 0 | 0.0000 | 0.4248 | 0.3781 | 0.3390 | 0.5078 | 0.6200 | 0.4614 | 0.3701 | 23.0806 | 0.9957 | 0.1301 |
| Nomic-embed-text | MTSamples | keyword | 1 | 0.0833 | 0.6005 | 0.5986 | 0.5257 | 0.6784 | 0.8000 | 0.5446 | 0.5005 | 22.9670 | 0.9942 | 0.1256 |
| Nomic-embed-text | MTSamples | keyword | 2 | 0.1667 | 0.4760 | 0.4424 | 0.3943 | 0.5587 | 0.7100 | 0.4378 | 0.3330 | 17.6271 | 0.9970 | 0.1637 |
| Nomic-embed-text | MTSamples | keyword | 3 | 0.2500 | 0.2754 | 0.1906 | 0.2015 | 0.3555 | 0.4800 | 0.3300 | 0.1638 | 13.6885 | 0.9967 | 0.1897 |
| Nomic-embed-text | MTSamples | keyword | 4 | 0.3333 | 0.2207 | 0.1220 | 0.1525 | 0.2953 | 0.4300 | 0.2576 | 0.0504 | 12.4978 | 0.9955 | 0.2128 |
| Nomic-embed-text | MTSamples | keyword | 5 | 0.4167 | 0.2303 | 0.1340 | 0.1656 | 0.3033 | 0.4500 | 0.3014 | 0.1190 | 11.5104 | 0.9940 | 0.2339 |
| Nomic-embed-text | MTSamples | keyword | 6 | 0.5000 | 0.1470 | 0.0295 | 0.0961 | 0.2041 | 0.3600 | 0.2255 | 0.0000 | 9.8267 | 0.9938 | 0.2615 |
| Nomic-embed-text | MTSamples | keyword | 7 | 0.5833 | 0.2209 | 0.1222 | 0.1568 | 0.2895 | 0.4400 | 0.2638 | 0.0601 | 11.6099 | 0.9908 | 0.2260 |
| Nomic-embed-text | MTSamples | keyword | 8 | 0.6667 | 0.3553 | 0.2909 | 0.2770 | 0.4401 | 0.6500 | 0.3554 | 0.2037 | 11.9147 | 0.9966 | 0.2272 |
| Nomic-embed-text | MTSamples | keyword | 9 | 0.7500 | 0.1262 | 0.0034 | 0.0819 | 0.1761 | 0.3400 | 0.2555 | 0.0471 | 11.4797 | 0.9963 | 0.2226 |
| Nomic-embed-text | MTSamples | keyword | 10 | 0.8333 | 0.1235 | 0.0000 | 0.0744 | 0.1798 | 0.2900 | 0.2427 | 0.0270 | 11.8367 | 0.9981 | 0.2173 |
| Nomic-embed-text | MTSamples | keyword | 11 | 0.9167 | 0.8245 | 0.8798 | 0.7568 | 0.8875 | 0.9200 | 0.7441 | 0.8133 | 18.3278 | 0.9948 | 0.1854 |
| Nomic-embed-text | MTSamples | keyword | 12 | 1.0000 | 0.9203 | 1.0000 | 0.8730 | 0.9633 | 0.9600 | 0.8631 | 1.0000 | 31.7409 | 0.6487 | 0.1213 |
| Nomic-embed-text | MTSamples | natural_language | 0 | 0.0000 | 0.6320 | 0.5451 | 0.5448 | 0.7164 | 0.8200 | 0.4614 | 0.3701 | 23.0806 | 0.9957 | 0.1301 |
| Nomic-embed-text | MTSamples | natural_language | 1 | 0.0833 | 0.6380 | 0.5542 | 0.5579 | 0.7190 | 0.8400 | 0.5446 | 0.5005 | 22.9670 | 0.9942 | 0.1256 |
| Nomic-embed-text | MTSamples | natural_language | 2 | 0.1667 | 0.4578 | 0.2807 | 0.3737 | 0.5346 | 0.7400 | 0.4378 | 0.3330 | 17.6271 | 0.9970 | 0.1637 |
| Nomic-embed-text | MTSamples | natural_language | 3 | 0.2500 | 0.3395 | 0.1011 | 0.2715 | 0.4105 | 0.6600 | 0.3300 | 0.1638 | 13.6885 | 0.9967 | 0.1897 |
| Nomic-embed-text | MTSamples | natural_language | 4 | 0.3333 | 0.2760 | 0.0047 | 0.2184 | 0.3357 | 0.6800 | 0.2576 | 0.0504 | 12.4978 | 0.9955 | 0.2128 |
| Nomic-embed-text | MTSamples | natural_language | 5 | 0.4167 | 0.3492 | 0.1158 | 0.2875 | 0.4176 | 0.6900 | 0.3014 | 0.1190 | 11.5104 | 0.9940 | 0.2339 |
| Nomic-embed-text | MTSamples | natural_language | 6 | 0.5000 | 0.2729 | 0.0000 | 0.2095 | 0.3377 | 0.5400 | 0.2255 | 0.0000 | 9.8267 | 0.9938 | 0.2615 |
| Nomic-embed-text | MTSamples | natural_language | 7 | 0.5833 | 0.2941 | 0.0322 | 0.2178 | 0.3729 | 0.4900 | 0.2638 | 0.0601 | 11.6099 | 0.9908 | 0.2260 |
| Nomic-embed-text | MTSamples | natural_language | 8 | 0.6667 | 0.4107 | 0.2092 | 0.3332 | 0.4935 | 0.7000 | 0.3554 | 0.2037 | 11.9147 | 0.9966 | 0.2272 |
| Nomic-embed-text | MTSamples | natural_language | 9 | 0.7500 | 0.2803 | 0.0112 | 0.2102 | 0.3552 | 0.5600 | 0.2555 | 0.0471 | 11.4797 | 0.9963 | 0.2226 |
| Nomic-embed-text | MTSamples | natural_language | 10 | 0.8333 | 0.3019 | 0.0440 | 0.2245 | 0.3813 | 0.5300 | 0.2427 | 0.0270 | 11.8367 | 0.9981 | 0.2173 |
| Nomic-embed-text | MTSamples | natural_language | 11 | 0.9167 | 0.8605 | 0.8919 | 0.7997 | 0.9144 | 0.9400 | 0.7441 | 0.8133 | 18.3278 | 0.9948 | 0.1854 |
| Nomic-embed-text | MTSamples | natural_language | 12 | 1.0000 | 0.9317 | 1.0000 | 0.8917 | 0.9767 | 0.9600 | 0.8631 | 1.0000 | 31.7409 | 0.6487 | 0.1213 |
| Nomic-embed-text | PMC-Patients | keyword | 0 | 0.0000 | 0.3679 | 0.2516 | 0.2803 | 0.4541 | 0.5300 | 0.4614 | 0.3701 | 28.1064 | 0.9965 | 0.1240 |
| Nomic-embed-text | PMC-Patients | keyword | 1 | 0.0833 | 0.5098 | 0.4348 | 0.4222 | 0.5980 | 0.6400 | 0.5446 | 0.5005 | 26.9581 | 0.9951 | 0.1289 |
| Nomic-embed-text | PMC-Patients | keyword | 2 | 0.1667 | 0.4070 | 0.3021 | 0.3208 | 0.4912 | 0.6200 | 0.4378 | 0.3330 | 20.7939 | 0.9974 | 0.1489 |
| Nomic-embed-text | PMC-Patients | keyword | 3 | 0.2500 | 0.3074 | 0.1736 | 0.2329 | 0.3908 | 0.4500 | 0.3300 | 0.1638 | 16.6095 | 0.9972 | 0.1620 |
| Nomic-embed-text | PMC-Patients | keyword | 4 | 0.3333 | 0.2324 | 0.0768 | 0.1673 | 0.3041 | 0.4500 | 0.2576 | 0.0504 | 16.7533 | 0.9962 | 0.1567 |
| Nomic-embed-text | PMC-Patients | keyword | 5 | 0.4167 | 0.2576 | 0.1093 | 0.1870 | 0.3317 | 0.4800 | 0.3014 | 0.1190 | 18.1841 | 0.9950 | 0.1402 |
| Nomic-embed-text | PMC-Patients | keyword | 6 | 0.5000 | 0.2052 | 0.0417 | 0.1391 | 0.2688 | 0.4400 | 0.2255 | 0.0000 | 15.8651 | 0.9950 | 0.1658 |
| Nomic-embed-text | PMC-Patients | keyword | 7 | 0.5833 | 0.2149 | 0.0542 | 0.1517 | 0.2840 | 0.4900 | 0.2638 | 0.0601 | 17.7932 | 0.9923 | 0.1550 |
| Nomic-embed-text | PMC-Patients | keyword | 8 | 0.6667 | 0.3133 | 0.1812 | 0.2445 | 0.3901 | 0.5800 | 0.3554 | 0.2037 | 19.6301 | 0.9972 | 0.1478 |
| Nomic-embed-text | PMC-Patients | keyword | 9 | 0.7500 | 0.2107 | 0.0488 | 0.1429 | 0.2745 | 0.4500 | 0.2555 | 0.0471 | 18.1974 | 0.9970 | 0.1575 |
| Nomic-embed-text | PMC-Patients | keyword | 10 | 0.8333 | 0.1729 | 0.0000 | 0.1113 | 0.2403 | 0.3700 | 0.2427 | 0.0270 | 19.3396 | 0.9984 | 0.1485 |
| Nomic-embed-text | PMC-Patients | keyword | 11 | 0.9167 | 0.7897 | 0.7960 | 0.7255 | 0.8522 | 0.9900 | 0.7441 | 0.8133 | 28.0169 | 0.9956 | 0.1148 |
| Nomic-embed-text | PMC-Patients | keyword | 12 | 1.0000 | 0.9478 | 1.0000 | 0.9128 | 0.9800 | 1.0000 | 0.8631 | 1.0000 | 43.1663 | 0.6605 | 0.0709 |
| Nomic-embed-text | PMC-Patients | natural_language | 0 | 0.0000 | 0.5981 | 0.5393 | 0.5117 | 0.6839 | 0.7700 | 0.4614 | 0.3701 | 28.1064 | 0.9965 | 0.1240 |
| Nomic-embed-text | PMC-Patients | natural_language | 1 | 0.0833 | 0.5789 | 0.5156 | 0.4983 | 0.6591 | 0.8000 | 0.5446 | 0.5005 | 26.9581 | 0.9951 | 0.1289 |
| Nomic-embed-text | PMC-Patients | natural_language | 2 | 0.1667 | 0.4044 | 0.2999 | 0.3226 | 0.4864 | 0.6800 | 0.4378 | 0.3330 | 20.7939 | 0.9974 | 0.1489 |
| Nomic-embed-text | PMC-Patients | natural_language | 3 | 0.2500 | 0.3268 | 0.2040 | 0.2510 | 0.4060 | 0.5500 | 0.3300 | 0.1638 | 16.6095 | 0.9972 | 0.1620 |
| Nomic-embed-text | PMC-Patients | natural_language | 4 | 0.3333 | 0.2370 | 0.0930 | 0.1767 | 0.3026 | 0.5300 | 0.2576 | 0.0504 | 16.7533 | 0.9962 | 0.1567 |
| Nomic-embed-text | PMC-Patients | natural_language | 5 | 0.4167 | 0.3178 | 0.1928 | 0.2448 | 0.3948 | 0.6000 | 0.3014 | 0.1190 | 18.1841 | 0.9950 | 0.1402 |
| Nomic-embed-text | PMC-Patients | natural_language | 6 | 0.5000 | 0.2029 | 0.0508 | 0.1451 | 0.2648 | 0.4900 | 0.2255 | 0.0000 | 15.8651 | 0.9950 | 0.1658 |
| Nomic-embed-text | PMC-Patients | natural_language | 7 | 0.5833 | 0.1618 | 0.0000 | 0.1103 | 0.2174 | 0.4200 | 0.2638 | 0.0601 | 17.7932 | 0.9923 | 0.1550 |
| Nomic-embed-text | PMC-Patients | natural_language | 8 | 0.6667 | 0.2423 | 0.0995 | 0.1816 | 0.3051 | 0.5500 | 0.3554 | 0.2037 | 19.6301 | 0.9972 | 0.1478 |
| Nomic-embed-text | PMC-Patients | natural_language | 9 | 0.7500 | 0.3236 | 0.2000 | 0.2465 | 0.3991 | 0.5600 | 0.2555 | 0.0471 | 18.1974 | 0.9970 | 0.1575 |
| Nomic-embed-text | PMC-Patients | natural_language | 10 | 0.8333 | 0.3767 | 0.2656 | 0.2946 | 0.4615 | 0.5900 | 0.2427 | 0.0270 | 19.3396 | 0.9984 | 0.1485 |
| Nomic-embed-text | PMC-Patients | natural_language | 11 | 0.9167 | 0.8051 | 0.7952 | 0.7366 | 0.8705 | 0.9400 | 0.7441 | 0.8133 | 28.0169 | 0.9956 | 0.1148 |
| Nomic-embed-text | PMC-Patients | natural_language | 12 | 1.0000 | 0.9708 | 1.0000 | 0.9442 | 0.9950 | 1.0000 | 0.8631 | 1.0000 | 43.1663 | 0.6605 | 0.0709 |
| Nomic-embed-text | Synthetic | keyword | 0 | 0.0000 | 0.3757 | 0.3159 | 0.3029 | 0.4514 | 0.7000 | 0.4614 | 0.3701 | 17.8218 | 0.9978 | 0.1706 |
| Nomic-embed-text | Synthetic | keyword | 1 | 0.0833 | 0.4863 | 0.5466 | 0.4154 | 0.5624 | 0.8200 | 0.5446 | 0.5005 | 18.1419 | 0.9971 | 0.1682 |
| Nomic-embed-text | Synthetic | keyword | 2 | 0.1667 | 0.4758 | 0.5247 | 0.3986 | 0.5522 | 0.7700 | 0.4378 | 0.3330 | 15.2133 | 0.9986 | 0.1965 |
| Nomic-embed-text | Synthetic | keyword | 3 | 0.2500 | 0.3687 | 0.3013 | 0.2931 | 0.4476 | 0.6500 | 0.3300 | 0.1638 | 12.2133 | 0.9986 | 0.2344 |
| Nomic-embed-text | Synthetic | keyword | 4 | 0.3333 | 0.2331 | 0.0184 | 0.1642 | 0.3066 | 0.4500 | 0.2576 | 0.0504 | 10.8727 | 0.9981 | 0.2575 |
| Nomic-embed-text | Synthetic | keyword | 5 | 0.4167 | 0.2900 | 0.1371 | 0.2213 | 0.3638 | 0.5300 | 0.3014 | 0.1190 | 11.6077 | 0.9976 | 0.2426 |
| Nomic-embed-text | Synthetic | keyword | 6 | 0.5000 | 0.2321 | 0.0163 | 0.1686 | 0.2998 | 0.4700 | 0.2255 | 0.0000 | 9.9889 | 0.9976 | 0.2736 |
| Nomic-embed-text | Synthetic | keyword | 7 | 0.5833 | 0.3124 | 0.1838 | 0.2449 | 0.3819 | 0.6300 | 0.2638 | 0.0601 | 12.9021 | 0.9964 | 0.2175 |
| Nomic-embed-text | Synthetic | keyword | 8 | 0.6667 | 0.3895 | 0.3447 | 0.3196 | 0.4643 | 0.7000 | 0.3554 | 0.2037 | 15.2235 | 0.9987 | 0.1837 |
| Nomic-embed-text | Synthetic | keyword | 9 | 0.7500 | 0.2587 | 0.0718 | 0.1925 | 0.3319 | 0.5400 | 0.2555 | 0.0471 | 14.9247 | 0.9985 | 0.1828 |
| Nomic-embed-text | Synthetic | keyword | 10 | 0.8333 | 0.2243 | 0.0000 | 0.1618 | 0.2900 | 0.5100 | 0.2427 | 0.0270 | 16.9912 | 0.9992 | 0.1472 |
| Nomic-embed-text | Synthetic | keyword | 11 | 0.9167 | 0.5724 | 0.7263 | 0.4853 | 0.6504 | 0.8200 | 0.7441 | 0.8133 | 17.7218 | 0.9970 | 0.1292 |
| Nomic-embed-text | Synthetic | keyword | 12 | 1.0000 | 0.7036 | 1.0000 | 0.6215 | 0.7704 | 0.9400 | 0.8631 | 1.0000 | 19.8625 | 0.7387 | 0.1167 |
| Nomic-embed-text | Synthetic | natural_language | 0 | 0.0000 | 0.3702 | 0.2529 | 0.2965 | 0.4447 | 0.6900 | 0.4614 | 0.3701 | 17.8218 | 0.9978 | 0.1706 |
| Nomic-embed-text | Synthetic | natural_language | 1 | 0.0833 | 0.4543 | 0.4409 | 0.3696 | 0.5351 | 0.7300 | 0.5446 | 0.5005 | 18.1419 | 0.9971 | 0.1682 |
| Nomic-embed-text | Synthetic | natural_language | 2 | 0.1667 | 0.4059 | 0.3327 | 0.3233 | 0.4869 | 0.6300 | 0.4378 | 0.3330 | 15.2133 | 0.9986 | 0.1965 |
| Nomic-embed-text | Synthetic | natural_language | 3 | 0.2500 | 0.3619 | 0.2343 | 0.2831 | 0.4450 | 0.6000 | 0.3300 | 0.1638 | 12.2133 | 0.9986 | 0.2344 |
| Nomic-embed-text | Synthetic | natural_language | 4 | 0.3333 | 0.3466 | 0.2001 | 0.2720 | 0.4266 | 0.5700 | 0.2576 | 0.0504 | 10.8727 | 0.9981 | 0.2575 |
| Nomic-embed-text | Synthetic | natural_language | 5 | 0.4167 | 0.3633 | 0.2374 | 0.2844 | 0.4394 | 0.6200 | 0.3014 | 0.1190 | 11.6077 | 0.9976 | 0.2426 |
| Nomic-embed-text | Synthetic | natural_language | 6 | 0.5000 | 0.2929 | 0.0800 | 0.2236 | 0.3676 | 0.5900 | 0.2255 | 0.0000 | 9.9889 | 0.9976 | 0.2736 |
| Nomic-embed-text | Synthetic | natural_language | 7 | 0.5833 | 0.3789 | 0.2723 | 0.2997 | 0.4624 | 0.6000 | 0.2638 | 0.0601 | 12.9021 | 0.9964 | 0.2175 |
| Nomic-embed-text | Synthetic | natural_language | 8 | 0.6667 | 0.4210 | 0.3664 | 0.3372 | 0.5037 | 0.6400 | 0.3554 | 0.2037 | 15.2235 | 0.9987 | 0.1837 |
| Nomic-embed-text | Synthetic | natural_language | 9 | 0.7500 | 0.3336 | 0.1710 | 0.2644 | 0.4051 | 0.6500 | 0.2555 | 0.0471 | 14.9247 | 0.9985 | 0.1828 |
| Nomic-embed-text | Synthetic | natural_language | 10 | 0.8333 | 0.2571 | 0.0000 | 0.1920 | 0.3249 | 0.5900 | 0.2427 | 0.0270 | 16.9912 | 0.9992 | 0.1472 |
| Nomic-embed-text | Synthetic | natural_language | 11 | 0.9167 | 0.6122 | 0.7939 | 0.5370 | 0.6914 | 0.8800 | 0.7441 | 0.8133 | 17.7218 | 0.9970 | 0.1292 |
| Nomic-embed-text | Synthetic | natural_language | 12 | 1.0000 | 0.7044 | 1.0000 | 0.6249 | 0.7617 | 0.9400 | 0.8631 | 1.0000 | 19.8625 | 0.7387 | 0.1167 |
| Nomic-embed-text-nopfx | MTSamples | keyword | 0 | 0.0000 | 0.4442 | 0.4456 | 0.3578 | 0.5272 | 0.6500 | 0.4723 | 0.4324 | 22.9620 | 0.9956 | 0.1295 |
| Nomic-embed-text-nopfx | MTSamples | keyword | 1 | 0.0833 | 0.5909 | 0.6217 | 0.5139 | 0.6705 | 0.8300 | 0.5351 | 0.5235 | 22.7269 | 0.9940 | 0.1259 |
| Nomic-embed-text-nopfx | MTSamples | keyword | 2 | 0.1667 | 0.4544 | 0.4579 | 0.3676 | 0.5437 | 0.6300 | 0.4326 | 0.3748 | 17.8307 | 0.9969 | 0.1611 |
| Nomic-embed-text-nopfx | MTSamples | keyword | 3 | 0.2500 | 0.1352 | 0.0748 | 0.0900 | 0.1874 | 0.3700 | 0.2632 | 0.1288 | 14.0254 | 0.9965 | 0.1842 |
| Nomic-embed-text-nopfx | MTSamples | keyword | 4 | 0.3333 | 0.1009 | 0.0336 | 0.0659 | 0.1410 | 0.3100 | 0.2118 | 0.0543 | 12.9123 | 0.9950 | 0.2049 |
| Nomic-embed-text-nopfx | MTSamples | keyword | 5 | 0.4167 | 0.1169 | 0.0528 | 0.0781 | 0.1682 | 0.3400 | 0.2318 | 0.0832 | 12.1940 | 0.9935 | 0.2190 |
| Nomic-embed-text-nopfx | MTSamples | keyword | 6 | 0.5000 | 0.0729 | 0.0000 | 0.0384 | 0.1048 | 0.2400 | 0.1745 | 0.0000 | 10.8530 | 0.9934 | 0.2302 |
| Nomic-embed-text-nopfx | MTSamples | keyword | 7 | 0.5833 | 0.1499 | 0.0924 | 0.1050 | 0.2097 | 0.4300 | 0.2123 | 0.0549 | 12.1682 | 0.9900 | 0.2207 |
| Nomic-embed-text-nopfx | MTSamples | keyword | 8 | 0.6667 | 0.3499 | 0.3325 | 0.2695 | 0.4285 | 0.6500 | 0.3440 | 0.2461 | 12.4232 | 0.9962 | 0.2211 |
| Nomic-embed-text-nopfx | MTSamples | keyword | 9 | 0.7500 | 0.1437 | 0.0850 | 0.0929 | 0.2014 | 0.3200 | 0.2204 | 0.0668 | 12.0089 | 0.9961 | 0.2240 |
| Nomic-embed-text-nopfx | MTSamples | keyword | 10 | 0.8333 | 0.1136 | 0.0488 | 0.0694 | 0.1639 | 0.2800 | 0.2051 | 0.0445 | 12.4085 | 0.9980 | 0.2163 |
| Nomic-embed-text-nopfx | MTSamples | keyword | 11 | 0.9167 | 0.8045 | 0.8781 | 0.7420 | 0.8734 | 0.9200 | 0.7497 | 0.8352 | 18.9540 | 0.9941 | 0.1763 |
| Nomic-embed-text-nopfx | MTSamples | keyword | 12 | 1.0000 | 0.9061 | 1.0000 | 0.8544 | 0.9500 | 0.9700 | 0.8633 | 1.0000 | 29.7551 | 0.6027 | 0.1254 |
| Nomic-embed-text-nopfx | MTSamples | natural_language | 0 | 0.0000 | 0.6832 | 0.6646 | 0.5982 | 0.7644 | 0.8300 | 0.4723 | 0.4324 | 22.9620 | 0.9956 | 0.1295 |
| Nomic-embed-text-nopfx | MTSamples | natural_language | 1 | 0.0833 | 0.6709 | 0.6484 | 0.5896 | 0.7460 | 0.8500 | 0.5351 | 0.5235 | 22.7269 | 0.9940 | 0.1259 |
| Nomic-embed-text-nopfx | MTSamples | natural_language | 2 | 0.1667 | 0.5085 | 0.4347 | 0.4266 | 0.5915 | 0.7700 | 0.4326 | 0.3748 | 17.8307 | 0.9969 | 0.1611 |
| Nomic-embed-text-nopfx | MTSamples | natural_language | 3 | 0.2500 | 0.2981 | 0.1578 | 0.2347 | 0.3681 | 0.6500 | 0.2632 | 0.1288 | 14.0254 | 0.9965 | 0.1842 |
| Nomic-embed-text-nopfx | MTSamples | natural_language | 4 | 0.3333 | 0.2616 | 0.1098 | 0.2020 | 0.3240 | 0.6400 | 0.2118 | 0.0543 | 12.9123 | 0.9950 | 0.2049 |
| Nomic-embed-text-nopfx | MTSamples | natural_language | 5 | 0.4167 | 0.2647 | 0.1138 | 0.1970 | 0.3292 | 0.6400 | 0.2318 | 0.0832 | 12.1940 | 0.9935 | 0.2190 |
| Nomic-embed-text-nopfx | MTSamples | natural_language | 6 | 0.5000 | 0.1782 | 0.0000 | 0.1255 | 0.2346 | 0.4700 | 0.1745 | 0.0000 | 10.8530 | 0.9934 | 0.2302 |
| Nomic-embed-text-nopfx | MTSamples | natural_language | 7 | 0.5833 | 0.1866 | 0.0111 | 0.1343 | 0.2473 | 0.4600 | 0.2123 | 0.0549 | 12.1682 | 0.9900 | 0.2207 |
| Nomic-embed-text-nopfx | MTSamples | natural_language | 8 | 0.6667 | 0.3050 | 0.1669 | 0.2317 | 0.3806 | 0.5900 | 0.3440 | 0.2461 | 12.4232 | 0.9962 | 0.2211 |
| Nomic-embed-text-nopfx | MTSamples | natural_language | 9 | 0.7500 | 0.1877 | 0.0125 | 0.1289 | 0.2508 | 0.3900 | 0.2204 | 0.0668 | 12.0089 | 0.9961 | 0.2240 |
| Nomic-embed-text-nopfx | MTSamples | natural_language | 10 | 0.8333 | 0.1922 | 0.0184 | 0.1312 | 0.2570 | 0.3900 | 0.2051 | 0.0445 | 12.4085 | 0.9980 | 0.2163 |
| Nomic-embed-text-nopfx | MTSamples | natural_language | 11 | 0.9167 | 0.8781 | 0.9210 | 0.8262 | 0.9334 | 0.9500 | 0.7497 | 0.8352 | 18.9540 | 0.9941 | 0.1763 |
| Nomic-embed-text-nopfx | MTSamples | natural_language | 12 | 1.0000 | 0.9381 | 1.0000 | 0.8871 | 0.9717 | 0.9800 | 0.8633 | 1.0000 | 29.7551 | 0.6027 | 0.1254 |
| Nomic-embed-text-nopfx | PMC-Patients | keyword | 0 | 0.0000 | 0.3870 | 0.2888 | 0.2968 | 0.4738 | 0.5300 | 0.4723 | 0.4324 | 28.0950 | 0.9965 | 0.1235 |
| Nomic-embed-text-nopfx | PMC-Patients | keyword | 1 | 0.0833 | 0.4642 | 0.3849 | 0.3780 | 0.5565 | 0.6300 | 0.5351 | 0.5235 | 27.0457 | 0.9950 | 0.1281 |
| Nomic-embed-text-nopfx | PMC-Patients | keyword | 2 | 0.1667 | 0.3935 | 0.2969 | 0.3080 | 0.4811 | 0.5700 | 0.4326 | 0.3748 | 21.0209 | 0.9974 | 0.1493 |
| Nomic-embed-text-nopfx | PMC-Patients | keyword | 3 | 0.2500 | 0.2349 | 0.0996 | 0.1680 | 0.3049 | 0.4100 | 0.2632 | 0.1288 | 17.1856 | 0.9970 | 0.1621 |
| Nomic-embed-text-nopfx | PMC-Patients | keyword | 4 | 0.3333 | 0.1930 | 0.0475 | 0.1322 | 0.2595 | 0.3800 | 0.2118 | 0.0543 | 17.4213 | 0.9959 | 0.1547 |
| Nomic-embed-text-nopfx | PMC-Patients | keyword | 5 | 0.4167 | 0.2199 | 0.0810 | 0.1565 | 0.2921 | 0.4300 | 0.2318 | 0.0832 | 18.6881 | 0.9948 | 0.1413 |
| Nomic-embed-text-nopfx | PMC-Patients | keyword | 6 | 0.5000 | 0.1548 | 0.0000 | 0.1018 | 0.2114 | 0.3800 | 0.1745 | 0.0000 | 17.1715 | 0.9947 | 0.1527 |
| Nomic-embed-text-nopfx | PMC-Patients | keyword | 7 | 0.5833 | 0.2391 | 0.1049 | 0.1715 | 0.3140 | 0.4700 | 0.2123 | 0.0549 | 19.5303 | 0.9915 | 0.1445 |
| Nomic-embed-text-nopfx | PMC-Patients | keyword | 8 | 0.6667 | 0.3564 | 0.2508 | 0.2774 | 0.4381 | 0.5800 | 0.3440 | 0.2461 | 20.1851 | 0.9968 | 0.1450 |
| Nomic-embed-text-nopfx | PMC-Patients | keyword | 9 | 0.7500 | 0.2309 | 0.0947 | 0.1674 | 0.3017 | 0.5000 | 0.2204 | 0.0668 | 18.0799 | 0.9966 | 0.1627 |
| Nomic-embed-text-nopfx | PMC-Patients | keyword | 10 | 0.8333 | 0.2085 | 0.0668 | 0.1484 | 0.2725 | 0.5000 | 0.2051 | 0.0445 | 20.8311 | 0.9982 | 0.1369 |
| Nomic-embed-text-nopfx | PMC-Patients | keyword | 11 | 0.9167 | 0.7748 | 0.7712 | 0.7105 | 0.8368 | 0.9800 | 0.7497 | 0.8352 | 27.8896 | 0.9945 | 0.1046 |
| Nomic-embed-text-nopfx | PMC-Patients | keyword | 12 | 1.0000 | 0.9587 | 1.0000 | 0.9267 | 0.9870 | 1.0000 | 0.8633 | 1.0000 | 40.5760 | 0.5898 | 0.0701 |
| Nomic-embed-text-nopfx | PMC-Patients | natural_language | 0 | 0.0000 | 0.6008 | 0.5263 | 0.5114 | 0.6892 | 0.7600 | 0.4723 | 0.4324 | 28.0950 | 0.9965 | 0.1235 |
| Nomic-embed-text-nopfx | PMC-Patients | natural_language | 1 | 0.0833 | 0.6108 | 0.5386 | 0.5274 | 0.6949 | 0.7900 | 0.5351 | 0.5235 | 27.0457 | 0.9950 | 0.1281 |
| Nomic-embed-text-nopfx | PMC-Patients | natural_language | 2 | 0.1667 | 0.4226 | 0.3065 | 0.3410 | 0.5042 | 0.6500 | 0.4326 | 0.3748 | 21.0209 | 0.9974 | 0.1493 |
| Nomic-embed-text-nopfx | PMC-Patients | natural_language | 3 | 0.2500 | 0.3281 | 0.1900 | 0.2529 | 0.4044 | 0.5500 | 0.2632 | 0.1288 | 17.1856 | 0.9970 | 0.1621 |
| Nomic-embed-text-nopfx | PMC-Patients | natural_language | 4 | 0.3333 | 0.2395 | 0.0808 | 0.1769 | 0.3104 | 0.5100 | 0.2118 | 0.0543 | 17.4213 | 0.9959 | 0.1547 |
| Nomic-embed-text-nopfx | PMC-Patients | natural_language | 5 | 0.4167 | 0.3216 | 0.1820 | 0.2405 | 0.4000 | 0.5900 | 0.2318 | 0.0832 | 18.6881 | 0.9948 | 0.1413 |
| Nomic-embed-text-nopfx | PMC-Patients | natural_language | 6 | 0.5000 | 0.2122 | 0.0471 | 0.1519 | 0.2791 | 0.4900 | 0.1745 | 0.0000 | 17.1715 | 0.9947 | 0.1527 |
| Nomic-embed-text-nopfx | PMC-Patients | natural_language | 7 | 0.5833 | 0.1740 | 0.0000 | 0.1185 | 0.2391 | 0.4600 | 0.2123 | 0.0549 | 19.5303 | 0.9915 | 0.1445 |
| Nomic-embed-text-nopfx | PMC-Patients | natural_language | 8 | 0.6667 | 0.2525 | 0.0968 | 0.1882 | 0.3221 | 0.5600 | 0.3440 | 0.2461 | 20.1851 | 0.9968 | 0.1450 |
| Nomic-embed-text-nopfx | PMC-Patients | natural_language | 9 | 0.7500 | 0.2096 | 0.0439 | 0.1508 | 0.2715 | 0.4700 | 0.2204 | 0.0668 | 18.0799 | 0.9966 | 0.1627 |
| Nomic-embed-text-nopfx | PMC-Patients | natural_language | 10 | 0.8333 | 0.2332 | 0.0730 | 0.1761 | 0.2941 | 0.5900 | 0.2051 | 0.0445 | 20.8311 | 0.9982 | 0.1369 |
| Nomic-embed-text-nopfx | PMC-Patients | natural_language | 11 | 0.9167 | 0.8709 | 0.8593 | 0.8172 | 0.9210 | 1.0000 | 0.7497 | 0.8352 | 27.8896 | 0.9945 | 0.1046 |
| Nomic-embed-text-nopfx | PMC-Patients | natural_language | 12 | 1.0000 | 0.9850 | 1.0000 | 0.9650 | 1.0000 | 1.0000 | 0.8633 | 1.0000 | 40.5760 | 0.5898 | 0.0701 |
| Nomic-embed-text-nopfx | Synthetic | keyword | 0 | 0.0000 | 0.3600 | 0.3799 | 0.2937 | 0.4334 | 0.7100 | 0.4723 | 0.4324 | 17.8670 | 0.9978 | 0.1707 |
| Nomic-embed-text-nopfx | Synthetic | keyword | 1 | 0.0833 | 0.4366 | 0.5200 | 0.3640 | 0.5071 | 0.8100 | 0.5351 | 0.5235 | 18.1169 | 0.9971 | 0.1687 |
| Nomic-embed-text-nopfx | Synthetic | keyword | 2 | 0.1667 | 0.3932 | 0.4406 | 0.3230 | 0.4638 | 0.7500 | 0.4326 | 0.3748 | 14.9600 | 0.9986 | 0.1995 |
| Nomic-embed-text-nopfx | Synthetic | keyword | 3 | 0.2500 | 0.2276 | 0.1376 | 0.1649 | 0.2923 | 0.4500 | 0.2632 | 0.1288 | 12.2462 | 0.9985 | 0.2339 |
| Nomic-embed-text-nopfx | Synthetic | keyword | 4 | 0.3333 | 0.1524 | 0.0000 | 0.1002 | 0.2116 | 0.3200 | 0.2118 | 0.0543 | 11.1594 | 0.9979 | 0.2525 |
| Nomic-embed-text-nopfx | Synthetic | keyword | 5 | 0.4167 | 0.1769 | 0.0448 | 0.1217 | 0.2351 | 0.3800 | 0.2318 | 0.0832 | 12.2685 | 0.9975 | 0.2317 |
| Nomic-embed-text-nopfx | Synthetic | keyword | 6 | 0.5000 | 0.1582 | 0.0106 | 0.1020 | 0.2168 | 0.3000 | 0.1745 | 0.0000 | 10.9704 | 0.9975 | 0.2548 |
| Nomic-embed-text-nopfx | Synthetic | keyword | 7 | 0.5833 | 0.2153 | 0.1151 | 0.1618 | 0.2745 | 0.5100 | 0.2123 | 0.0549 | 12.7578 | 0.9962 | 0.2209 |
| Nomic-embed-text-nopfx | Synthetic | keyword | 8 | 0.6667 | 0.3634 | 0.3861 | 0.2962 | 0.4388 | 0.7000 | 0.3440 | 0.2461 | 14.3804 | 0.9985 | 0.1951 |
| Nomic-embed-text-nopfx | Synthetic | keyword | 9 | 0.7500 | 0.2795 | 0.2326 | 0.2118 | 0.3504 | 0.5700 | 0.2204 | 0.0668 | 14.5906 | 0.9984 | 0.1851 |
| Nomic-embed-text-nopfx | Synthetic | keyword | 10 | 0.8333 | 0.2653 | 0.2066 | 0.2012 | 0.3360 | 0.5600 | 0.2051 | 0.0445 | 16.2453 | 0.9991 | 0.1506 |
| Nomic-embed-text-nopfx | Synthetic | keyword | 11 | 0.9167 | 0.5814 | 0.7850 | 0.5031 | 0.6576 | 0.8500 | 0.7497 | 0.8352 | 16.1435 | 0.9966 | 0.1467 |
| Nomic-embed-text-nopfx | Synthetic | keyword | 12 | 1.0000 | 0.6989 | 1.0000 | 0.6422 | 0.7764 | 0.9500 | 0.8633 | 1.0000 | 18.6531 | 0.7225 | 0.1296 |
| Nomic-embed-text-nopfx | Synthetic | natural_language | 0 | 0.0000 | 0.3585 | 0.2961 | 0.2894 | 0.4296 | 0.6800 | 0.4723 | 0.4324 | 17.8670 | 0.9978 | 0.1707 |
| Nomic-embed-text-nopfx | Synthetic | natural_language | 1 | 0.0833 | 0.4370 | 0.4614 | 0.3593 | 0.5187 | 0.6900 | 0.5351 | 0.5235 | 18.1169 | 0.9971 | 0.1687 |
| Nomic-embed-text-nopfx | Synthetic | natural_language | 2 | 0.1667 | 0.4236 | 0.4332 | 0.3460 | 0.5058 | 0.6600 | 0.4326 | 0.3748 | 14.9600 | 0.9986 | 0.1995 |
| Nomic-embed-text-nopfx | Synthetic | natural_language | 3 | 0.2500 | 0.3553 | 0.2894 | 0.2755 | 0.4319 | 0.6500 | 0.2632 | 0.1288 | 12.2462 | 0.9985 | 0.2339 |
| Nomic-embed-text-nopfx | Synthetic | natural_language | 4 | 0.3333 | 0.3237 | 0.2229 | 0.2458 | 0.4060 | 0.6000 | 0.2118 | 0.0543 | 11.1594 | 0.9979 | 0.2525 |
| Nomic-embed-text-nopfx | Synthetic | natural_language | 5 | 0.4167 | 0.2906 | 0.1532 | 0.2218 | 0.3662 | 0.5900 | 0.2318 | 0.0832 | 12.2685 | 0.9975 | 0.2317 |
| Nomic-embed-text-nopfx | Synthetic | natural_language | 6 | 0.5000 | 0.2705 | 0.1109 | 0.2070 | 0.3417 | 0.5100 | 0.1745 | 0.0000 | 10.9704 | 0.9975 | 0.2548 |
| Nomic-embed-text-nopfx | Synthetic | natural_language | 7 | 0.5833 | 0.3089 | 0.1917 | 0.2352 | 0.3838 | 0.5800 | 0.2123 | 0.0549 | 12.7578 | 0.9962 | 0.2209 |
| Nomic-embed-text-nopfx | Synthetic | natural_language | 8 | 0.6667 | 0.4369 | 0.4612 | 0.3553 | 0.5214 | 0.6700 | 0.3440 | 0.2461 | 14.3804 | 0.9985 | 0.1951 |
| Nomic-embed-text-nopfx | Synthetic | natural_language | 9 | 0.7500 | 0.2713 | 0.1126 | 0.2029 | 0.3466 | 0.5200 | 0.2204 | 0.0668 | 14.5906 | 0.9984 | 0.1851 |
| Nomic-embed-text-nopfx | Synthetic | natural_language | 10 | 0.8333 | 0.2178 | 0.0000 | 0.1587 | 0.2862 | 0.4800 | 0.2051 | 0.0445 | 16.2453 | 0.9991 | 0.1506 |
| Nomic-embed-text-nopfx | Synthetic | natural_language | 11 | 0.9167 | 0.5887 | 0.7807 | 0.5125 | 0.6712 | 0.8700 | 0.7497 | 0.8352 | 16.1435 | 0.9966 | 0.1467 |
| Nomic-embed-text-nopfx | Synthetic | natural_language | 12 | 1.0000 | 0.6929 | 1.0000 | 0.6353 | 0.7760 | 0.9300 | 0.8633 | 1.0000 | 18.6531 | 0.7225 | 0.1296 |
| Phi-3-mini | MTSamples | keyword | 0 | 0.0000 | 0.4631 | 0.8465 | 0.3872 | 0.5492 | 0.7200 | 0.5515 | 1.0000 | 28.8689 | 0.6932 | 0.1268 |
| Phi-3-mini | MTSamples | keyword | 1 | 0.0312 | 0.1594 | 0.2563 | 0.1038 | 0.2197 | 0.3500 | 0.2377 | 0.3991 | 18.1550 | 0.9956 | 0.1620 |
| Phi-3-mini | MTSamples | keyword | 2 | 0.0625 | 0.0396 | 0.0235 | 0.0200 | 0.0599 | 0.1800 | 0.1154 | 0.1648 | 11.3902 | 0.9929 | 0.2233 |
| Phi-3-mini | MTSamples | keyword | 3 | 0.0938 | 0.0293 | 0.0035 | 0.0097 | 0.0556 | 0.1000 | 0.0293 | 0.0000 | 1.3498 | 0.9640 | 0.8598 |
| Phi-3-mini | MTSamples | keyword | 4 | 0.1250 | 0.0293 | 0.0035 | 0.0096 | 0.0579 | 0.1000 | 0.0296 | 0.0007 | 1.5342 | 0.9590 | 0.8058 |
| Phi-3-mini | MTSamples | keyword | 5 | 0.1562 | 0.0293 | 0.0035 | 0.0100 | 0.0572 | 0.1000 | 0.0307 | 0.0028 | 1.4666 | 0.9572 | 0.8244 |
| Phi-3-mini | MTSamples | keyword | 6 | 0.1875 | 0.0293 | 0.0035 | 0.0093 | 0.0561 | 0.1000 | 0.0304 | 0.0023 | 1.7136 | 0.9532 | 0.7611 |
| Phi-3-mini | MTSamples | keyword | 7 | 0.2188 | 0.0293 | 0.0035 | 0.0098 | 0.0571 | 0.1000 | 0.0304 | 0.0022 | 2.0398 | 0.9503 | 0.6948 |
| Phi-3-mini | MTSamples | keyword | 8 | 0.2500 | 0.0303 | 0.0054 | 0.0095 | 0.0570 | 0.1100 | 0.0313 | 0.0039 | 2.9425 | 0.9512 | 0.5677 |
| Phi-3-mini | MTSamples | keyword | 9 | 0.2812 | 0.0293 | 0.0035 | 0.0091 | 0.0560 | 0.1000 | 0.0314 | 0.0041 | 3.3914 | 0.9561 | 0.5209 |
| Phi-3-mini | MTSamples | keyword | 10 | 0.3125 | 0.0303 | 0.0054 | 0.0106 | 0.0577 | 0.1100 | 0.0315 | 0.0043 | 3.7059 | 0.9571 | 0.4918 |
| Phi-3-mini | MTSamples | keyword | 11 | 0.3438 | 0.0293 | 0.0035 | 0.0089 | 0.0572 | 0.1000 | 0.0317 | 0.0047 | 4.1274 | 0.9594 | 0.4601 |
| Phi-3-mini | MTSamples | keyword | 12 | 0.3750 | 0.0293 | 0.0035 | 0.0097 | 0.0565 | 0.1000 | 0.0311 | 0.0036 | 4.7594 | 0.9586 | 0.4145 |
| Phi-3-mini | MTSamples | keyword | 13 | 0.4062 | 0.0293 | 0.0035 | 0.0092 | 0.0564 | 0.1000 | 0.0312 | 0.0037 | 5.3029 | 0.9588 | 0.3845 |
| Phi-3-mini | MTSamples | keyword | 14 | 0.4375 | 0.0295 | 0.0039 | 0.0089 | 0.0568 | 0.1000 | 0.0321 | 0.0055 | 6.4930 | 0.9691 | 0.3405 |
| Phi-3-mini | MTSamples | keyword | 15 | 0.4688 | 0.0281 | 0.0012 | 0.0090 | 0.0542 | 0.1000 | 0.0330 | 0.0071 | 7.2702 | 0.9684 | 0.3048 |
| Phi-3-mini | MTSamples | keyword | 16 | 0.5000 | 0.0284 | 0.0017 | 0.0095 | 0.0533 | 0.1000 | 0.0325 | 0.0063 | 7.9113 | 0.9675 | 0.2795 |
| Phi-3-mini | MTSamples | keyword | 17 | 0.5312 | 0.0282 | 0.0014 | 0.0096 | 0.0534 | 0.1000 | 0.0335 | 0.0081 | 8.3587 | 0.9668 | 0.2618 |
| Phi-3-mini | MTSamples | keyword | 18 | 0.5625 | 0.0280 | 0.0010 | 0.0102 | 0.0532 | 0.1000 | 0.0362 | 0.0134 | 9.6634 | 0.9618 | 0.2394 |
| Phi-3-mini | MTSamples | keyword | 19 | 0.5938 | 0.0283 | 0.0016 | 0.0091 | 0.0544 | 0.1000 | 0.0399 | 0.0203 | 10.5382 | 0.9600 | 0.2363 |
| Phi-3-mini | MTSamples | keyword | 20 | 0.6250 | 0.0275 | 0.0000 | 0.0096 | 0.0543 | 0.1000 | 0.0403 | 0.0211 | 10.9121 | 0.9610 | 0.2298 |
| Phi-3-mini | MTSamples | keyword | 21 | 0.6562 | 0.0292 | 0.0033 | 0.0104 | 0.0558 | 0.1000 | 0.0443 | 0.0288 | 11.2292 | 0.9583 | 0.2352 |
| Phi-3-mini | MTSamples | keyword | 22 | 0.6875 | 0.0302 | 0.0052 | 0.0110 | 0.0572 | 0.1100 | 0.0496 | 0.0389 | 10.8722 | 0.9578 | 0.2473 |
| Phi-3-mini | MTSamples | keyword | 23 | 0.7188 | 0.0386 | 0.0216 | 0.0176 | 0.0641 | 0.1600 | 0.0816 | 0.1003 | 10.0515 | 0.9427 | 0.2713 |
| Phi-3-mini | MTSamples | keyword | 24 | 0.7500 | 0.0509 | 0.0455 | 0.0262 | 0.0790 | 0.1900 | 0.1166 | 0.1672 | 10.9640 | 0.9360 | 0.2537 |
| Phi-3-mini | MTSamples | keyword | 25 | 0.7812 | 0.0789 | 0.0999 | 0.0546 | 0.1036 | 0.3600 | 0.1832 | 0.2947 | 10.7372 | 0.9280 | 0.2575 |
| Phi-3-mini | MTSamples | keyword | 26 | 0.8125 | 0.0979 | 0.1368 | 0.0701 | 0.1256 | 0.4400 | 0.2006 | 0.3280 | 11.0064 | 0.9353 | 0.2528 |
| Phi-3-mini | MTSamples | keyword | 27 | 0.8438 | 0.1144 | 0.1689 | 0.0884 | 0.1431 | 0.5100 | 0.2244 | 0.3736 | 10.8788 | 0.9342 | 0.2550 |
| Phi-3-mini | MTSamples | keyword | 28 | 0.8750 | 0.1284 | 0.1961 | 0.0979 | 0.1627 | 0.5200 | 0.2389 | 0.4015 | 10.9800 | 0.9404 | 0.2549 |
| Phi-3-mini | MTSamples | keyword | 29 | 0.9062 | 0.1403 | 0.2192 | 0.1047 | 0.1736 | 0.5400 | 0.2532 | 0.4287 | 11.4821 | 0.9425 | 0.2481 |
| Phi-3-mini | MTSamples | keyword | 30 | 0.9375 | 0.4664 | 0.8529 | 0.3860 | 0.5411 | 0.7700 | 0.4371 | 0.7809 | 11.6639 | 0.9478 | 0.2470 |
| Phi-3-mini | MTSamples | keyword | 31 | 0.9688 | 0.5061 | 0.9300 | 0.4200 | 0.5794 | 0.8100 | 0.4482 | 0.8021 | 12.4429 | 0.9567 | 0.2354 |
| Phi-3-mini | MTSamples | keyword | 32 | 1.0000 | 0.5421 | 1.0000 | 0.4692 | 0.6256 | 0.8300 | 0.5216 | 0.9427 | 12.5823 | 0.9736 | 0.2300 |
| Phi-3-mini | MTSamples | natural_language | 0 | 0.0000 | 0.6152 | 1.0000 | 0.5314 | 0.6980 | 0.8100 | 0.5515 | 1.0000 | 28.8689 | 0.6932 | 0.1268 |
| Phi-3-mini | MTSamples | natural_language | 1 | 0.0312 | 0.2319 | 0.3458 | 0.1615 | 0.3032 | 0.4000 | 0.2377 | 0.3991 | 18.1550 | 0.9956 | 0.1620 |
| Phi-3-mini | MTSamples | natural_language | 2 | 0.0625 | 0.0855 | 0.0959 | 0.0451 | 0.1323 | 0.2200 | 0.1154 | 0.1648 | 11.3902 | 0.9929 | 0.2233 |
| Phi-3-mini | MTSamples | natural_language | 3 | 0.0938 | 0.0293 | 0.0000 | 0.0091 | 0.0575 | 0.1000 | 0.0293 | 0.0000 | 1.3498 | 0.9640 | 0.8598 |
| Phi-3-mini | MTSamples | natural_language | 4 | 0.1250 | 0.0295 | 0.0003 | 0.0097 | 0.0564 | 0.1000 | 0.0296 | 0.0007 | 1.5342 | 0.9590 | 0.8058 |
| Phi-3-mini | MTSamples | natural_language | 5 | 0.1562 | 0.0294 | 0.0002 | 0.0104 | 0.0572 | 0.1000 | 0.0307 | 0.0028 | 1.4666 | 0.9572 | 0.8244 |
| Phi-3-mini | MTSamples | natural_language | 6 | 0.1875 | 0.0293 | 0.0000 | 0.0096 | 0.0561 | 0.1000 | 0.0304 | 0.0023 | 1.7136 | 0.9532 | 0.7611 |
| Phi-3-mini | MTSamples | natural_language | 7 | 0.2188 | 0.0296 | 0.0005 | 0.0098 | 0.0549 | 0.1000 | 0.0304 | 0.0022 | 2.0398 | 0.9503 | 0.6948 |
| Phi-3-mini | MTSamples | natural_language | 8 | 0.2500 | 0.0303 | 0.0017 | 0.0106 | 0.0578 | 0.1100 | 0.0313 | 0.0039 | 2.9425 | 0.9512 | 0.5677 |
| Phi-3-mini | MTSamples | natural_language | 9 | 0.2812 | 0.0303 | 0.0017 | 0.0101 | 0.0582 | 0.1100 | 0.0314 | 0.0041 | 3.3914 | 0.9561 | 0.5209 |
| Phi-3-mini | MTSamples | natural_language | 10 | 0.3125 | 0.0323 | 0.0051 | 0.0131 | 0.0586 | 0.1300 | 0.0315 | 0.0043 | 3.7059 | 0.9571 | 0.4918 |
| Phi-3-mini | MTSamples | natural_language | 11 | 0.3438 | 0.0323 | 0.0051 | 0.0127 | 0.0598 | 0.1300 | 0.0317 | 0.0047 | 4.1274 | 0.9594 | 0.4601 |
| Phi-3-mini | MTSamples | natural_language | 12 | 0.3750 | 0.0328 | 0.0060 | 0.0128 | 0.0606 | 0.1300 | 0.0311 | 0.0036 | 4.7594 | 0.9586 | 0.4145 |
| Phi-3-mini | MTSamples | natural_language | 13 | 0.4062 | 0.0338 | 0.0077 | 0.0136 | 0.0603 | 0.1400 | 0.0312 | 0.0037 | 5.3029 | 0.9588 | 0.3845 |
| Phi-3-mini | MTSamples | natural_language | 14 | 0.4375 | 0.0331 | 0.0065 | 0.0116 | 0.0594 | 0.1100 | 0.0321 | 0.0055 | 6.4930 | 0.9691 | 0.3405 |
| Phi-3-mini | MTSamples | natural_language | 15 | 0.4688 | 0.0337 | 0.0075 | 0.0123 | 0.0602 | 0.1100 | 0.0330 | 0.0071 | 7.2702 | 0.9684 | 0.3048 |
| Phi-3-mini | MTSamples | natural_language | 16 | 0.5000 | 0.0350 | 0.0097 | 0.0130 | 0.0632 | 0.1200 | 0.0325 | 0.0063 | 7.9113 | 0.9675 | 0.2795 |
| Phi-3-mini | MTSamples | natural_language | 17 | 0.5312 | 0.0351 | 0.0099 | 0.0137 | 0.0646 | 0.1200 | 0.0335 | 0.0081 | 8.3587 | 0.9668 | 0.2618 |
| Phi-3-mini | MTSamples | natural_language | 18 | 0.5625 | 0.0446 | 0.0261 | 0.0167 | 0.0794 | 0.1300 | 0.0362 | 0.0134 | 9.6634 | 0.9618 | 0.2394 |
| Phi-3-mini | MTSamples | natural_language | 19 | 0.5938 | 0.0498 | 0.0350 | 0.0224 | 0.0856 | 0.1600 | 0.0399 | 0.0203 | 10.5382 | 0.9600 | 0.2363 |
| Phi-3-mini | MTSamples | natural_language | 20 | 0.6250 | 0.0513 | 0.0375 | 0.0233 | 0.0857 | 0.1700 | 0.0403 | 0.0211 | 10.9121 | 0.9610 | 0.2298 |
| Phi-3-mini | MTSamples | natural_language | 21 | 0.6562 | 0.0519 | 0.0386 | 0.0246 | 0.0882 | 0.1600 | 0.0443 | 0.0288 | 11.2292 | 0.9583 | 0.2352 |
| Phi-3-mini | MTSamples | natural_language | 22 | 0.6875 | 0.0561 | 0.0457 | 0.0278 | 0.0924 | 0.1700 | 0.0496 | 0.0389 | 10.8722 | 0.9578 | 0.2473 |
| Phi-3-mini | MTSamples | natural_language | 23 | 0.7188 | 0.1011 | 0.1225 | 0.0580 | 0.1477 | 0.2900 | 0.0816 | 0.1003 | 10.0515 | 0.9427 | 0.2713 |
| Phi-3-mini | MTSamples | natural_language | 24 | 0.7500 | 0.1375 | 0.1847 | 0.0921 | 0.1889 | 0.4300 | 0.1166 | 0.1672 | 10.9640 | 0.9360 | 0.2537 |
| Phi-3-mini | MTSamples | natural_language | 25 | 0.7812 | 0.1925 | 0.2785 | 0.1399 | 0.2498 | 0.5300 | 0.1832 | 0.2947 | 10.7372 | 0.9280 | 0.2575 |
| Phi-3-mini | MTSamples | natural_language | 26 | 0.8125 | 0.2091 | 0.3069 | 0.1554 | 0.2697 | 0.5400 | 0.2006 | 0.3280 | 11.0064 | 0.9353 | 0.2528 |
| Phi-3-mini | MTSamples | natural_language | 27 | 0.8438 | 0.2396 | 0.3589 | 0.1803 | 0.3010 | 0.6000 | 0.2244 | 0.3736 | 10.8788 | 0.9342 | 0.2550 |
| Phi-3-mini | MTSamples | natural_language | 28 | 0.8750 | 0.2633 | 0.3994 | 0.2005 | 0.3227 | 0.6300 | 0.2389 | 0.4015 | 10.9800 | 0.9404 | 0.2549 |
| Phi-3-mini | MTSamples | natural_language | 29 | 0.9062 | 0.2770 | 0.4228 | 0.2159 | 0.3422 | 0.6200 | 0.2532 | 0.4287 | 11.4821 | 0.9425 | 0.2481 |
| Phi-3-mini | MTSamples | natural_language | 30 | 0.9375 | 0.3672 | 0.5767 | 0.2885 | 0.4422 | 0.6700 | 0.4371 | 0.7809 | 11.6639 | 0.9478 | 0.2470 |
| Phi-3-mini | MTSamples | natural_language | 31 | 0.9688 | 0.4056 | 0.6423 | 0.3289 | 0.4792 | 0.7000 | 0.4482 | 0.8021 | 12.4429 | 0.9567 | 0.2354 |
| Phi-3-mini | MTSamples | natural_language | 32 | 1.0000 | 0.5409 | 0.8732 | 0.4572 | 0.6248 | 0.7500 | 0.5216 | 0.9427 | 12.5823 | 0.9736 | 0.2300 |
| Phi-3-mini | PMC-Patients | keyword | 0 | 0.0000 | 0.4074 | 0.6955 | 0.3255 | 0.4886 | 0.6200 | 0.5515 | 1.0000 | 34.9583 | 0.7118 | 0.1207 |
| Phi-3-mini | PMC-Patients | keyword | 1 | 0.0312 | 0.1332 | 0.1911 | 0.0800 | 0.1920 | 0.2600 | 0.2377 | 0.3991 | 11.2125 | 0.9958 | 0.2684 |
| Phi-3-mini | PMC-Patients | keyword | 2 | 0.0625 | 0.0586 | 0.0539 | 0.0280 | 0.0969 | 0.2000 | 0.1154 | 0.1648 | 8.0509 | 0.9935 | 0.2893 |
| Phi-3-mini | PMC-Patients | keyword | 3 | 0.0938 | 0.0293 | 0.0000 | 0.0098 | 0.0558 | 0.1000 | 0.0293 | 0.0000 | 1.2506 | 0.9631 | 0.8936 |
| Phi-3-mini | PMC-Patients | keyword | 4 | 0.1250 | 0.0298 | 0.0009 | 0.0094 | 0.0570 | 0.1000 | 0.0296 | 0.0007 | 1.4077 | 0.9581 | 0.8419 |
| Phi-3-mini | PMC-Patients | keyword | 5 | 0.1562 | 0.0319 | 0.0048 | 0.0105 | 0.0615 | 0.1000 | 0.0307 | 0.0028 | 1.3499 | 0.9560 | 0.8600 |
| Phi-3-mini | PMC-Patients | keyword | 6 | 0.1875 | 0.0294 | 0.0002 | 0.0099 | 0.0565 | 0.1000 | 0.0304 | 0.0023 | 1.5450 | 0.9520 | 0.8032 |
| Phi-3-mini | PMC-Patients | keyword | 7 | 0.2188 | 0.0308 | 0.0028 | 0.0102 | 0.0578 | 0.1000 | 0.0304 | 0.0022 | 1.7634 | 0.9488 | 0.7510 |
| Phi-3-mini | PMC-Patients | keyword | 8 | 0.2500 | 0.0303 | 0.0018 | 0.0112 | 0.0563 | 0.1000 | 0.0313 | 0.0039 | 2.5292 | 0.9479 | 0.6232 |
| Phi-3-mini | PMC-Patients | keyword | 9 | 0.2812 | 0.0293 | 0.0000 | 0.0100 | 0.0546 | 0.1000 | 0.0314 | 0.0041 | 2.9371 | 0.9540 | 0.5762 |
| Phi-3-mini | PMC-Patients | keyword | 10 | 0.3125 | 0.0293 | 0.0000 | 0.0097 | 0.0565 | 0.1000 | 0.0315 | 0.0043 | 3.1381 | 0.9561 | 0.5565 |
| Phi-3-mini | PMC-Patients | keyword | 11 | 0.3438 | 0.0298 | 0.0009 | 0.0100 | 0.0557 | 0.1000 | 0.0317 | 0.0047 | 3.5582 | 0.9595 | 0.5202 |
| Phi-3-mini | PMC-Patients | keyword | 12 | 0.3750 | 0.0299 | 0.0011 | 0.0098 | 0.0577 | 0.1000 | 0.0311 | 0.0036 | 4.1883 | 0.9611 | 0.4755 |
| Phi-3-mini | PMC-Patients | keyword | 13 | 0.4062 | 0.0298 | 0.0009 | 0.0092 | 0.0565 | 0.1000 | 0.0312 | 0.0037 | 4.6543 | 0.9620 | 0.4482 |
| Phi-3-mini | PMC-Patients | keyword | 14 | 0.4375 | 0.0298 | 0.0009 | 0.0096 | 0.0570 | 0.1000 | 0.0321 | 0.0055 | 7.5518 | 0.9722 | 0.3282 |
| Phi-3-mini | PMC-Patients | keyword | 15 | 0.4688 | 0.0306 | 0.0024 | 0.0107 | 0.0577 | 0.1000 | 0.0330 | 0.0071 | 8.7912 | 0.9728 | 0.2926 |
| Phi-3-mini | PMC-Patients | keyword | 16 | 0.5000 | 0.0308 | 0.0028 | 0.0107 | 0.0597 | 0.1000 | 0.0325 | 0.0063 | 10.1431 | 0.9718 | 0.2627 |
| Phi-3-mini | PMC-Patients | keyword | 17 | 0.5312 | 0.0322 | 0.0053 | 0.0112 | 0.0604 | 0.1000 | 0.0335 | 0.0081 | 11.1631 | 0.9711 | 0.2402 |
| Phi-3-mini | PMC-Patients | keyword | 18 | 0.5625 | 0.0331 | 0.0070 | 0.0114 | 0.0595 | 0.1000 | 0.0362 | 0.0134 | 15.3188 | 0.9653 | 0.1824 |
| Phi-3-mini | PMC-Patients | keyword | 19 | 0.5938 | 0.0340 | 0.0086 | 0.0135 | 0.0615 | 0.1000 | 0.0399 | 0.0203 | 18.5130 | 0.9628 | 0.1462 |
| Phi-3-mini | PMC-Patients | keyword | 20 | 0.6250 | 0.0334 | 0.0075 | 0.0115 | 0.0623 | 0.1000 | 0.0403 | 0.0211 | 18.3497 | 0.9652 | 0.1479 |
| Phi-3-mini | PMC-Patients | keyword | 21 | 0.6562 | 0.0343 | 0.0092 | 0.0129 | 0.0600 | 0.1000 | 0.0443 | 0.0288 | 20.6010 | 0.9623 | 0.1272 |
| Phi-3-mini | PMC-Patients | keyword | 22 | 0.6875 | 0.0407 | 0.0210 | 0.0132 | 0.0640 | 0.1000 | 0.0496 | 0.0389 | 21.2622 | 0.9602 | 0.1221 |
| Phi-3-mini | PMC-Patients | keyword | 23 | 0.7188 | 0.0697 | 0.0743 | 0.0320 | 0.1140 | 0.1600 | 0.0816 | 0.1003 | 22.6782 | 0.9429 | 0.1128 |
| Phi-3-mini | PMC-Patients | keyword | 24 | 0.7500 | 0.0965 | 0.1236 | 0.0517 | 0.1465 | 0.2500 | 0.1166 | 0.1672 | 23.1638 | 0.9360 | 0.1115 |
| Phi-3-mini | PMC-Patients | keyword | 25 | 0.7812 | 0.1497 | 0.2215 | 0.0964 | 0.2095 | 0.3700 | 0.1832 | 0.2947 | 22.4315 | 0.9294 | 0.1140 |
| Phi-3-mini | PMC-Patients | keyword | 26 | 0.8125 | 0.1699 | 0.2586 | 0.1134 | 0.2330 | 0.4700 | 0.2006 | 0.3280 | 22.7629 | 0.9353 | 0.1123 |
| Phi-3-mini | PMC-Patients | keyword | 27 | 0.8438 | 0.1916 | 0.2986 | 0.1399 | 0.2491 | 0.5600 | 0.2244 | 0.3736 | 22.5831 | 0.9358 | 0.1165 |
| Phi-3-mini | PMC-Patients | keyword | 28 | 0.8750 | 0.2020 | 0.3177 | 0.1472 | 0.2659 | 0.5500 | 0.2389 | 0.4015 | 22.8324 | 0.9440 | 0.1171 |
| Phi-3-mini | PMC-Patients | keyword | 29 | 0.9062 | 0.2048 | 0.3228 | 0.1490 | 0.2660 | 0.5700 | 0.2532 | 0.4287 | 23.8984 | 0.9479 | 0.1138 |
| Phi-3-mini | PMC-Patients | keyword | 30 | 0.9375 | 0.4900 | 0.8475 | 0.4167 | 0.5696 | 0.8400 | 0.4371 | 0.7809 | 24.5844 | 0.9533 | 0.1145 |
| Phi-3-mini | PMC-Patients | keyword | 31 | 0.9688 | 0.4512 | 0.7761 | 0.3802 | 0.5208 | 0.8300 | 0.4482 | 0.8021 | 25.2836 | 0.9613 | 0.1125 |
| Phi-3-mini | PMC-Patients | keyword | 32 | 1.0000 | 0.5729 | 1.0000 | 0.4930 | 0.6565 | 0.8700 | 0.5216 | 0.9427 | 25.2473 | 0.9743 | 0.1098 |
| Phi-3-mini | PMC-Patients | natural_language | 0 | 0.0000 | 0.6544 | 0.9956 | 0.5734 | 0.7331 | 0.8700 | 0.5515 | 1.0000 | 34.9583 | 0.7118 | 0.1207 |
| Phi-3-mini | PMC-Patients | natural_language | 1 | 0.0312 | 0.2359 | 0.3312 | 0.1639 | 0.3110 | 0.4200 | 0.2377 | 0.3991 | 11.2125 | 0.9958 | 0.2684 |
| Phi-3-mini | PMC-Patients | natural_language | 2 | 0.0625 | 0.0804 | 0.0843 | 0.0375 | 0.1291 | 0.1900 | 0.1154 | 0.1648 | 8.0509 | 0.9935 | 0.2893 |
| Phi-3-mini | PMC-Patients | natural_language | 3 | 0.0938 | 0.0273 | 0.0000 | 0.0096 | 0.0522 | 0.1000 | 0.0293 | 0.0000 | 1.2506 | 0.9631 | 0.8936 |
| Phi-3-mini | PMC-Patients | natural_language | 4 | 0.1250 | 0.0300 | 0.0043 | 0.0092 | 0.0564 | 0.1000 | 0.0296 | 0.0007 | 1.4077 | 0.9581 | 0.8419 |
| Phi-3-mini | PMC-Patients | natural_language | 5 | 0.1562 | 0.0319 | 0.0073 | 0.0106 | 0.0613 | 0.1000 | 0.0307 | 0.0028 | 1.3499 | 0.9560 | 0.8600 |
| Phi-3-mini | PMC-Patients | natural_language | 6 | 0.1875 | 0.0303 | 0.0048 | 0.0106 | 0.0569 | 0.1000 | 0.0304 | 0.0023 | 1.5450 | 0.9520 | 0.8032 |
| Phi-3-mini | PMC-Patients | natural_language | 7 | 0.2188 | 0.0308 | 0.0056 | 0.0101 | 0.0581 | 0.1000 | 0.0304 | 0.0022 | 1.7634 | 0.9488 | 0.7510 |
| Phi-3-mini | PMC-Patients | natural_language | 8 | 0.2500 | 0.0319 | 0.0073 | 0.0107 | 0.0603 | 0.1000 | 0.0313 | 0.0039 | 2.5292 | 0.9479 | 0.6232 |
| Phi-3-mini | PMC-Patients | natural_language | 9 | 0.2812 | 0.0304 | 0.0049 | 0.0106 | 0.0599 | 0.1000 | 0.0314 | 0.0041 | 2.9371 | 0.9540 | 0.5762 |
| Phi-3-mini | PMC-Patients | natural_language | 10 | 0.3125 | 0.0300 | 0.0043 | 0.0102 | 0.0588 | 0.1000 | 0.0315 | 0.0043 | 3.1381 | 0.9561 | 0.5565 |
| Phi-3-mini | PMC-Patients | natural_language | 11 | 0.3438 | 0.0303 | 0.0048 | 0.0097 | 0.0567 | 0.1000 | 0.0317 | 0.0047 | 3.5582 | 0.9595 | 0.5202 |
| Phi-3-mini | PMC-Patients | natural_language | 12 | 0.3750 | 0.0309 | 0.0057 | 0.0107 | 0.0571 | 0.1000 | 0.0311 | 0.0036 | 4.1883 | 0.9611 | 0.4755 |
| Phi-3-mini | PMC-Patients | natural_language | 13 | 0.4062 | 0.0308 | 0.0056 | 0.0099 | 0.0569 | 0.1000 | 0.0312 | 0.0037 | 4.6543 | 0.9620 | 0.4482 |
| Phi-3-mini | PMC-Patients | natural_language | 14 | 0.4375 | 0.0314 | 0.0065 | 0.0102 | 0.0573 | 0.1000 | 0.0321 | 0.0055 | 7.5518 | 0.9722 | 0.3282 |
| Phi-3-mini | PMC-Patients | natural_language | 15 | 0.4688 | 0.0344 | 0.0113 | 0.0130 | 0.0638 | 0.1000 | 0.0330 | 0.0071 | 8.7912 | 0.9728 | 0.2926 |
| Phi-3-mini | PMC-Patients | natural_language | 16 | 0.5000 | 0.0330 | 0.0090 | 0.0123 | 0.0620 | 0.1000 | 0.0325 | 0.0063 | 10.1431 | 0.9718 | 0.2627 |
| Phi-3-mini | PMC-Patients | natural_language | 17 | 0.5312 | 0.0341 | 0.0108 | 0.0129 | 0.0635 | 0.1000 | 0.0335 | 0.0081 | 11.1631 | 0.9711 | 0.2402 |
| Phi-3-mini | PMC-Patients | natural_language | 18 | 0.5625 | 0.0364 | 0.0144 | 0.0153 | 0.0629 | 0.1100 | 0.0362 | 0.0134 | 15.3188 | 0.9653 | 0.1824 |
| Phi-3-mini | PMC-Patients | natural_language | 19 | 0.5938 | 0.0439 | 0.0264 | 0.0198 | 0.0718 | 0.1500 | 0.0399 | 0.0203 | 18.5130 | 0.9628 | 0.1462 |
| Phi-3-mini | PMC-Patients | natural_language | 20 | 0.6250 | 0.0427 | 0.0244 | 0.0197 | 0.0729 | 0.1500 | 0.0403 | 0.0211 | 18.3497 | 0.9652 | 0.1479 |
| Phi-3-mini | PMC-Patients | natural_language | 21 | 0.6562 | 0.0584 | 0.0494 | 0.0284 | 0.0959 | 0.1500 | 0.0443 | 0.0288 | 20.6010 | 0.9623 | 0.1272 |
| Phi-3-mini | PMC-Patients | natural_language | 22 | 0.6875 | 0.0698 | 0.0675 | 0.0358 | 0.1069 | 0.1800 | 0.0496 | 0.0389 | 21.2622 | 0.9602 | 0.1221 |
| Phi-3-mini | PMC-Patients | natural_language | 23 | 0.7188 | 0.1310 | 0.1646 | 0.0793 | 0.1877 | 0.3000 | 0.0816 | 0.1003 | 22.6782 | 0.9429 | 0.1128 |
| Phi-3-mini | PMC-Patients | natural_language | 24 | 0.7500 | 0.1872 | 0.2538 | 0.1249 | 0.2535 | 0.4400 | 0.1166 | 0.1672 | 23.1638 | 0.9360 | 0.1115 |
| Phi-3-mini | PMC-Patients | natural_language | 25 | 0.7812 | 0.2823 | 0.4048 | 0.2144 | 0.3563 | 0.6600 | 0.1832 | 0.2947 | 22.4315 | 0.9294 | 0.1140 |
| Phi-3-mini | PMC-Patients | natural_language | 26 | 0.8125 | 0.2801 | 0.4013 | 0.2158 | 0.3516 | 0.6800 | 0.2006 | 0.3280 | 22.7629 | 0.9353 | 0.1123 |
| Phi-3-mini | PMC-Patients | natural_language | 27 | 0.8438 | 0.3100 | 0.4488 | 0.2428 | 0.3817 | 0.7300 | 0.2244 | 0.3736 | 22.5831 | 0.9358 | 0.1165 |
| Phi-3-mini | PMC-Patients | natural_language | 28 | 0.8750 | 0.3254 | 0.4732 | 0.2560 | 0.3987 | 0.7300 | 0.2389 | 0.4015 | 22.8324 | 0.9440 | 0.1171 |
| Phi-3-mini | PMC-Patients | natural_language | 29 | 0.9062 | 0.3313 | 0.4826 | 0.2625 | 0.4051 | 0.7200 | 0.2532 | 0.4287 | 23.8984 | 0.9479 | 0.1138 |
| Phi-3-mini | PMC-Patients | natural_language | 30 | 0.9375 | 0.3444 | 0.5034 | 0.2759 | 0.4162 | 0.7100 | 0.4371 | 0.7809 | 24.5844 | 0.9533 | 0.1145 |
| Phi-3-mini | PMC-Patients | natural_language | 31 | 0.9688 | 0.3341 | 0.4871 | 0.2723 | 0.4057 | 0.7000 | 0.4482 | 0.8021 | 25.2836 | 0.9613 | 0.1125 |
| Phi-3-mini | PMC-Patients | natural_language | 32 | 1.0000 | 0.6572 | 1.0000 | 0.5764 | 0.7363 | 0.8700 | 0.5216 | 0.9427 | 25.2473 | 0.9743 | 0.1098 |
| Phi-3-mini | Synthetic | keyword | 0 | 0.0000 | 0.5223 | 0.9293 | 0.4402 | 0.6071 | 0.7700 | 0.5515 | 1.0000 | 19.9905 | 0.8097 | 0.1732 |
| Phi-3-mini | Synthetic | keyword | 1 | 0.0312 | 0.2399 | 0.3968 | 0.1800 | 0.3077 | 0.5300 | 0.2377 | 0.3991 | 8.2260 | 0.9978 | 0.3267 |
| Phi-3-mini | Synthetic | keyword | 2 | 0.0625 | 0.1788 | 0.2815 | 0.1208 | 0.2416 | 0.4300 | 0.1154 | 0.1648 | 5.7920 | 0.9968 | 0.4003 |
| Phi-3-mini | Synthetic | keyword | 3 | 0.0938 | 0.0302 | 0.0013 | 0.0093 | 0.0573 | 0.1000 | 0.0293 | 0.0000 | 4.2867 | 0.9963 | 0.4455 |
| Phi-3-mini | Synthetic | keyword | 4 | 0.1250 | 0.0295 | 0.0000 | 0.0104 | 0.0579 | 0.1000 | 0.0296 | 0.0007 | 6.4478 | 0.9949 | 0.3507 |
| Phi-3-mini | Synthetic | keyword | 5 | 0.1562 | 0.0303 | 0.0015 | 0.0106 | 0.0575 | 0.1100 | 0.0307 | 0.0028 | 8.2504 | 0.9957 | 0.3025 |
| Phi-3-mini | Synthetic | keyword | 6 | 0.1875 | 0.0314 | 0.0036 | 0.0119 | 0.0590 | 0.1200 | 0.0304 | 0.0023 | 10.6938 | 0.9935 | 0.2510 |
| Phi-3-mini | Synthetic | keyword | 7 | 0.2188 | 0.0309 | 0.0026 | 0.0108 | 0.0592 | 0.1100 | 0.0304 | 0.0022 | 11.2255 | 0.9913 | 0.2434 |
| Phi-3-mini | Synthetic | keyword | 8 | 0.2500 | 0.0315 | 0.0038 | 0.0111 | 0.0589 | 0.1100 | 0.0313 | 0.0039 | 12.2533 | 0.9881 | 0.2101 |
| Phi-3-mini | Synthetic | keyword | 9 | 0.2812 | 0.0338 | 0.0081 | 0.0139 | 0.0586 | 0.1400 | 0.0314 | 0.0041 | 12.0403 | 0.9890 | 0.2203 |
| Phi-3-mini | Synthetic | keyword | 10 | 0.3125 | 0.0329 | 0.0064 | 0.0123 | 0.0621 | 0.1300 | 0.0315 | 0.0043 | 11.6436 | 0.9894 | 0.2346 |
| Phi-3-mini | Synthetic | keyword | 11 | 0.3438 | 0.0333 | 0.0072 | 0.0138 | 0.0618 | 0.1300 | 0.0317 | 0.0047 | 11.2915 | 0.9889 | 0.2393 |
| Phi-3-mini | Synthetic | keyword | 12 | 0.3750 | 0.0296 | 0.0002 | 0.0089 | 0.0560 | 0.1000 | 0.0311 | 0.0036 | 10.8188 | 0.9886 | 0.2504 |
| Phi-3-mini | Synthetic | keyword | 13 | 0.4062 | 0.0300 | 0.0009 | 0.0100 | 0.0578 | 0.1000 | 0.0312 | 0.0037 | 10.5096 | 0.9887 | 0.2587 |
| Phi-3-mini | Synthetic | keyword | 14 | 0.4375 | 0.0330 | 0.0066 | 0.0122 | 0.0618 | 0.1100 | 0.0321 | 0.0055 | 6.4601 | 0.9891 | 0.3377 |
| Phi-3-mini | Synthetic | keyword | 15 | 0.4688 | 0.0337 | 0.0079 | 0.0114 | 0.0603 | 0.1000 | 0.0330 | 0.0071 | 7.0034 | 0.9895 | 0.3081 |
| Phi-3-mini | Synthetic | keyword | 16 | 0.5000 | 0.0312 | 0.0032 | 0.0104 | 0.0580 | 0.1000 | 0.0325 | 0.0063 | 8.0911 | 0.9890 | 0.2646 |
| Phi-3-mini | Synthetic | keyword | 17 | 0.5312 | 0.0338 | 0.0081 | 0.0132 | 0.0616 | 0.1100 | 0.0335 | 0.0081 | 8.3019 | 0.9894 | 0.2497 |
| Phi-3-mini | Synthetic | keyword | 18 | 0.5625 | 0.0349 | 0.0102 | 0.0139 | 0.0632 | 0.1200 | 0.0362 | 0.0134 | 11.4524 | 0.9858 | 0.1941 |
| Phi-3-mini | Synthetic | keyword | 19 | 0.5938 | 0.0395 | 0.0189 | 0.0167 | 0.0674 | 0.1400 | 0.0399 | 0.0203 | 12.6600 | 0.9840 | 0.1938 |
| Phi-3-mini | Synthetic | keyword | 20 | 0.6250 | 0.0409 | 0.0215 | 0.0175 | 0.0689 | 0.1400 | 0.0403 | 0.0211 | 12.7284 | 0.9845 | 0.1962 |
| Phi-3-mini | Synthetic | keyword | 21 | 0.6562 | 0.0440 | 0.0273 | 0.0210 | 0.0757 | 0.1200 | 0.0443 | 0.0288 | 13.6077 | 0.9824 | 0.1881 |
| Phi-3-mini | Synthetic | keyword | 22 | 0.6875 | 0.0505 | 0.0396 | 0.0258 | 0.0813 | 0.1500 | 0.0496 | 0.0389 | 13.2383 | 0.9821 | 0.1998 |
| Phi-3-mini | Synthetic | keyword | 23 | 0.7188 | 0.0573 | 0.0524 | 0.0320 | 0.0870 | 0.1700 | 0.0816 | 0.1003 | 14.0615 | 0.9743 | 0.1751 |
| Phi-3-mini | Synthetic | keyword | 24 | 0.7500 | 0.0823 | 0.0996 | 0.0474 | 0.1238 | 0.2300 | 0.1166 | 0.1672 | 13.8434 | 0.9689 | 0.1700 |
| Phi-3-mini | Synthetic | keyword | 25 | 0.7812 | 0.1490 | 0.2253 | 0.0971 | 0.2118 | 0.3500 | 0.1832 | 0.2947 | 13.7733 | 0.9645 | 0.1676 |
| Phi-3-mini | Synthetic | keyword | 26 | 0.8125 | 0.1832 | 0.2898 | 0.1247 | 0.2466 | 0.3600 | 0.2006 | 0.3280 | 13.9039 | 0.9679 | 0.1677 |
| Phi-3-mini | Synthetic | keyword | 27 | 0.8438 | 0.2099 | 0.3402 | 0.1490 | 0.2798 | 0.4300 | 0.2244 | 0.3736 | 14.0139 | 0.9682 | 0.1658 |
| Phi-3-mini | Synthetic | keyword | 28 | 0.8750 | 0.2230 | 0.3649 | 0.1585 | 0.2889 | 0.4600 | 0.2389 | 0.4015 | 14.4074 | 0.9723 | 0.1621 |
| Phi-3-mini | Synthetic | keyword | 29 | 0.9062 | 0.2541 | 0.4235 | 0.1845 | 0.3264 | 0.5300 | 0.2532 | 0.4287 | 14.7545 | 0.9753 | 0.1612 |
| Phi-3-mini | Synthetic | keyword | 30 | 0.9375 | 0.5422 | 0.9668 | 0.4634 | 0.6169 | 0.8500 | 0.4371 | 0.7809 | 15.0993 | 0.9790 | 0.1585 |
| Phi-3-mini | Synthetic | keyword | 31 | 0.9688 | 0.5598 | 1.0000 | 0.4821 | 0.6364 | 0.8500 | 0.4482 | 0.8021 | 15.1863 | 0.9827 | 0.1618 |
| Phi-3-mini | Synthetic | keyword | 32 | 1.0000 | 0.4193 | 0.7351 | 0.3471 | 0.4994 | 0.7400 | 0.5216 | 0.9427 | 15.1520 | 0.9748 | 0.1655 |
| Phi-3-mini | Synthetic | natural_language | 0 | 0.0000 | 0.6465 | 1.0000 | 0.5652 | 0.7242 | 0.8600 | 0.5515 | 1.0000 | 19.9905 | 0.8097 | 0.1732 |
| Phi-3-mini | Synthetic | natural_language | 1 | 0.0312 | 0.4257 | 0.6421 | 0.3326 | 0.4971 | 0.7000 | 0.2377 | 0.3991 | 8.2260 | 0.9978 | 0.3267 |
| Phi-3-mini | Synthetic | natural_language | 2 | 0.0625 | 0.2492 | 0.3560 | 0.1854 | 0.3195 | 0.5500 | 0.1154 | 0.1648 | 5.7920 | 0.9968 | 0.4003 |
| Phi-3-mini | Synthetic | natural_language | 3 | 0.0938 | 0.0302 | 0.0010 | 0.0107 | 0.0572 | 0.1000 | 0.0293 | 0.0000 | 4.2867 | 0.9963 | 0.4455 |
| Phi-3-mini | Synthetic | natural_language | 4 | 0.1250 | 0.0296 | 0.0000 | 0.0101 | 0.0568 | 0.1000 | 0.0296 | 0.0007 | 6.4478 | 0.9949 | 0.3507 |
| Phi-3-mini | Synthetic | natural_language | 5 | 0.1562 | 0.0315 | 0.0031 | 0.0122 | 0.0585 | 0.1200 | 0.0307 | 0.0028 | 8.2504 | 0.9957 | 0.3025 |
| Phi-3-mini | Synthetic | natural_language | 6 | 0.1875 | 0.0330 | 0.0055 | 0.0127 | 0.0593 | 0.1300 | 0.0304 | 0.0023 | 10.6938 | 0.9935 | 0.2510 |
| Phi-3-mini | Synthetic | natural_language | 7 | 0.2188 | 0.0312 | 0.0026 | 0.0112 | 0.0576 | 0.1100 | 0.0304 | 0.0022 | 11.2255 | 0.9913 | 0.2434 |
| Phi-3-mini | Synthetic | natural_language | 8 | 0.2500 | 0.0335 | 0.0063 | 0.0129 | 0.0601 | 0.1200 | 0.0313 | 0.0039 | 12.2533 | 0.9881 | 0.2101 |
| Phi-3-mini | Synthetic | natural_language | 9 | 0.2812 | 0.0353 | 0.0092 | 0.0150 | 0.0633 | 0.1500 | 0.0314 | 0.0041 | 12.0403 | 0.9890 | 0.2203 |
| Phi-3-mini | Synthetic | natural_language | 10 | 0.3125 | 0.0342 | 0.0075 | 0.0146 | 0.0604 | 0.1400 | 0.0315 | 0.0043 | 11.6436 | 0.9894 | 0.2346 |
| Phi-3-mini | Synthetic | natural_language | 11 | 0.3438 | 0.0352 | 0.0091 | 0.0157 | 0.0619 | 0.1500 | 0.0317 | 0.0047 | 11.2915 | 0.9889 | 0.2393 |
| Phi-3-mini | Synthetic | natural_language | 12 | 0.3750 | 0.0343 | 0.0076 | 0.0136 | 0.0638 | 0.1300 | 0.0311 | 0.0036 | 10.8188 | 0.9886 | 0.2504 |
| Phi-3-mini | Synthetic | natural_language | 13 | 0.4062 | 0.0336 | 0.0065 | 0.0134 | 0.0589 | 0.1300 | 0.0312 | 0.0037 | 10.5096 | 0.9887 | 0.2587 |
| Phi-3-mini | Synthetic | natural_language | 14 | 0.4375 | 0.0360 | 0.0104 | 0.0143 | 0.0650 | 0.1000 | 0.0321 | 0.0055 | 6.4601 | 0.9891 | 0.3377 |
| Phi-3-mini | Synthetic | natural_language | 15 | 0.4688 | 0.0375 | 0.0128 | 0.0158 | 0.0667 | 0.1000 | 0.0330 | 0.0071 | 7.0034 | 0.9895 | 0.3081 |
| Phi-3-mini | Synthetic | natural_language | 16 | 0.5000 | 0.0368 | 0.0117 | 0.0131 | 0.0666 | 0.1000 | 0.0325 | 0.0063 | 8.0911 | 0.9890 | 0.2646 |
| Phi-3-mini | Synthetic | natural_language | 17 | 0.5312 | 0.0376 | 0.0130 | 0.0149 | 0.0676 | 0.1100 | 0.0335 | 0.0081 | 8.3019 | 0.9894 | 0.2497 |
| Phi-3-mini | Synthetic | natural_language | 18 | 0.5625 | 0.0405 | 0.0177 | 0.0176 | 0.0708 | 0.1200 | 0.0362 | 0.0134 | 11.4524 | 0.9858 | 0.1941 |
| Phi-3-mini | Synthetic | natural_language | 19 | 0.5938 | 0.0436 | 0.0227 | 0.0195 | 0.0737 | 0.1400 | 0.0399 | 0.0203 | 12.6600 | 0.9840 | 0.1938 |
| Phi-3-mini | Synthetic | natural_language | 20 | 0.6250 | 0.0458 | 0.0263 | 0.0218 | 0.0765 | 0.1300 | 0.0403 | 0.0211 | 12.7284 | 0.9845 | 0.1962 |
| Phi-3-mini | Synthetic | natural_language | 21 | 0.6562 | 0.0479 | 0.0297 | 0.0220 | 0.0773 | 0.1400 | 0.0443 | 0.0288 | 13.6077 | 0.9824 | 0.1881 |
| Phi-3-mini | Synthetic | natural_language | 22 | 0.6875 | 0.0501 | 0.0332 | 0.0259 | 0.0781 | 0.1700 | 0.0496 | 0.0389 | 13.2383 | 0.9821 | 0.1998 |
| Phi-3-mini | Synthetic | natural_language | 23 | 0.7188 | 0.0921 | 0.1013 | 0.0542 | 0.1345 | 0.2800 | 0.0816 | 0.1003 | 14.0615 | 0.9743 | 0.1751 |
| Phi-3-mini | Synthetic | natural_language | 24 | 0.7500 | 0.1450 | 0.1871 | 0.0957 | 0.1981 | 0.4000 | 0.1166 | 0.1672 | 13.8434 | 0.9689 | 0.1700 |
| Phi-3-mini | Synthetic | natural_language | 25 | 0.7812 | 0.2467 | 0.3519 | 0.1790 | 0.3131 | 0.5600 | 0.1832 | 0.2947 | 13.7733 | 0.9645 | 0.1676 |
| Phi-3-mini | Synthetic | natural_language | 26 | 0.8125 | 0.2632 | 0.3787 | 0.1985 | 0.3305 | 0.6200 | 0.2006 | 0.3280 | 13.9039 | 0.9679 | 0.1677 |
| Phi-3-mini | Synthetic | natural_language | 27 | 0.8438 | 0.2807 | 0.4070 | 0.2198 | 0.3494 | 0.6700 | 0.2244 | 0.3736 | 14.0139 | 0.9682 | 0.1658 |
| Phi-3-mini | Synthetic | natural_language | 28 | 0.8750 | 0.2915 | 0.4245 | 0.2257 | 0.3580 | 0.6900 | 0.2389 | 0.4015 | 14.4074 | 0.9723 | 0.1621 |
| Phi-3-mini | Synthetic | natural_language | 29 | 0.9062 | 0.3115 | 0.4570 | 0.2426 | 0.3826 | 0.7200 | 0.2532 | 0.4287 | 14.7545 | 0.9753 | 0.1612 |
| Phi-3-mini | Synthetic | natural_language | 30 | 0.9375 | 0.4123 | 0.6204 | 0.3416 | 0.4849 | 0.7600 | 0.4371 | 0.7809 | 15.0993 | 0.9790 | 0.1585 |
| Phi-3-mini | Synthetic | natural_language | 31 | 0.9688 | 0.4321 | 0.6525 | 0.3591 | 0.5112 | 0.7600 | 0.4482 | 0.8021 | 15.1863 | 0.9827 | 0.1618 |
| Phi-3-mini | Synthetic | natural_language | 32 | 1.0000 | 0.3969 | 0.5954 | 0.3254 | 0.4747 | 0.7000 | 0.5216 | 0.9427 | 15.1520 | 0.9748 | 0.1655 |
