# Supplementary material for: Effects of Model Choice, Corpus Context, and Post Hoc Correction on Layer-Level Embedding Degradation in Clinical Document Retrieval: Experimental Study
Source: JMIR Med Inform. 2026 Jul 29;14:e99639. doi: 10.2196/99639 (PMC13469822; doi:10.2196/99639)
Supplement: Supplementary file 7 [file medinform_v14i1e99639_app7.docx]

# Multimedia Appendix 7 — Addendum

Per-model final-layer MRR@10 under each post-hoc intervention condition: baseline (no intervention), corpus-only ZCA whitening (fitted on document embeddings only, ε=10⁻⁵), and transductive ZCA whitening (fitted on the union of document and query embeddings, retained as a query-contaminated reference). All values averaged across 3 corpora × 2 query formats (n=6 per cell). Δ values relative to baseline shown in parentheses. Tier assignment per the matched-comparison analysis (Tier 1 = retrieval-trained; Tier 2 = non-retrieval-trained).

| Model | Baseline | Corpus-only ZCA | Transductive ZCA | Tier |
| --- | --- | --- | --- | --- |
| BERT-base | 0.250 | 0.286 (+0.037) | 0.415 (+0.165) | 2 |
| BioBERT | 0.396 | 0.459 (+0.063) | 0.585 (+0.188) | 2 |
| ClinicalBERT | 0.229 | 0.282 (+0.053) | 0.387 (+0.157) | 2 |
| BioMistral | 0.424 | 0.483 (+0.059) | 0.745 (+0.322) | 2 |
| Phi-3-mini | 0.523 | 0.562 (+0.039) | 0.795 (+0.272) | 2 |
| E5-Mistral-abl. | 0.663 | 0.648 (−0.016) | 0.790 (+0.127) | 2 |
| E5-Mistral | 0.318 | 0.521 (+0.203) | 0.583 (+0.265) | 2 |
| BioLORD | 0.798 | 0.720 (−0.079) | 0.694 (−0.104) | 1 |
| MedCPT | 0.816 | 0.681 (−0.135) | 0.545 (−0.270) | 1 |
| BGE-base | 0.872 | 0.799 (−0.073) | 0.737 (−0.135) | 1 |
| GTE-base | 0.879 | 0.810 (−0.069) | 0.746 (−0.133) | 1 |
| Nomic | 0.862 | 0.777 (−0.085) | 0.754 (−0.107) | 1 |
| Nomic-nopfx | 0.867 | 0.786 (−0.081) | 0.760 (−0.107) | 1 |

*Two-tier pattern is visible in corpus-only ZCA column: Tier 1 models show small negative or null Δ; Tier 2 models show substantial positive Δ. Transductive ZCA produces large positive Δ across all Tier 2 models, consistent with its access to test-query embedding information at the fit stage, but degrades every Tier 1 model — more severely than corpus-only ZCA in 5 of 6 cases.*
