# Supplementary material for: Effects of Model Choice, Corpus Context, and Post Hoc Correction on Layer-Level Embedding Degradation in Clinical Document Retrieval: Experimental Study
Source: JMIR Med Inform. 2026 Jul 29;14:e99639. doi: 10.2196/99639 (PMC13469822; doi:10.2196/99639)
Supplement: Supplementary file 8 [file medinform_v14i1e99639_app8.docx]

# Multimedia Appendix 8

Per-model layer-wise Spearman correlations between geometric measures (participation ratio, average pairwise cosine) and MRR@10 across layers, for the 11 configurations included in this full per-layer geometric correlation table. BERT-base-uncased and BioMistral-7B were extracted layer-wise for the Table 2 trajectory metrics and rel_layer LME analysis but were not included in this correlation table. Per-model values aggregate across 3 corpora × 2 query formats (length_tercile='all'). Correlations are Spearman ρ; P-values are two-sided.

| **Model** | **Layers (n)** | **PR vs MRR@10 (Spearman ρ)** | **PR-vs-MRR P-value** | **Cos vs MRR@10 (Spearman ρ)** | **Cos-vs-MRR P-value** | **PR stronger than \|cos\|** |
| --- | --- | --- | --- | --- | --- | --- |
| BGE-base | 13 | +0.973 | <.001 | -0.516 | 0.071 | Yes |
| BioBERT | 13 | +0.978 | <.001 | -0.066 | 0.831 | Yes |
| BioLORD-2023 | 13 | +0.940 | <.001 | -0.868 | <.001 | Yes |
| ClinicalBERT | 7 | +0.571 | 0.180 | -0.429 | 0.337 | Yes |
| E5-Mistral-7B | 33 | +0.358 | 0.041 | +0.488 | 0.004 | No |
| E5-Mistral-7B-ablation | 33 | +0.958 | <.001 | -0.649 | <.001 | Yes |
| GTE-base | 13 | +0.989 | <.001 | -0.462 | 0.112 | Yes |
| MedCPT | 13 | +0.016 | 0.957 | -0.060 | 0.845 | No |
| Nomic-embed-text | 13 | +0.802 | <.001 | -0.214 | 0.482 | Yes |
| Nomic-embed-text-nopfx | 13 | +0.819 | <.001 | -0.236 | 0.437 | Yes |
| Phi-3-mini | 33 | +0.959 | <.001 | -0.347 | 0.048 | Yes |

*Summary: PR-vs-MRR ρ was larger in absolute value than cos-vs-MRR ρ for 9 of 11 configurations (median PR-vs-MRR ρ = +0.939). The 2 exceptions are MedCPT (neither correlation significant due to the layer-0 dual-encoder artefact) and instruction-tuned E5-Mistral-7B (cos-vs-MRR ρ positive, opposite in sign to the other 10 configurations). Computation: pandas.groupby([model, layer]) → scipy.stats.spearmanr on per-model layer-wise means.*
