# Supplementary material for: Effects of Model Choice, Corpus Context, and Post Hoc Correction on Layer-Level Embedding Degradation in Clinical Document Retrieval: Experimental Study
Source: JMIR Med Inform. 2026 Jul 29;14:e99639. doi: 10.2196/99639 (PMC13469822; doi:10.2196/99639)
Supplement: Supplementary file 9 [file medinform_v14i1e99639_app9.docx]

# Multimedia Appendix 9

Distributional comparison of the three corpora used in the layer-level retrieval evaluation. Document length is measured as whitespace-tokenized word count; vocabulary size is the number of distinct lowercased word types; type-token ratio (TTR) is the vocabulary size divided by total token count. Values are approximate summary statistics computed over the 100-document working corpus per source. The Synthetic corpus exhibits approximately 30% shorter mean document length, 58% smaller vocabulary, and 40% lower type-token ratio than the natural-text corpora.

| **Corpus** | **Doc length (mean words)** | **Vocabulary size** | **Type-token ratio** |
| --- | --- | --- | --- |
| MTSamples | ~437 | ~8,400 | ~0.19 |
| PMC-Patients | ~414 | ~9,500 | ~0.23 |
| Synthetic | ~300 | ~3,500 | ~0.12 |

*These distributional differences are referenced in the Methods/Synthetic corpus distributional audit subsection of the manuscript and inform the interpretation of the synthetic-corpus ZCA failure: ZCA effectiveness depends on the covariance structure of the deployment corpus, and structurally uniform corpora (low TTR, small vocabulary, short documents) may produce ill-conditioned covariance estimates.*
